# Jaceosidin induces apoptosis and inhibits migration in AGS gastric cancer cells by regulating ROS-mediated signaling pathways

**DOI:** 10.1080/13510002.2024.2313366

**Published:** 2024-02-06

**Authors:** Jian Liu, Shu-Mei Li, Yan-Jun Tang, Jing-Long Cao, Wen-Shuang Hou, An-Qi Wang, Chang Wang, Cheng-Hao Jin

**Affiliations:** a College of Life Science and Technology, Heilongjiang Bayi Agricultural University, Daqing, People’s Republic of China; b Hemodialysis Center, Daqing Oilfield General Hospital, Daqing, People’s Republic of China; c College of Food Science and Technology, Heilongjiang Bayi Agricultural University, Daqing, People’s Republic of China; d College of Science, Heilongjiang Bayi Agricultural University, Daqing, People’s Republic of China; e National Coarse Cereals Engineering Research Center, Daqing, People’s Republic of China

**Keywords:** Jaceosidin, gastric cancer, reactive oxygen species, cell apoptosis, cell cycle, cell migration, cytotoxicity, network pharmacology

## Abstract

Jaceosidin (JAC) is a natural flavonoid with anti-oxidant and other pharmacological activities; however, its anti-cancer mechanism remains unclear. We investigated the mechanism of action of JAC in gastric cancer cells. Cytotoxicity and apoptosis assays showed that JAC effectively killed multiple gastric cancer cells and induced apoptosis in human gastric adenocarcinoma AGS cells via the mitochondrial pathway. Network pharmacological analysis suggested that its activity was linked to reactive oxygen species (ROS), AKT, and MAPK signaling pathways. Furthermore, JAC accumulated ROS to up-regulate p-JNK, p-p38, and IκB-α protein expressions and down-regulate the p-ERK, p-STAT3, and NF-κB protein expressions. Cell cycle assay results showed that JAC accumulated ROS to up-regulate p21 and p27 protein expressions and down-regulate p-AKT, CDK2, CDK4, CDK6, Cyclin D1, and Cyclin E protein expressions to induce G0/G1 phase arrest. Cell migration assay results showed JAC accumulated ROS to down-regulate Wnt-3a, p-GSK-3β, N-cadherin, and β-catenin protein expressions and up-regulate E-cadherin protein expression to inhibit migration. Furthermore, N-acetyl cysteine pre-treatment prevented the change of these protein expressions. In summary, JAC induced apoptosis and G0/G1 phase arrest and inhibited migration through ROS-mediated signaling pathways in AGS cells.

## Introduction

Gastric cancer (GC) is highly invasive and heterogeneous. It is the fourth most common malignancy and the leading cause of cancer death globally [1–3]. Currently, GC treatment includes targeted therapy, immunotherapy, surgical therapy, and chemotherapy. However, these methods are non-specific and have serious side effects [4,5]; therefore, efficient and safe drugs must be needed.

Recently, naturally active substances have attracted attention owing to their high efficiency, multiple targets, and low toxicity [6,7]. Flavonoids are mainly found in higher plants and show numerous biological activities [8–12]. Jaceosidin (JAC), a natural flavonoid extracted from *Folium Artemisia Argyi* [13], has anti-inflammatory, anti-oxidant, and other pharmacological activities [14–16]. JAC inhibits the proliferation of oral, breast, and ovarian cancer cells and induces their apoptosis [17–19]. However, its specific anti-cancer molecular mechanisms remain unclear.

Inducing cancer cell apoptosis is a treatment method [20]. Mitochondria-dependent apoptosis is mainly caused by oxidative stress, which reduces mitochondrial membrane potential (MMP) and increases intracellular reactive oxygen species (ROS) [21,22]. Following stimulation by excessive ROS [23], the mitogen-activated protein kinase (MAPK) signaling pathway including ERK, JNK and p38 regulates the signal transducer and activator of transcription 3 (STAT3) and nuclear factor kappa B (NF-κB) signaling pathways [24,25]. Then activating the Bcl-2 family of proteins changes the MMP and induces the release of cytochrome c (Cyto-c) into the cytoplasm [26]. Cyto-c induces caspase family proteins to cleave substrates such as poly ADP-ribose polymerase (PARP) and induce cell apoptosis [27,28].

The cell cycle is the entire process of cell division. Naturally active substances arrest cancer cells at various stages of division by regulating p53, AKT, and other signaling pathways [29,30]. The AKT signaling pathway regulates cyclin-dependent kinase (CDK), which combines with cyclin to form heterodimers and catalyze phosphorylation to regulate cell cycle [31,32].

Excessive migration of tumor cells is an important characteristic of malignancy and cause of death in patients with cancer [33]. The Wnt-3a signaling pathway inhibits GSK-3β phosphorylation to inhibit β-catenin expression in the cytoplasm, thereby regulating the expression of target genes such as E-cadherin [34]. The increased E-cadherin expression enhances intercellular adhesion and inhibits cancer cell migration [35].

This study evaluated the anti-GC effects of JAC and its specific molecular mechanisms using network pharmacology and molecular analysis.

## Materials and methods

### Drugs and reagents

Cell-level dimethyl sulfoxide (DMSO) (Solarbio, Beijing, China) was used to dissolve JAC (HerbPurify, Chengdu, China) and 5-fluorouracil (5-FU) (Med Chem Express, Princeton, NJ, USA) and the solutions were stored at −20°C. Cell Counting kit-8 (CCK-8), Annexin V-FITC/PI apoptosis kit, MMP assay kit, and DNA quantification kit were acquired from Solarbio. ROS assay kit was obtained from Beyotime (Shanghai, China).

### Cell culture

Human GC cells (AGS, KATO-3, MKN-28, MKN-45, NCI-N87, SNU-5, SNU-216, SNU-484, SNU-668, YCC-1, YCC-6, and YCC-16) and normal lung IMR-90 cells were obtained from the American Type Culture Collection (Manassas, VA, USA). Normal gastric GES-1 cells, normal kidney 293 T cells, and normal liver THLE-2 cells were obtained from Sage Biotechnology (Shanghai, China) and OWTO Biotech (Shenzhen, China). Cells were cultured in RPMI 1640 or Dulbecco's modified Eagle's medium (DMEM) culture medium containing 10% fetal bovine serum (FBS) (Gibco, Waltham, MA, USA) and 100 U/mL penicillin and 100 μg/mL streptomycin (Solarbio). Cells were cultured at 37°C in an incubator (Osaka, Japan) with 5% CO_2_.

### Cell cytotoxic assay

Cells were seeded in 96-well plates (1 × 10^4^ cells/well) for 24 h. JAC or 5-FU were used to treat cells at 20, 40, 60, 80, and 100 μM and for 6, 12, 18, 24, and 30 h. CCK-8 (10 μL) was added to each well and incubated light-free for 3 h. Optical density (OD) was measured at 450 nm using a multifunctional microplate reader (Tecan, Mannedorf, Switzerland).

### Cell apoptosis assay

AGS and GES-1 cells were seeded in 6-well plates (1 × 10^5^ cells/well) for 24 h. Afterwards, cells were treated with 39 μM JAC or 5-FU at 3, 6, 12, and 24 h. The treated cells were collected, and apoptotic cells were assessed using an Annexin V-FITC/PI apoptosis kit and flow cytometry (Sysmex, Kobe, Japan). In addition, AGS cells were pre-treated for 30 min with 10 mM N-acetyl cysteine (NAC) before JAC treatment and redetected.

### Cell MMP assay

AGS cells were seeded in 6-well plates (1 × 10^5^ cells/well) for 24 h. Afterwards, the cells were treated with 39 μM JAC at 3, 6, 12, and 24 h. The treated cells were collected, and MMP in cells were assessed using an MMP assay kit and flow cytometry.

### Cross-target screening

The 2D structure of JAC was downloaded from PubChem (https://pubchem.ncbi.nlm.nih.gov) and uploaded to the Swiss Target Prediction (http://swisstargetprediction.ch) database to predict target genes. We obtained 1766 targets for GC from Gene Cards (https://www.genecards.org) after three median screenings. Venny 2.1 was used to identify cross-targets for 100 drugs and 1766 disease targets. The cross-targets were entered into the STRING database, and the obtained drug-disease Protein–Protein Interaction (PPI) network was imported into Cytoscape.

### GO and KEGG enrichment analysis

The Database for Annotation, Visualization, and Integrated Discovery (https://david.ncifcrf.gov) was used to obtain data on the biological functions and signaling pathways in which cross-targets may be involved. After sorting the obtained data, the top 25 were selected for visualization using a bioinformatics database.

### Cell ROS accumulation assay

AGS and GES-1 cells were seeded in 3.5 cm plates (1 × 10^5^ cells/well) for 24 h. Afterwards, cells were treated with 39 μM JAC at 3, 6, 12, and 24 h. The treated cells were collected, and ROS levels were assessed using a ROS assay kit and flow cytometry.

### Cell cycle assay

AGS cells were seeded in 3.5 cm plates (1 × 10^5^ cells/well) for 24 h. Afterwards, cells were treated with 39 μM JAC at 3, 6, 12, and 24 h. The treated cells were collected, and the percentages of cells in different phases were assessed using a DNA quantification kit and flow cytometry. In addition, AGS cells were pre-treated for 30 min with 10 mM NAC before JAC treatment and redetected.

### Western blotting

AGS cells were seeded in 6 cm plates (1 × 10^5^ cells/well) for 24 h. Afterwards, cells were treated with 39 μM JAC at 3, 6, 12, and 24 h. The treated cells were centrifuged (12000rpm, 30 min, 4°C), mixed with lysis buffer for 30 min on ice, and resuspended every 5 min. OD was measured at 595 nm with a spectrophotometer, mixed with 5× buffer to a final concentration of 1.5 μg/μL, and boiled for 5 min. An equal amount of protein (20 μL) was added to the spot sample hole of SDS-PAGE gels, and western blotting was performed [23]. The primary and secondary antibodies were purchased from Santa Cruz Biotechnology (Table S1; Dallas, TX, USA) and ZSGBBio (Beijing, China), respectively.

### Cell migration assay

AGS cells were seeded in 6-well plates (1 × 10^5^ cells/well) for 24 h. Afterwards, cells were treated with 39 μM JAC at 3, 6, 12, and 24 h. The degree of cell migration was assessed using wound healing assay. Additionally, the number of migrated cells was assessed using transwell assay. AGS cells were seeded in the upper chamber (1 × 10^5^ cells/well) containing serum-free medium, and DMEM containing 20% FBS was added to the lower chamber for 24 h. Cells were treated with 39 μM JAC at 3, 6, 12, and 24 h and mixed with 0.1% crystal violet solution. A fluorescence microscope was used to observe cell migration.

### Statistical analyses

All experiments were repeated in triplicate and all data were used mean ± standard deviation (SD). The half-maximal inhibitory concentration (IC_50_) was determined using GraphPad Prism 5.0. Parameter analysis was performed using a one-way analysis of variance (ANOVA) followed by Tukey's post hoc test for comparisons across the groups using SPSS 21.0, and *p *< 0.05 was considered significant (* *p *< 0.05, ** *p *< 0.01, and *** *p *< 0.001).

## Results

### JAC killed GC cells

**Figure 1.**
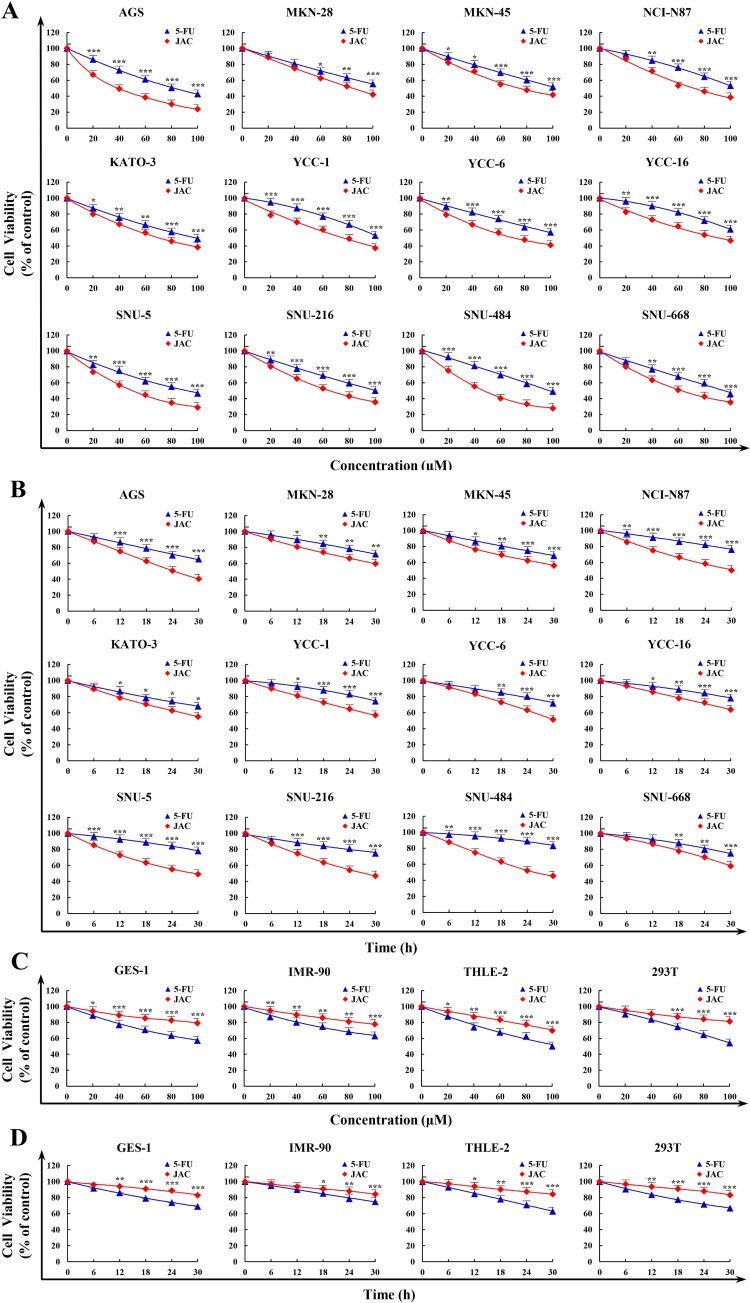
Killing effects of JAC in cells. All cell viabilities were determined by CCK-8 assay. **(A)** Twelve human gastric cancer cells were treated with JAC and 5-FU (20, 40, 60, 80, and 100 μM) for 24 h. **(B)** Twelve human gastric cancer cells were treated with JAC and 5-FU (6, 12,18, 24, and 30 h). **(C)** Four human normal cells were treated with JAC and 5-FU (20, 40, 60, 80, and 100 μM) for 24 h. **(D)** Four human normal cells were treated with the IC_50_ of JAC and 5-FU (6, 12, 18, 24, and 30 h). * *p* < 0.05, ** *p* < 0.01, *** *p* < 0.001 vs. 5-FU group.

JAC showed higher killing effects on various GC cells and lower cytotoxicity in normal cells than 5-FU (Figure 1(a–d)). The treatment time and concentration of JAC determined its cytotoxicity, and the IC_50_ values of JAC in GC cells are summarized in Table 1. IC_50_ was used to evaluate the anticancer activity and compare side effects of drugs. Because the lowest IC_50_ was observed in AGS cells, they were employed as models.

**Table 1.** IC_50_ values of JAC and 5-FU in gastric cancer cells.

Cell line	5-FU (μM)	JAC (μM)
AGS	82.36 ± 2.23	38.65 ± 2.13
MKN-28	109.29 ± 2.43	83.61 ± 2.41
MKN-45	103.48 ± 2.5	72.52 ± 2.37
NCI-N87	102.17 ± 2.55	69.18 ± 2.82
SNU-5	94.89 ± 2.18	52.65 ± 2.09
SNU-216	102.49 ± 2.43	65.16 ± 2.03
SNU-484	98.92 ± 2.46	47.84 ± 2.24
SNU-668	92.65 ± 2.79	64.45 ± 1.98
KATO-3	97.52 ± 2.33	68.47 ± 2.25
YCC-1	107.36 ± 2.44	78.35 ± 2.49
YCC-6	124.27 ± 2.4	74.52 ± 2.47
YCC-16	117.49 ± 2.57	90.46 ± 2.39

### JAC induced mitochondria-dependent apoptosis of AGS cells

**Figure 2.**
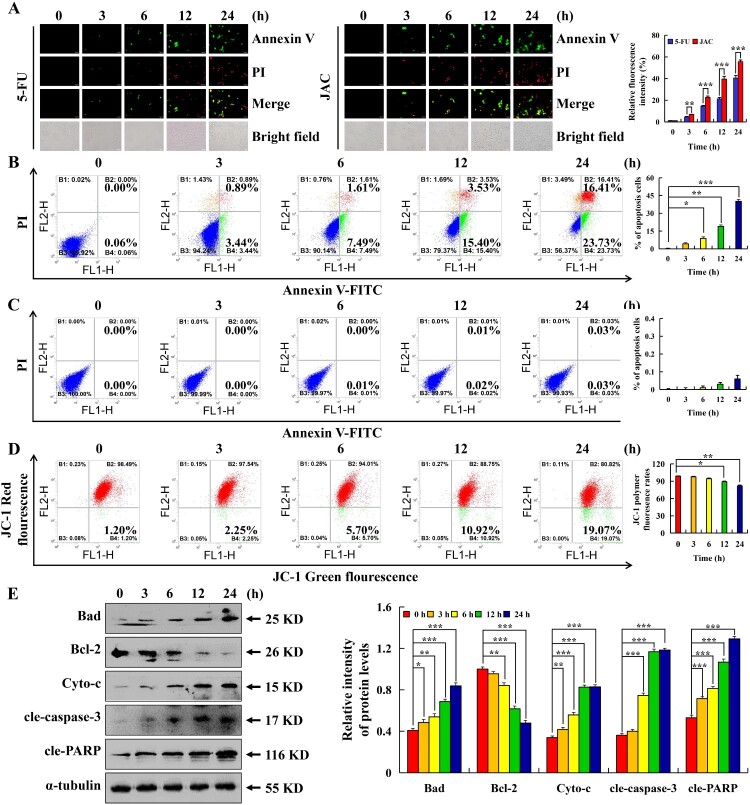
Apoptotic effects of JAC in AGS and GES-1 cells. AGS and GES-1 cells were treated with 39 μM JAC or 5-FU for 3, 6, 12, and 24 h. **(A)** AGS cells were incubated with Annexin V-FITC/PI and fluorescence microscope images were determined (original magnification, 200 ×). **(B)** AGS cells were incubated with Annexin V-FITC/PI and analyzed by flow cytometry. **(C)** GES-1 cells were incubated with Annexin V-FITC/PI and analyzed by flow cytometry. **(D)** AGS cells were incubated with JC-1 reagent and analyzed by flow cytometry. **(E)** The protein expression was measured by western blotting following treatment of AGS cells with JAC. α-tubulin was used as an internal control. Data are representative of three independent experiments (n = 3), * *p* < 0.05, ** *p* < 0.01, and *** *p* < 0.001 vs. 0 h.

Following JAC treatment, the degree of AGS cell fluorescence was considerably higher than that in the 5-FU control group (Figure 2(a)). AGS cell apoptosis was enhanced by 40.08% and MMP was reduced by 17.87%; however, GES-1 cell apoptosis did not increase (Figure 2(b–d)). Western blotting results showed that Cyto-c, Bad, cleaved-caspase-3 (cle-caspase-3), and cleaved-PARP (cle-PARP) expressions were significantly increased, and that of Bcl-2 was significantly decreased in AGS cells (*p* < 0.001, Figure 2(e)).

### EGFR and AKT1 may the key roles in JAC anti-GC effect

**Figure 3.**
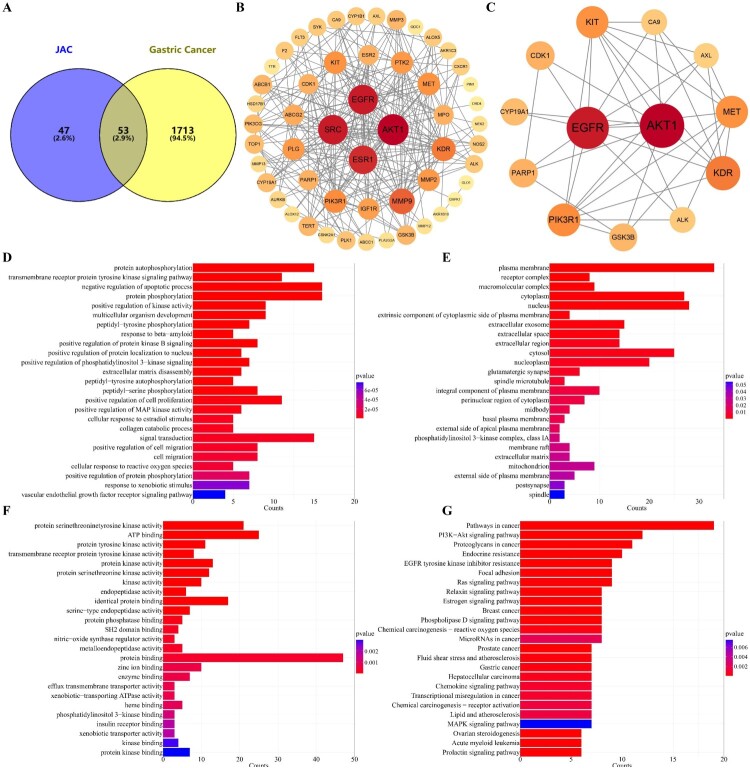
Network pharmacological analysis of JAC and gastric cancer. **(A)** Cross targets of JAC and gastric cancer drawn using Venny diagram. JAC targets are shown in blue, gastric cancer targets are shown in yellow, and cross targets are shown as a shadow. In the PPI network, nodes represent proteins, size represents the amount of gene enrichment, color represents confidence. **(B)** PPI network drawn using the STRING database and Cystoscope. **(C)** PPI Core network drawn using Cystoscope. In GO enrichment and KEGG pathway analyses, bar chart length represents the amount of gene enrichment, color represents confidence. **(D)** BP analysis of cross targets of JAC and gastric cancer. **(E)** CC of cross targets of JAC and gastric cancer. **(F)** MF of cross targets of JAC and gastric cancer. **(G)** KEGG pathway analysis of cross targets of JAC and gastric cancer.

Cross-target screening indicated that EGFR and AKT1 proteins of 53 cross-targets may play key roles (Figure 3(a–c)). AKT1’s threshold and number of interacting proteins were higher than those of EGFR, indicating that AKT1 is probably the most important target. GO analysis showed that positive regulation of MAP kinase activity, protein phosphorylation, protein kinase activity, and protein serine/threonine/tyrosine kinase activity were important (Figure 3(d–f)). KEGG analysis showed that PI3K-AKT, Ras, and MAPK, three important signaling pathways in cancer, may be crucial for the anti-GC effect of JAC. Moreover, ROS were similarly important in the KEGG analysis (Figure 3(g)).

### JAC induced AGS cell apoptosis through the MAPK/STAT3/NF-κB signaling pathway

**Figure 4.**
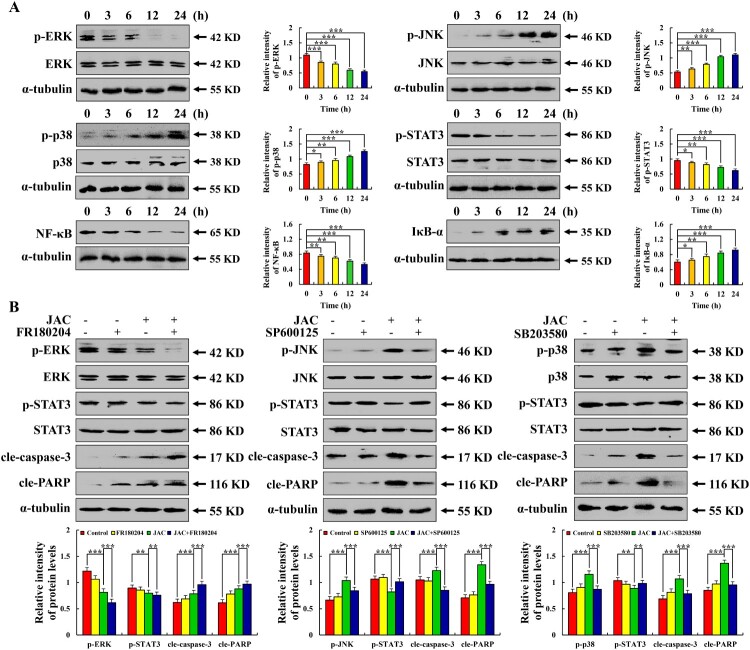
Effects of JAC on the MAPK/STAT3/NF-κB signaling pathway in AGS cells. AGS cells were treated with 39 μM JAC for 3, 6, 12, and 24 h. **(A)** Expression of MAPK, STAT3, and NF-κB signaling pathway-related proteins detected by western blotting. AGS cells treated with 39 µM JAC and 10 µM MAPK signaling pathway inhibitors for 24 h. **(B)** Expression of MAPK signaling pathway (ERK, JNK, and p38) proteins detected by western blotting analysis. α-tubulin was used as an internal control. Data are representative of three independent experiments (n = 3), * *p* < 0.05, ** *p* < 0.01, and *** *p* < 0.001 vs. 0 h, control or JAC + MAPK inhibitor groups.

Following JAC treatment, p-JNK, p-p38, and IκB-α expressions were significantly increased, while those of p-ERK, p-STAT3, and NF-κB were significantly decreased (*p* < 0.001, Figure 4(a)). After pre-treatment with MAPK inhibitors, p-JNK and p-p38 expressions were significantly decreased, while p-STAT3 expression was significantly increased compared to that from JAC treatment alone. However, p-ERK and p-STAT3 expressions were significantly decreased (*p* < 0.001, Figure 4(b)).

### JAC regulated the MAPK/STAT3/NF-κB signaling pathway by accumulating ROS

**Figure 5.**
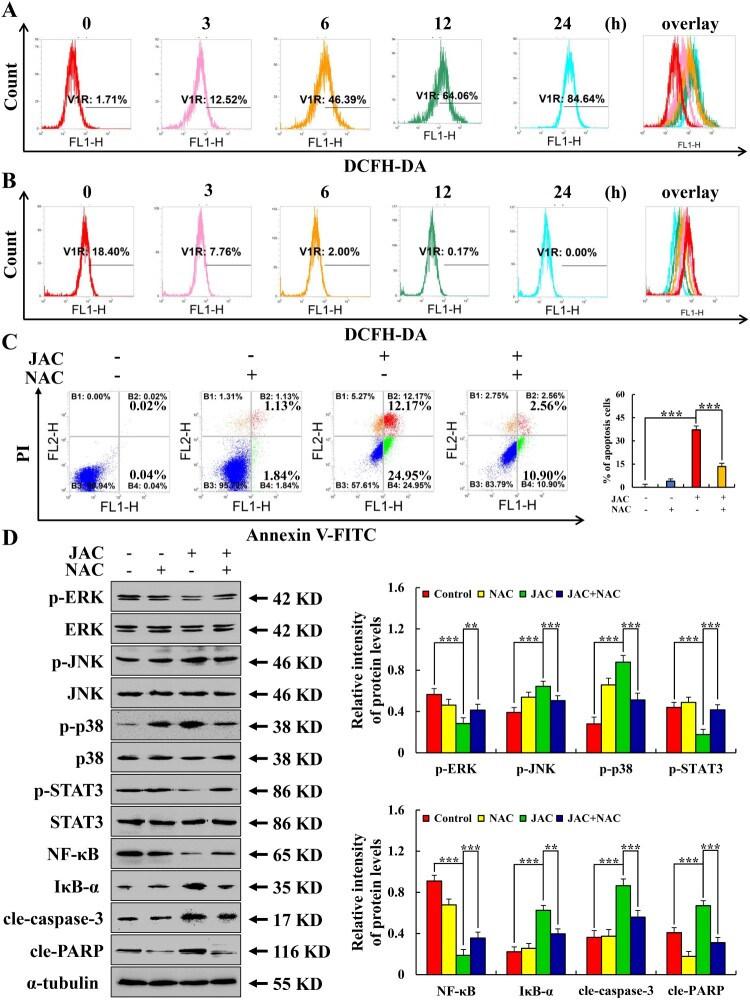
Effects of JAC on ROS accumulation in AGS and GES-1 cells. AGS and GES-1 cells were treated with 39 µM JAC for 3, 6, 12, and 24 h. **(A)** ROS accumulation in AGS cells was analyzed by DCFH-DA staining and flow cytometry. **(B)** ROS accumulation in GES-1 cells was analyzed by DCFH-DA staining and flow cytometry. AGS cells were treated with 39 µM JAC and 10 mM NAC for 24 h. **(C)** AGS cells were incubated with Annexin V-FITC/PI and flow cytometry analysis was performed to determine apoptosis. **(D)** Expression levels of MAPK, STAT3, NF-κB signaling pathway-related proteins, cle-caspase-3, and cle-PARP proteins detected by western blotting. α-tubulin was used as an internal control. Data are representative of three independent experiments (n = 3), * *p* < 0.05, ** *p* < 0.01, and *** *p* < 0.001 vs. control or JAC + NAC groups.

Following JAC treatment, ROS accumulation in AGS and GES-1 cells increased and decreased, respectively (Figure 5(a, b)). After pre-treatment with NAC before JAC, the apoptotic rate of AGS cells decreased from 37.12% to 13.46%. Moreover, related protein expressions were prevented by NAC pre-treatment (Figure 5(c,d)).

### JAC arrested AGS cells in the G0/G1 phase

**Figure 6.**
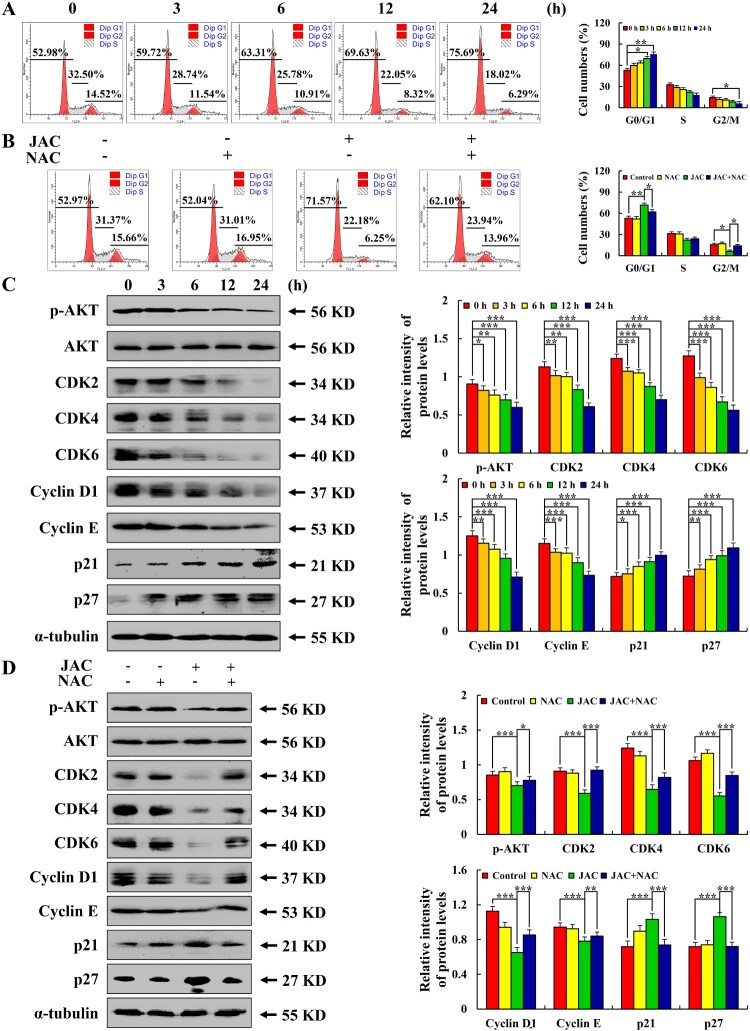
Effects of JAC on cell cycle. AGS cells were treated with 39 µM JAC for 3, 6, 12, and 24 h. **(A)** AGS cells were incubated with RNase and PI staining and analyzed by flow cytometry. **(B)** AGS cells treated with 39 µM JAC and/or 10 mM NAC for 24 h. AGS cells were incubated with RNase and PI staining and analyzed by flow cytometry. **(C)** AGS cells were treated with 39 µM JAC for 3, 6, 12, and 24 h. Expression of p-AKT, CDK2, CDK4, CDK6, Cyclin D1, Cyclin E, p21, and p27 detected by western blotting. α-tubulin was used as the internal control. **(D)** AGS cells treated with 39 µM JAC and/or 10 mM NAC for 24 h. Expression of p-AKT, CDK2, CDK4, CDK6, Cyclin D1, Cyclin E, and p27 detected by western blotting. α-tubulin was used as an internal control. Data are representative of three independent experiments (n = 3), * *p* < 0.05, ** *p* < 0.01, and *** *p* < 0.001 vs. 0 h, control or JAC + NAC groups.

Following JAC treatment, the proportion of G0/G1 phase cells increased from 52.98% to 75.69%, whereas this change was prevented by NAC pre-treatment (Figure 6(a,b)). Cell cycle-related protein analysis showed that p21 and p27 expressions were significantly increased, whereas those of p-AKT, CDK2, CDK4, CDK6, Cyclin D1, and Cyclin E were significantly decreased. Moreover, these changes were prevented by NAC pre-treatment (*p* < 0.001, Figure 6(c,d)).

### JAC inhibited AGS cell migration

**Figure 7.**
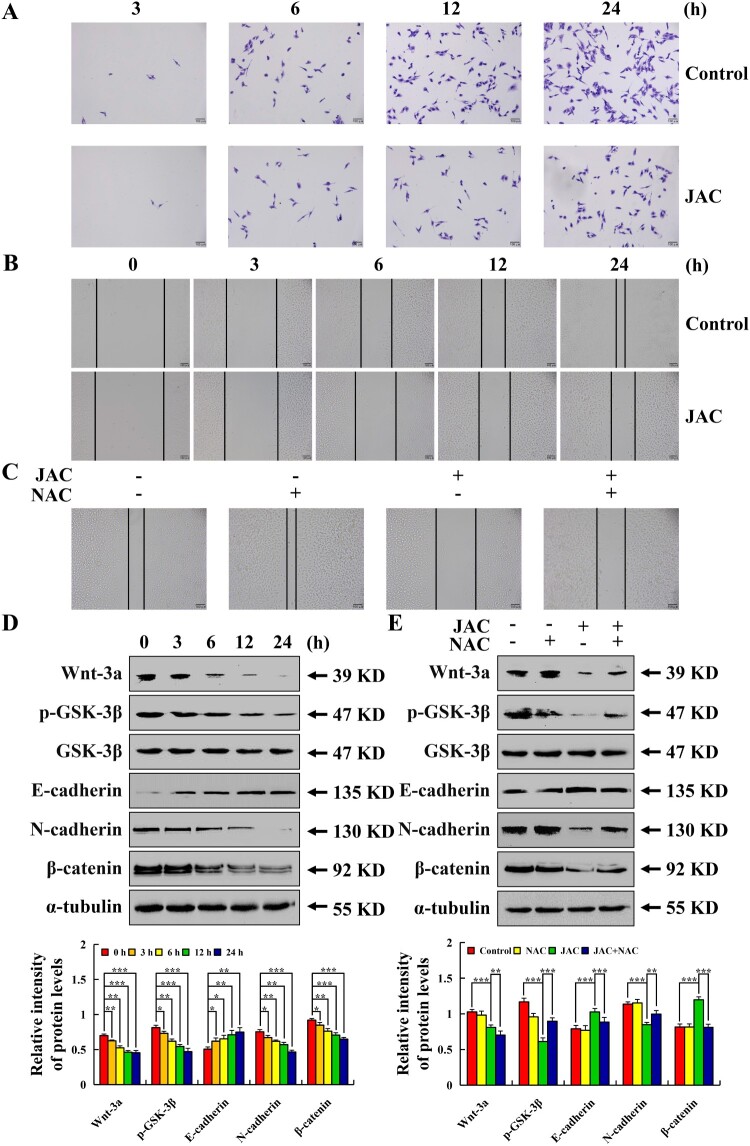
Effects of JAC on migration in AGS cells. AGS cells were treated with 39 µM JAC for 3, 6, 12, and 24 h, followed by fluorescence microscope (original magnification, 200 ×). **(A)** Transwell assay analysis of cell migration. **(B)** Wound-healing assay analysis of cell migration. **(C)** AGS cells were treated with 39 µM JAC and/or 10 mM NAC for 24 h. Wound healing assay analysis of cell migration rate. **(D)** AGS cells were treated with 39 µM JAC for 3, 6, 12, and 24 h. Expressions of Wnt-3a, p-GSK-3β, E-cadherin, N-cadherin, and β-catenin were determined by western blotting. **(E)** AGS cells were treated with 39 µM JAC and/or 10 mM NAC for 24 h. Expression of Wnt-3a, p-GSK-3β, E-cadherin, N-cadherin, and β-catenin were determined by western blotting. α-tubulin was used as an internal control. Data are representative of three independent experiments (n = 3), * *p* < 0.05, ** *p* < 0.01, and *** *p* < 0.001 vs. 0 h, control, or JAC + NAC groups.

Following JAC treatment, the migration ability of AGS cells was considerably reduced, whereas this change prevented by NAC pre-treatment (Figure 7(a–c)). Cell migration-related protein analysis showed Wnt-3a, p-GSK-3β, N-cadherin, and β-catenin expressions were significantly decreased, while E-cadherin expression was significantly increased. Moreover, these changes were prevented by NAC pre-treatment (*p* < 0.001, Figure 7(d,e)).

## Discussion

Despite the proven cytocidal activity of JAC against various cancer cells, the underlying molecular mechanisms remain unclear. In a preliminary experiment, 5-FU showed greater cytocidal activity against GC cells than other chemotherapy drugs. Therefore, 5-FU was chosen as the positive control in subsequent experiments. JAC showed high cytotoxicity towards the 12 types of GC cells, with the highest cytotoxicity against AGS cells (IC_50 _= 39 μΜ). Subsequently, we studied the anti-GC effects of JAC in AGS cells.

We found that following JAC treatment, apoptotic cells increased by 40.08%, but PI + Annexin V cells also increased. However, subsequent results explain this phenomenon. We found that JAC increased Cyto-c, Bad, cle-caspase-3, and cle-PARP expressions and decreased Bcl-2 expression. The decrease in MMP and Bad/Bcl-2 ratio indicated that JAC induced apoptosis via the mitochondrial pathway in AGS cells.

We predicted the mechanism underlying JAC-induced apoptosis using network pharmacology. The results showed that positive regulation of MAP kinase activity, protein phosphorylation, protein kinase activity, and protein serine/threonine/tyrosine kinase activity were the main biological processes and molecular functions. MAPK is a serine/threonine kinase that acts primarily through protein phosphorylation and positive regulation of kinase activity. These biological processes and molecular functions were in the network pharmacology results. Additionally, cross-targeting mainly involved the PI3K-AKT, Ras, and MAPK signaling pathways. The MAPK signaling pathway can down-regulate Bcl-2 expression to induce apoptosis [36]. We speculated that the MAPK signaling pathway might be a potential mechanism of JAC anti-GC activity. We verified this hypothesis using western blotting and found that p-JNK, p-p38, and IκB-α expressions were increased, while p-ERK and p-STAT3 expressions were decreased after JAC treatment. Moreover, these effects were prevented by adding MAPK signaling pathway inhibitors. These results demonstrated that JAC induced mitochondria-dependent apoptosis of AGS cells through the MAPK/STAT3/NF-κB signaling pathway.

Some antitumor drugs induce apoptosis by up-regulating ROS accumulation in cancer cells [37,38]. Herein, JAC increased ROS accumulation in AGS cells, confirming the accuracy of KEGG analysis. Moreover, flavonoids can be anti-oxidant or pro-oxidant in cancerous or normal cells. Herein, JAC decreased ROS accumulation in GES-1 cells. These results suggested that JAC accumulating intracellular ROS in AGS cells but showed anti-oxidant activity in GES-1 cells.

NAC is a sulfhydryl anti-oxidant that protects against oxidative stress-induced damage. It effectively reduced JAC-induced apoptosis and inhibited changes in MAPK signaling pathway protein expressions. This suggested that JAC induced AGS cell apoptosis by accumulating ROS to regulate the MAPK/STAT3/NF-κB signaling pathway. To the best of our knowledge, this is the first study to elucidate the mechanism of JAC in inducing GC cell apoptosis. To date, the molecular mechanism of JAC in inducing AGS cell apoptosis has been clarified. However, the downstream signaling pathways regulated by ROS are relatively complex, and induction of apoptosis is not the only anti-cancer mechanism of naturally active substances, with autophagy and other cell death modes having been studied [39–41]. Future studies should explore other pathways of ROS on apoptosis induction in AGS cells or comprehensively explore other modes of death to further elucidate the anti-cancer mechanism of JAC.

Cell cycle arrest is one of the effective methods to treat cancer [42]. Network pharmacological analysis and subsequent results showed that JAC arrested AGS cells in the G0/G1 phase by accumulating ROS to inhibit the AKT signaling pathway. Combined with similar studies [43,44], this finding indicates that cell cycle arrest induced by the same drug can follow different pathways in different cancer cells; however, the reasons remain to be elucidated.

Subsequent results showed that JAC inhibited AGS cell migration effectively, and E-cadherin expression was increased, while Wnt-3a, p-GSK-3β, N-cadherin, and β-catenin expressions were decreased. These protein expressions were prevented by NAC pre-treatment. These results suggested that JAC inhibited the migration of AGS cells by accumulating ROS to inhibit the Wnt-3a/GSK-3β/β-catenin signaling pathway.

## Conclusion

**Figure 8.**
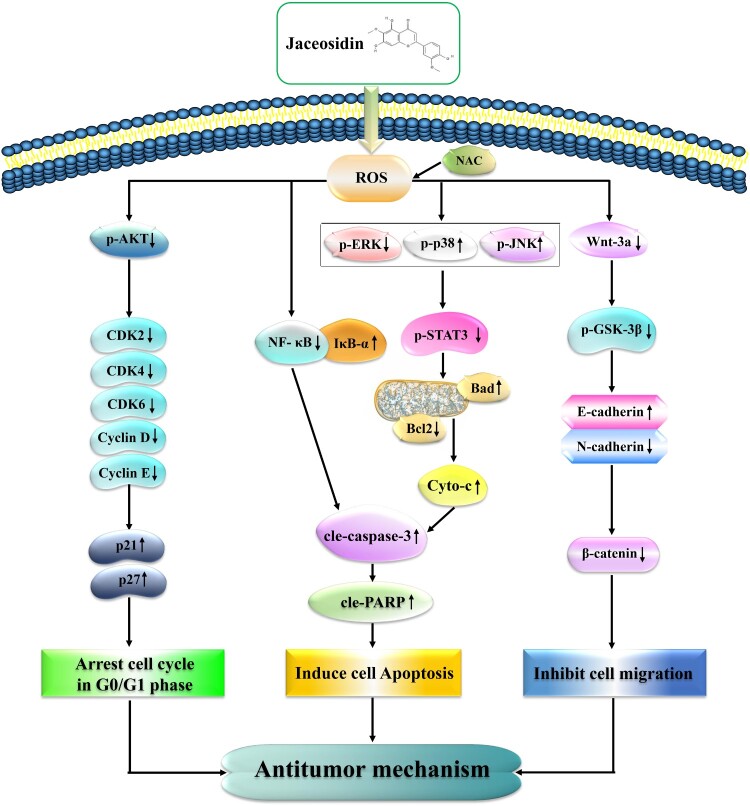
Schematic diagram of the anticancer role of JAC in AGS cells. JAC plays an anti-GC activity by accumulating ROS further induced mitochondria-dependent apoptosis through the MAPK/STAT3/NF-κB signaling pathway, arresting cells in the G0/G1 phase by inhibiting the AKT signaling pathway and inhibiting cell migration by inhibiting the Wnt-3a/GSK-3β/β-catenin signaling pathway.

In summary, JAC exhibits anti-GC activity by accumulating ROS further induced mitochondria-dependent apoptosis through the MAPK/STAT3/NF-κB signaling pathway, arresting cells in the G0/G1 phase by inhibiting the AKT signaling pathway and inhibiting cell migration by inhibiting the Wnt-3a/GSK-3β/β-catenin signaling pathway (Figure 8). These findings provide a fresh perspective for future studies on the pharmacological effects of JAC.

## Supplementary Material

Table S1.docx

Original Images for Blots.zip

## Data Availability

The data used to support the findings of this study are available from the corresponding authors upon request.
