# Supplementary material for: Jaceosidin induces apoptosis and inhibits migration in AGS gastric cancer cells by regulating ROS-mediated signaling pathways
Source: Redox Rep. 2024 Feb 6;29(1):2313366. doi: 10.1080/13510002.2024.2313366 (PMC10854459; doi:10.1080/13510002.2024.2313366)
Supplement: Table S1.docx [file YRER_A_2313366_SM3876.docx]

Table S1. Primary antibodies used for western blotting.

| Primary antibodies | Catalog number | Dilution |
| --- | --- | --- |
| Bad | Cat#sc-493 | 1:1500 |
| Bcl-2 | Cat#sc-7382 | 1:1500 |
| Cyto-c | Cat#sc-13156 | 1:2000 |
| cle-caspase-3 | Cat#sc-373730 | 1:1500 |
| cle-PARP | Cat#sc-8007 | 1:1500 |
| α-tubulin | Cat#sc-47778 | 1:2500 |
| p-ERK | Cat#sc-7383 | 1:1500 |
| ERK | Cat#sc-154 | 1:1000 |
| p-JNK | Cat#sc-6254 | 1:1500 |
| JNK | Cat#sc-7345 | 1:1500 |
| p-p38 | Cat#sc-7973 | 1:1500 |
| p38 | Cat#sc-7149 | 1:1500 |
| p-STAT3 | Cat#sc-8059 | 1:1500 |
| STAT3 | Cat#sc-8019 | 1:1500 |
| NF-κB | Cat#sc-8008; | 1:1500 |
| I-κBα | Cat#sc-1643 | 1:1500 |
| p-AKT | Cat#sc-7985-R | 1:1000 |
| AKT | Cat#sc-8312 | 1:1000 |
| CDK2 | Cat#sc-163 | 1:1500 |
| CDK4 | Cat#sc-260 | 1:1500 |
| CDK6 | Cat#sc-177 | 1:1500 |
| Cyclin D1 | Cat#sc-753 | 1:1500 |
| Cyclin E | Cat#sc-182 | 1:1500 |
| p21 | Cat#sc-397 | 1:1500 |
| p27 | Cat#sc-528 | 1:1500 |
| Wnt-3a | Cat#sc-74537 | 1:1500 |
| p-GSK-3β | Cat#sc-373800 | 1:1000 |
| GSK-3β | Cat#sc-377213 | 1:1500 |
| E-cadherin | Cat#sc-8426 | 1:1000 |
| N-cadherin | Cat#sc-59987 | 1:1000 |
| β-catenin | Cat#sc-7963 | 1:1000 |
