# Supplementary figures and images for: Jaceosidin induces apoptosis and inhibits migration in AGS gastric cancer cells by regulating ROS-mediated signaling pathways (part 2 of 2)
Source: Redox Rep. 2024 Feb 6;29(1):2313366. doi: 10.1080/13510002.2024.2313366 (PMC10854459; doi:10.1080/13510002.2024.2313366)

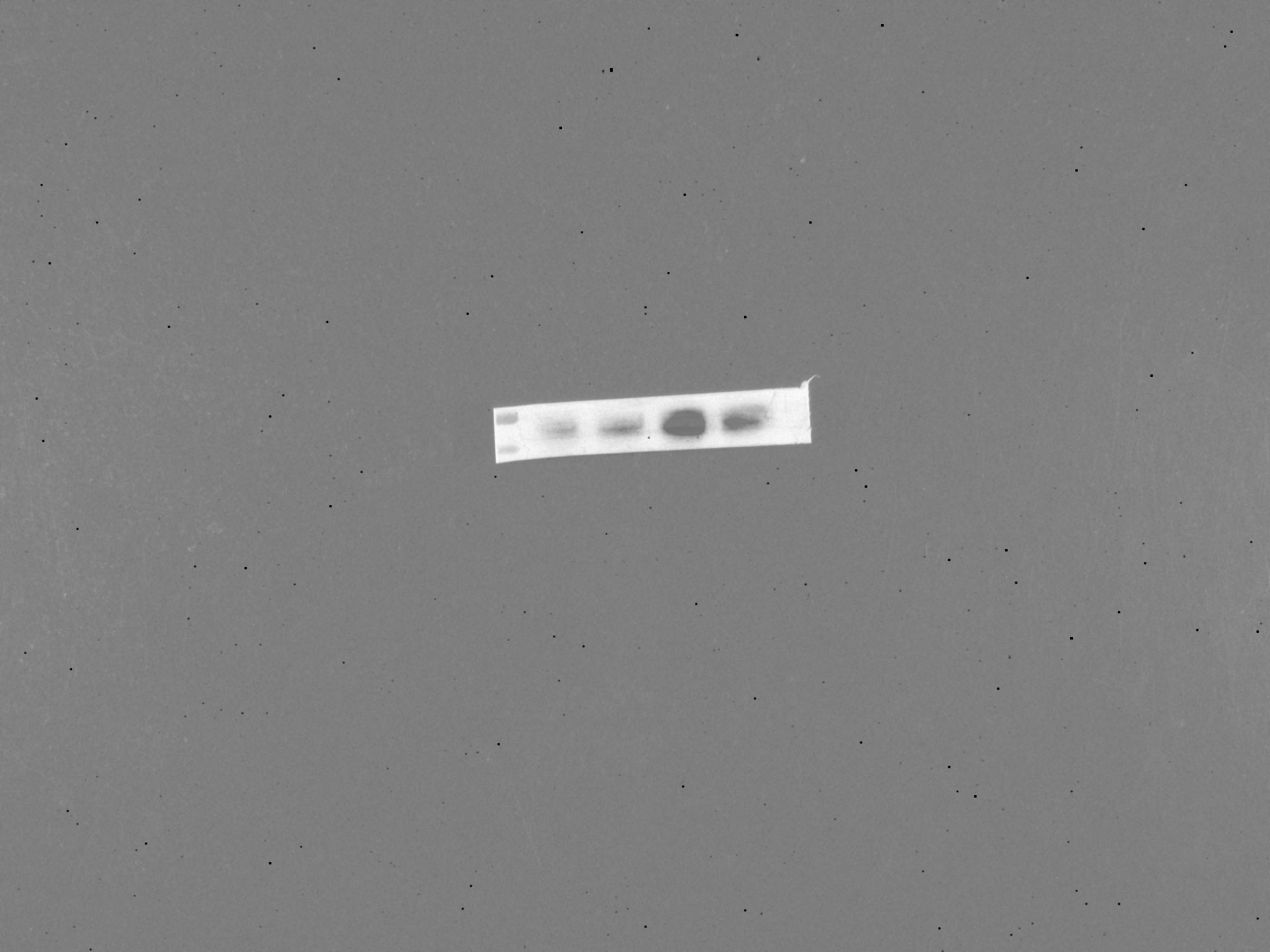

Supplement: Original Images for Blots.zip [file YRER_A_2313366_SM3875.zip › Original Images for Blots/Figure 4/Figure 4B/JNK signaling pathway/p-JNK/Marker+p-JNK.jpg]

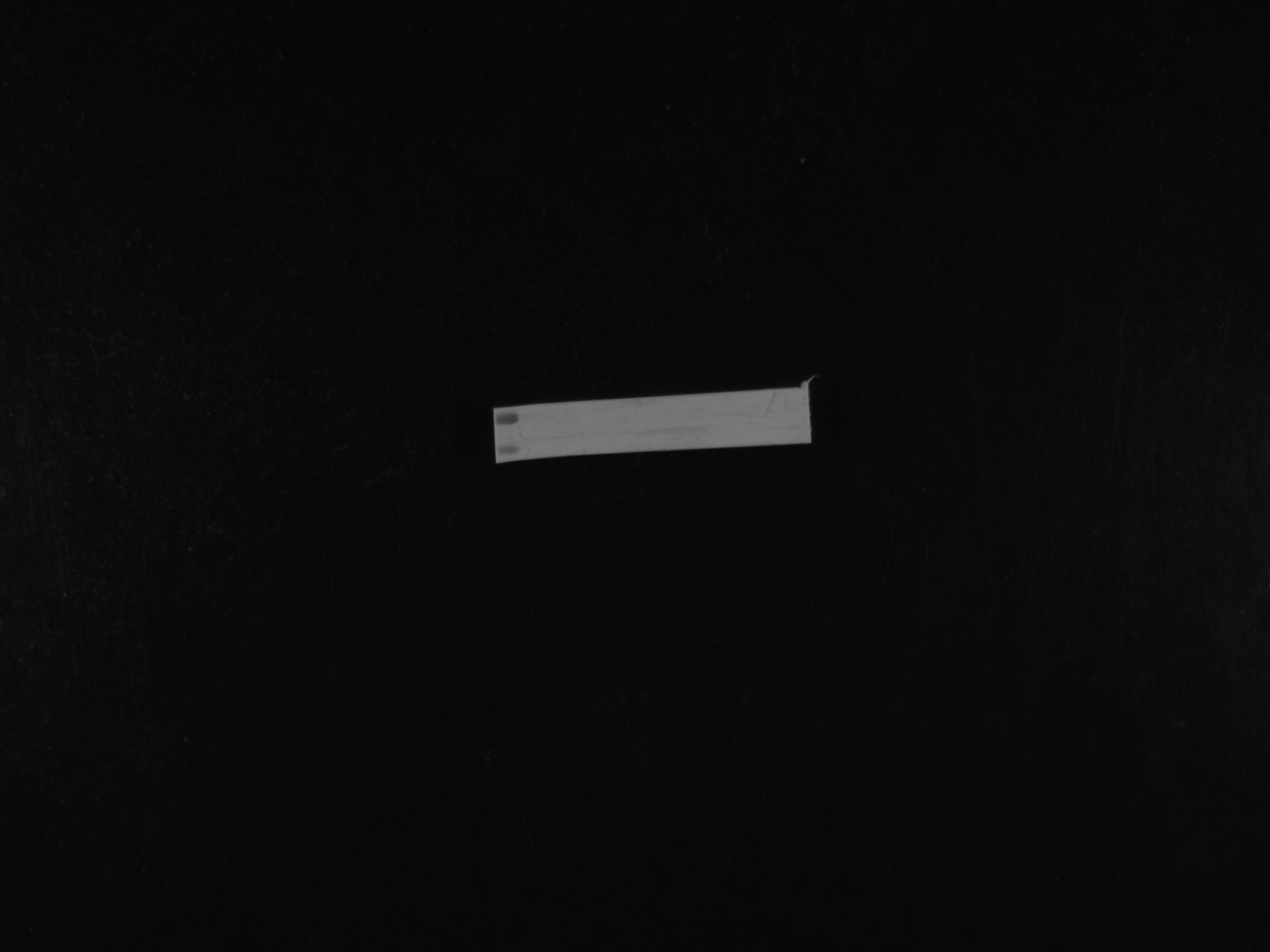

Supplement: Original Images for Blots.zip [file YRER_A_2313366_SM3875.zip › Original Images for Blots/Figure 4/Figure 4B/JNK signaling pathway/p-JNK/Marker.jpg]

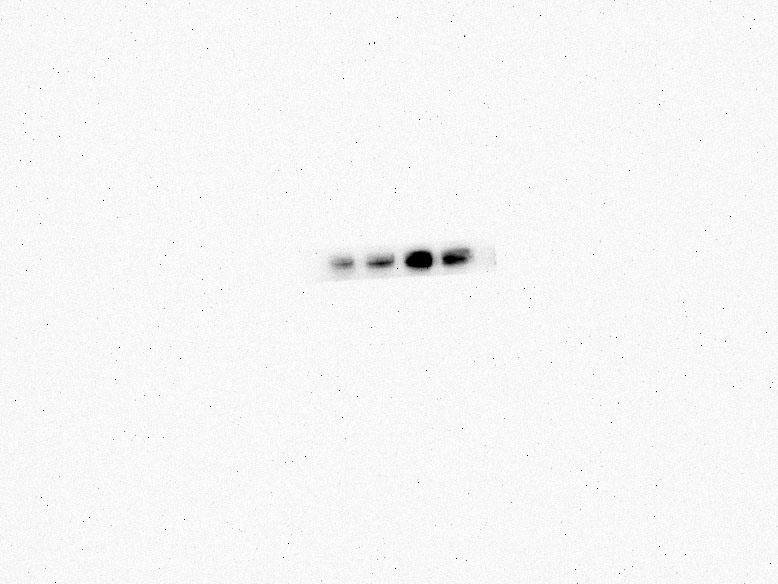

Supplement: Original Images for Blots.zip [file YRER_A_2313366_SM3875.zip › Original Images for Blots/Figure 4/Figure 4B/JNK signaling pathway/p-JNK/p-JNK.jpg]

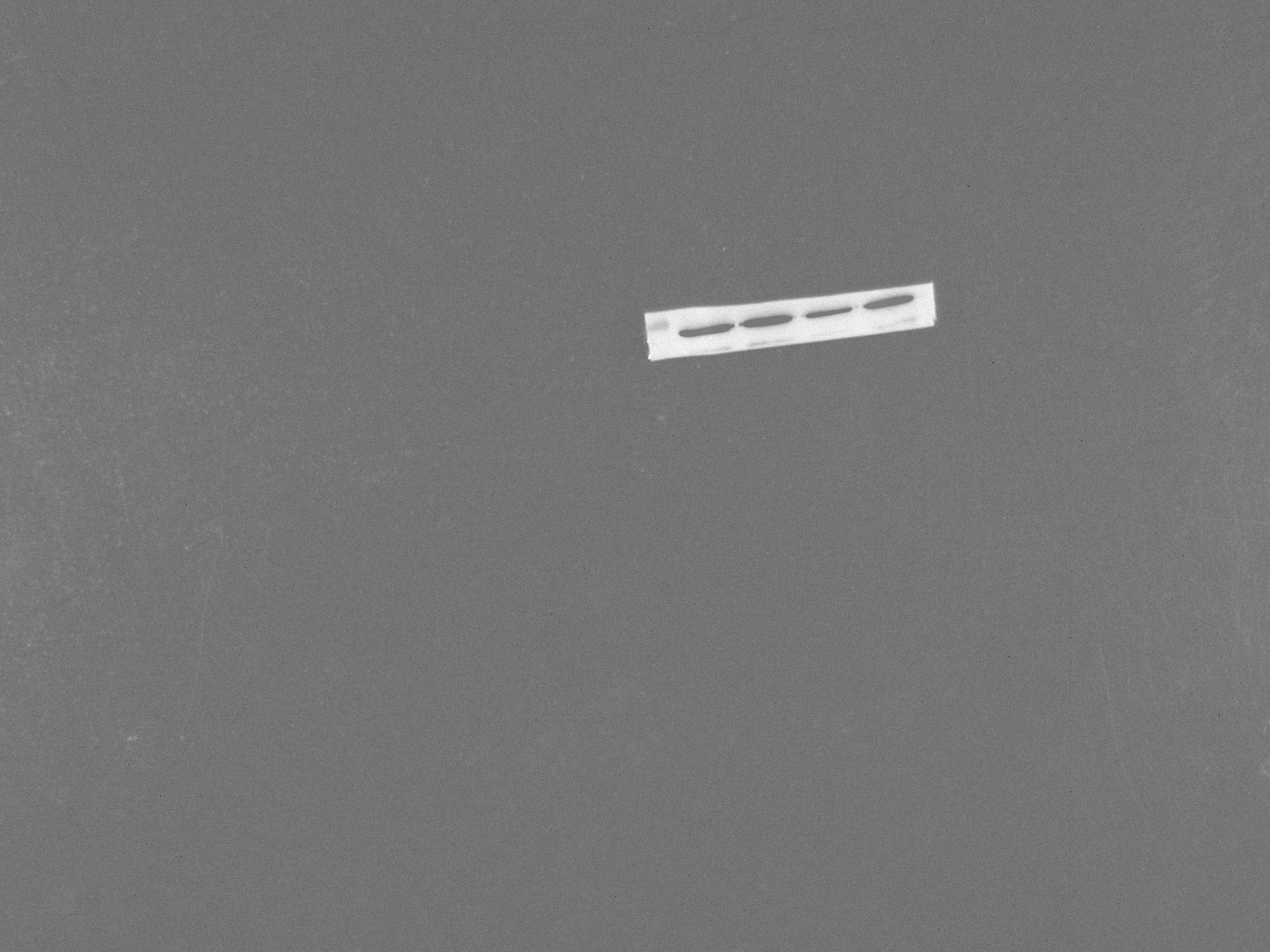

Supplement: Original Images for Blots.zip [file YRER_A_2313366_SM3875.zip › Original Images for Blots/Figure 4/Figure 4B/JNK signaling pathway/p-STAT3/Marker+p-STAT3.jpg]

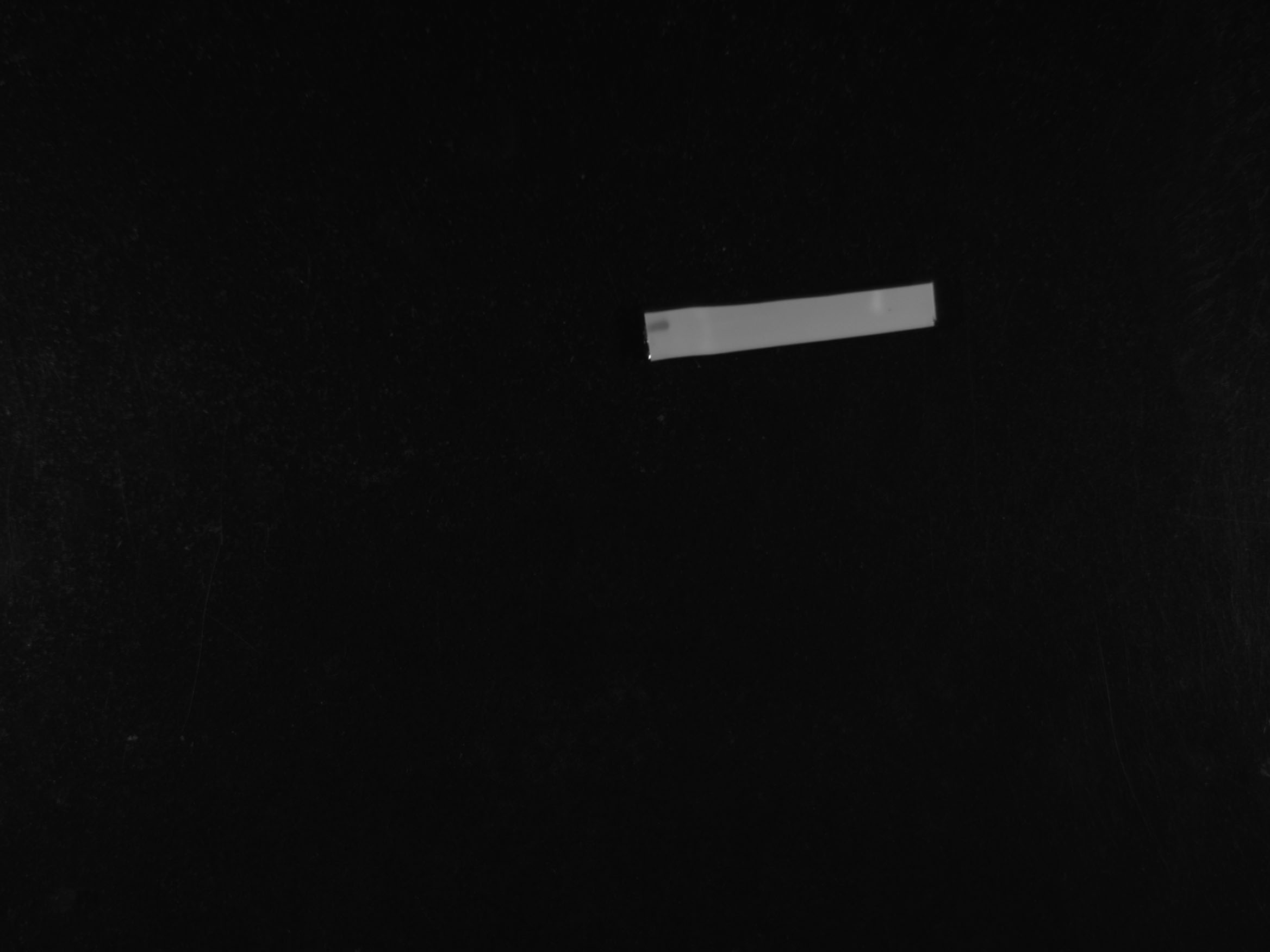

Supplement: Original Images for Blots.zip [file YRER_A_2313366_SM3875.zip › Original Images for Blots/Figure 4/Figure 4B/JNK signaling pathway/p-STAT3/Marker.jpg]

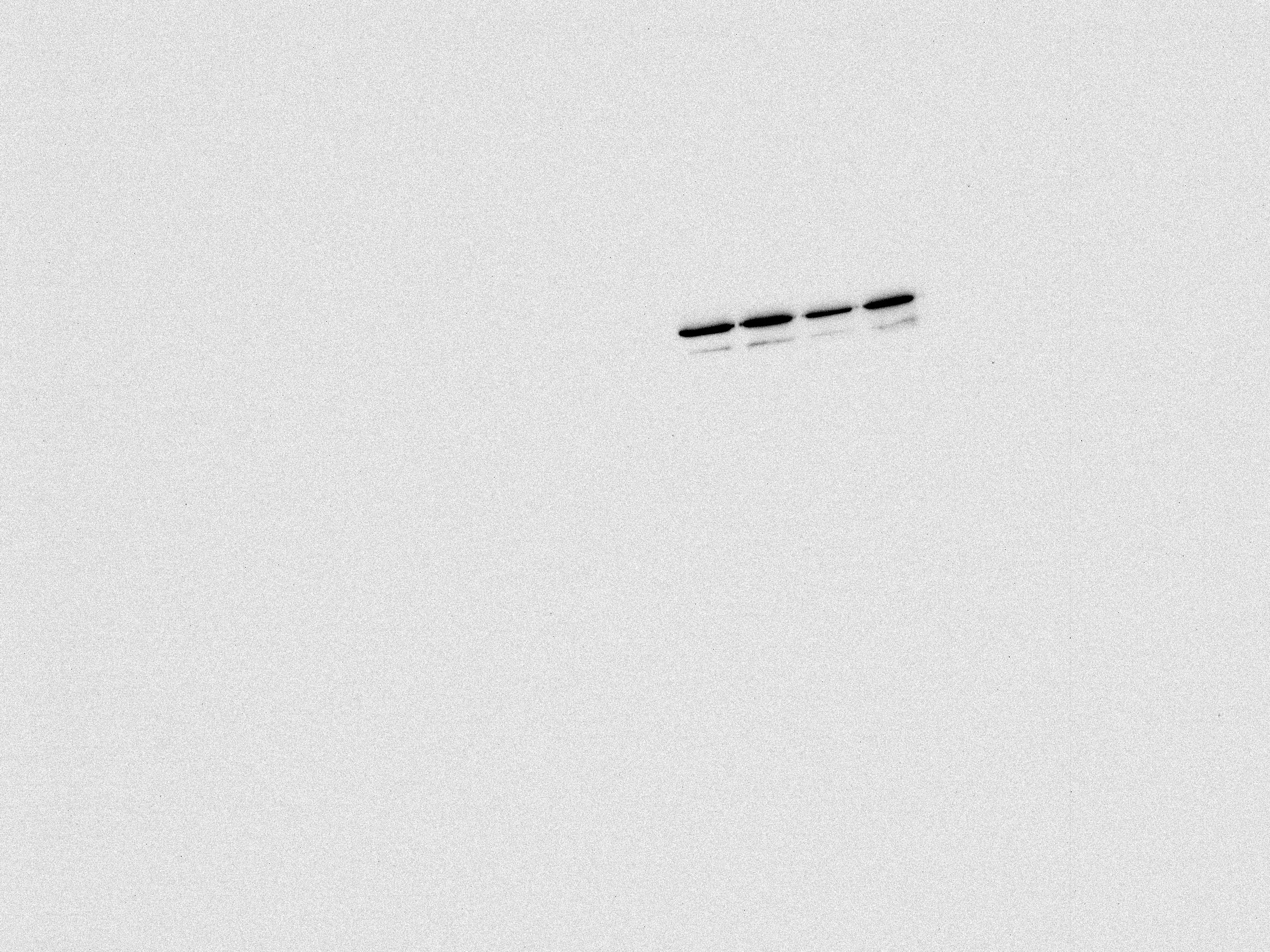

Supplement: Original Images for Blots.zip [file YRER_A_2313366_SM3875.zip › Original Images for Blots/Figure 4/Figure 4B/JNK signaling pathway/p-STAT3/p-STAT3.jpg]

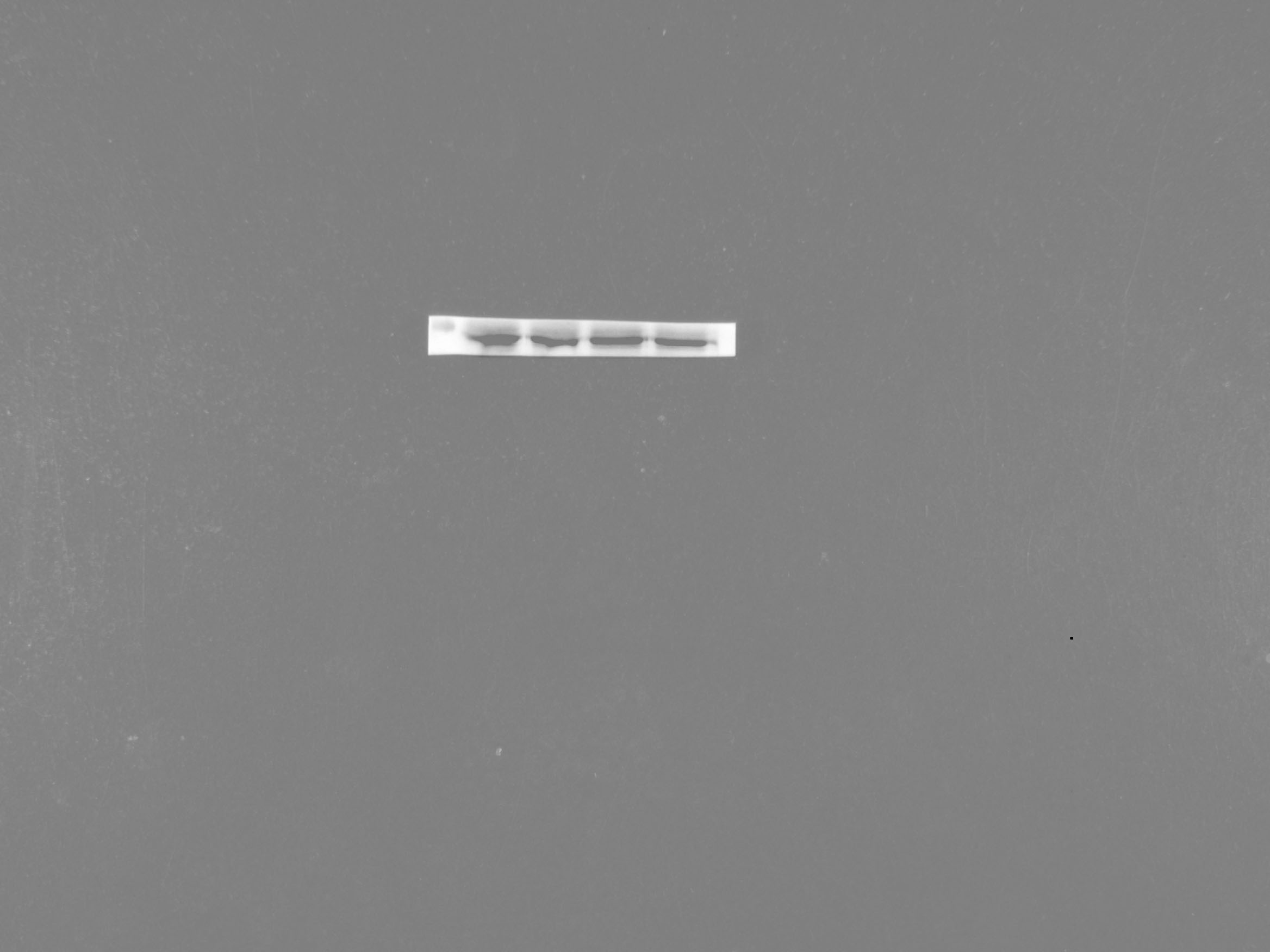

Supplement: Original Images for Blots.zip [file YRER_A_2313366_SM3875.zip › Original Images for Blots/Figure 4/Figure 4B/JNK signaling pathway/STAT3/Marker+STAT3.jpg]

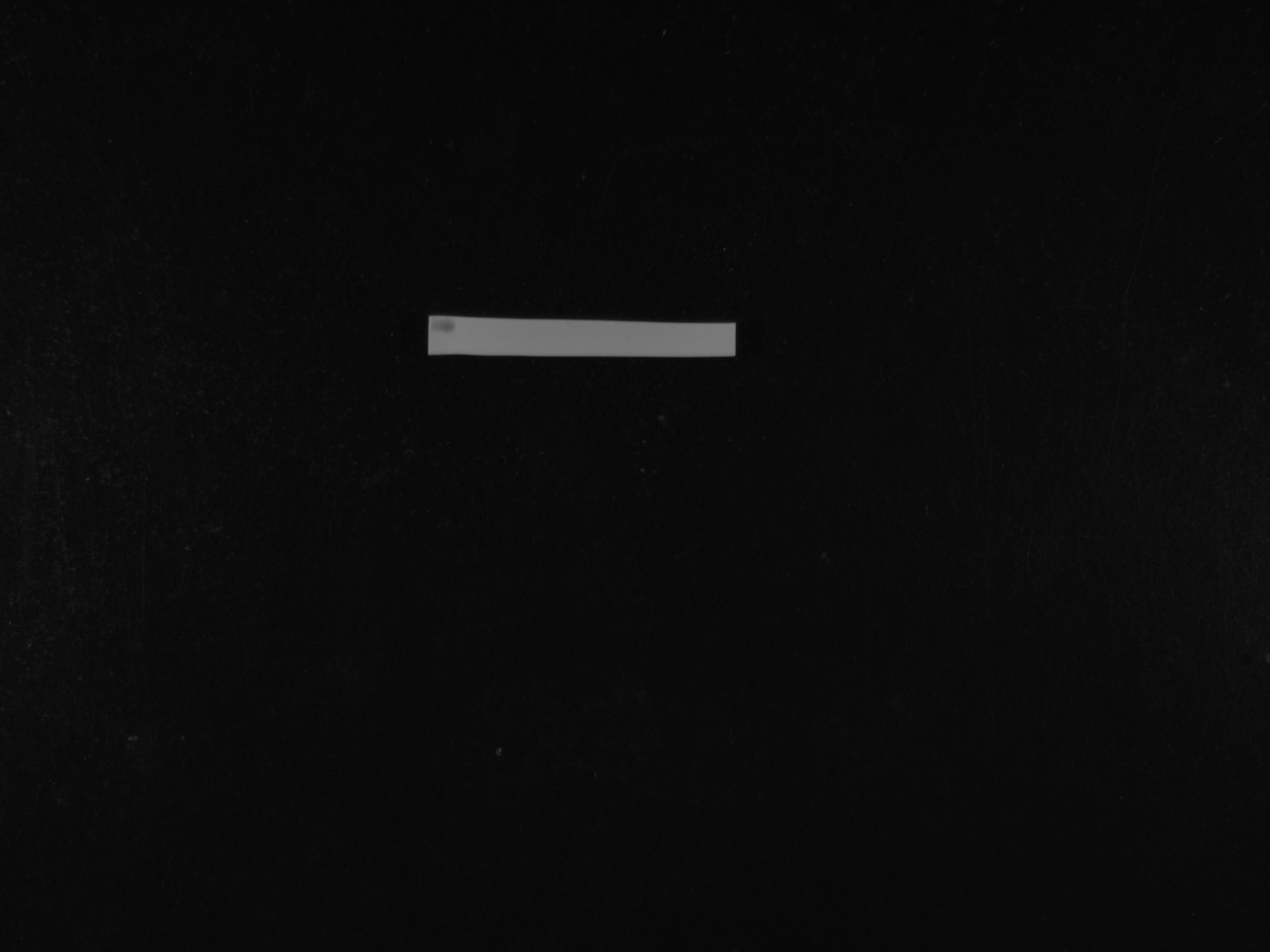

Supplement: Original Images for Blots.zip [file YRER_A_2313366_SM3875.zip › Original Images for Blots/Figure 4/Figure 4B/JNK signaling pathway/STAT3/Marker.jpg]

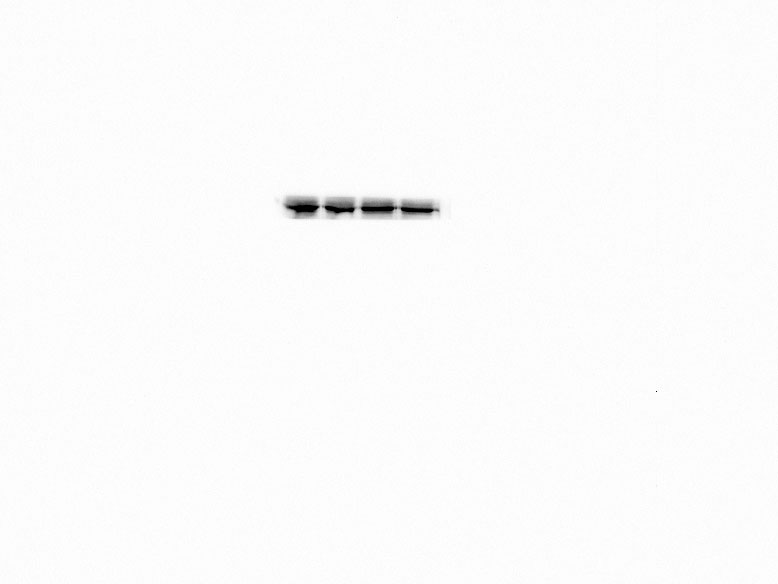

Supplement: Original Images for Blots.zip [file YRER_A_2313366_SM3875.zip › Original Images for Blots/Figure 4/Figure 4B/JNK signaling pathway/STAT3/STAT3.jpg]

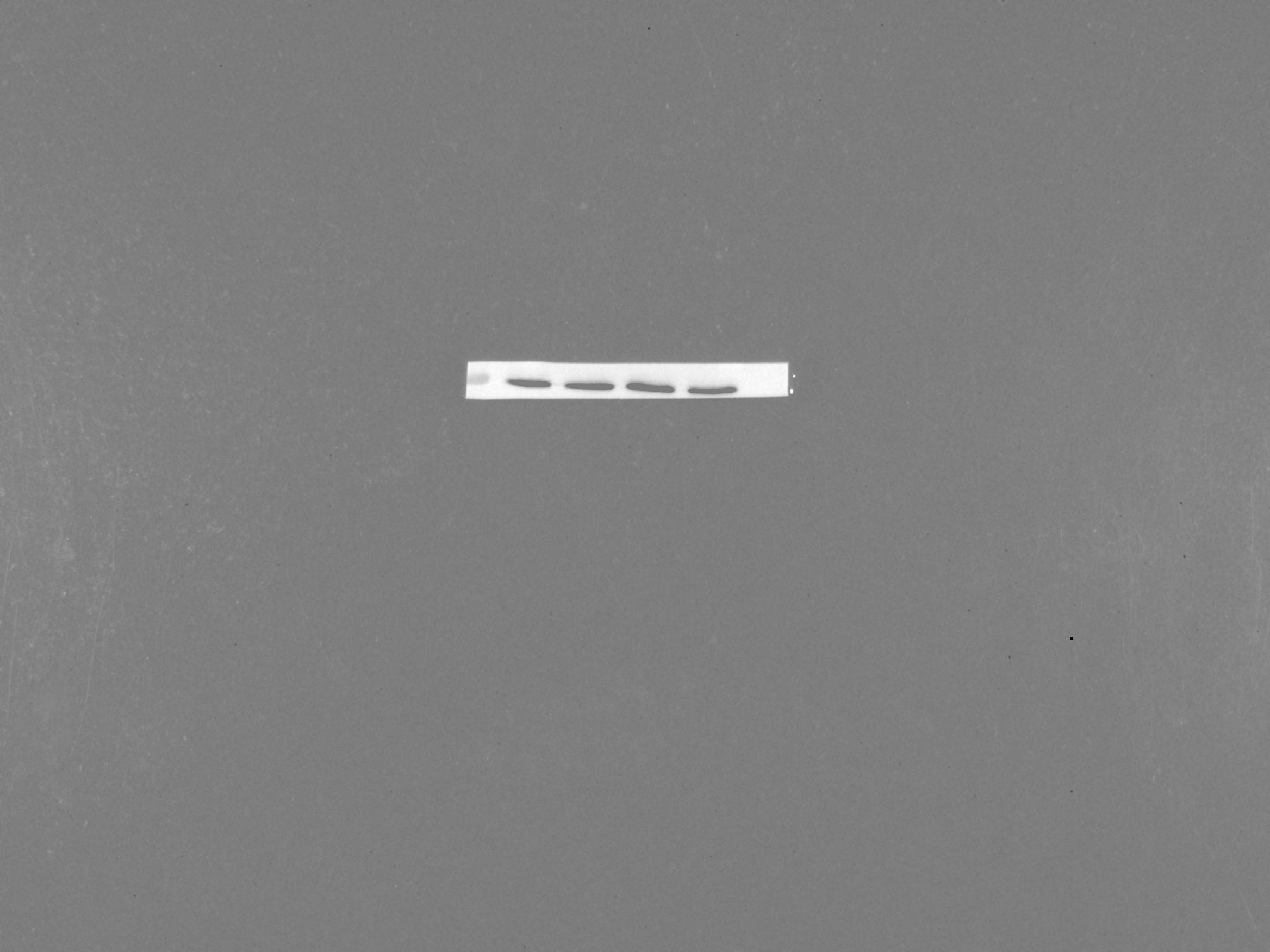

Supplement: Original Images for Blots.zip [file YRER_A_2313366_SM3875.zip › Original Images for Blots/Figure 4/Figure 4B/JNK signaling pathway/α-tubulin/Marker+α-tubulin.jpg]

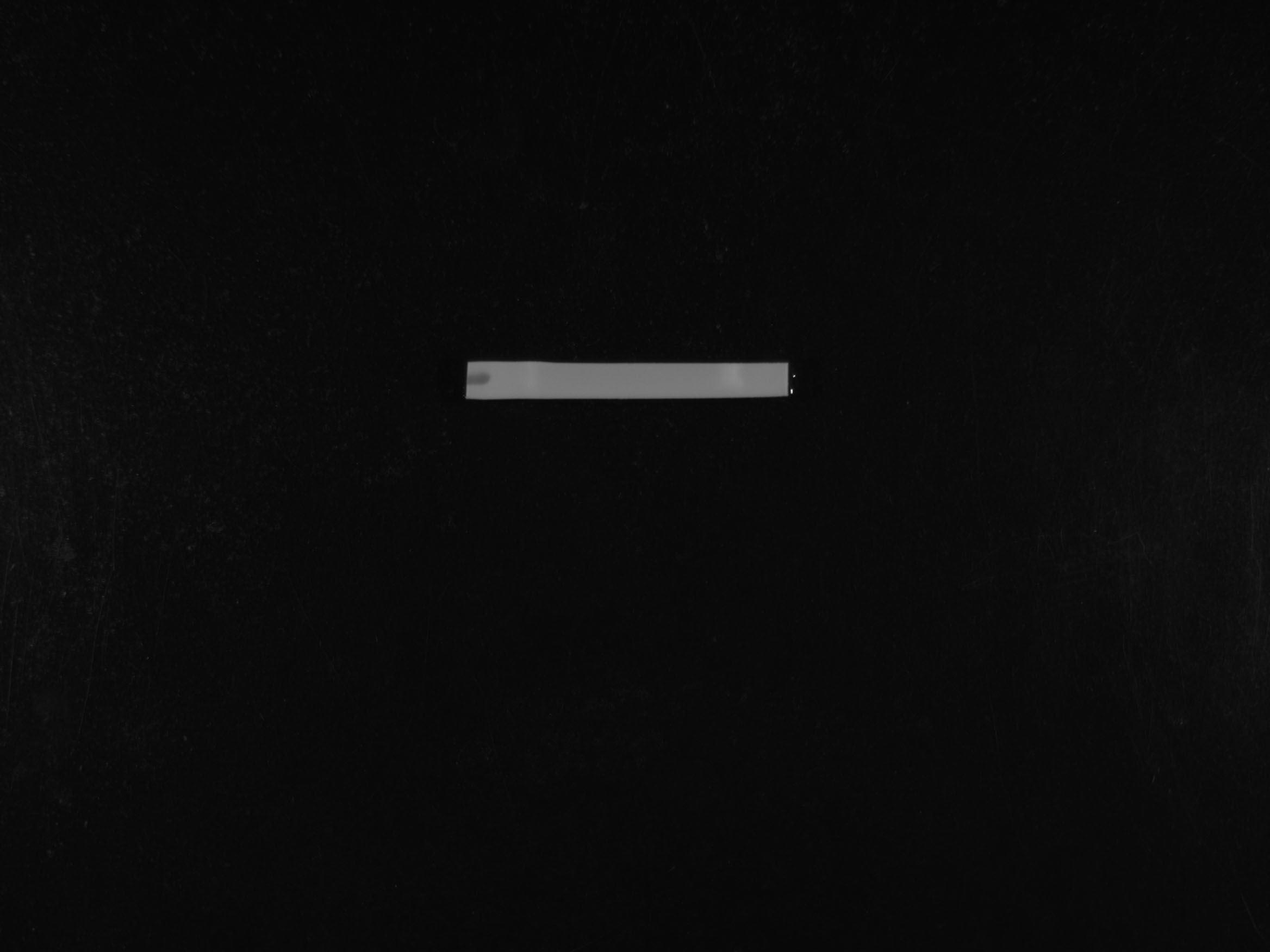

Supplement: Original Images for Blots.zip [file YRER_A_2313366_SM3875.zip › Original Images for Blots/Figure 4/Figure 4B/JNK signaling pathway/α-tubulin/Marker.jpg]

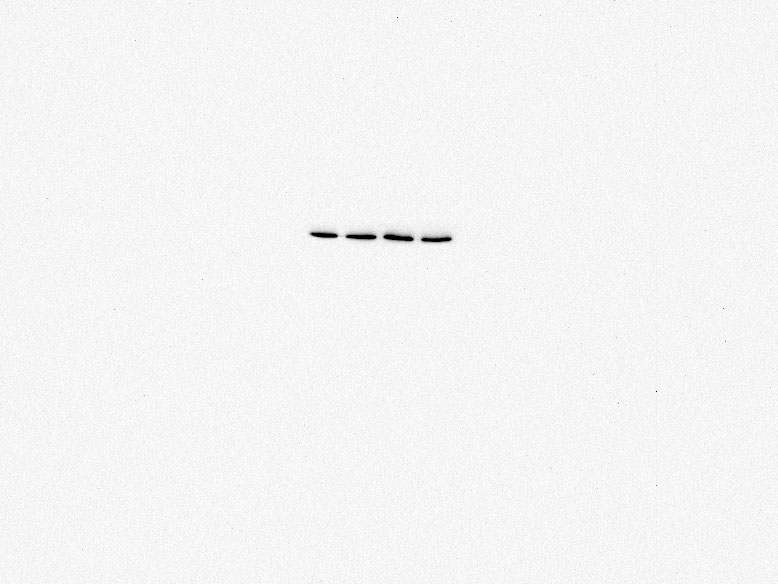

Supplement: Original Images for Blots.zip [file YRER_A_2313366_SM3875.zip › Original Images for Blots/Figure 4/Figure 4B/JNK signaling pathway/α-tubulin/α-tubulin.jpg]

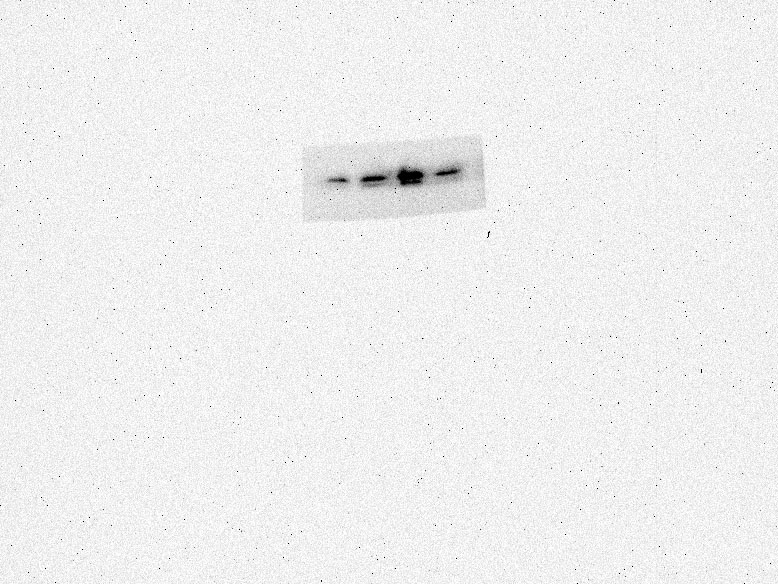

Supplement: Original Images for Blots.zip [file YRER_A_2313366_SM3875.zip › Original Images for Blots/Figure 4/Figure 4B/p38 signaling pathway/cle-caspase-3/cle-caspase-3.jpg]

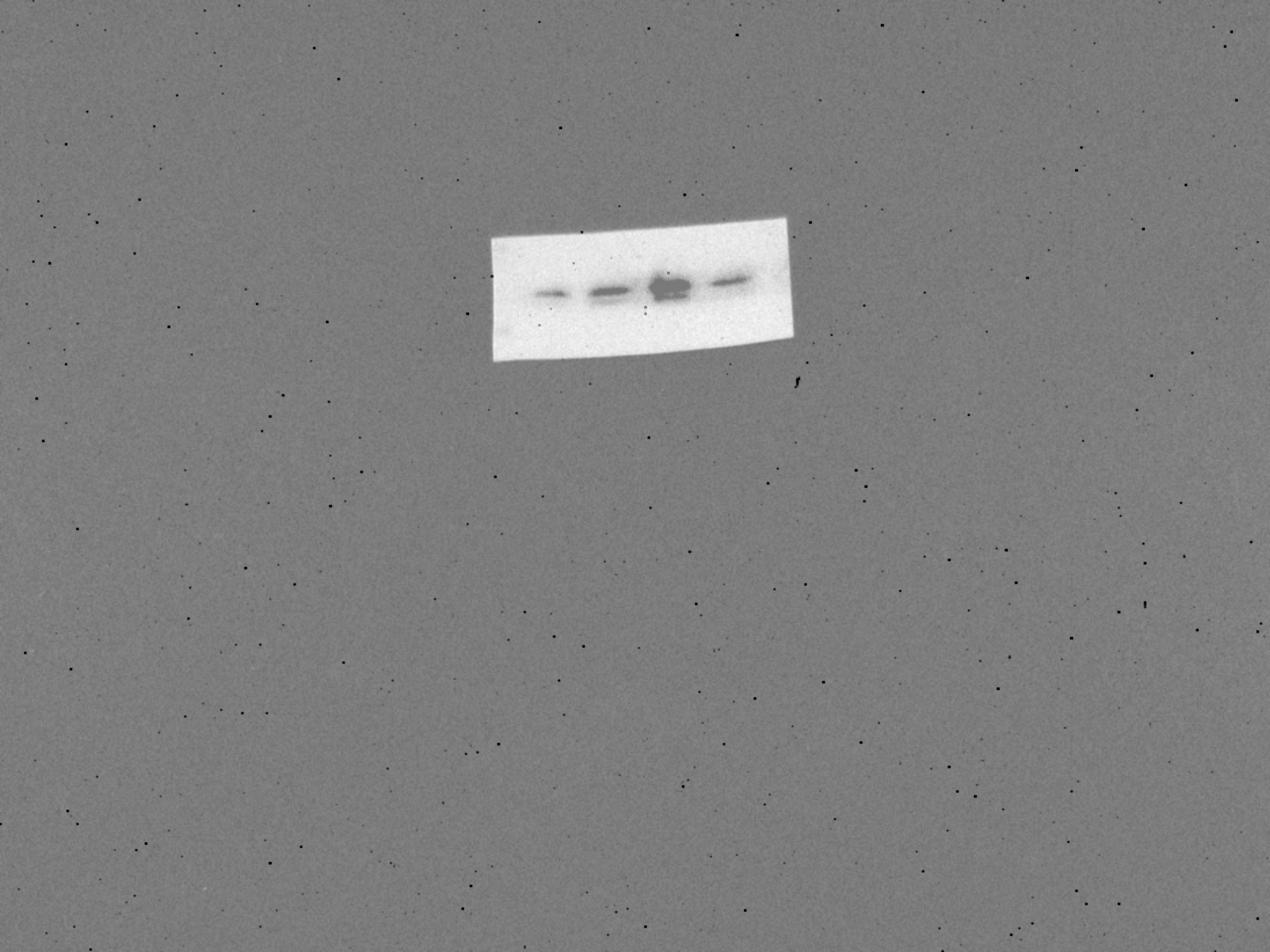

Supplement: Original Images for Blots.zip [file YRER_A_2313366_SM3875.zip › Original Images for Blots/Figure 4/Figure 4B/p38 signaling pathway/cle-caspase-3/Marker+cle-caspase-3.jpg]

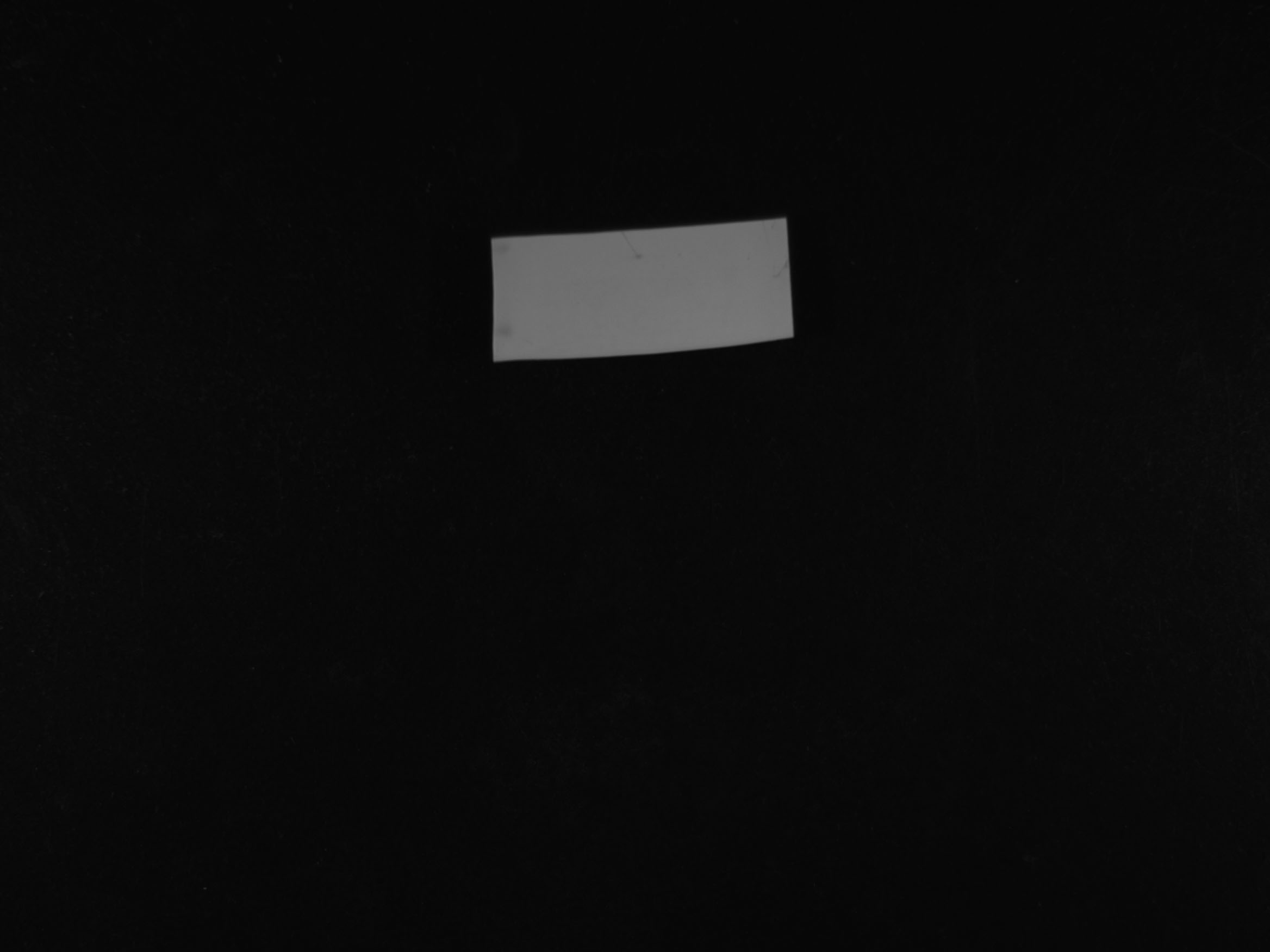

Supplement: Original Images for Blots.zip [file YRER_A_2313366_SM3875.zip › Original Images for Blots/Figure 4/Figure 4B/p38 signaling pathway/cle-caspase-3/Marker.jpg]

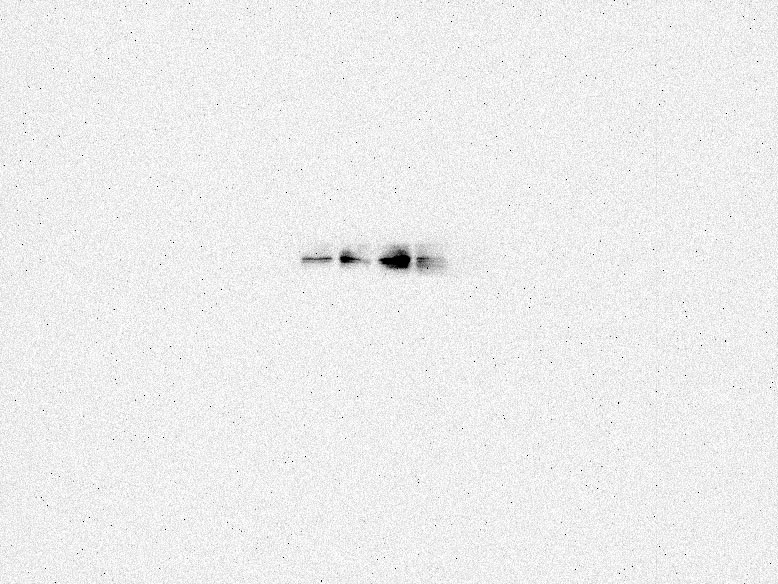

Supplement: Original Images for Blots.zip [file YRER_A_2313366_SM3875.zip › Original Images for Blots/Figure 4/Figure 4B/p38 signaling pathway/cle-PARP/cle-PARP.jpg]

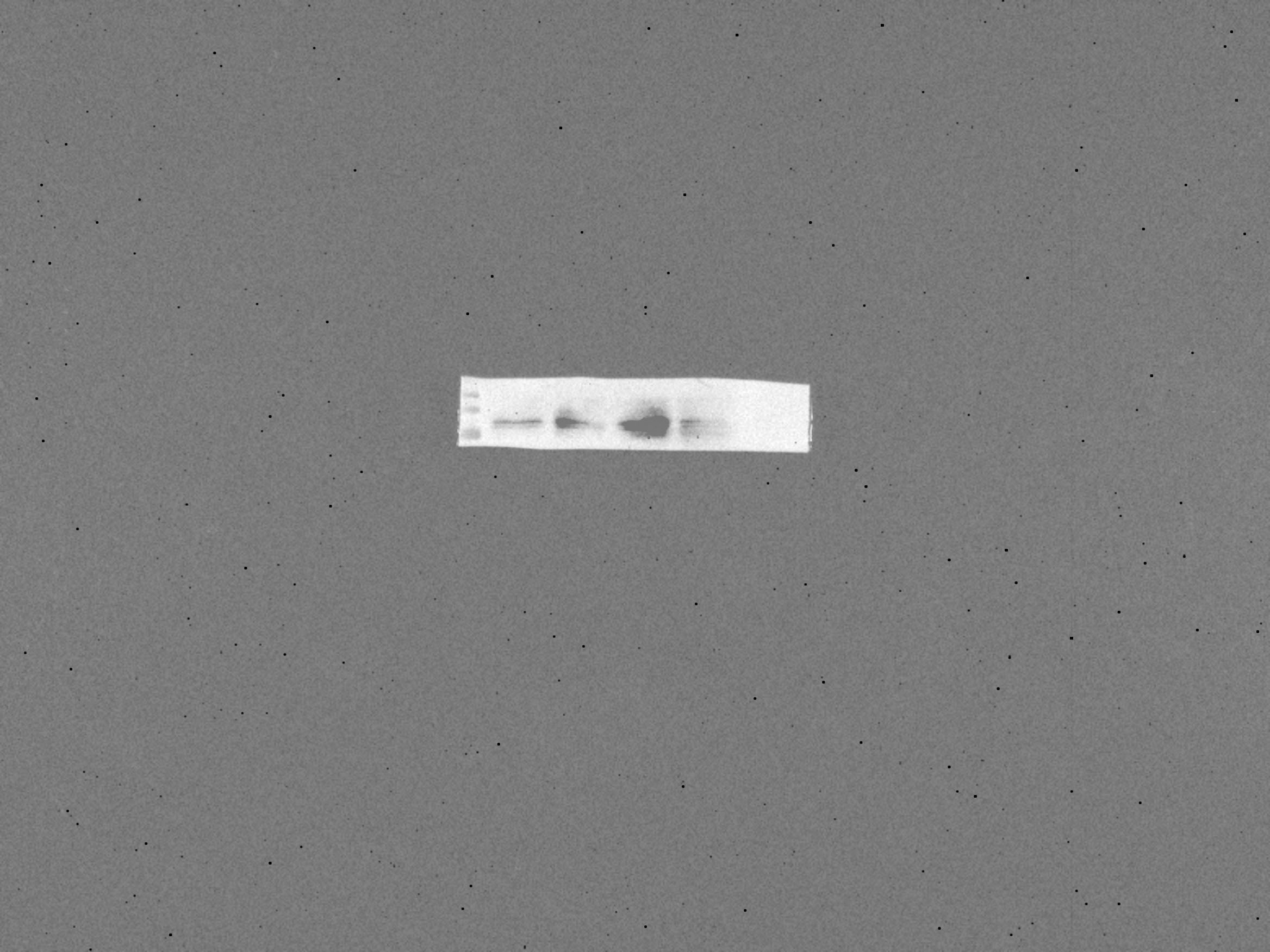

Supplement: Original Images for Blots.zip [file YRER_A_2313366_SM3875.zip › Original Images for Blots/Figure 4/Figure 4B/p38 signaling pathway/cle-PARP/Marker+cle-PARP.jpg]

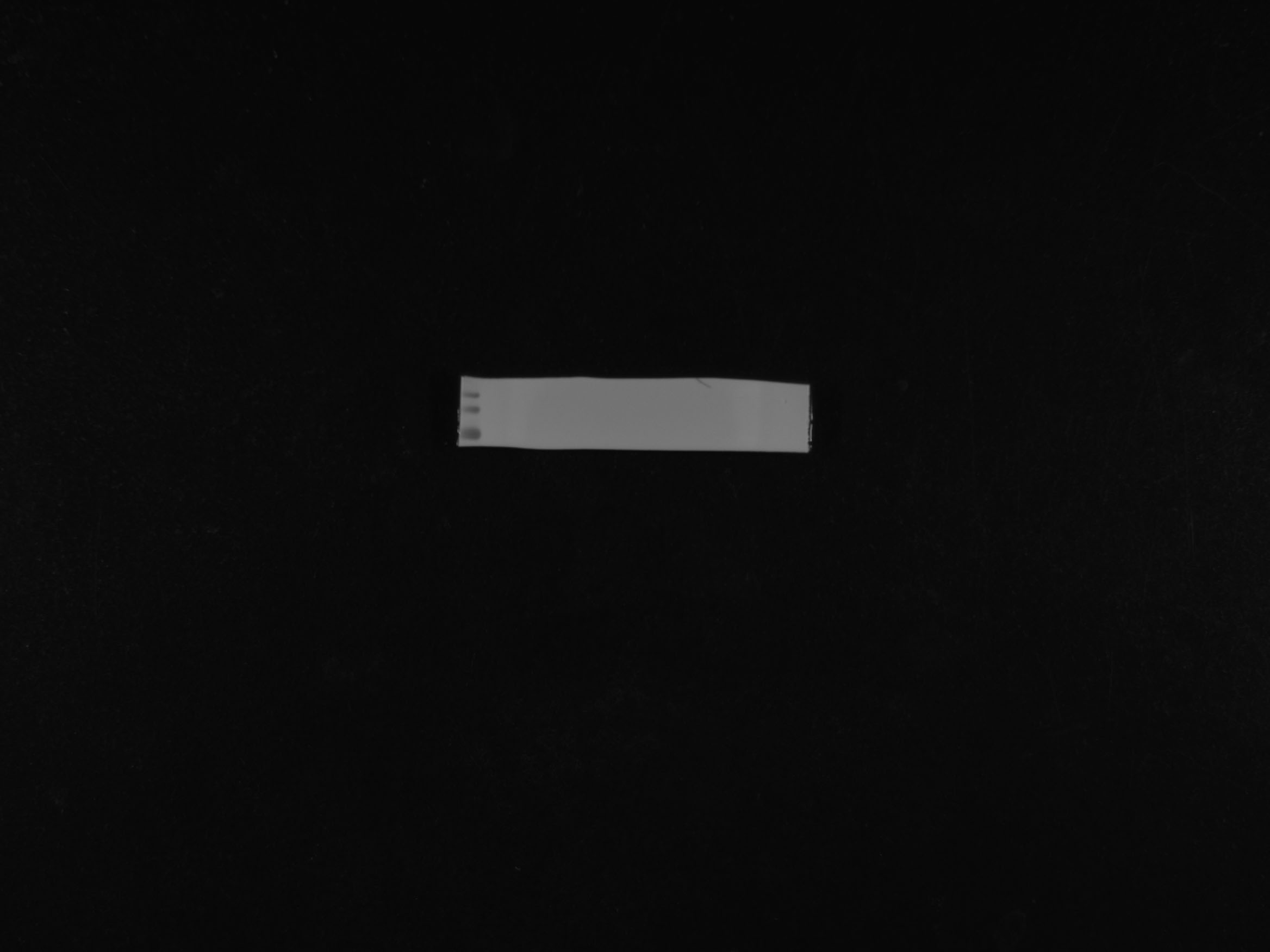

Supplement: Original Images for Blots.zip [file YRER_A_2313366_SM3875.zip › Original Images for Blots/Figure 4/Figure 4B/p38 signaling pathway/cle-PARP/Marker.jpg]

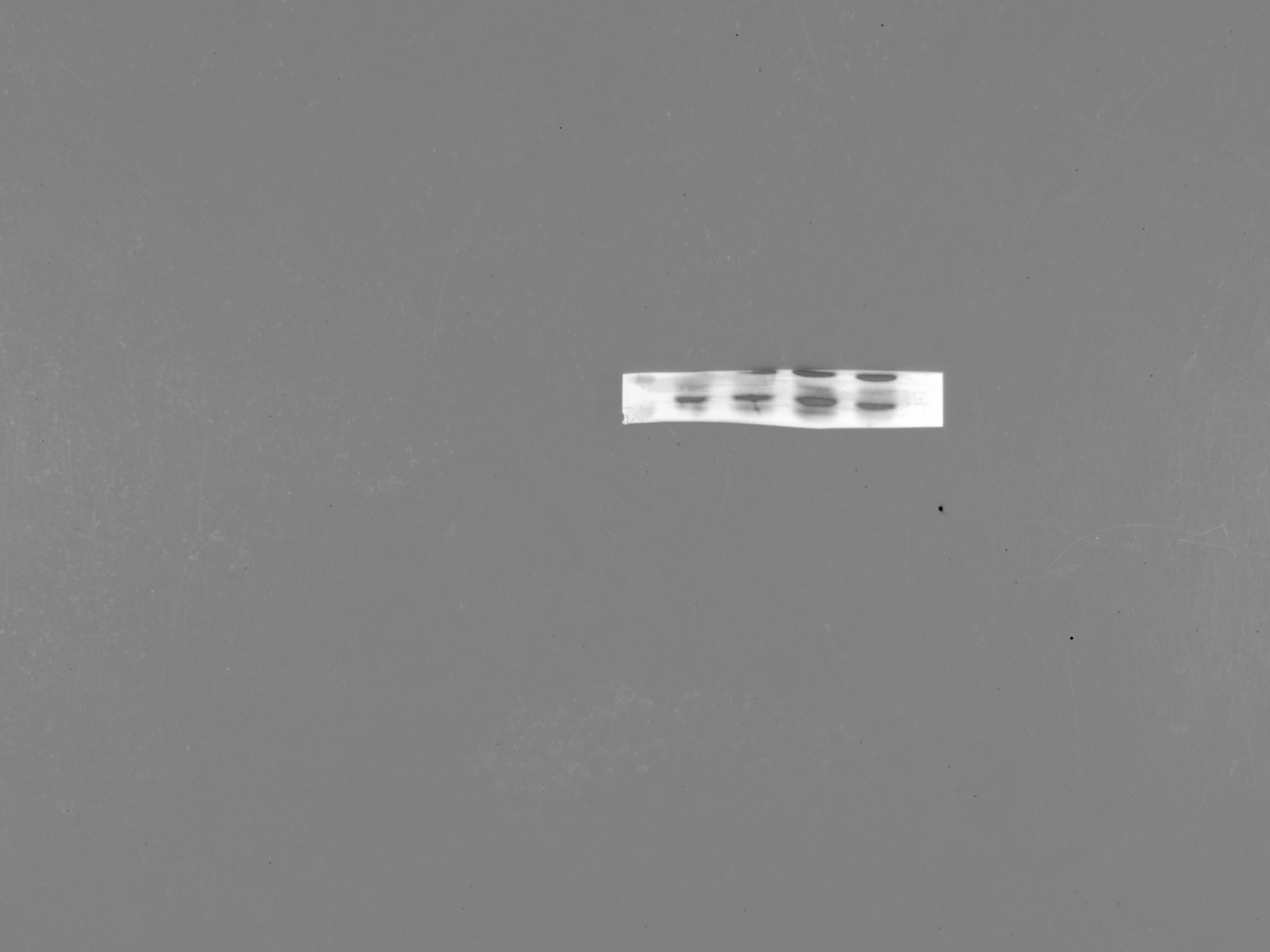

Supplement: Original Images for Blots.zip [file YRER_A_2313366_SM3875.zip › Original Images for Blots/Figure 4/Figure 4B/p38 signaling pathway/p-p38/Marker+p-p38.jpg]

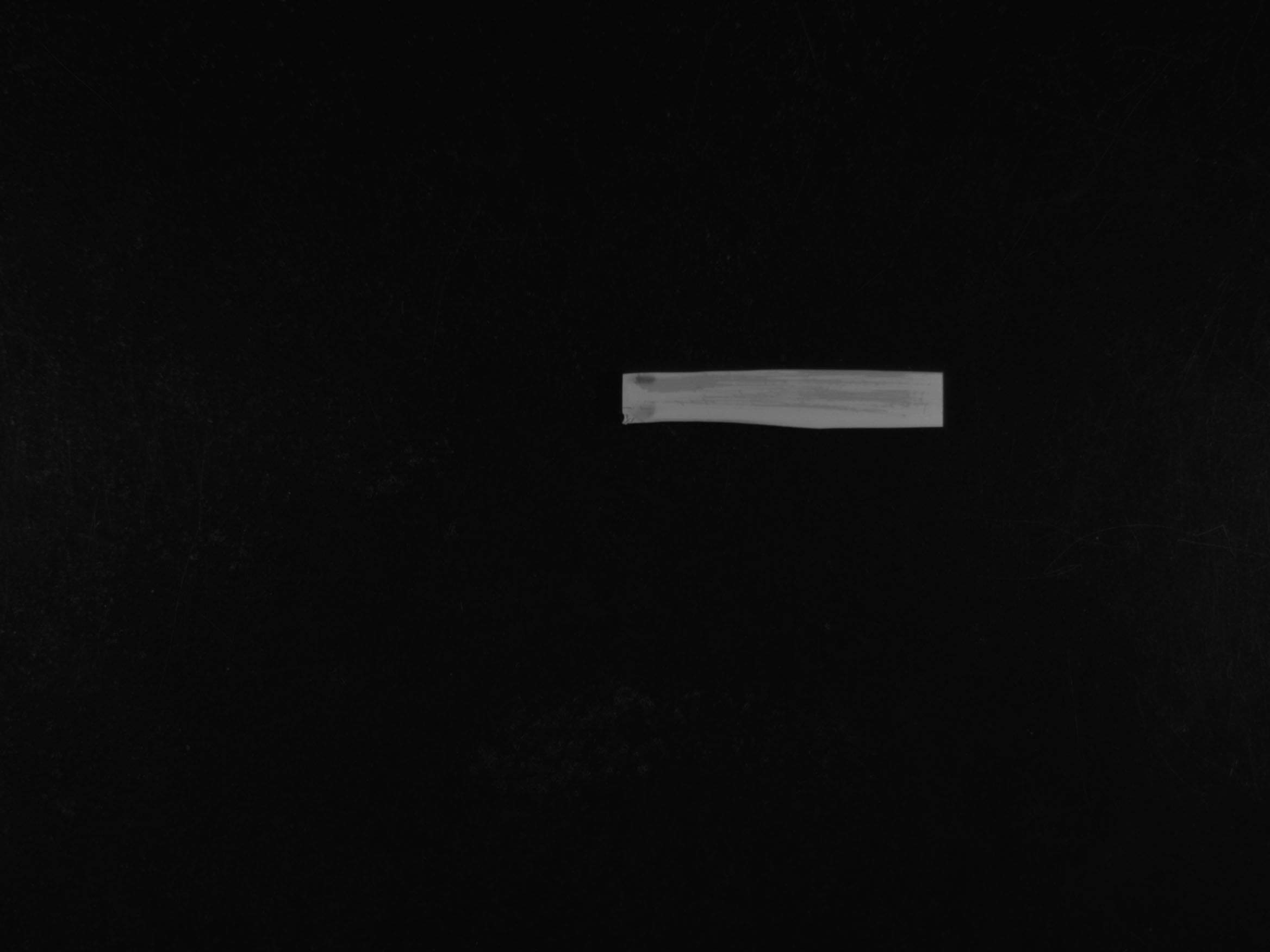

Supplement: Original Images for Blots.zip [file YRER_A_2313366_SM3875.zip › Original Images for Blots/Figure 4/Figure 4B/p38 signaling pathway/p-p38/Marker.jpg]

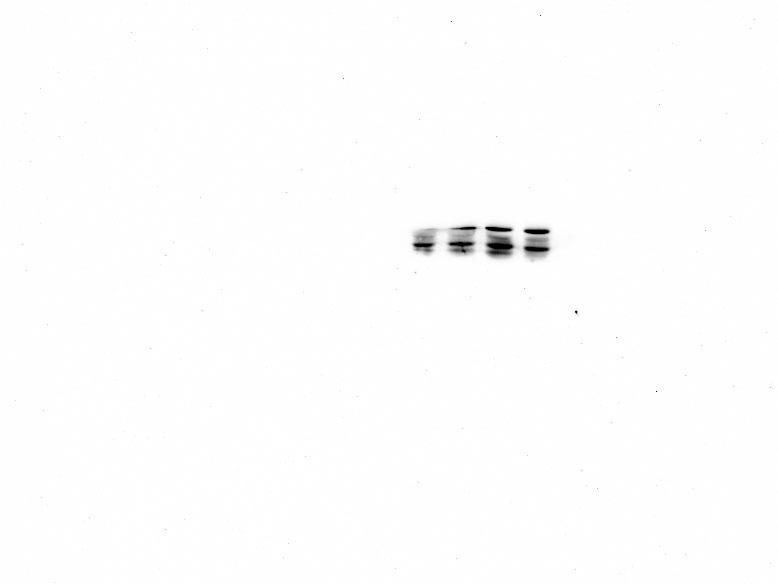

Supplement: Original Images for Blots.zip [file YRER_A_2313366_SM3875.zip › Original Images for Blots/Figure 4/Figure 4B/p38 signaling pathway/p-p38/p-p38.jpg]

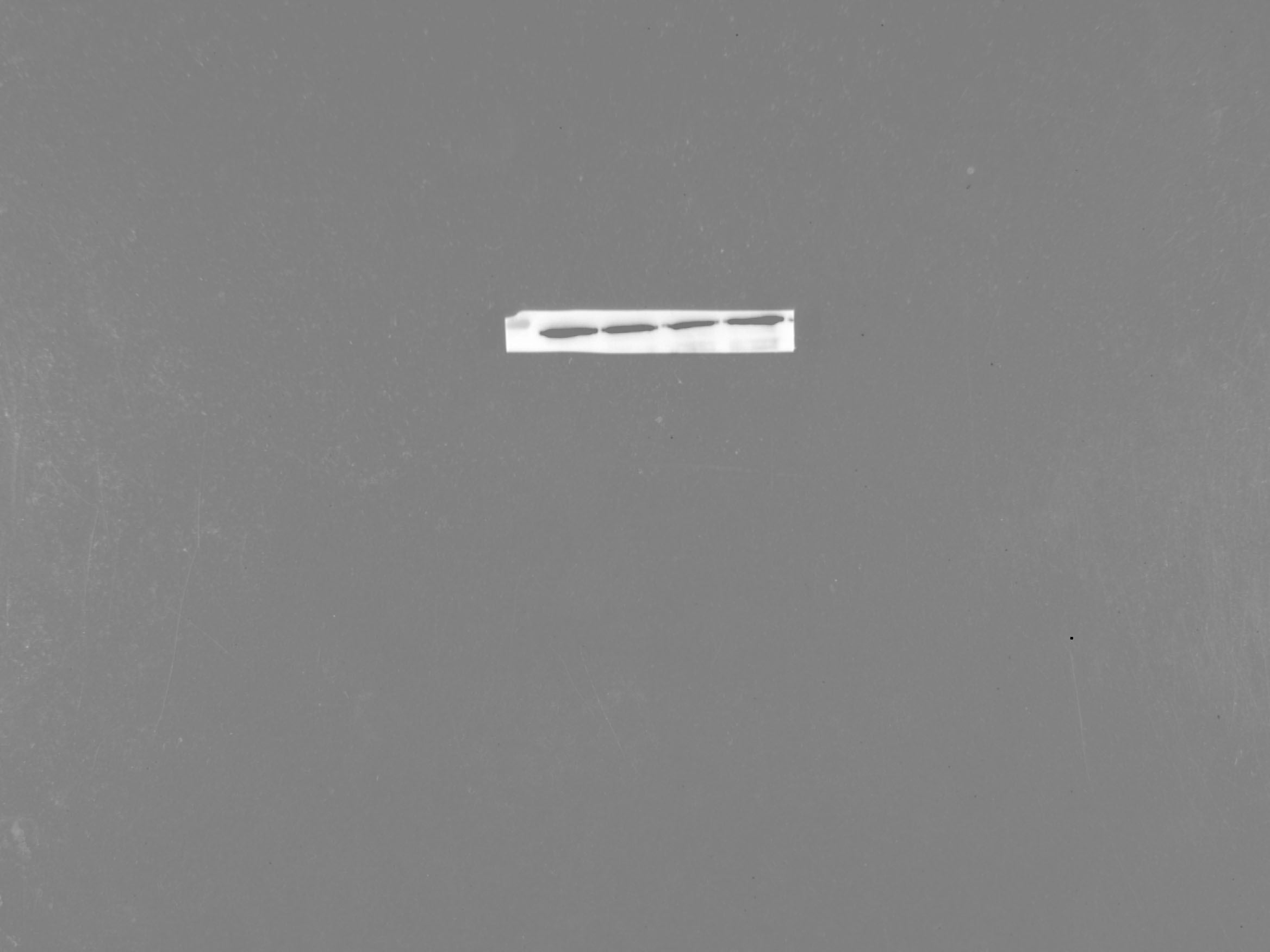

Supplement: Original Images for Blots.zip [file YRER_A_2313366_SM3875.zip › Original Images for Blots/Figure 4/Figure 4B/p38 signaling pathway/p-STAT3/Marker+p-STAT3.jpg]

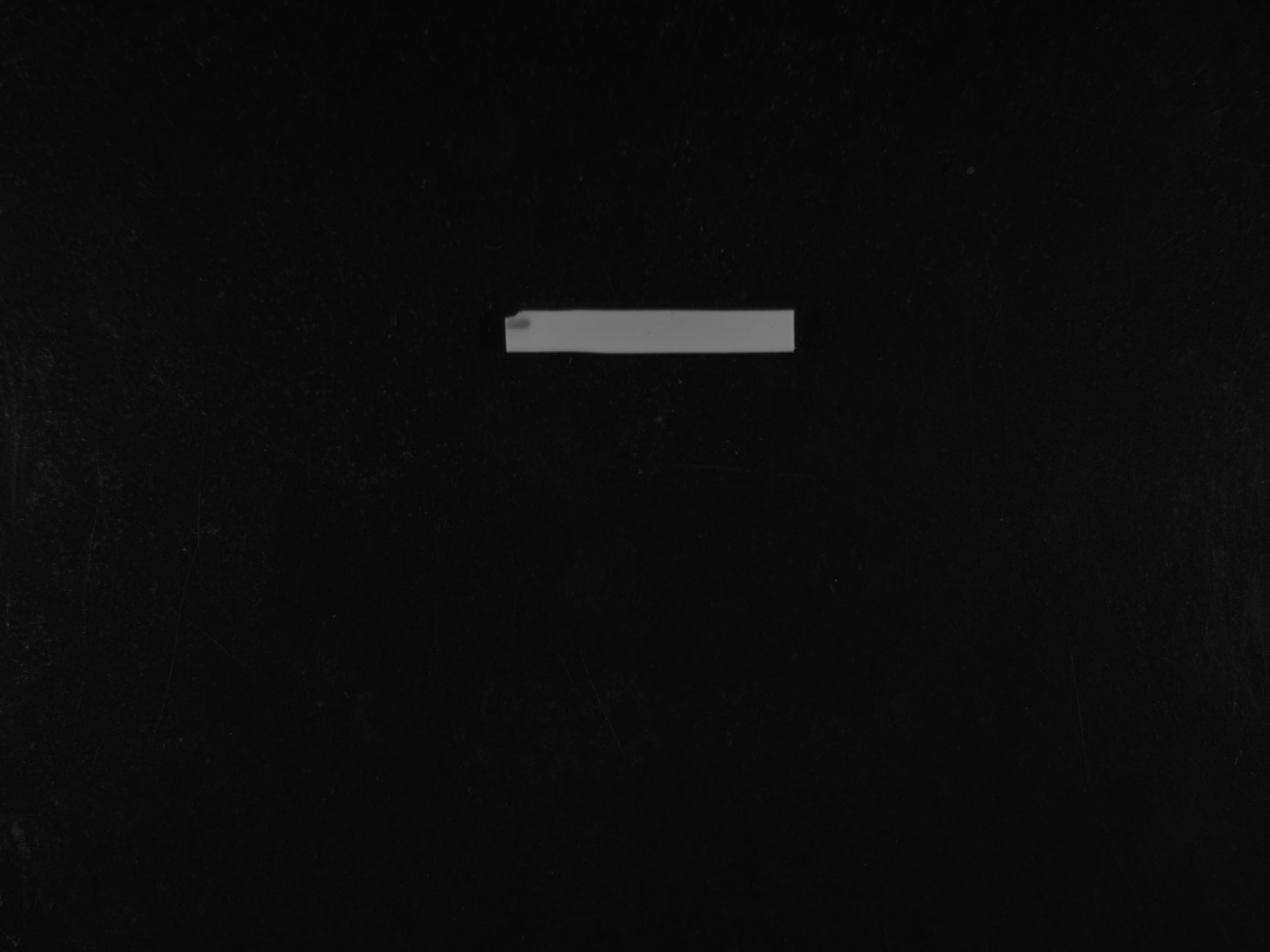

Supplement: Original Images for Blots.zip [file YRER_A_2313366_SM3875.zip › Original Images for Blots/Figure 4/Figure 4B/p38 signaling pathway/p-STAT3/Marker.jpg]

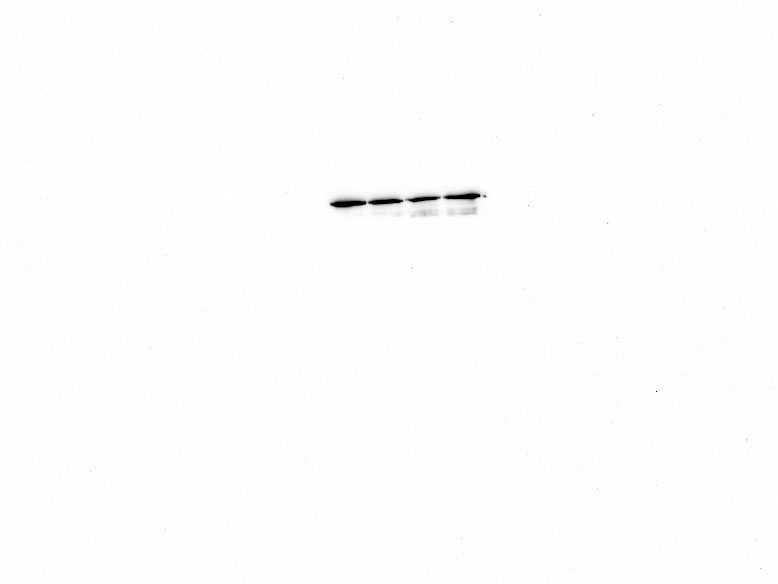

Supplement: Original Images for Blots.zip [file YRER_A_2313366_SM3875.zip › Original Images for Blots/Figure 4/Figure 4B/p38 signaling pathway/p-STAT3/p-STAT3.jpg]

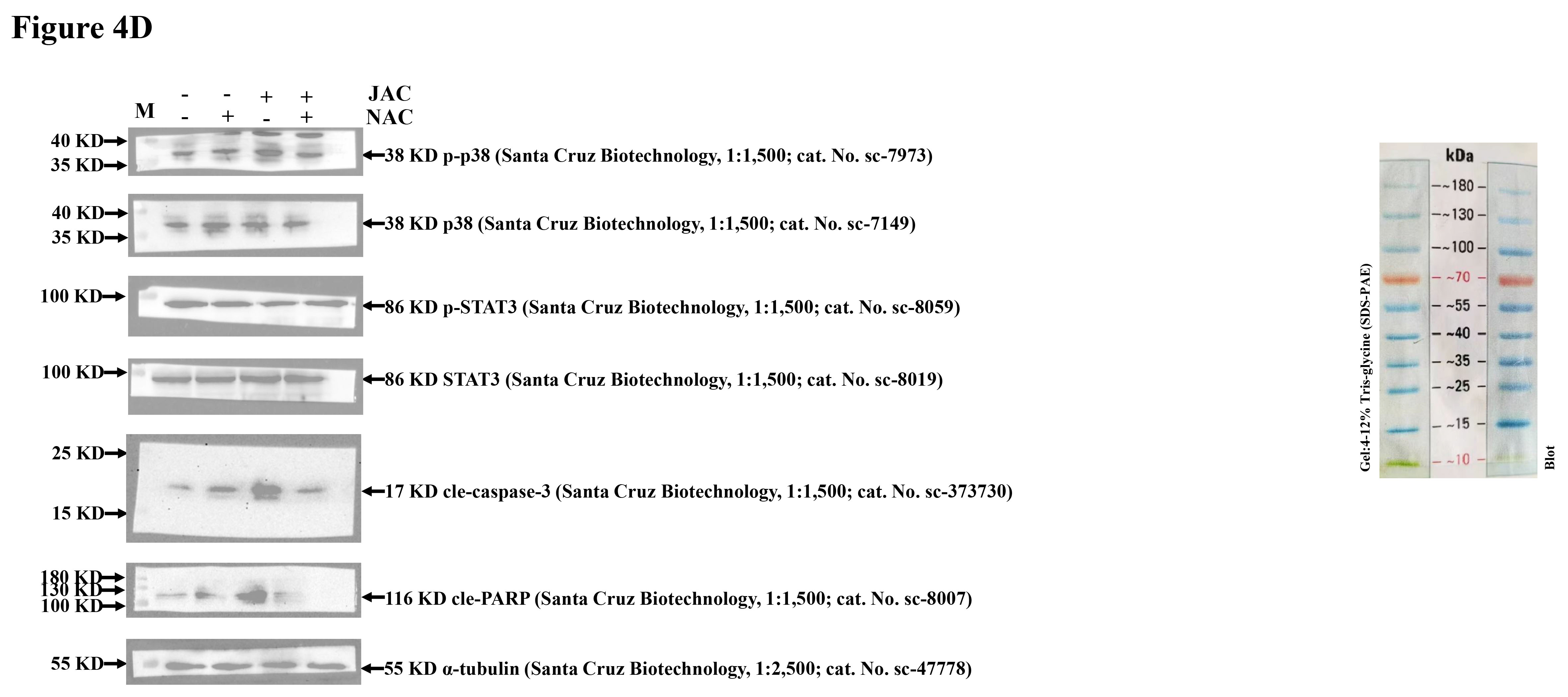

Supplement: Original Images for Blots.zip [file YRER_A_2313366_SM3875.zip › Original Images for Blots/Figure 4/Figure 4B/p38 signaling pathway/p38 signaling pathway.jpg]

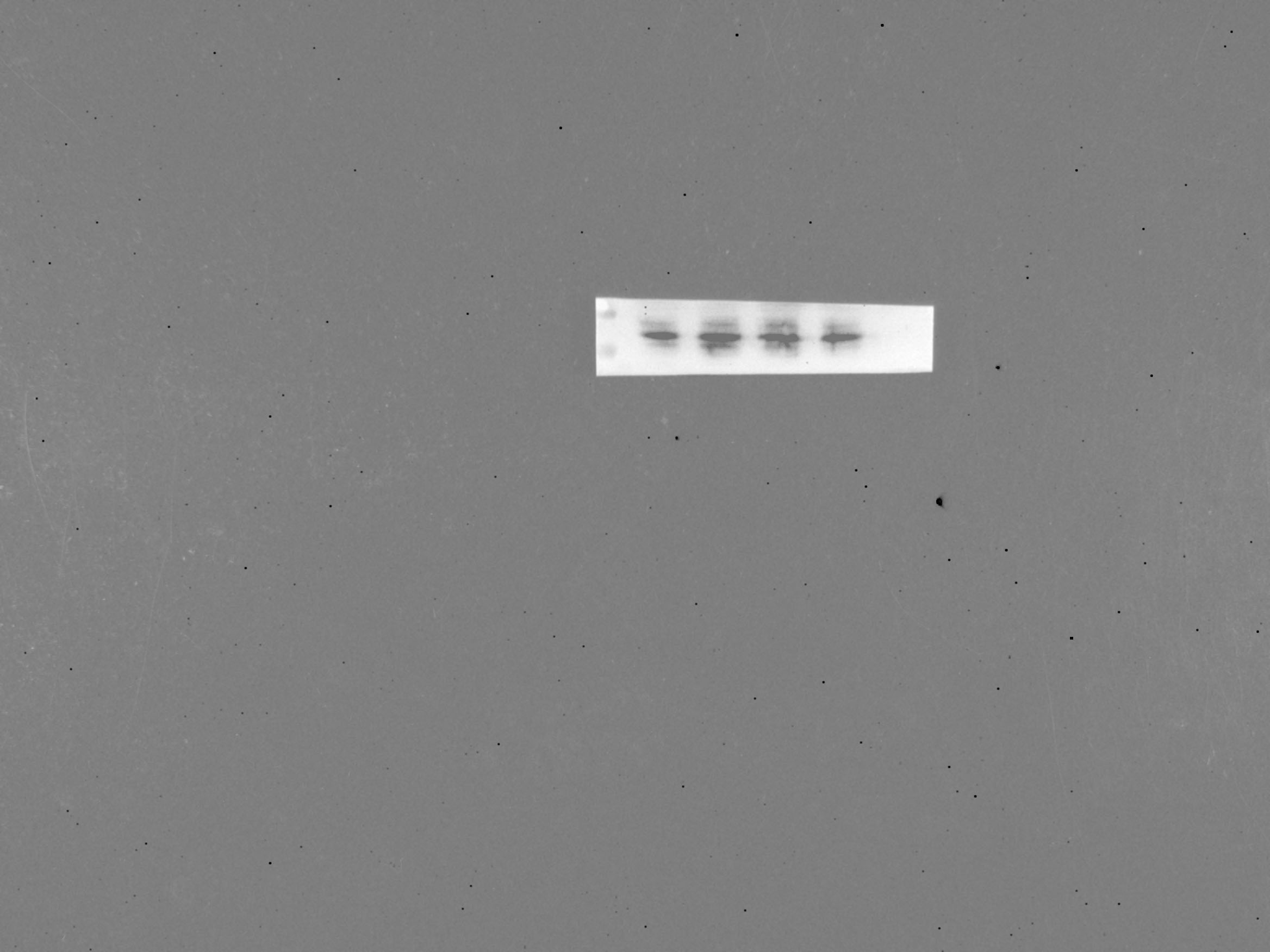

Supplement: Original Images for Blots.zip [file YRER_A_2313366_SM3875.zip › Original Images for Blots/Figure 4/Figure 4B/p38 signaling pathway/p38/Marker+p38.jpg]

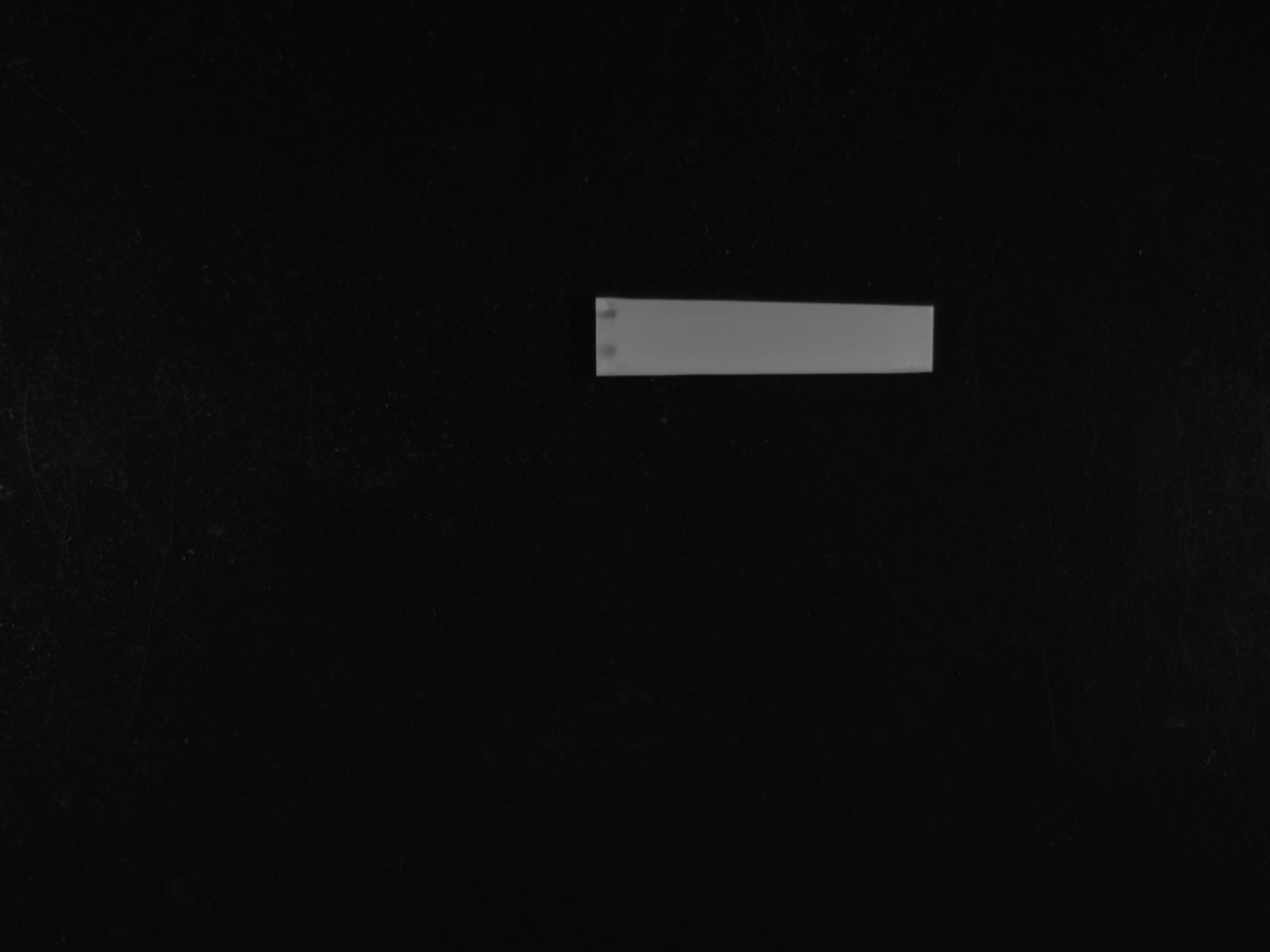

Supplement: Original Images for Blots.zip [file YRER_A_2313366_SM3875.zip › Original Images for Blots/Figure 4/Figure 4B/p38 signaling pathway/p38/Marker.jpg]

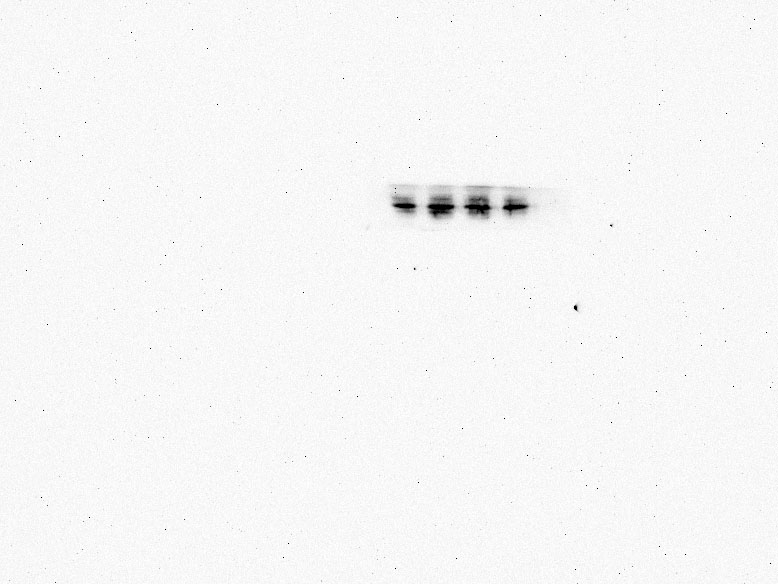

Supplement: Original Images for Blots.zip [file YRER_A_2313366_SM3875.zip › Original Images for Blots/Figure 4/Figure 4B/p38 signaling pathway/p38/p38.jpg]

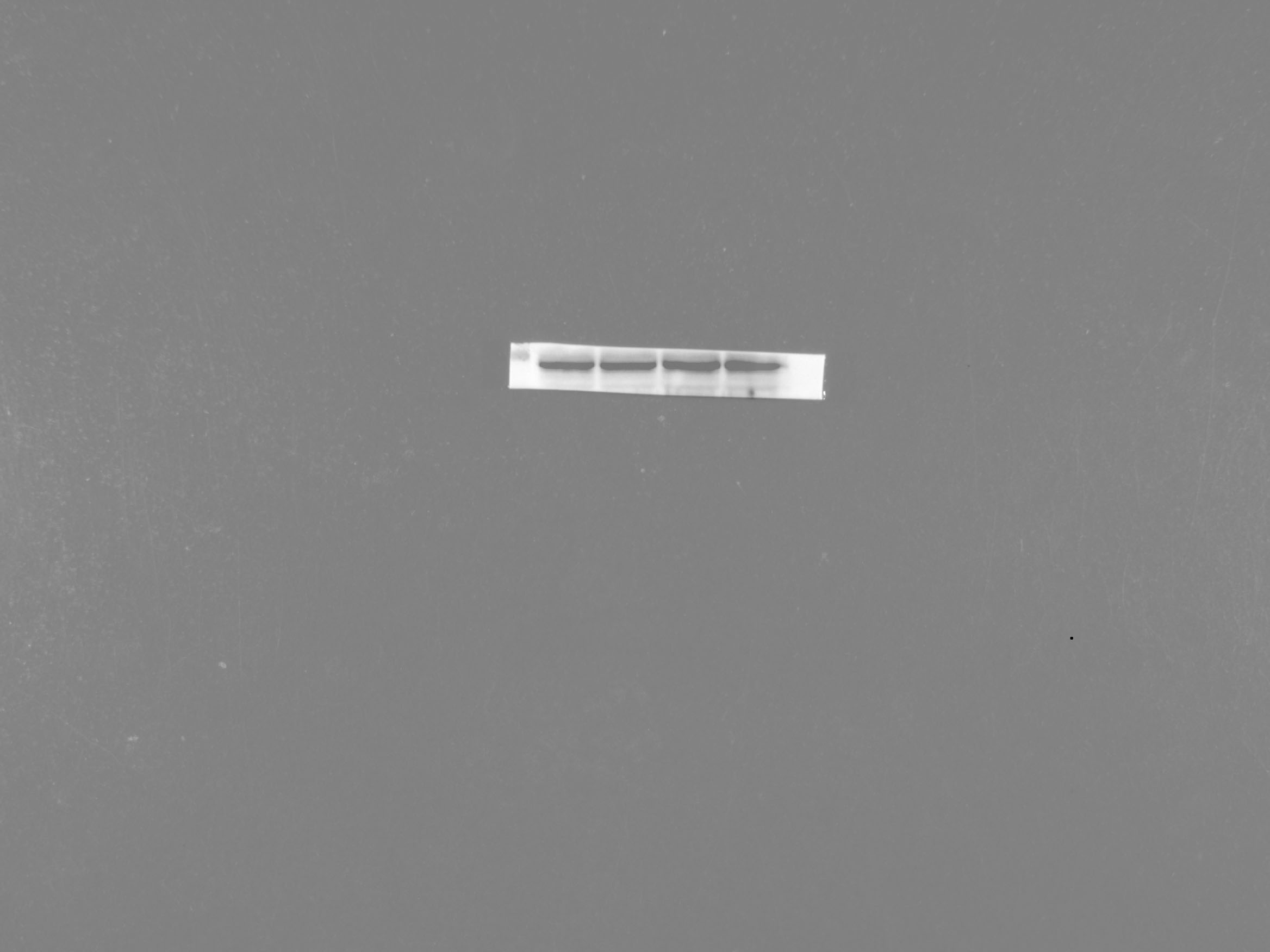

Supplement: Original Images for Blots.zip [file YRER_A_2313366_SM3875.zip › Original Images for Blots/Figure 4/Figure 4B/p38 signaling pathway/STAT3/Marker+STAT3.jpg]

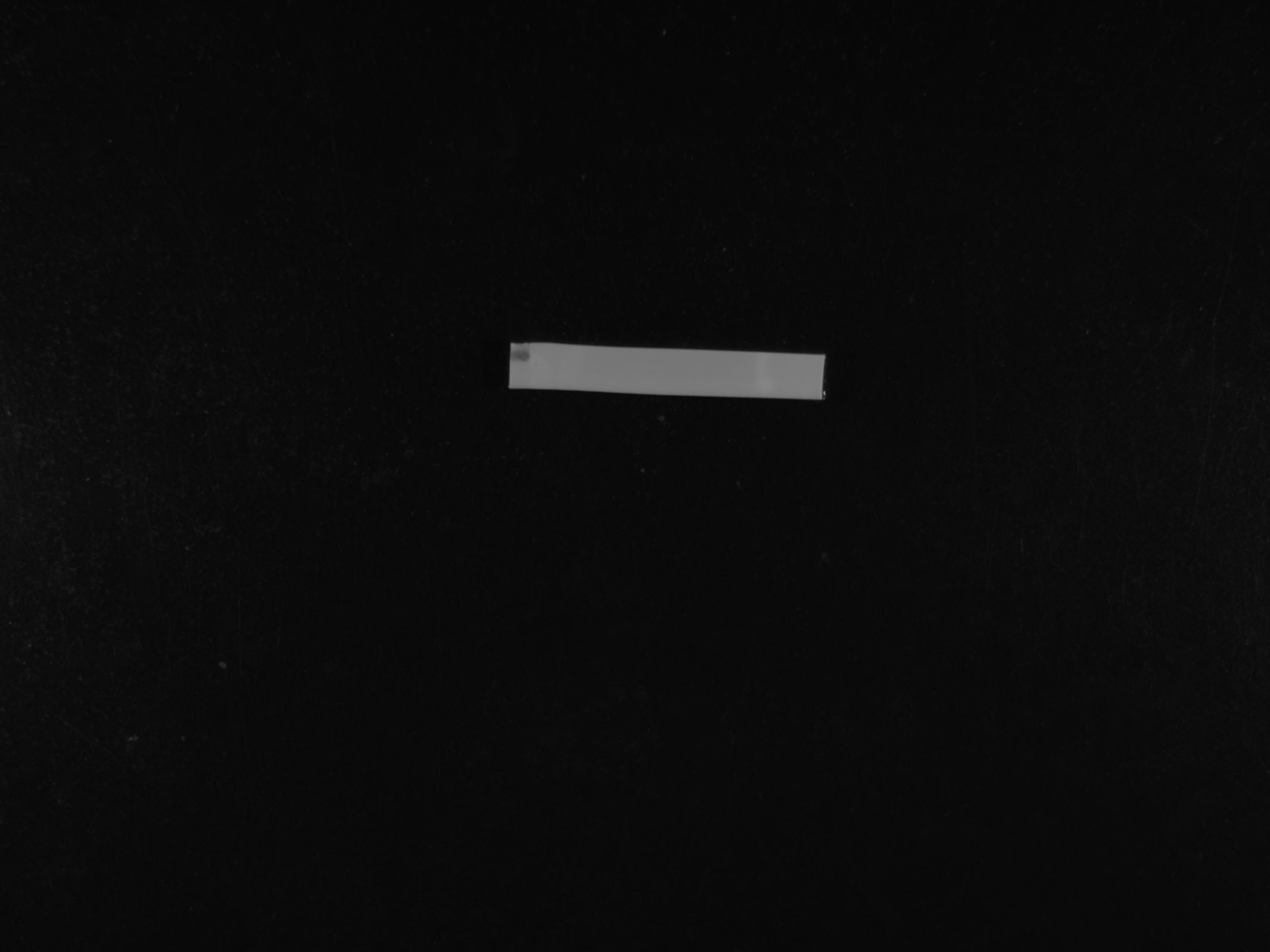

Supplement: Original Images for Blots.zip [file YRER_A_2313366_SM3875.zip › Original Images for Blots/Figure 4/Figure 4B/p38 signaling pathway/STAT3/Marker.jpg]

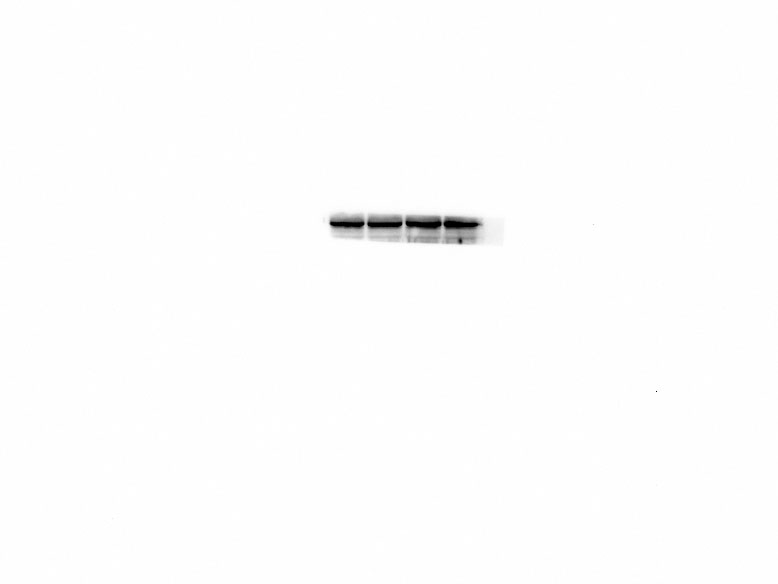

Supplement: Original Images for Blots.zip [file YRER_A_2313366_SM3875.zip › Original Images for Blots/Figure 4/Figure 4B/p38 signaling pathway/STAT3/STAT3.jpg]

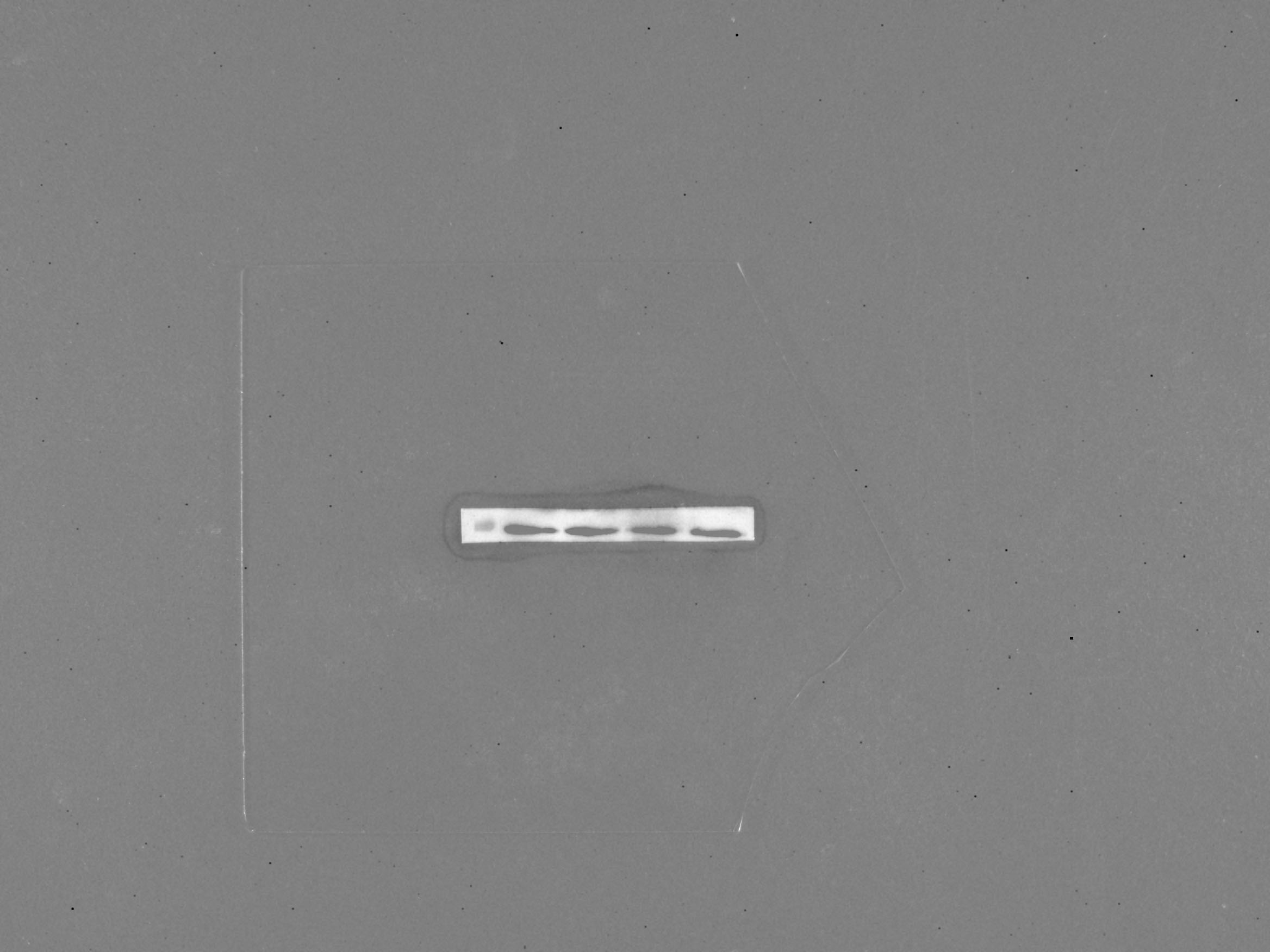

Supplement: Original Images for Blots.zip [file YRER_A_2313366_SM3875.zip › Original Images for Blots/Figure 4/Figure 4B/p38 signaling pathway/α-tubulin/Marker+α-tubulin.jpg]

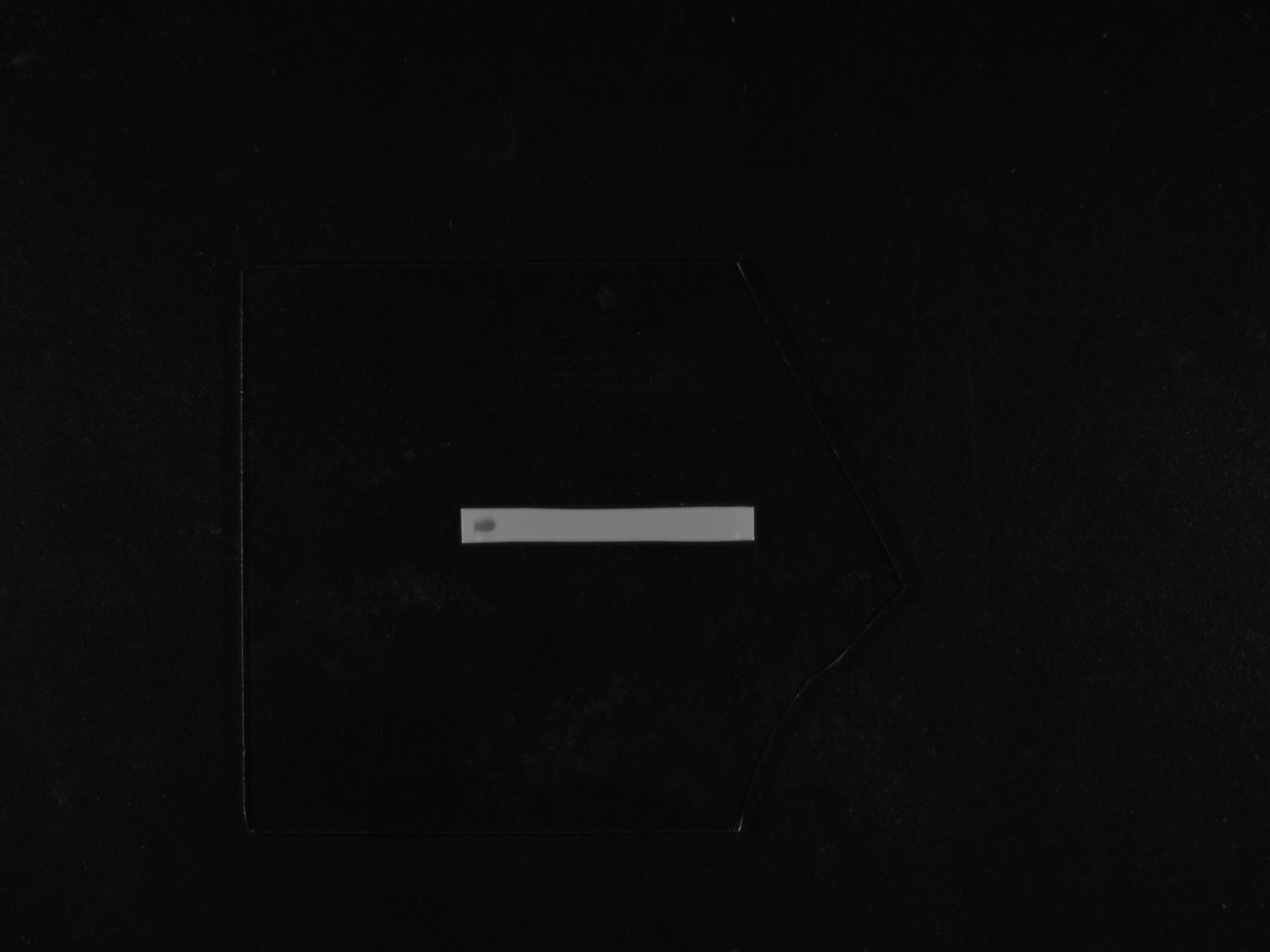

Supplement: Original Images for Blots.zip [file YRER_A_2313366_SM3875.zip › Original Images for Blots/Figure 4/Figure 4B/p38 signaling pathway/α-tubulin/Marker.jpg]

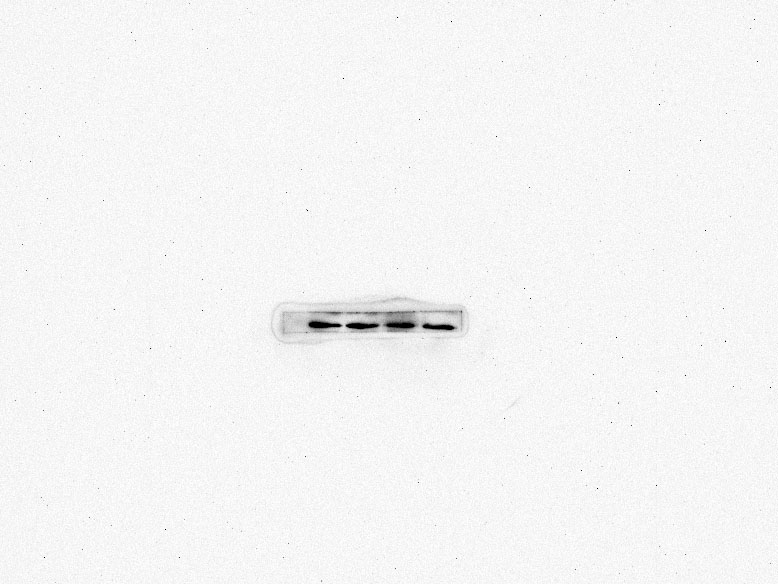

Supplement: Original Images for Blots.zip [file YRER_A_2313366_SM3875.zip › Original Images for Blots/Figure 4/Figure 4B/p38 signaling pathway/α-tubulin/α-tubulin.jpg]

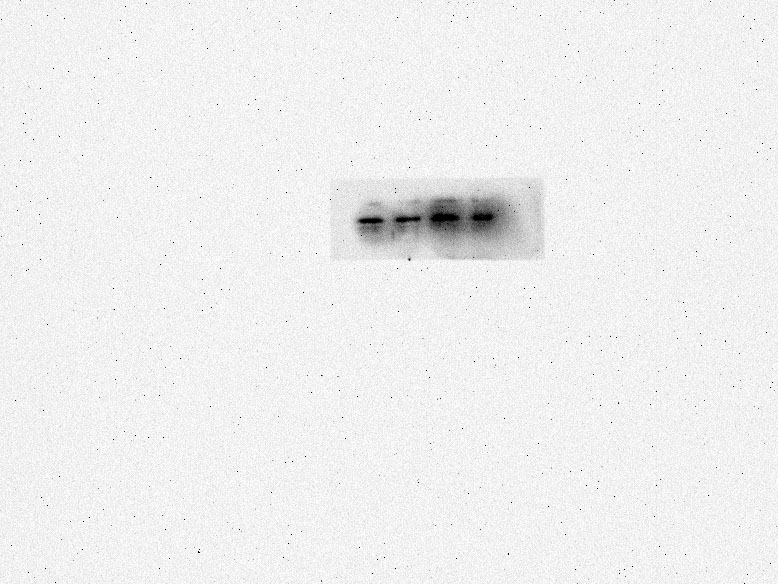

Supplement: Original Images for Blots.zip [file YRER_A_2313366_SM3875.zip › Original Images for Blots/Figure 5/Figure 5D/cle-caspase-3/cle-caspase-3.jpg]

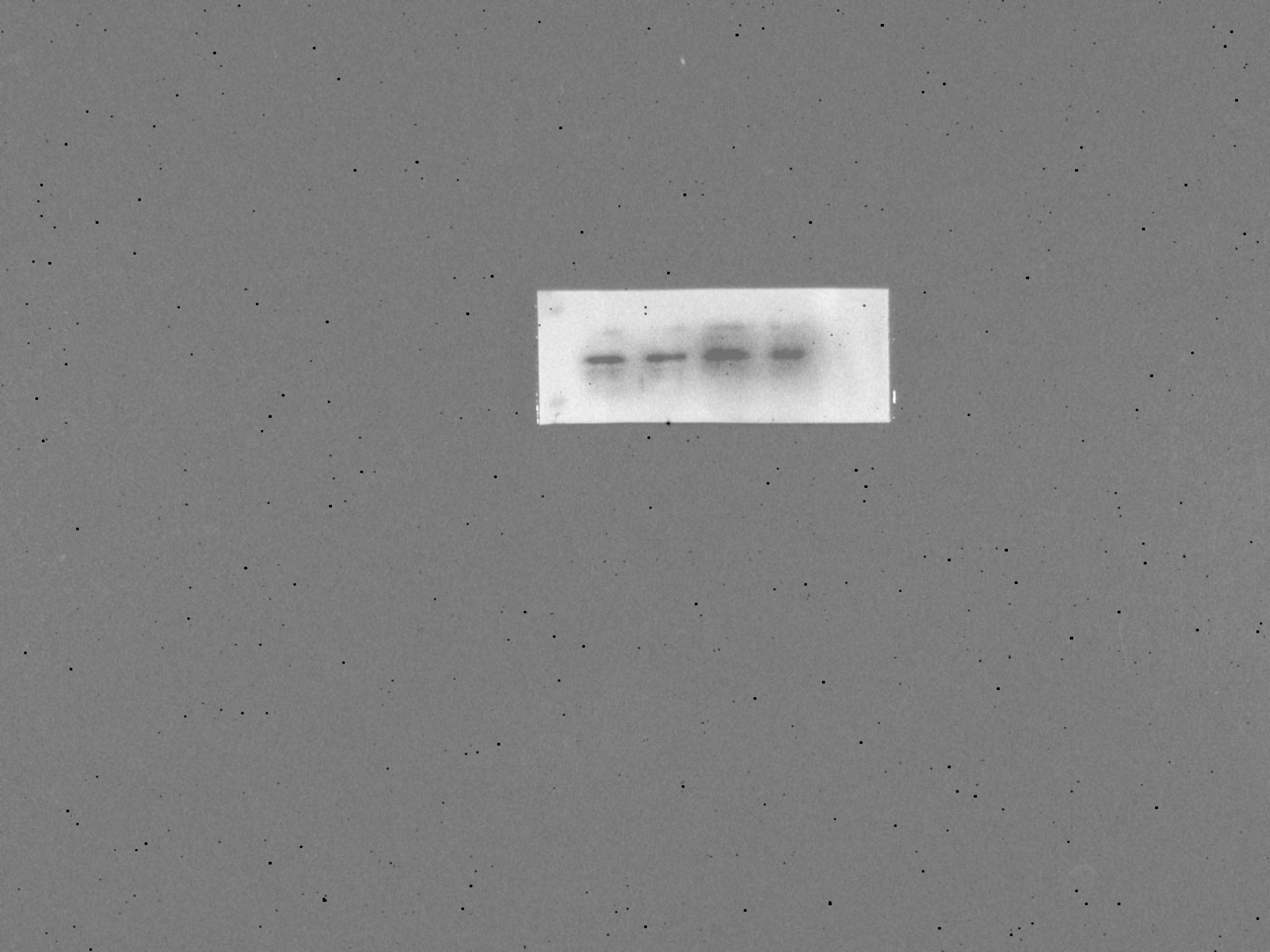

Supplement: Original Images for Blots.zip [file YRER_A_2313366_SM3875.zip › Original Images for Blots/Figure 5/Figure 5D/cle-caspase-3/Marker+cle-caspase-3.jpg]

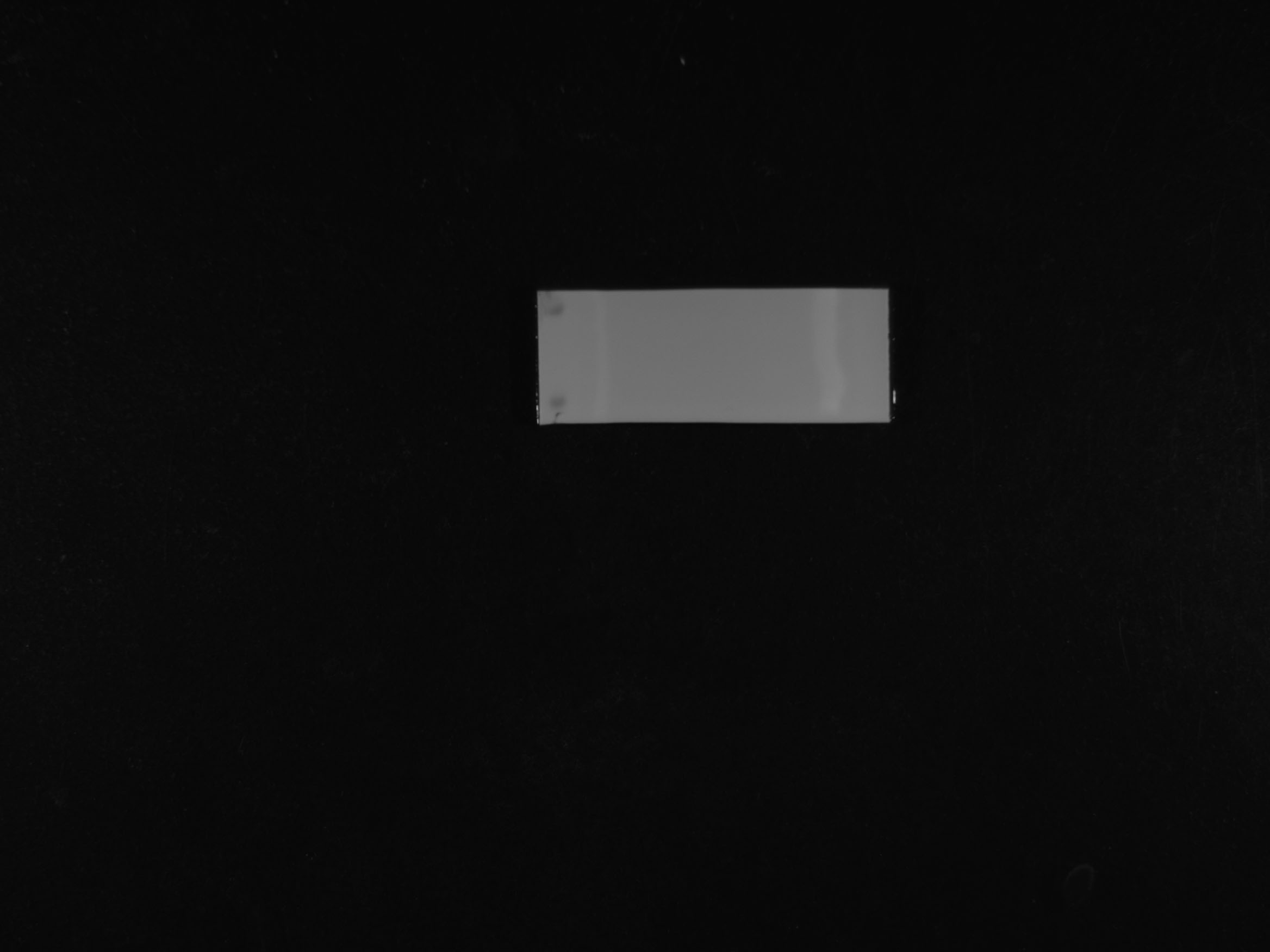

Supplement: Original Images for Blots.zip [file YRER_A_2313366_SM3875.zip › Original Images for Blots/Figure 5/Figure 5D/cle-caspase-3/Marker.jpg]

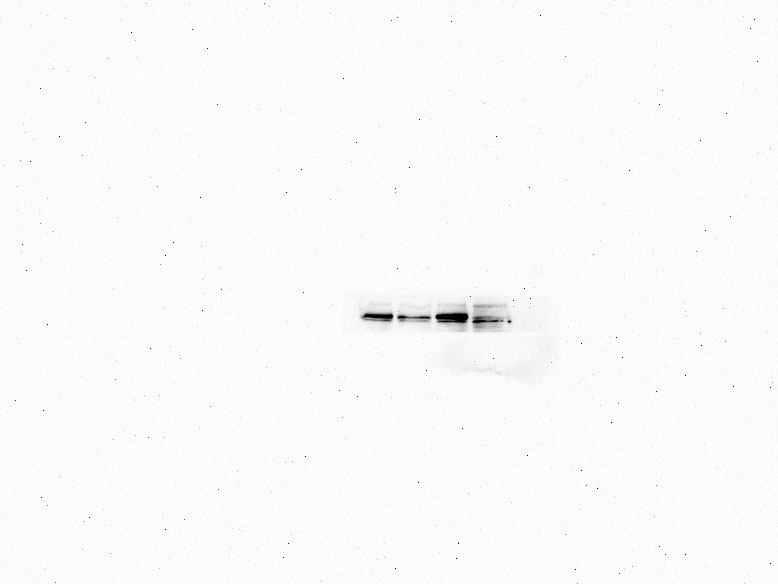

Supplement: Original Images for Blots.zip [file YRER_A_2313366_SM3875.zip › Original Images for Blots/Figure 5/Figure 5D/cle-PARP/cle-PARP.jpg]

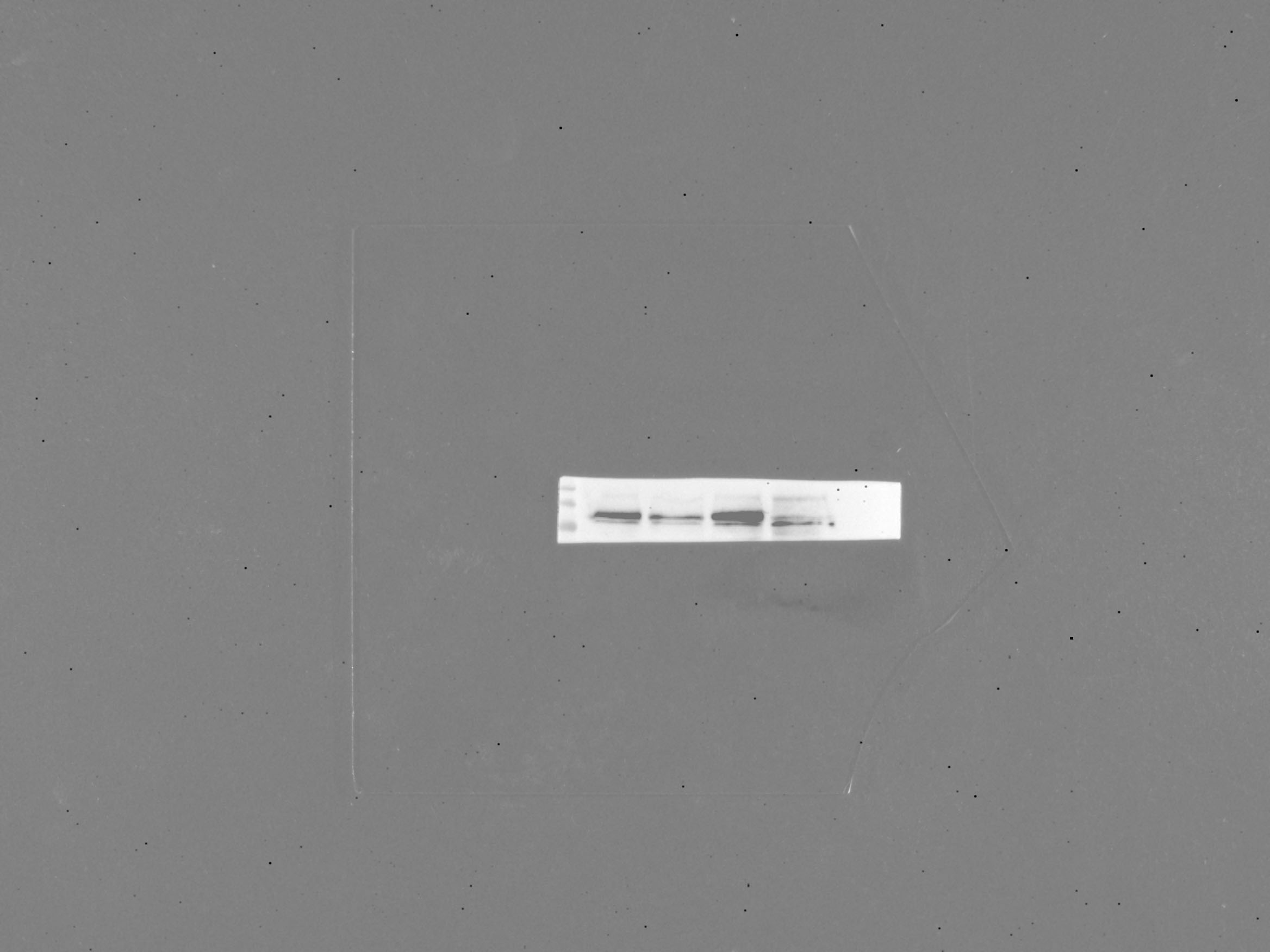

Supplement: Original Images for Blots.zip [file YRER_A_2313366_SM3875.zip › Original Images for Blots/Figure 5/Figure 5D/cle-PARP/Marker+cle-PARP.jpg]

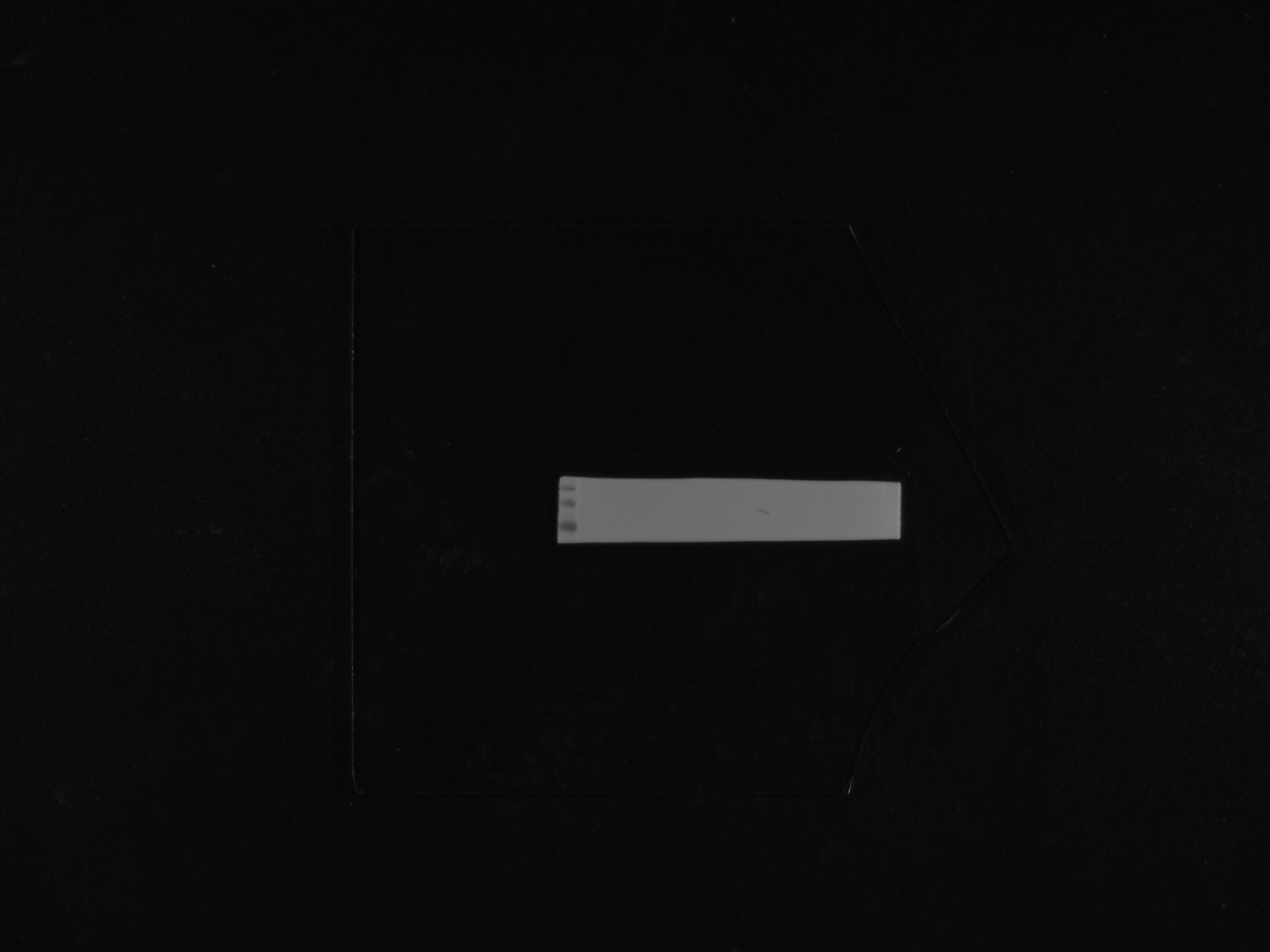

Supplement: Original Images for Blots.zip [file YRER_A_2313366_SM3875.zip › Original Images for Blots/Figure 5/Figure 5D/cle-PARP/Marker.jpg]

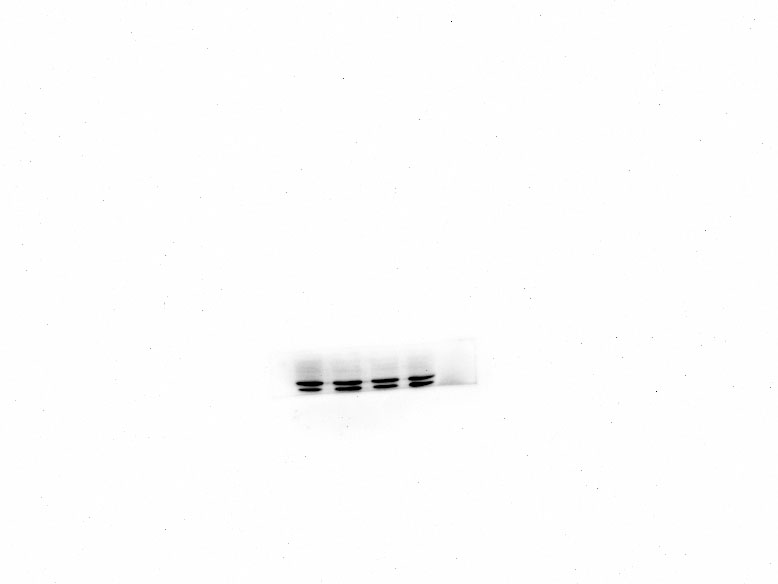

Supplement: Original Images for Blots.zip [file YRER_A_2313366_SM3875.zip › Original Images for Blots/Figure 5/Figure 5D/ERK/ERK.jpg]

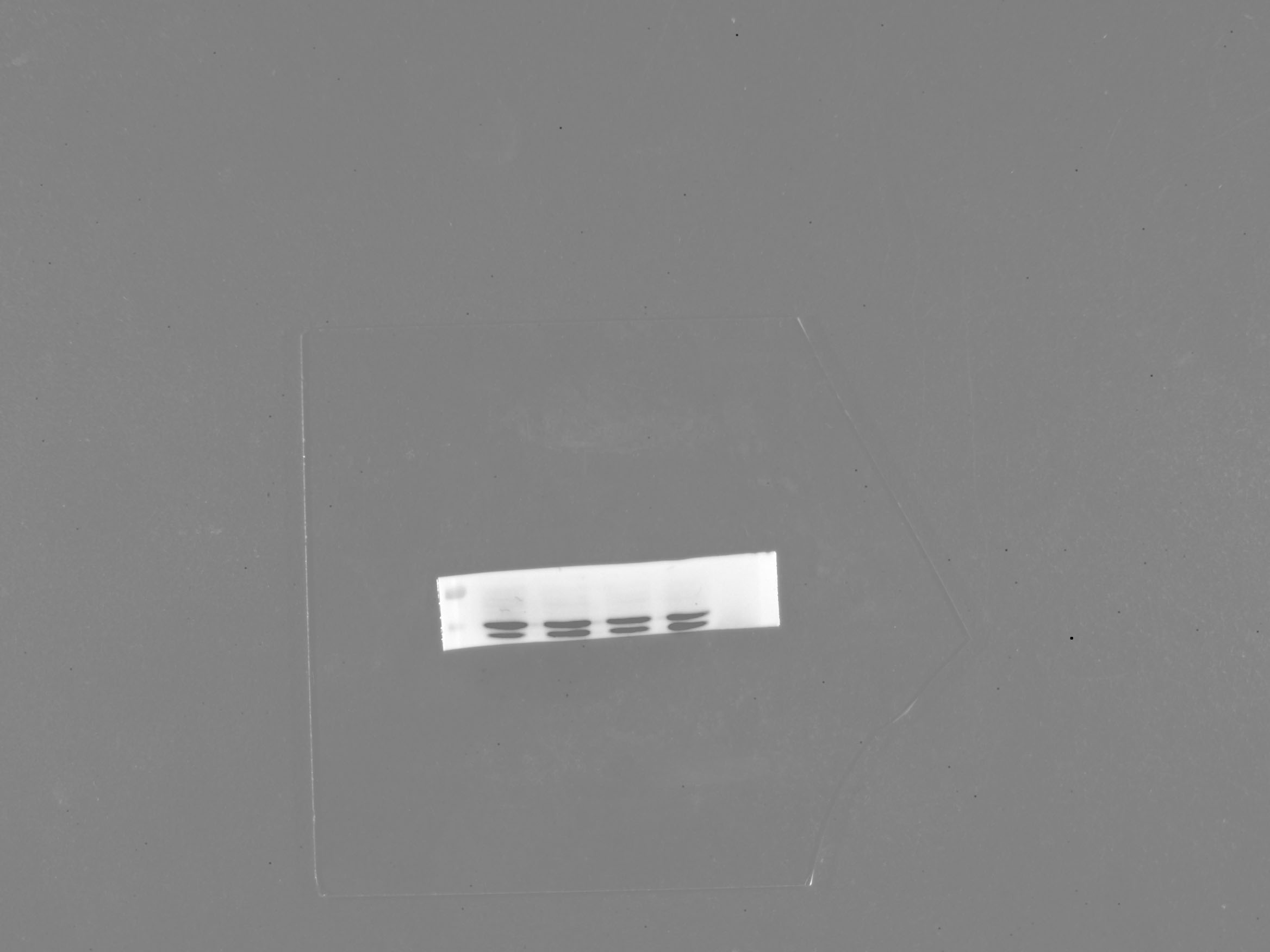

Supplement: Original Images for Blots.zip [file YRER_A_2313366_SM3875.zip › Original Images for Blots/Figure 5/Figure 5D/ERK/Marker+ERK.jpg]

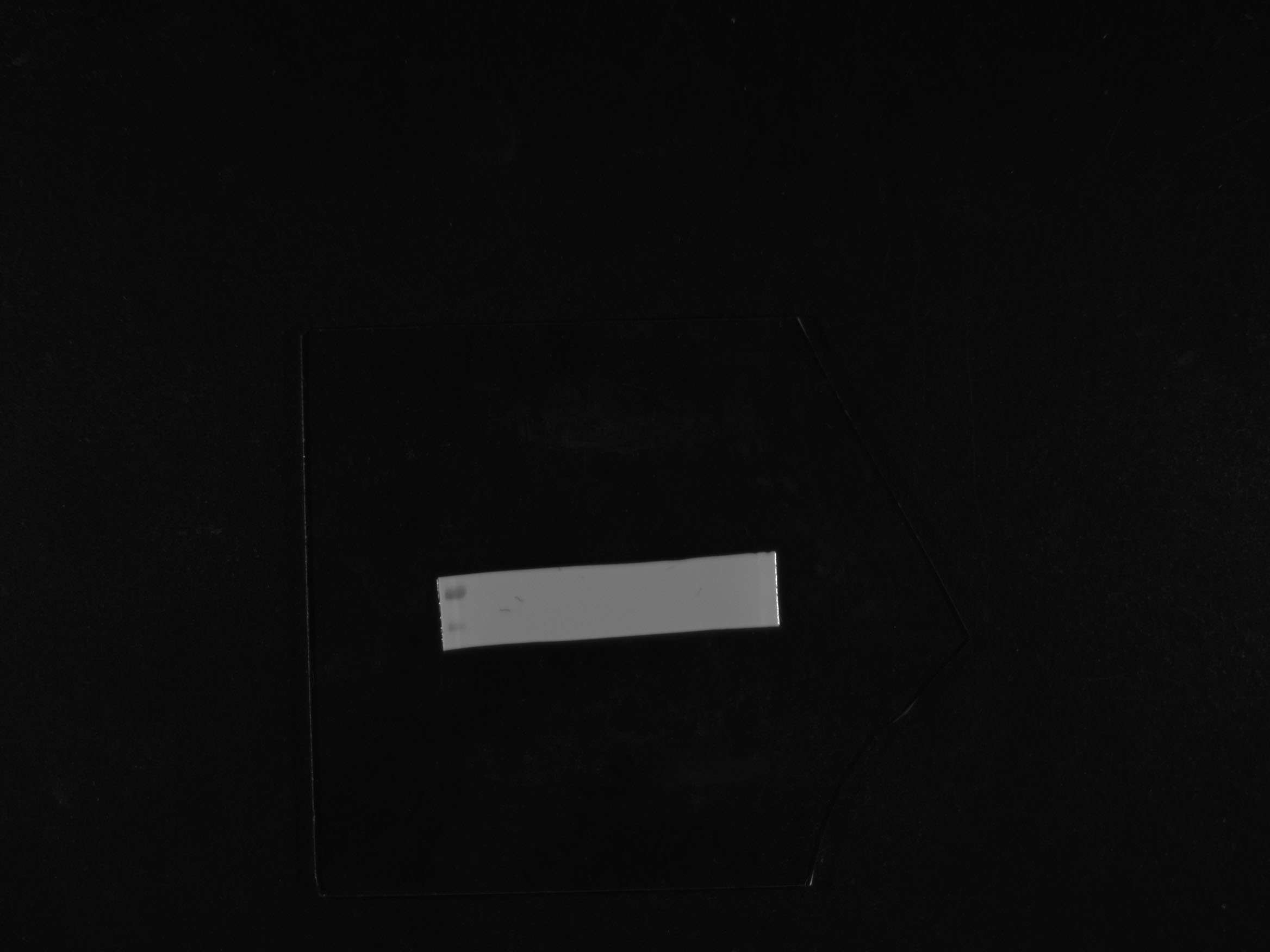

Supplement: Original Images for Blots.zip [file YRER_A_2313366_SM3875.zip › Original Images for Blots/Figure 5/Figure 5D/ERK/Marker.jpg]

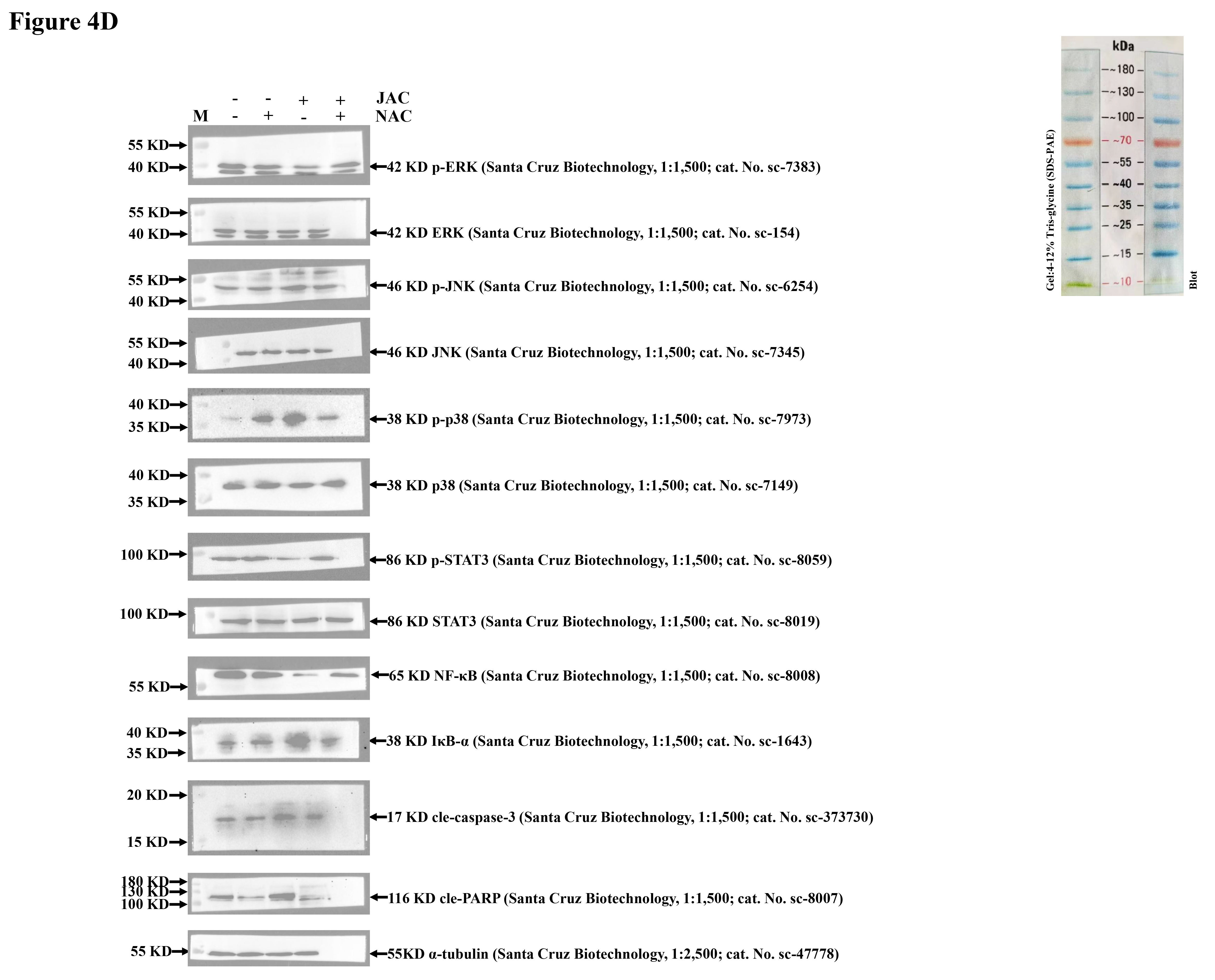

Supplement: Original Images for Blots.zip [file YRER_A_2313366_SM3875.zip › Original Images for Blots/Figure 5/Figure 5D/Figure 5D.jpg]

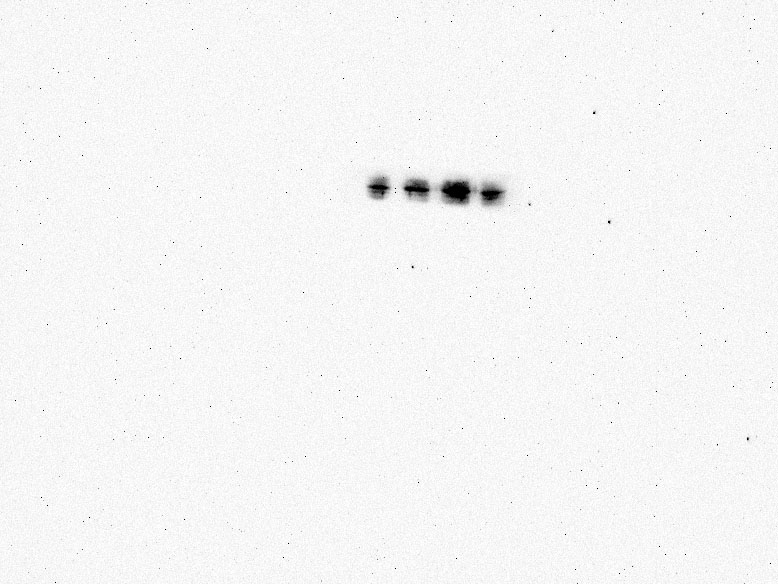

Supplement: Original Images for Blots.zip [file YRER_A_2313366_SM3875.zip › Original Images for Blots/Figure 5/Figure 5D/IκB-α/IκB-α.jpg]

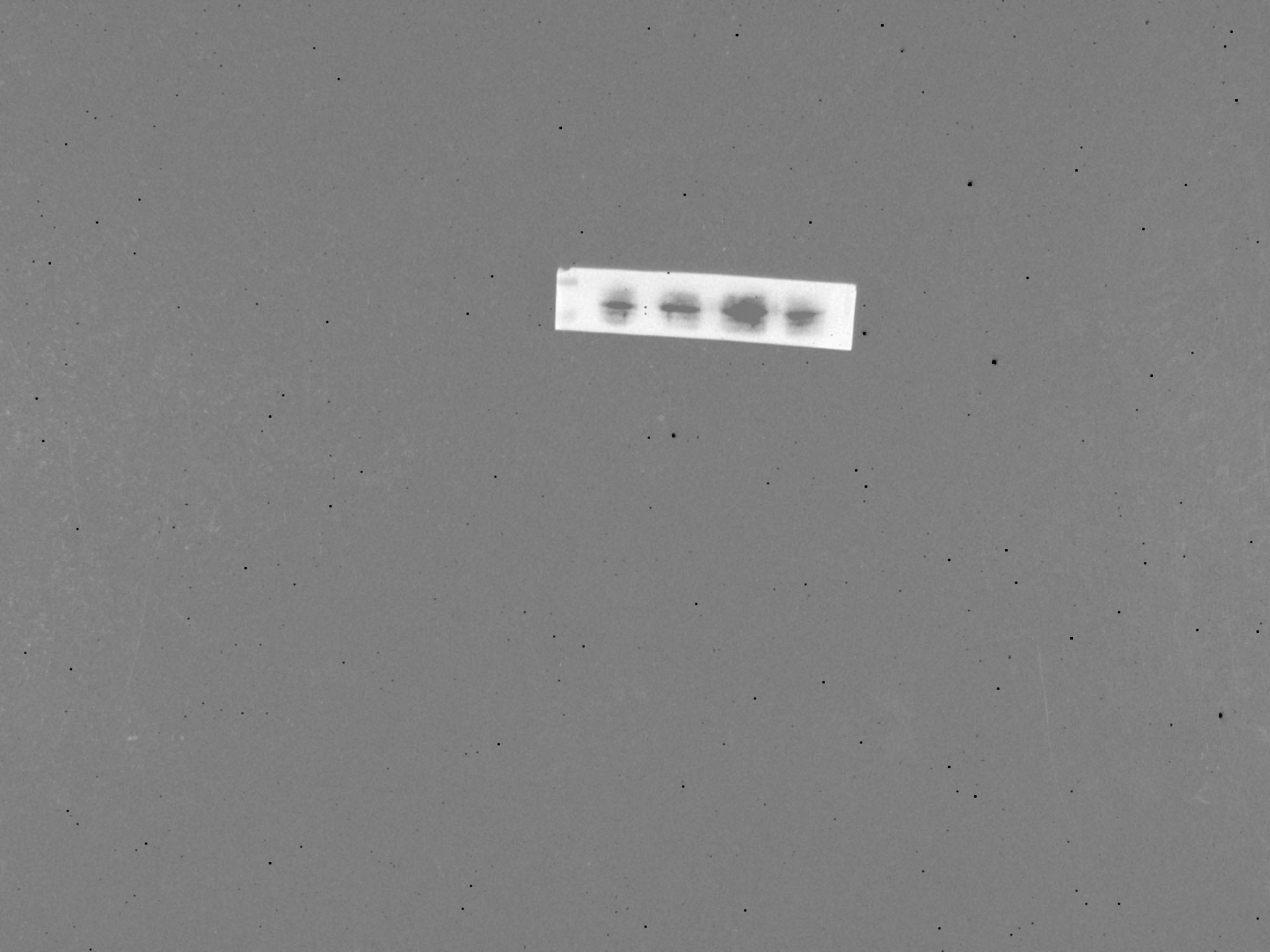

Supplement: Original Images for Blots.zip [file YRER_A_2313366_SM3875.zip › Original Images for Blots/Figure 5/Figure 5D/IκB-α/Marker+IκB-α.jpg]

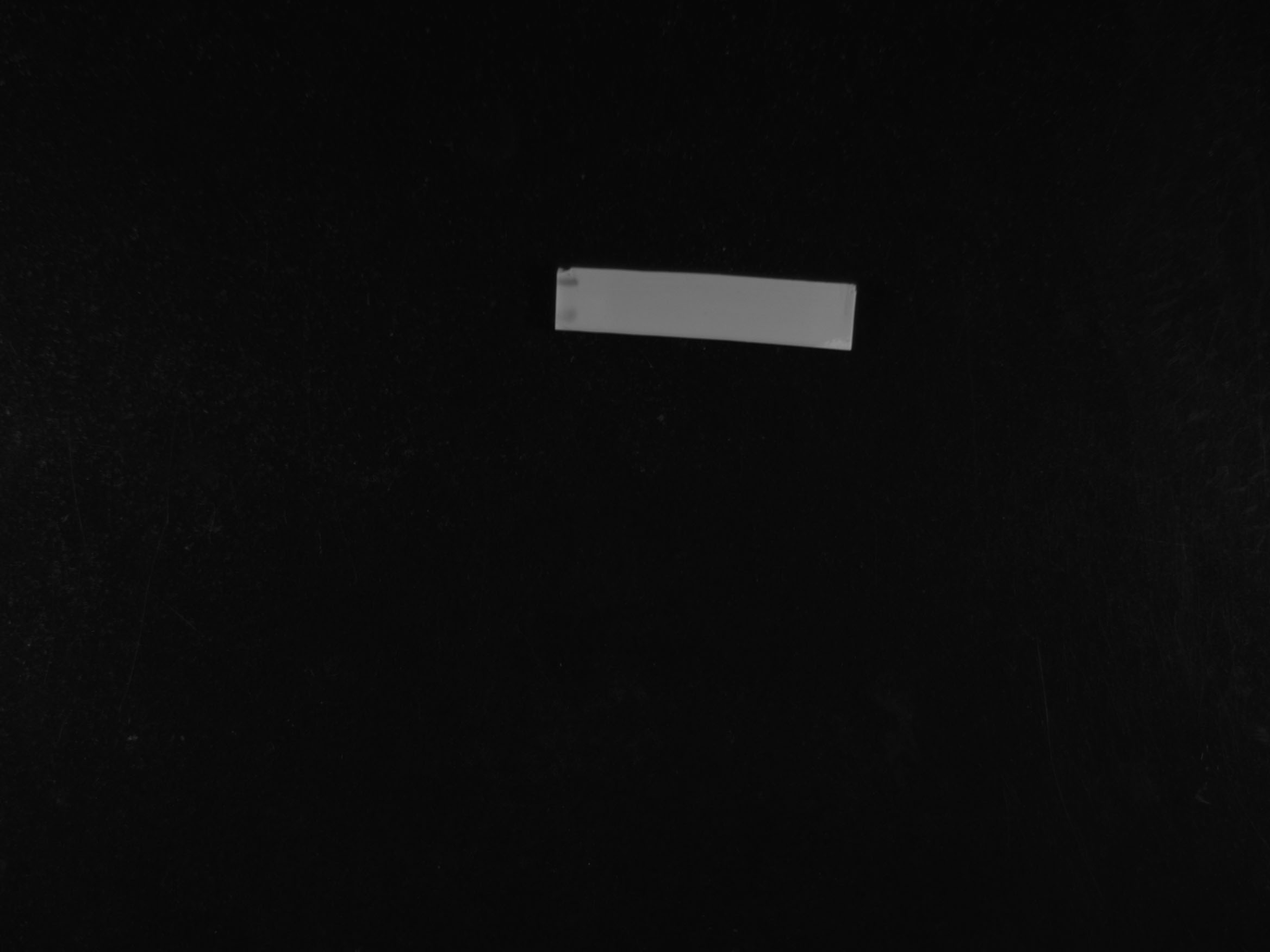

Supplement: Original Images for Blots.zip [file YRER_A_2313366_SM3875.zip › Original Images for Blots/Figure 5/Figure 5D/IκB-α/Marker.jpg]

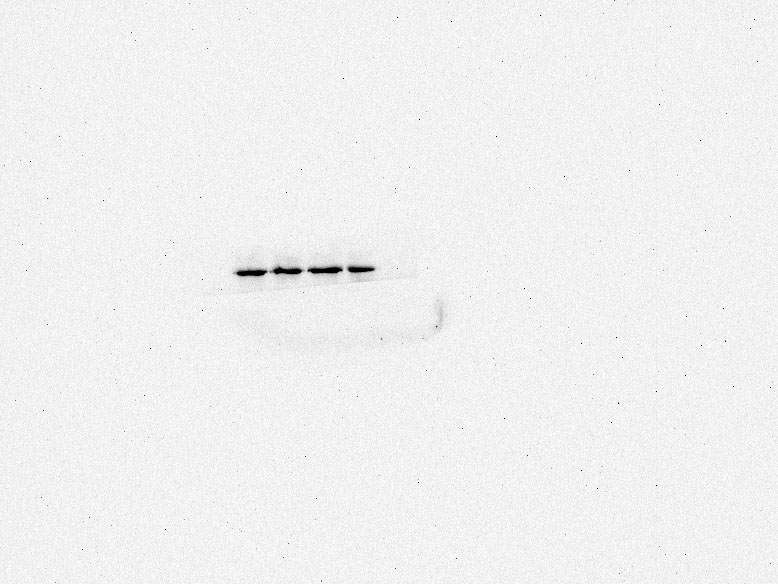

Supplement: Original Images for Blots.zip [file YRER_A_2313366_SM3875.zip › Original Images for Blots/Figure 5/Figure 5D/JNK/JNK.jpg]

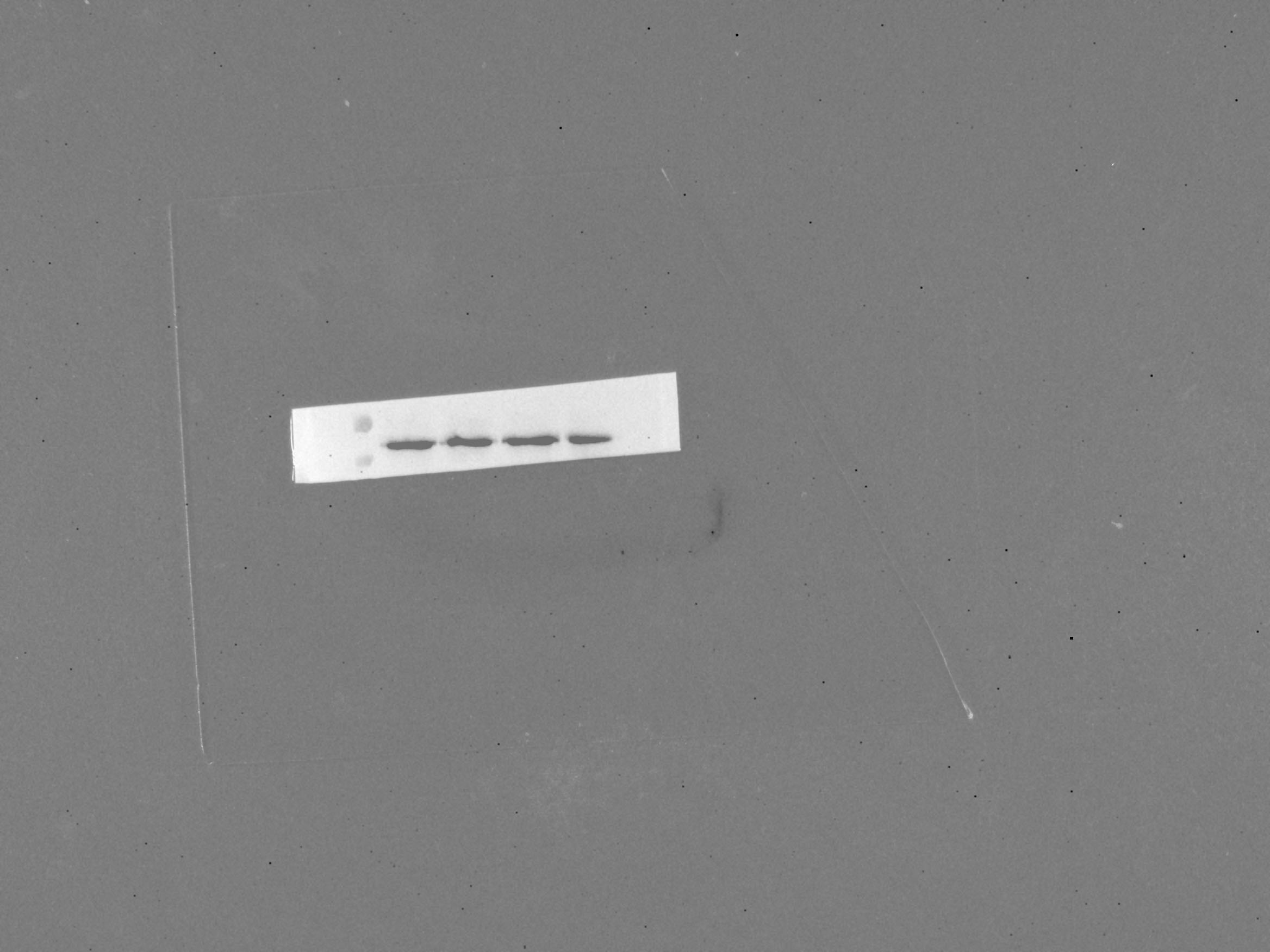

Supplement: Original Images for Blots.zip [file YRER_A_2313366_SM3875.zip › Original Images for Blots/Figure 5/Figure 5D/JNK/Marker+JNK.jpg]

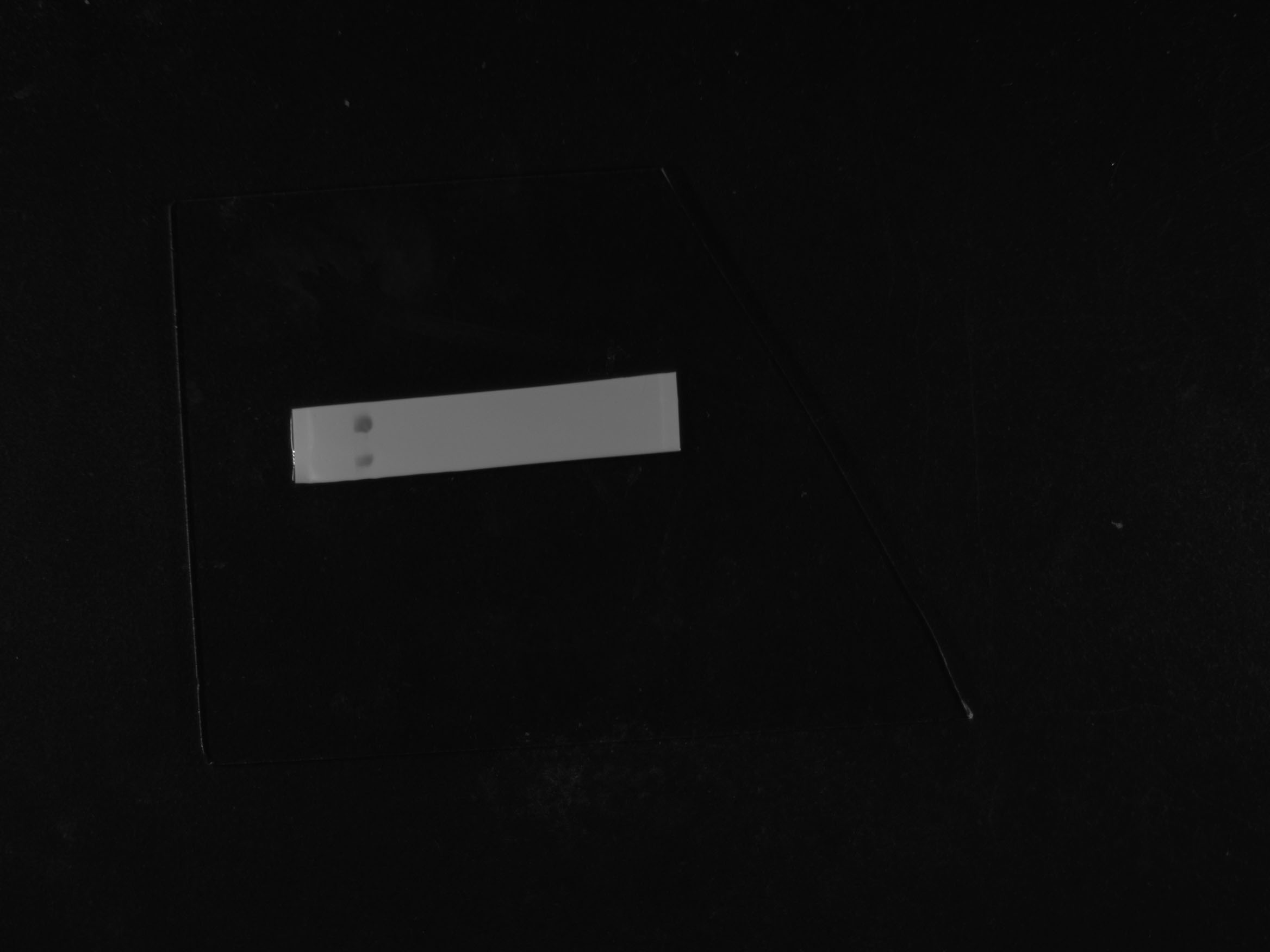

Supplement: Original Images for Blots.zip [file YRER_A_2313366_SM3875.zip › Original Images for Blots/Figure 5/Figure 5D/JNK/Marker.jpg]

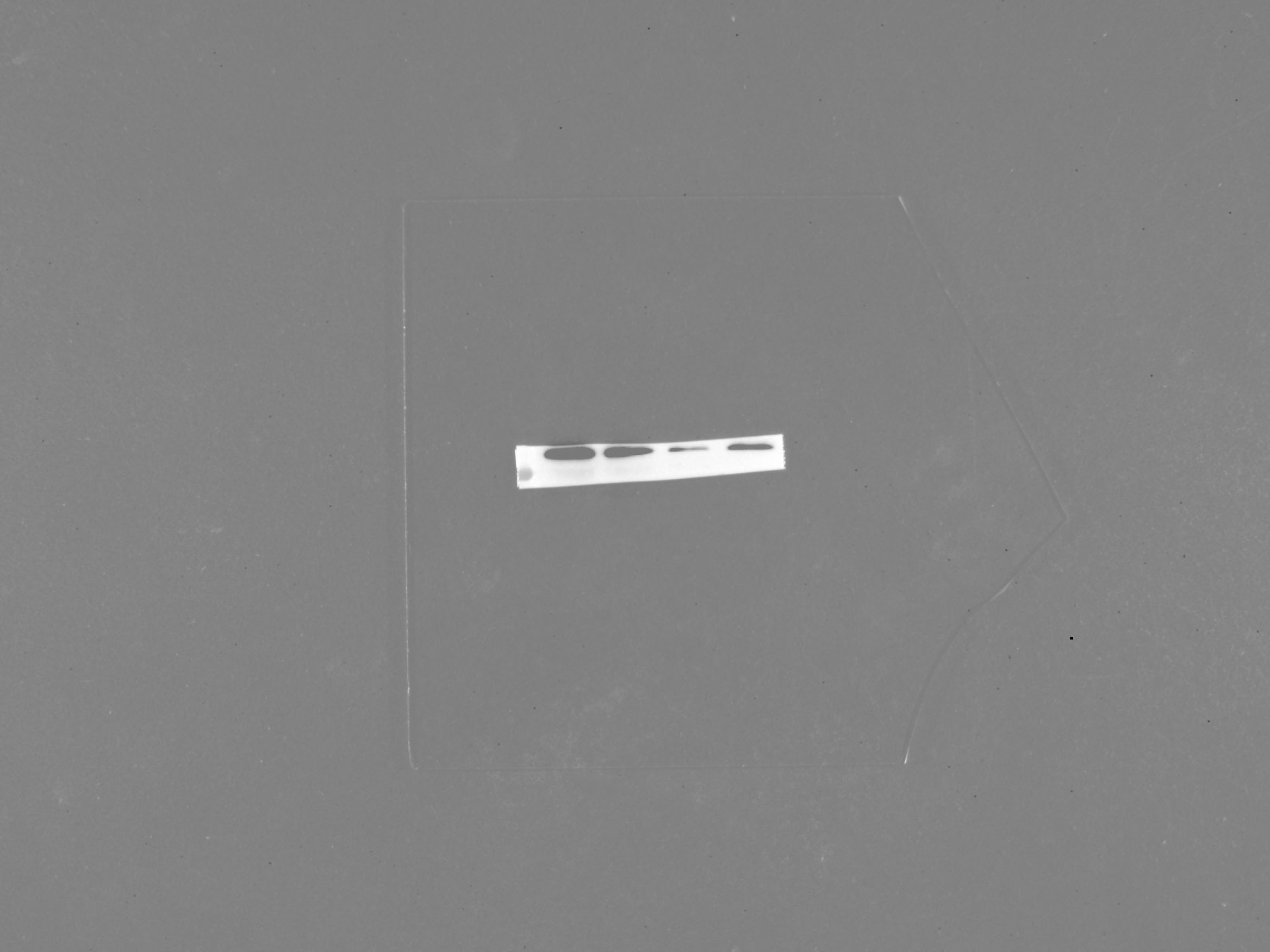

Supplement: Original Images for Blots.zip [file YRER_A_2313366_SM3875.zip › Original Images for Blots/Figure 5/Figure 5D/NF-κB/Marker+NF-κB.jpg]

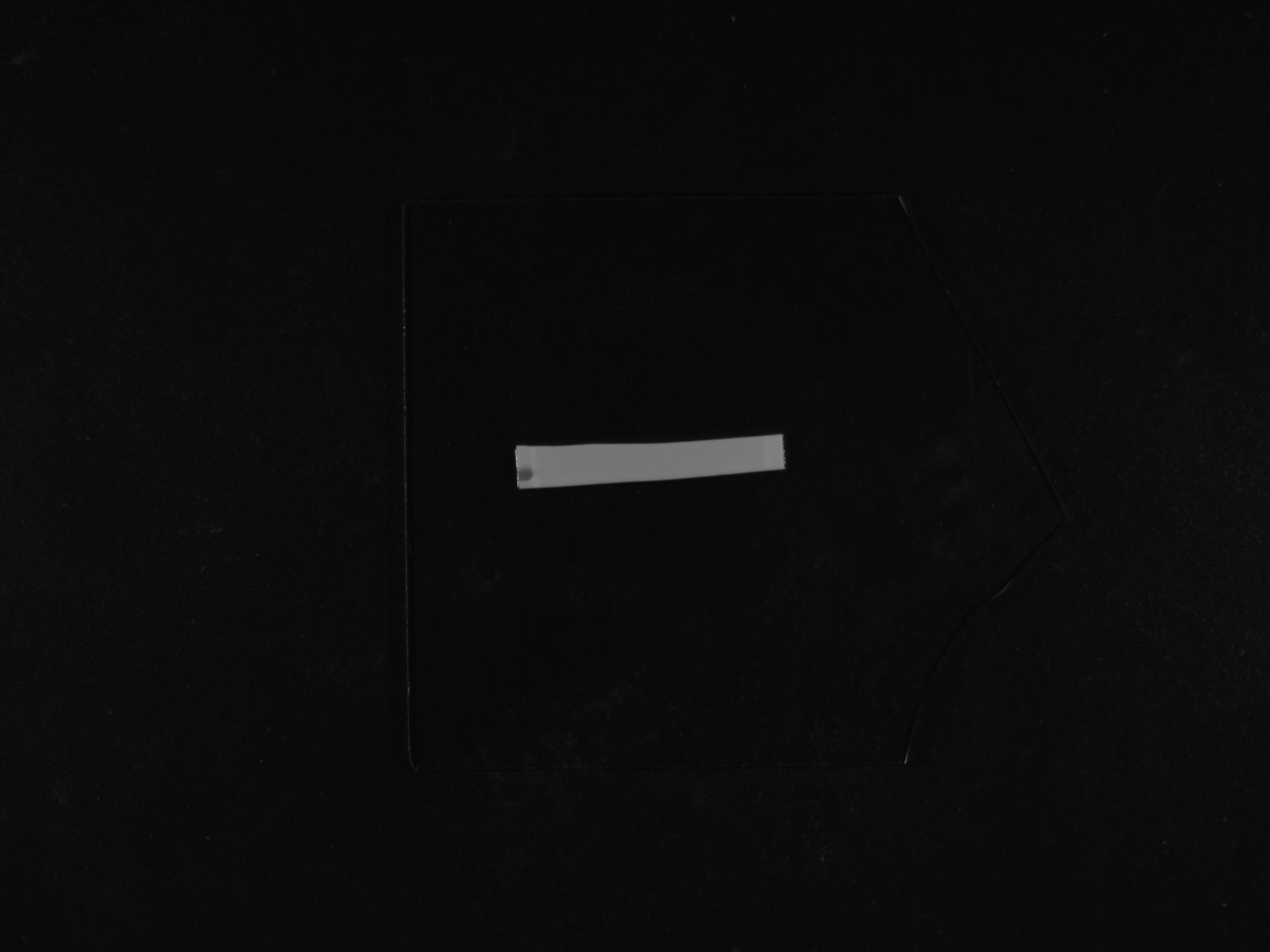

Supplement: Original Images for Blots.zip [file YRER_A_2313366_SM3875.zip › Original Images for Blots/Figure 5/Figure 5D/NF-κB/Marker.jpg]

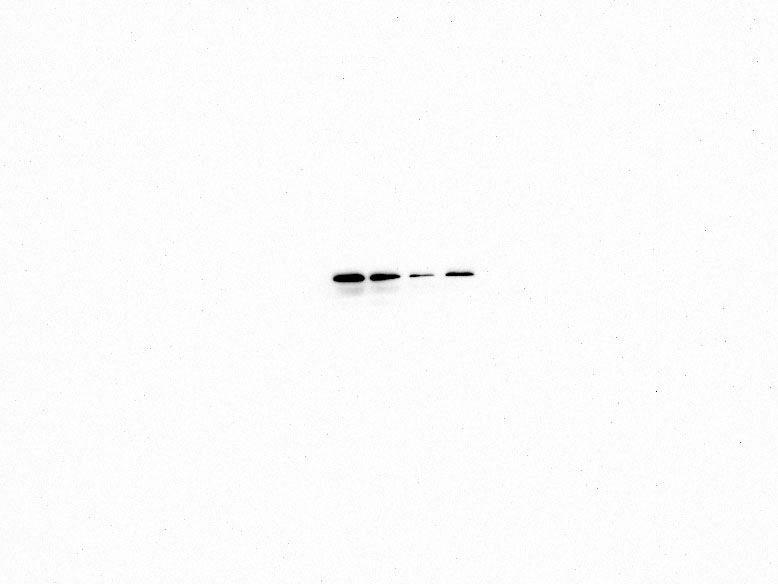

Supplement: Original Images for Blots.zip [file YRER_A_2313366_SM3875.zip › Original Images for Blots/Figure 5/Figure 5D/NF-κB/NF-κB.jpg]

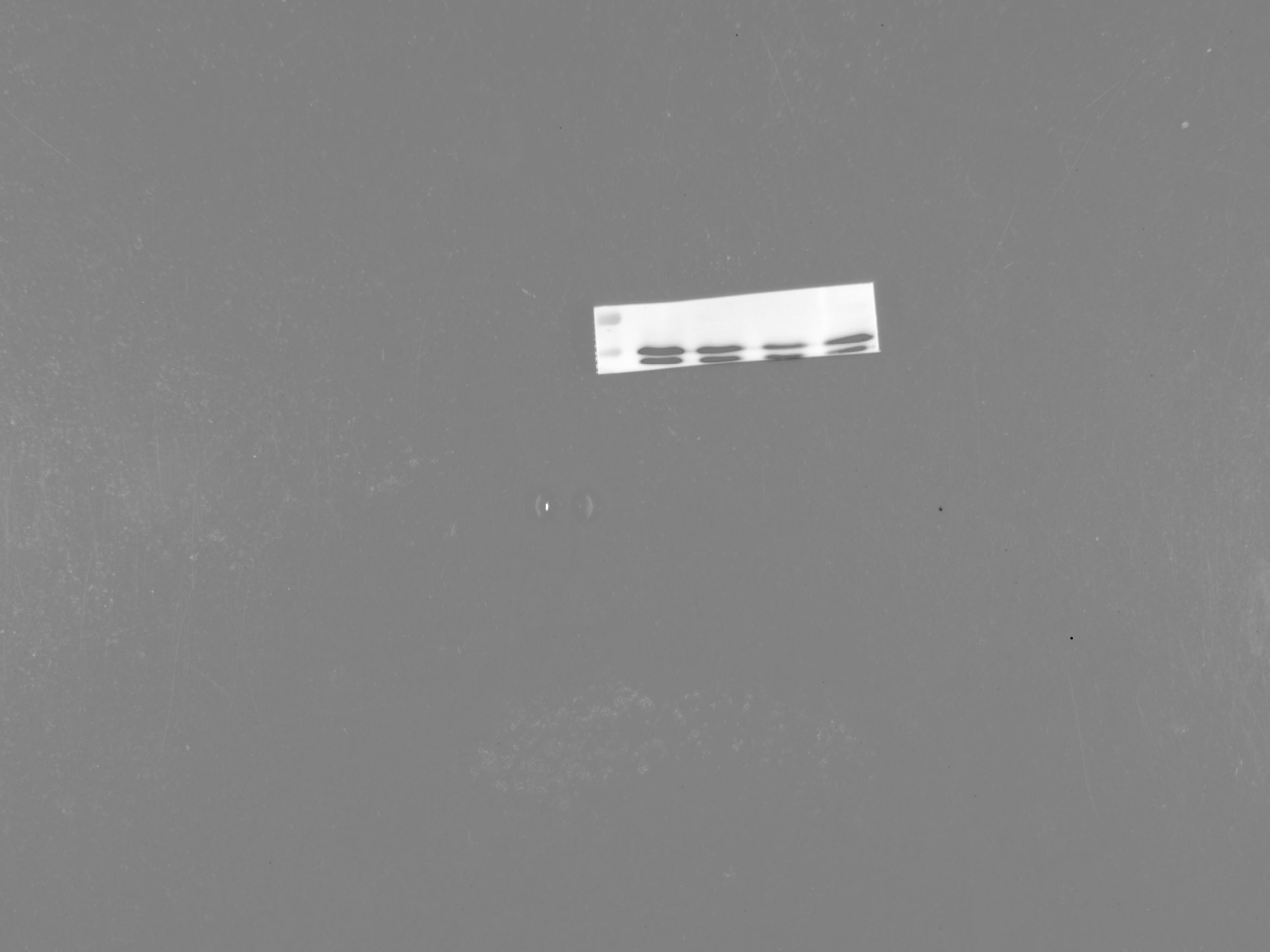

Supplement: Original Images for Blots.zip [file YRER_A_2313366_SM3875.zip › Original Images for Blots/Figure 5/Figure 5D/p-ERK/Marker+p-ERK.jpg]

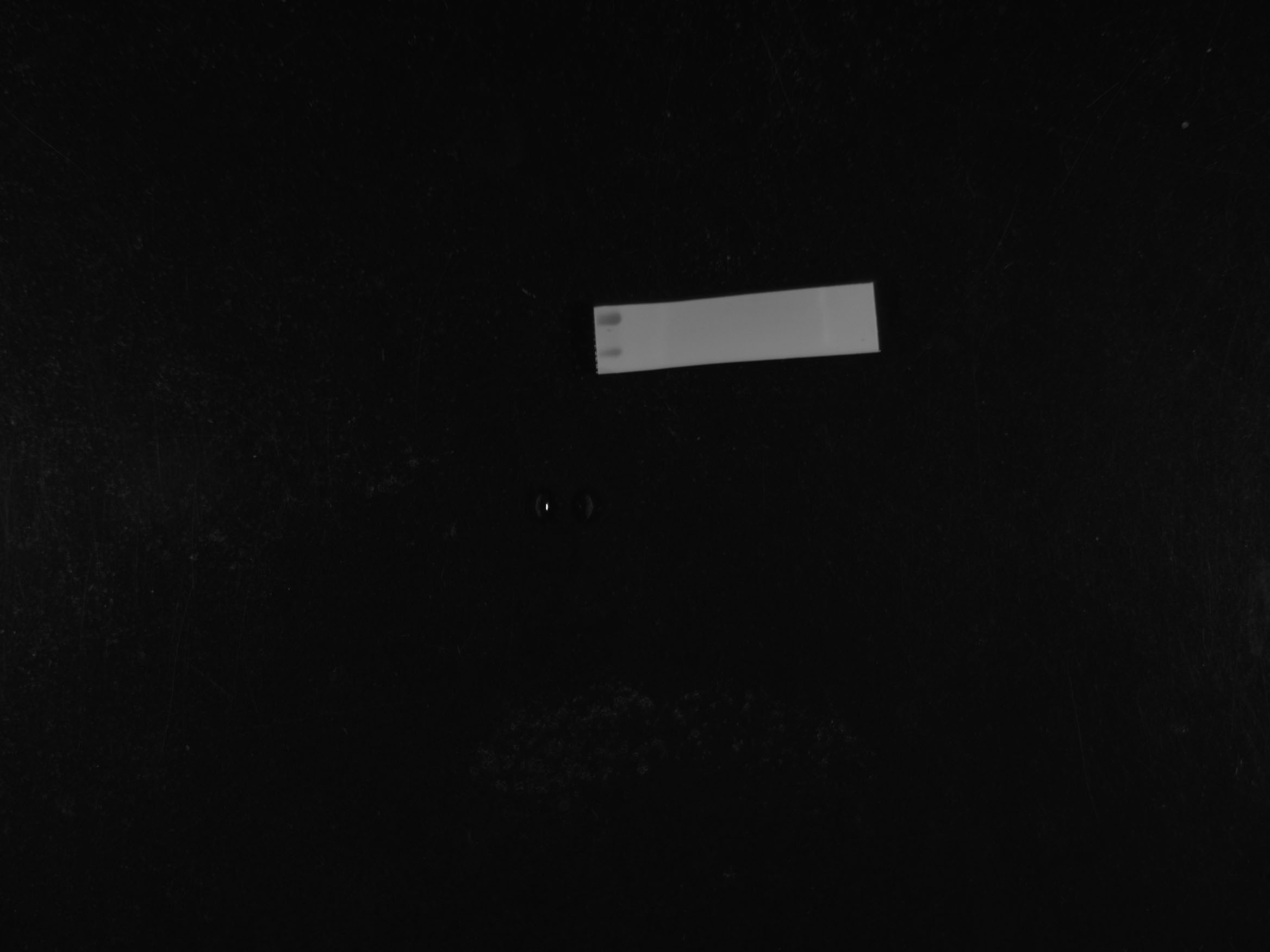

Supplement: Original Images for Blots.zip [file YRER_A_2313366_SM3875.zip › Original Images for Blots/Figure 5/Figure 5D/p-ERK/Marker.jpg]

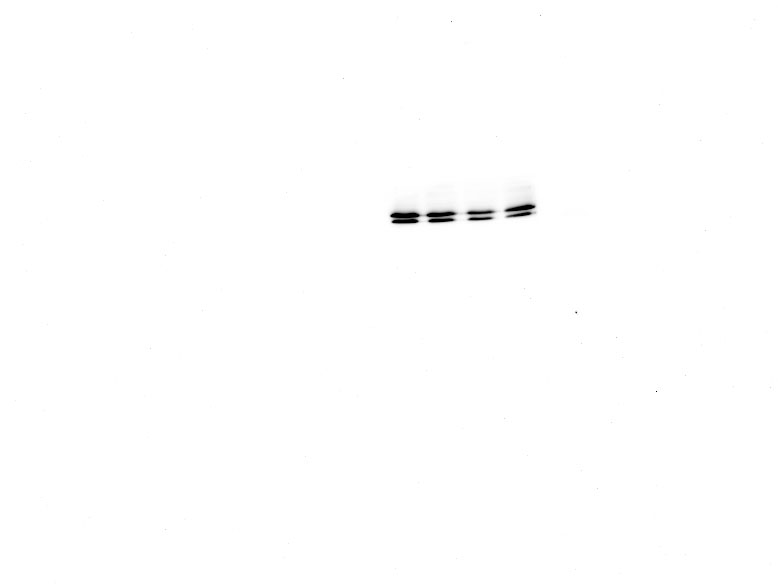

Supplement: Original Images for Blots.zip [file YRER_A_2313366_SM3875.zip › Original Images for Blots/Figure 5/Figure 5D/p-ERK/p-ERK.jpg]

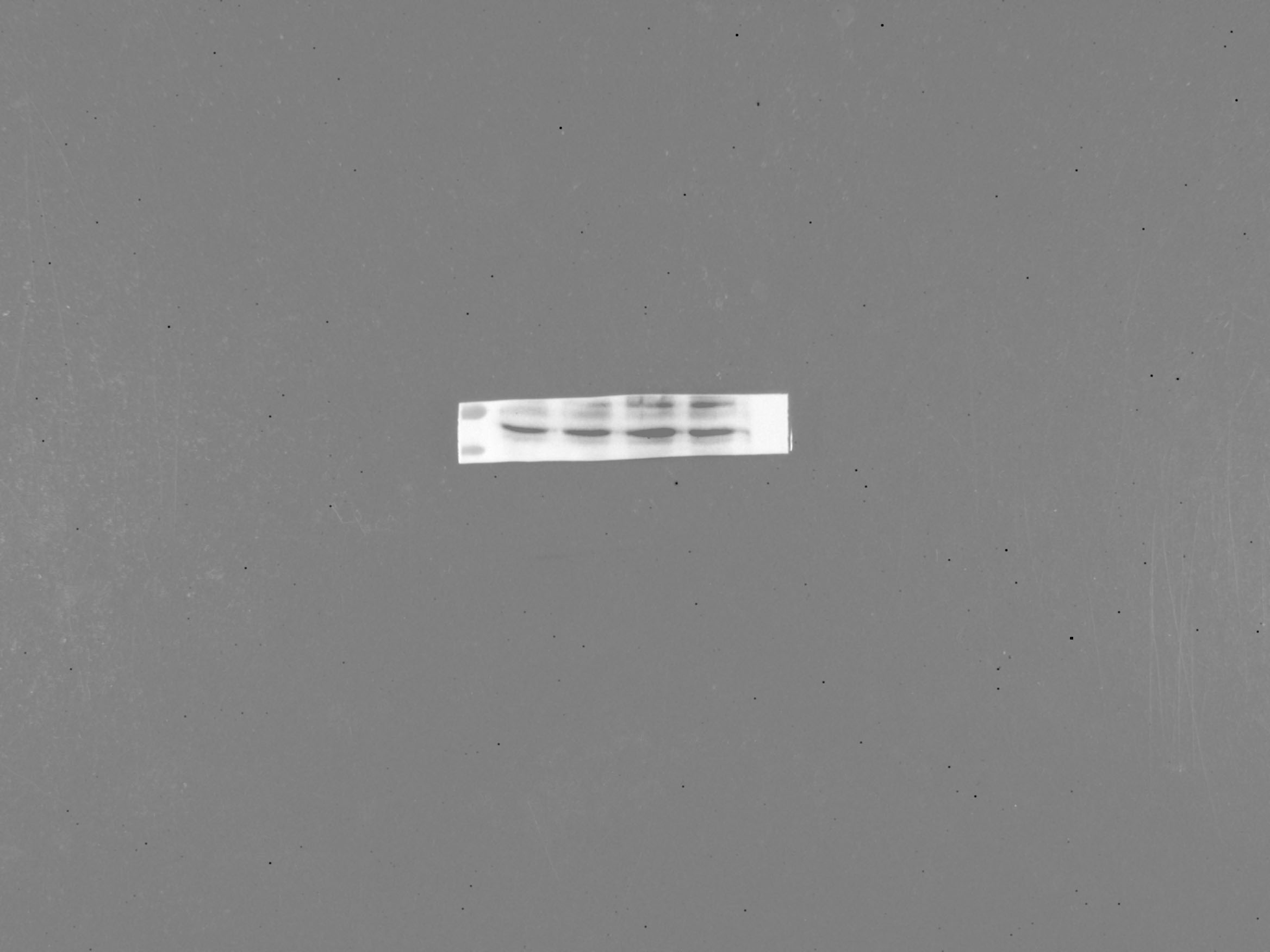

Supplement: Original Images for Blots.zip [file YRER_A_2313366_SM3875.zip › Original Images for Blots/Figure 5/Figure 5D/p-JNK/Marker+p-JNK.jpg]

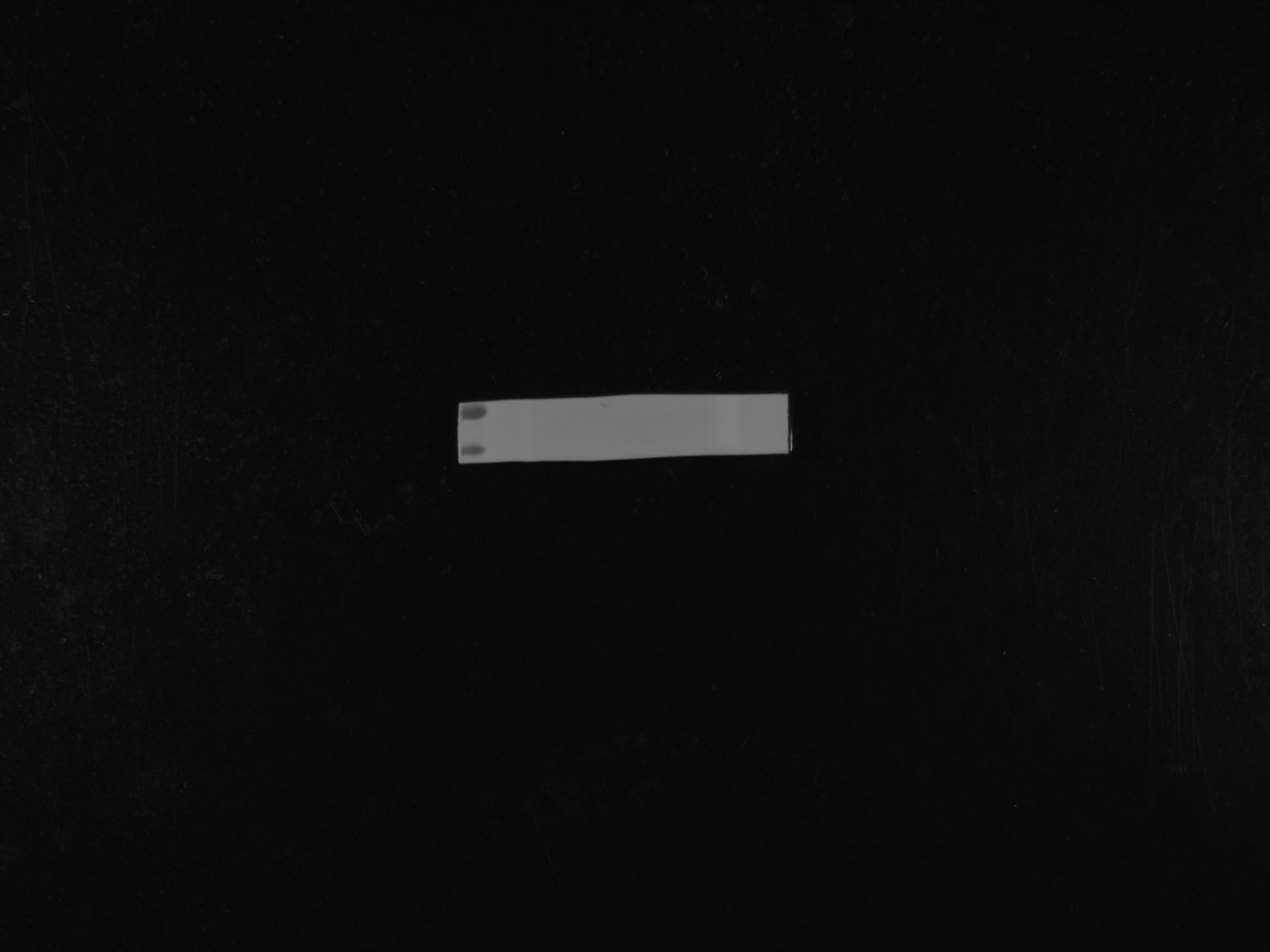

Supplement: Original Images for Blots.zip [file YRER_A_2313366_SM3875.zip › Original Images for Blots/Figure 5/Figure 5D/p-JNK/Marker.jpg]

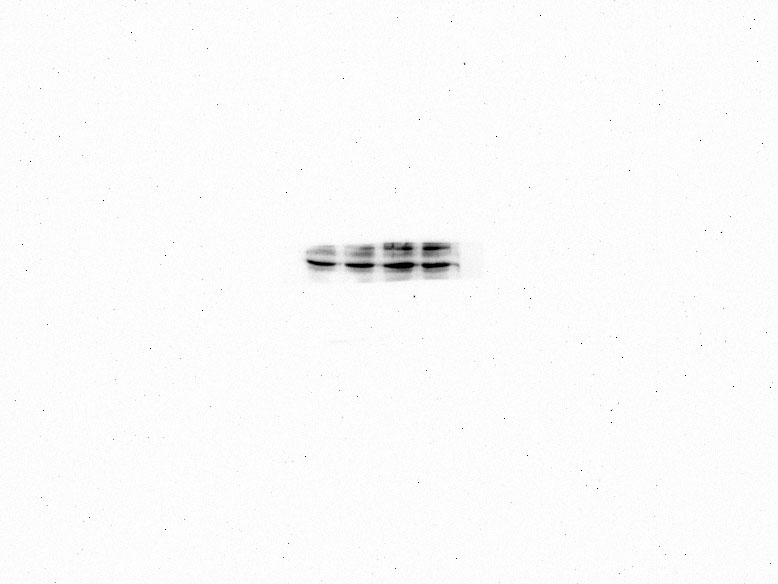

Supplement: Original Images for Blots.zip [file YRER_A_2313366_SM3875.zip › Original Images for Blots/Figure 5/Figure 5D/p-JNK/p-JNK.jpg]

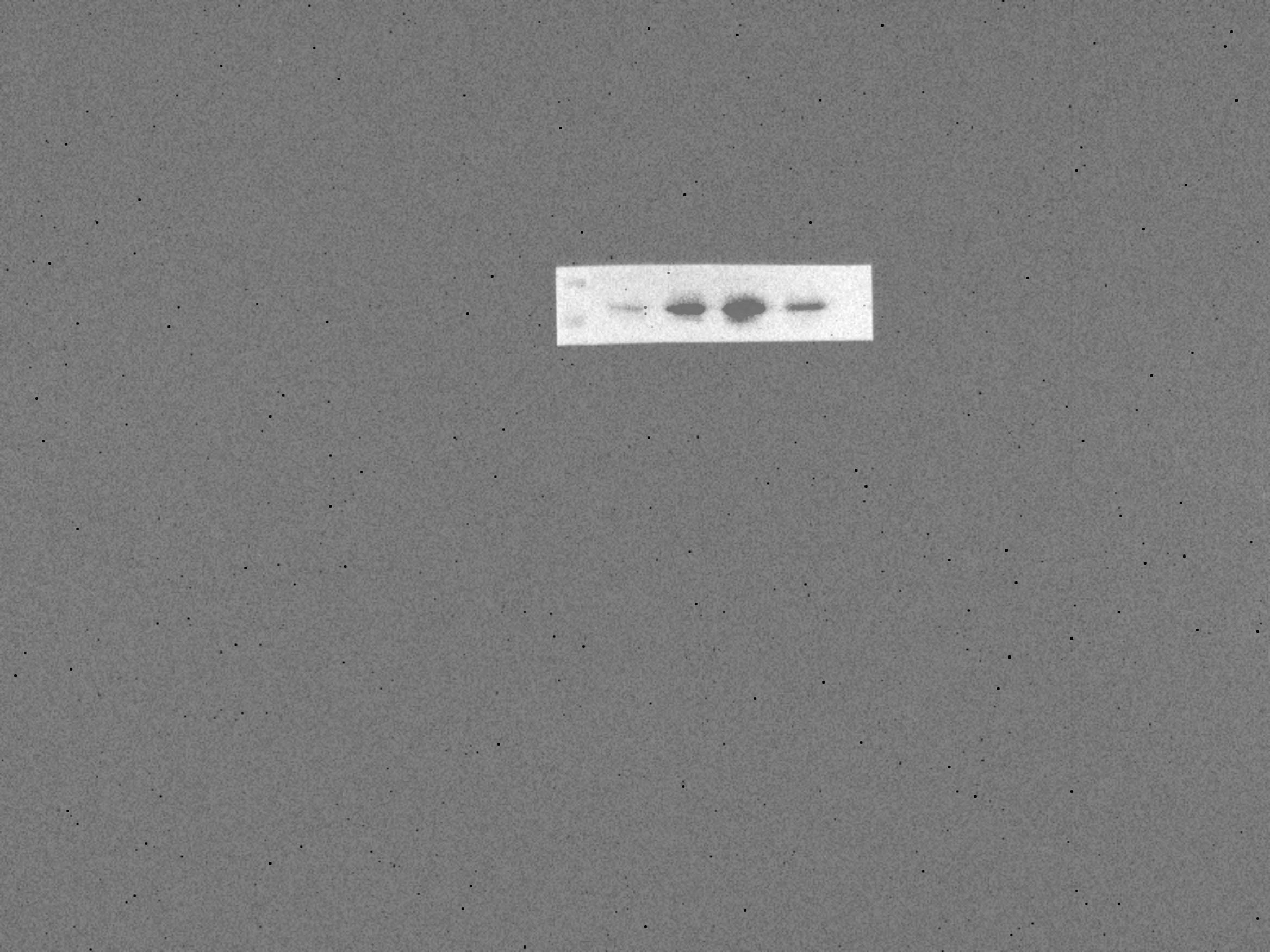

Supplement: Original Images for Blots.zip [file YRER_A_2313366_SM3875.zip › Original Images for Blots/Figure 5/Figure 5D/p-p38/Marker+p-p38.jpg]

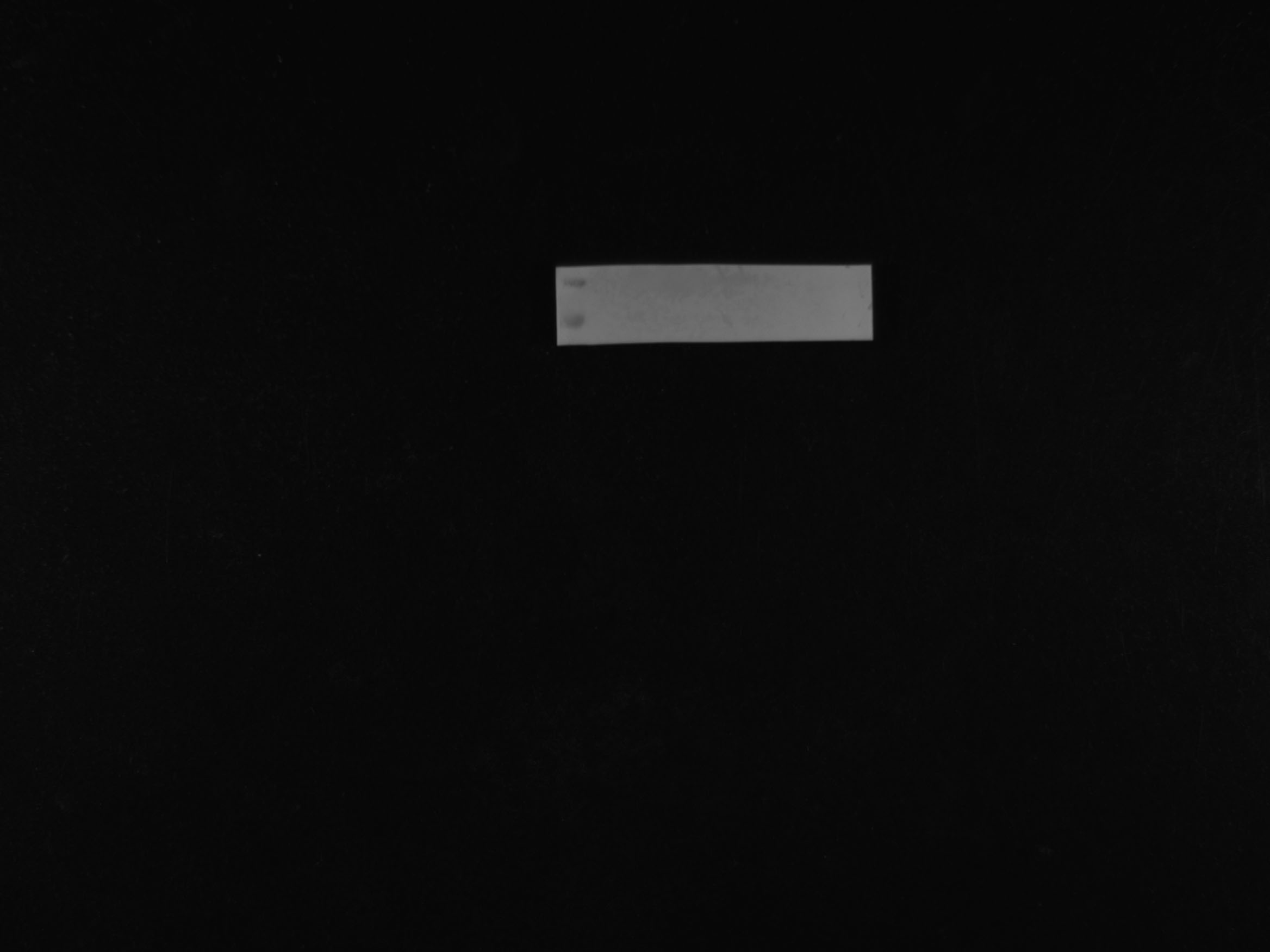

Supplement: Original Images for Blots.zip [file YRER_A_2313366_SM3875.zip › Original Images for Blots/Figure 5/Figure 5D/p-p38/Marker.jpg]

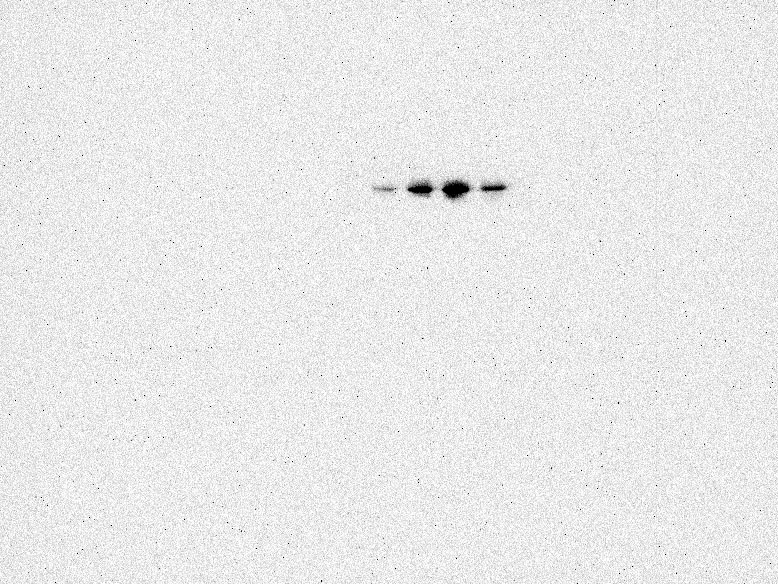

Supplement: Original Images for Blots.zip [file YRER_A_2313366_SM3875.zip › Original Images for Blots/Figure 5/Figure 5D/p-p38/p-p38.jpg]

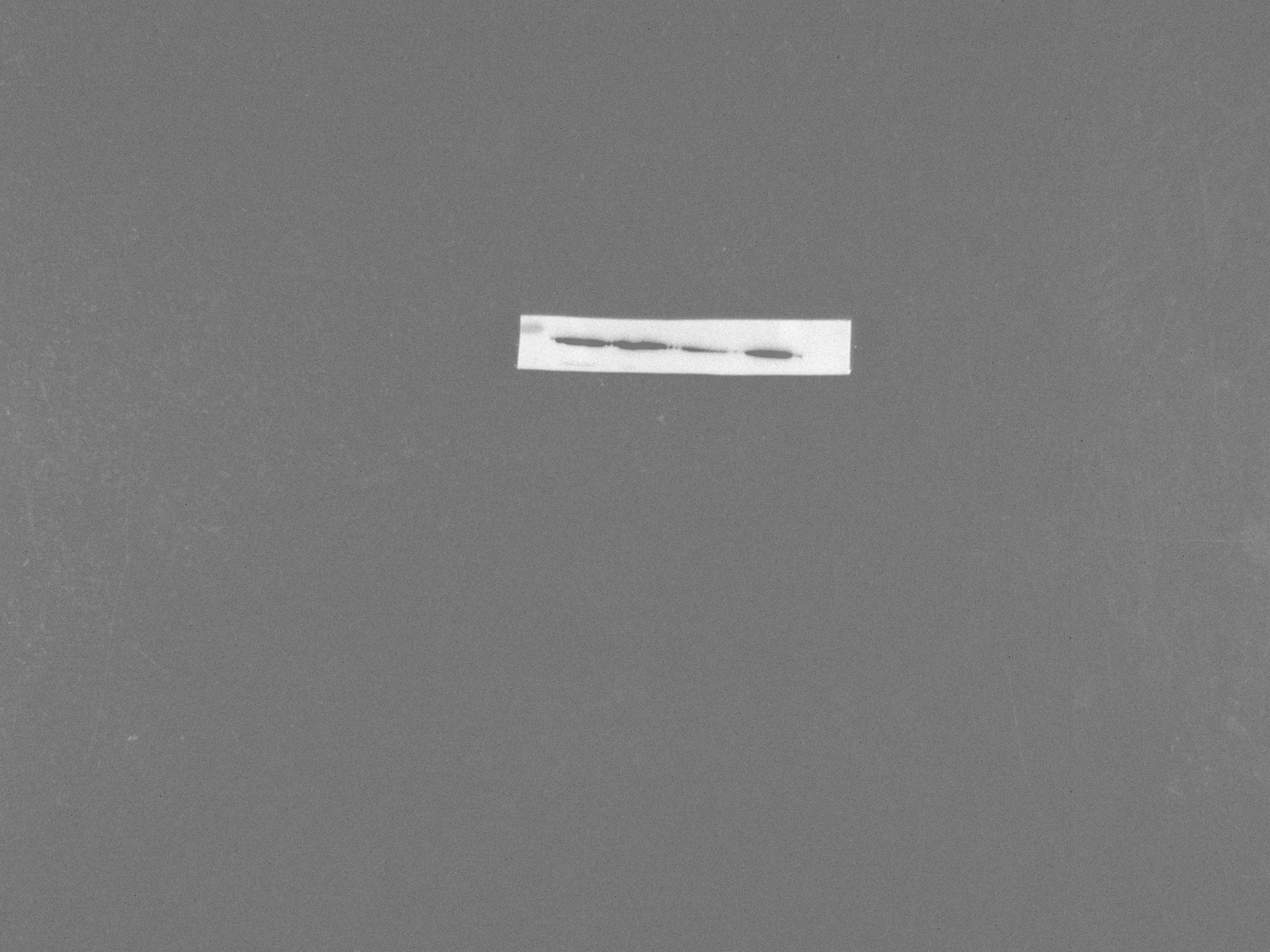

Supplement: Original Images for Blots.zip [file YRER_A_2313366_SM3875.zip › Original Images for Blots/Figure 5/Figure 5D/p-STAT3/Marker+p-STAT3.jpg]

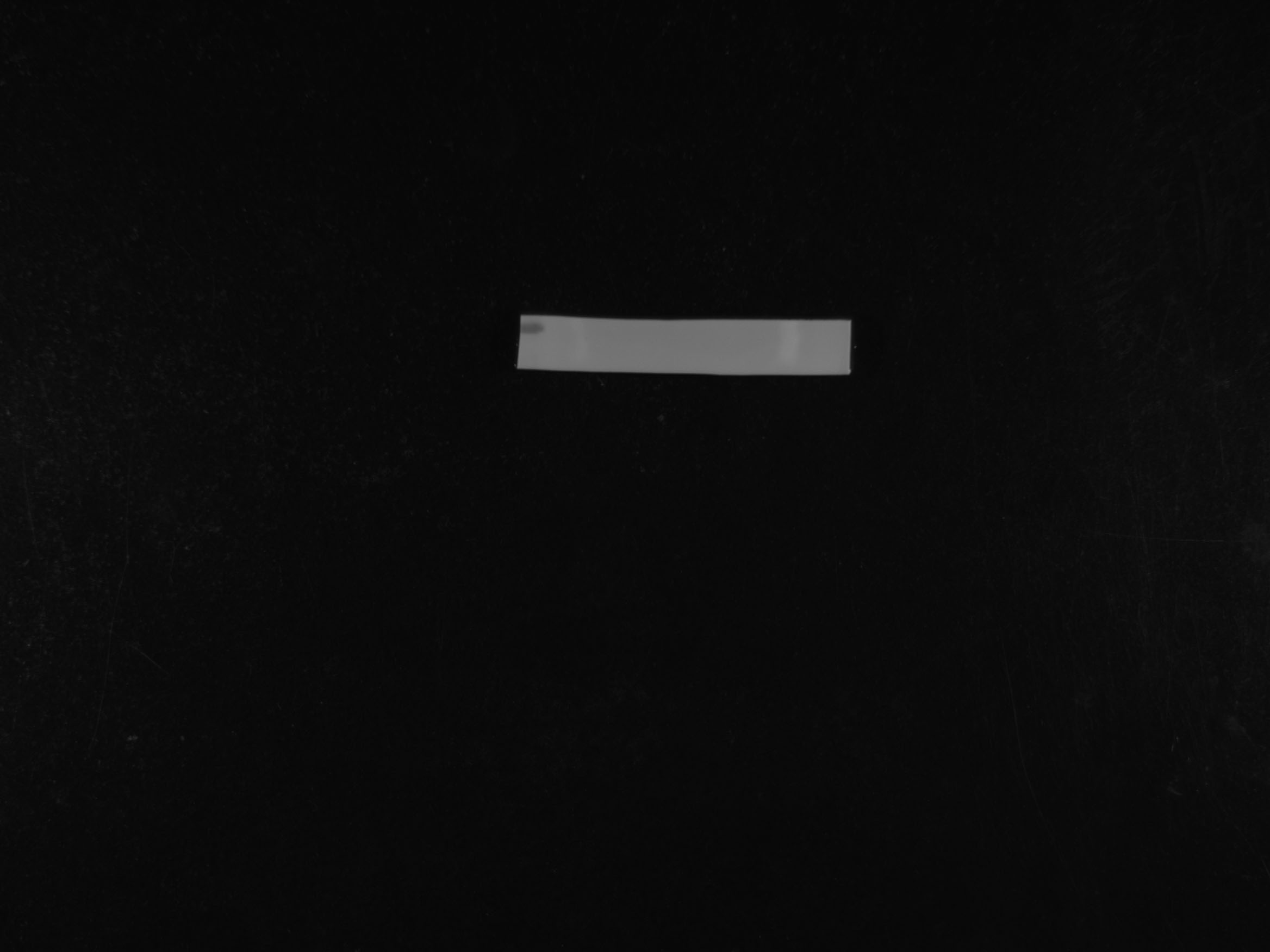

Supplement: Original Images for Blots.zip [file YRER_A_2313366_SM3875.zip › Original Images for Blots/Figure 5/Figure 5D/p-STAT3/Marker.jpg]

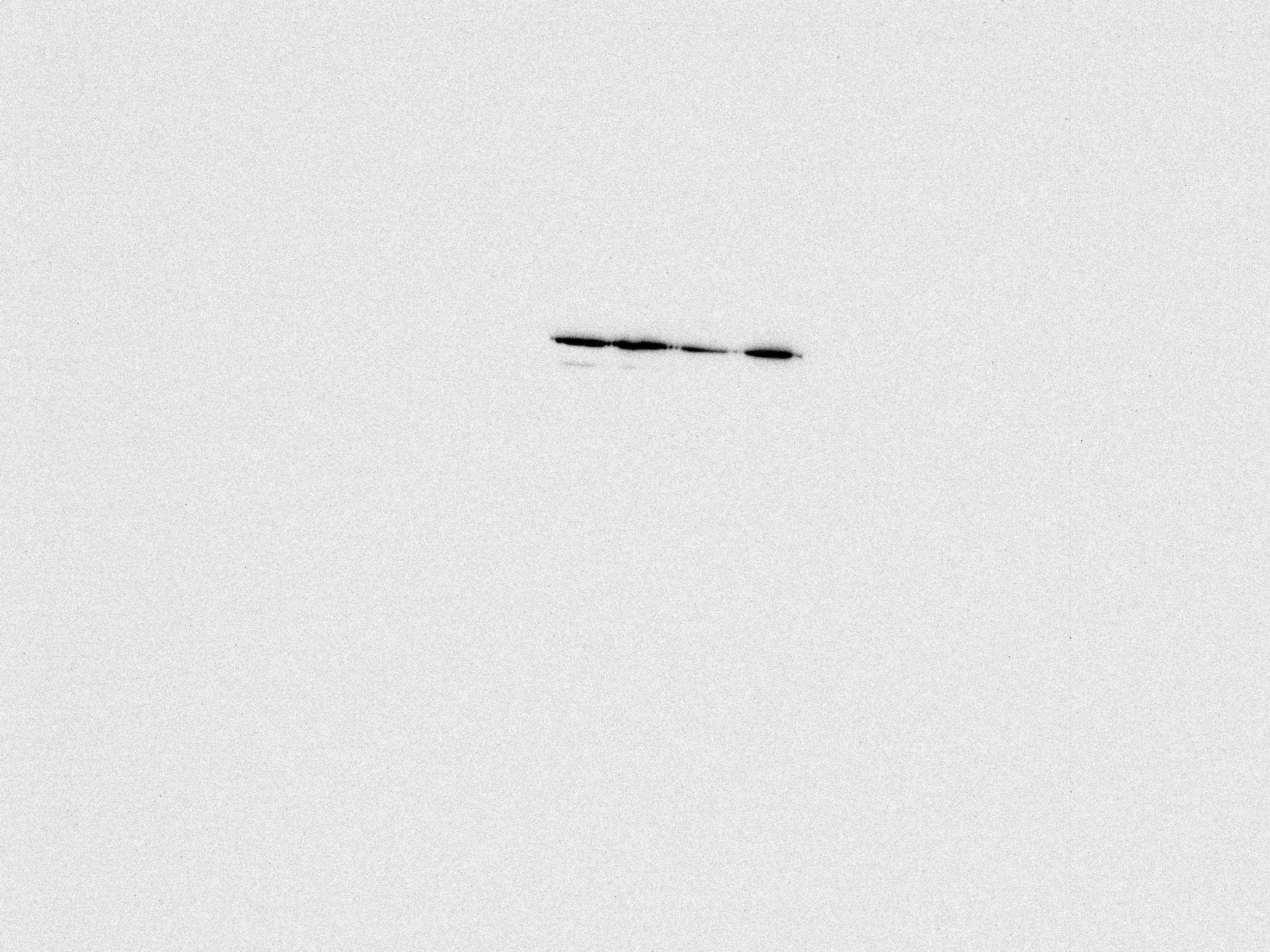

Supplement: Original Images for Blots.zip [file YRER_A_2313366_SM3875.zip › Original Images for Blots/Figure 5/Figure 5D/p-STAT3/p-STAT3.jpg]

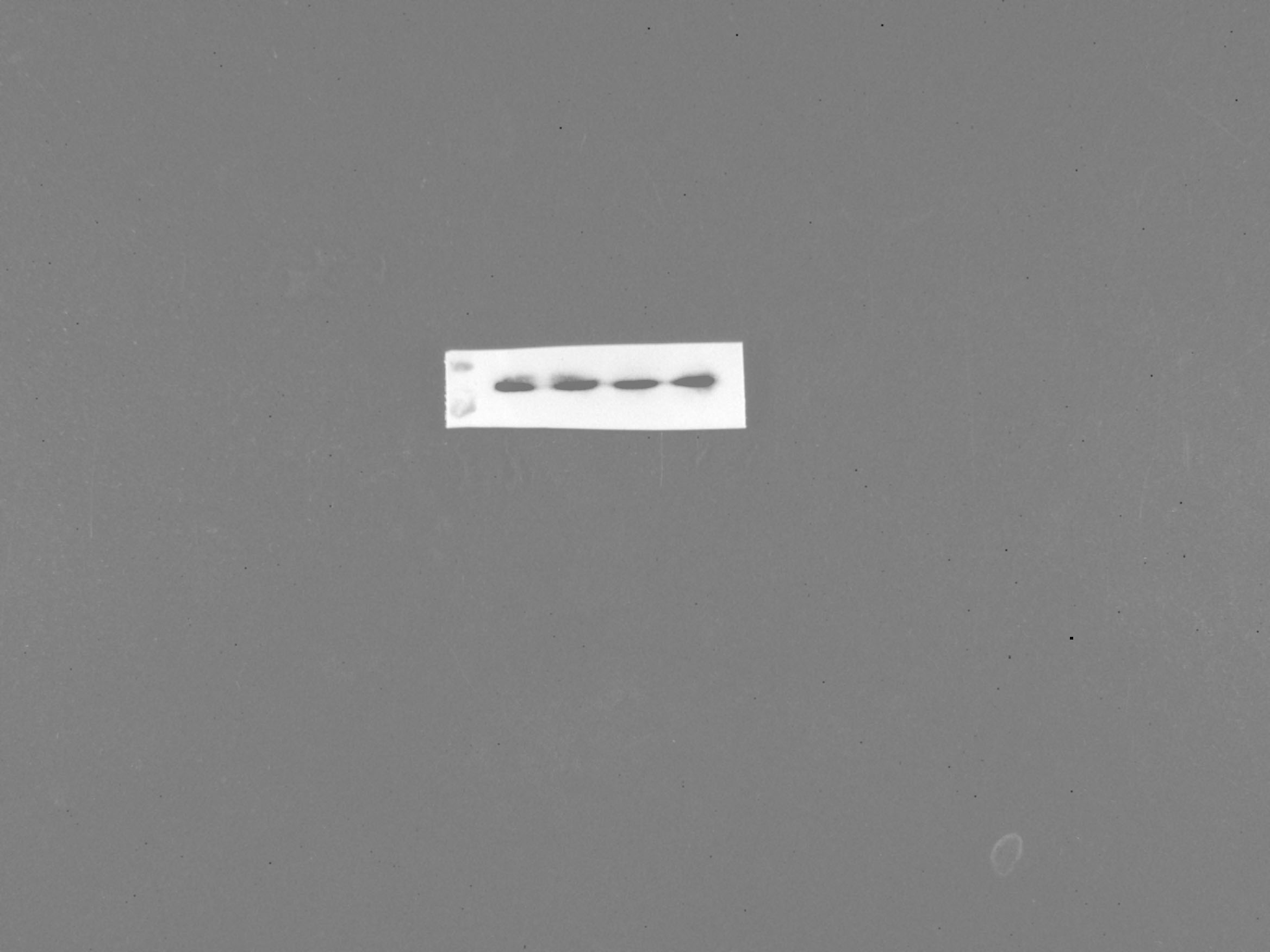

Supplement: Original Images for Blots.zip [file YRER_A_2313366_SM3875.zip › Original Images for Blots/Figure 5/Figure 5D/p38/Marker+p38.jpg]

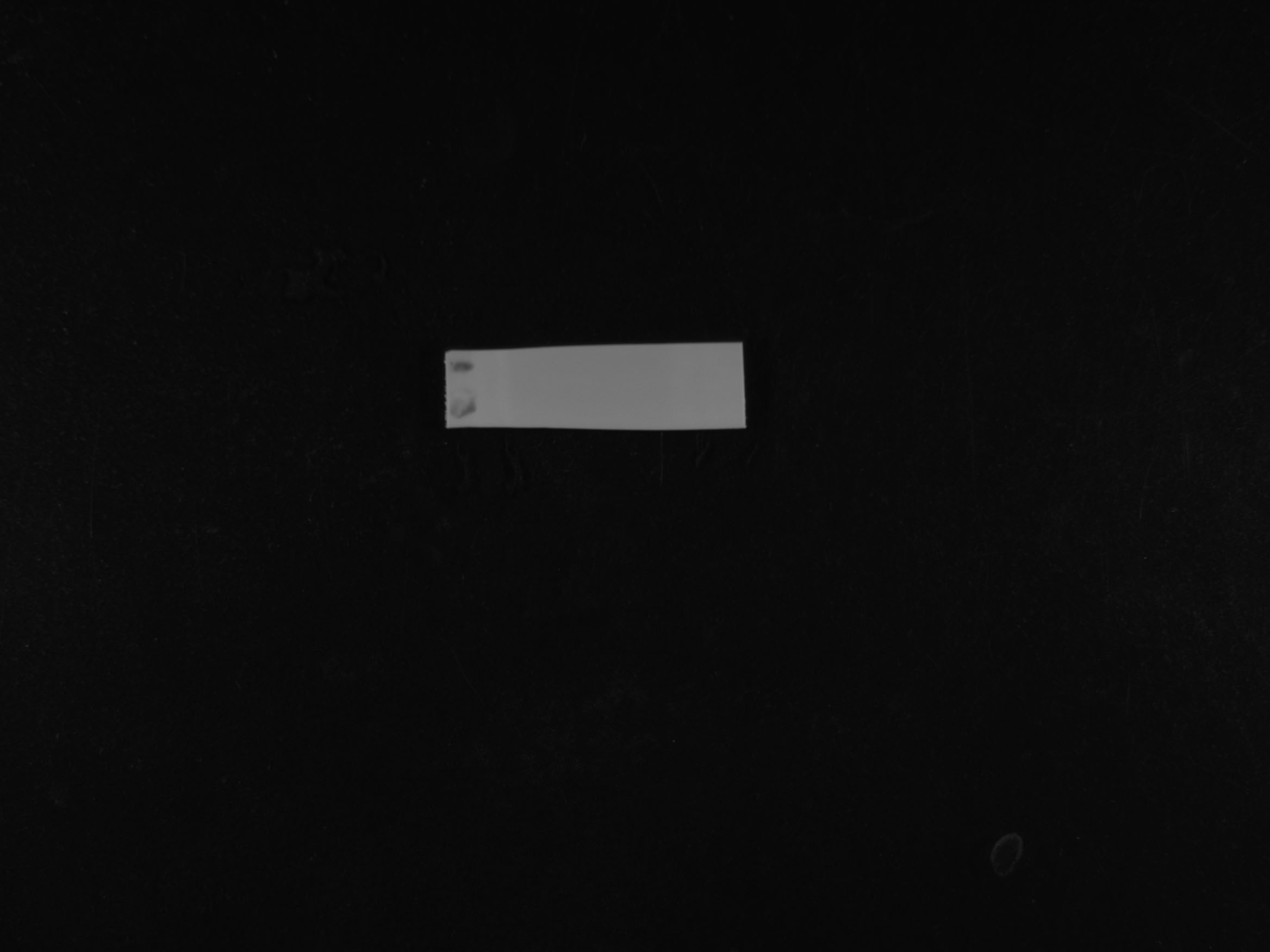

Supplement: Original Images for Blots.zip [file YRER_A_2313366_SM3875.zip › Original Images for Blots/Figure 5/Figure 5D/p38/Marker.jpg]

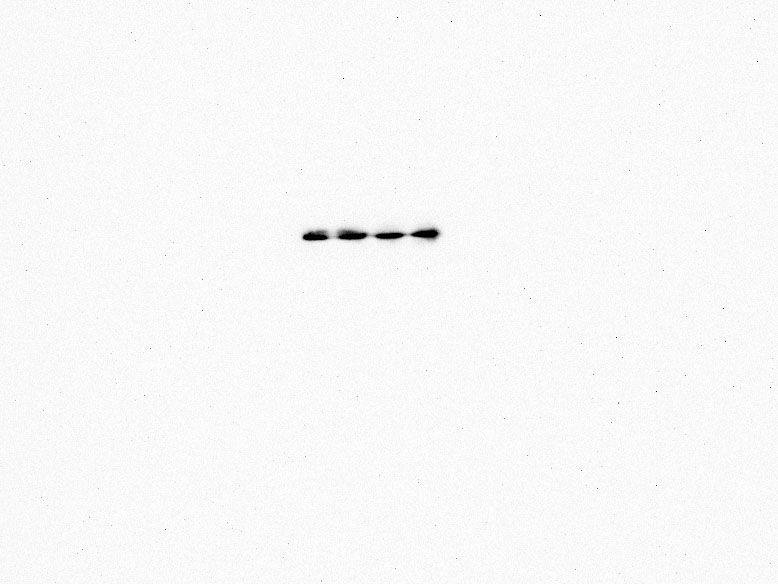

Supplement: Original Images for Blots.zip [file YRER_A_2313366_SM3875.zip › Original Images for Blots/Figure 5/Figure 5D/p38/p38.jpg]

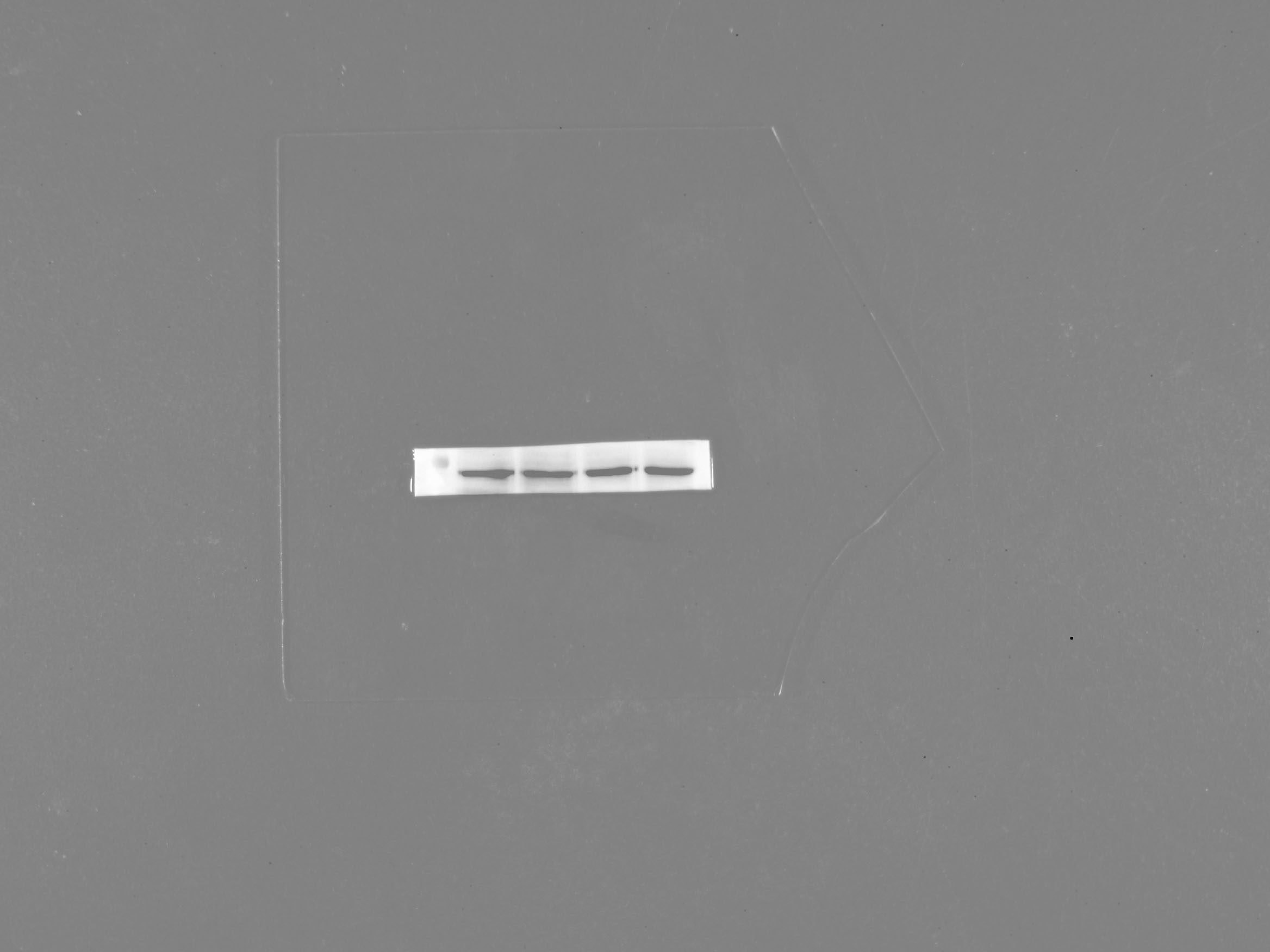

Supplement: Original Images for Blots.zip [file YRER_A_2313366_SM3875.zip › Original Images for Blots/Figure 5/Figure 5D/STAT3/Marker+STAT3.jpg]

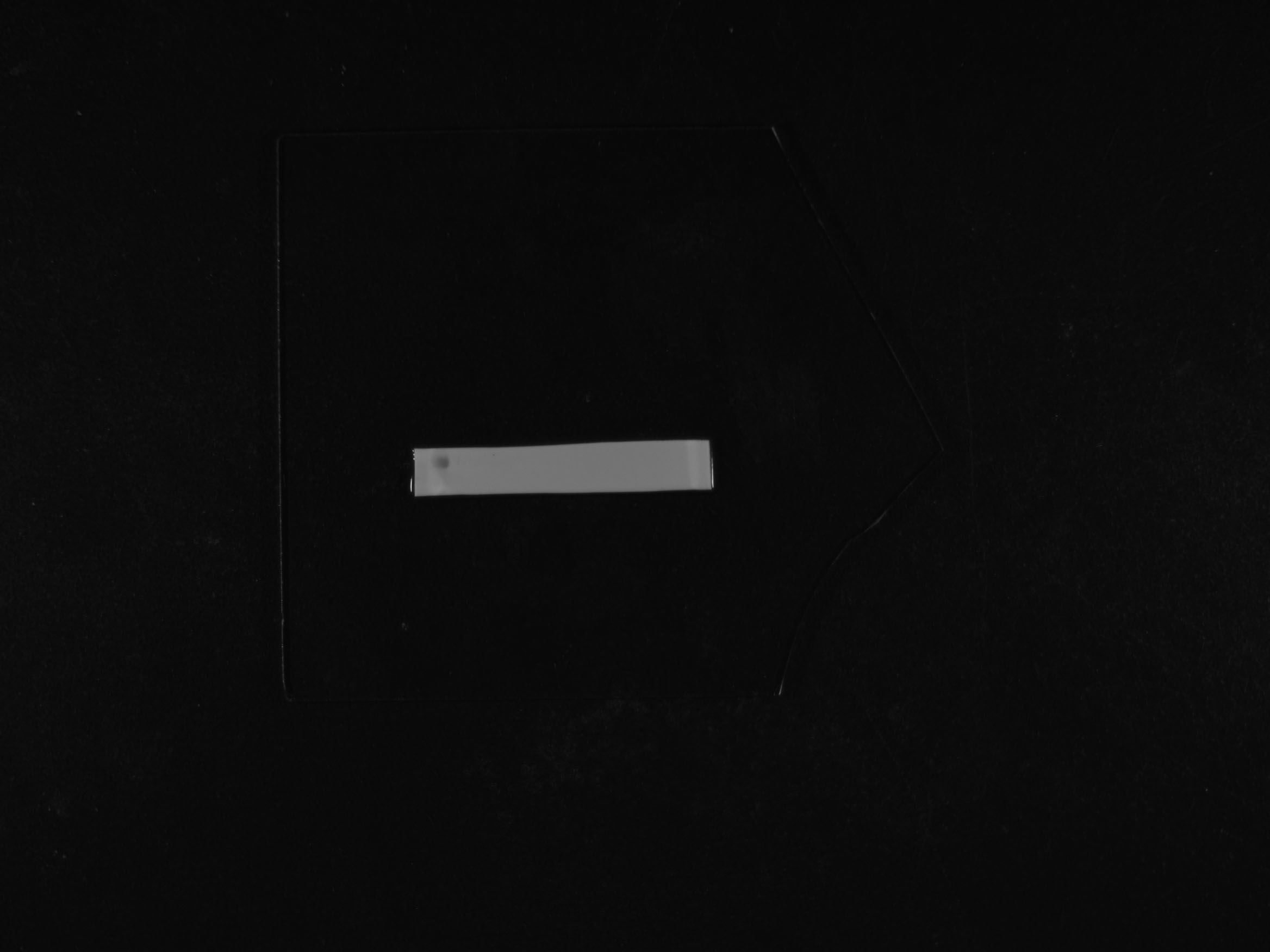

Supplement: Original Images for Blots.zip [file YRER_A_2313366_SM3875.zip › Original Images for Blots/Figure 5/Figure 5D/STAT3/Marker.jpg]

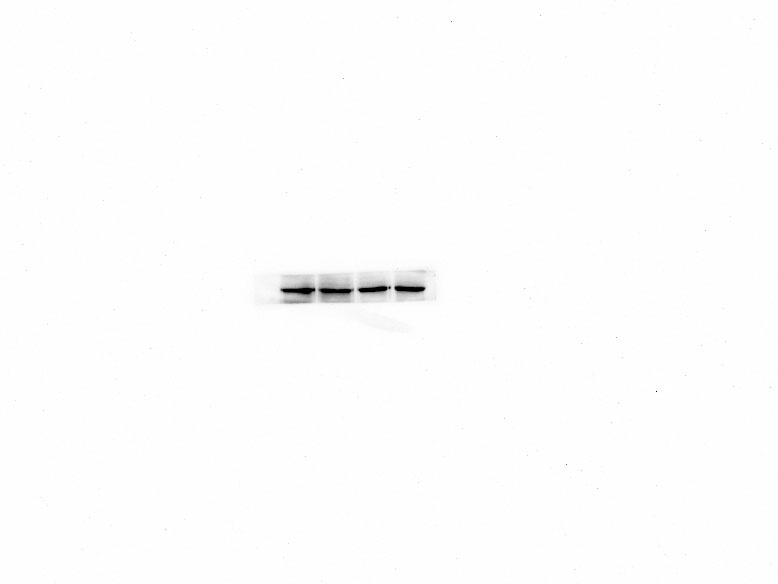

Supplement: Original Images for Blots.zip [file YRER_A_2313366_SM3875.zip › Original Images for Blots/Figure 5/Figure 5D/STAT3/STAT3.jpg]

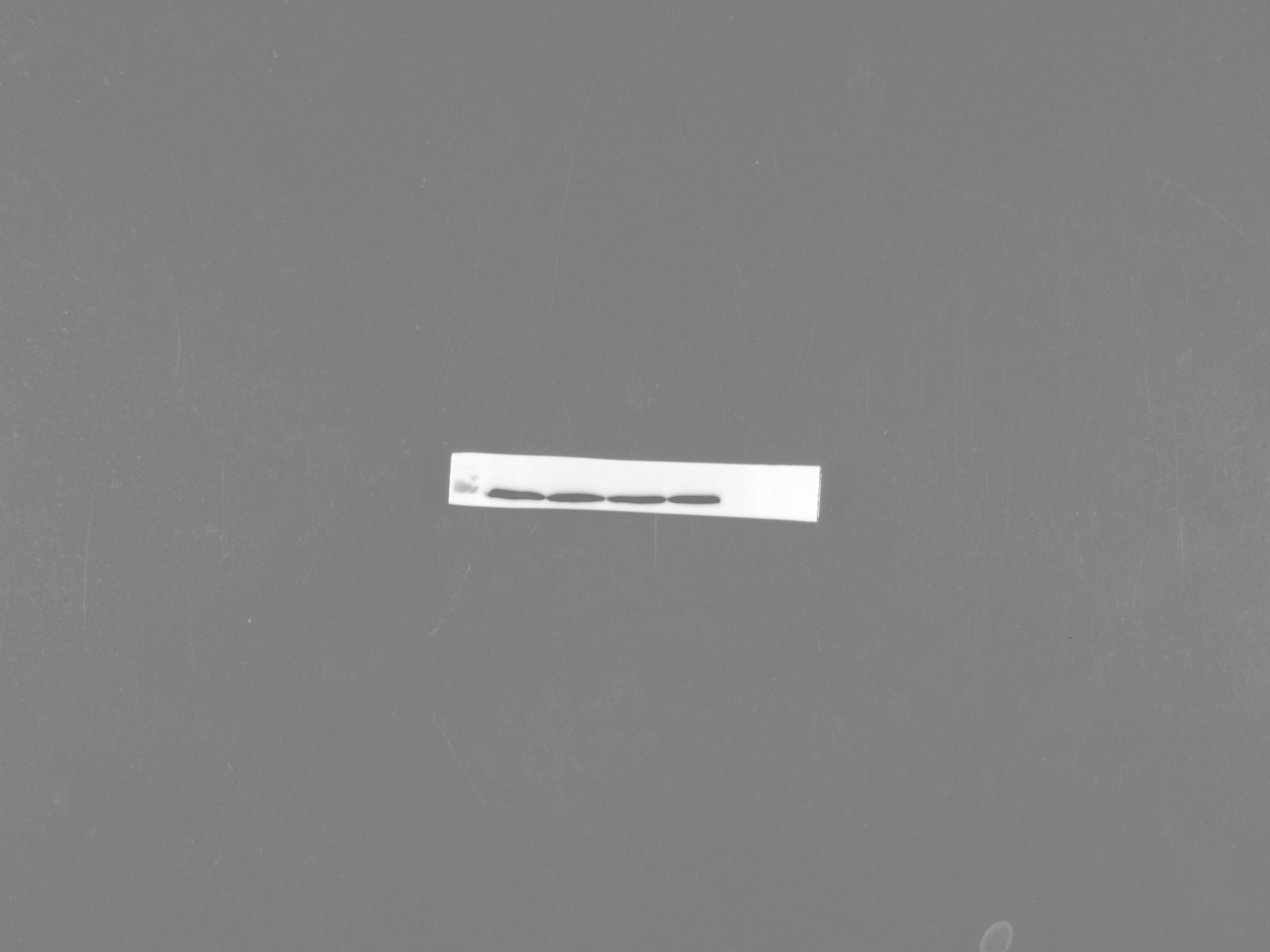

Supplement: Original Images for Blots.zip [file YRER_A_2313366_SM3875.zip › Original Images for Blots/Figure 5/Figure 5D/α-tubulin/Marker+α-tubulin.jpg]

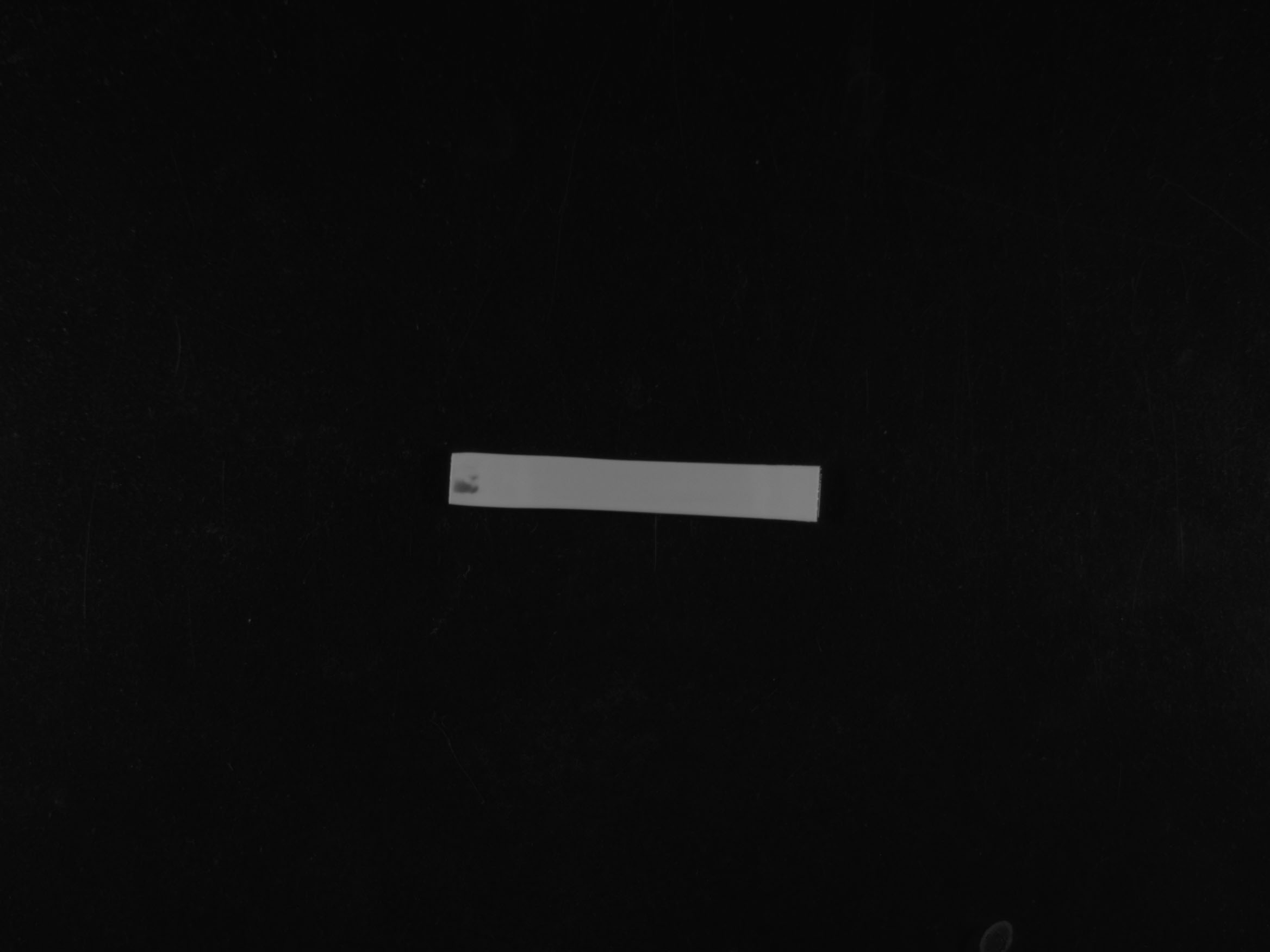

Supplement: Original Images for Blots.zip [file YRER_A_2313366_SM3875.zip › Original Images for Blots/Figure 5/Figure 5D/α-tubulin/Marker.jpg]

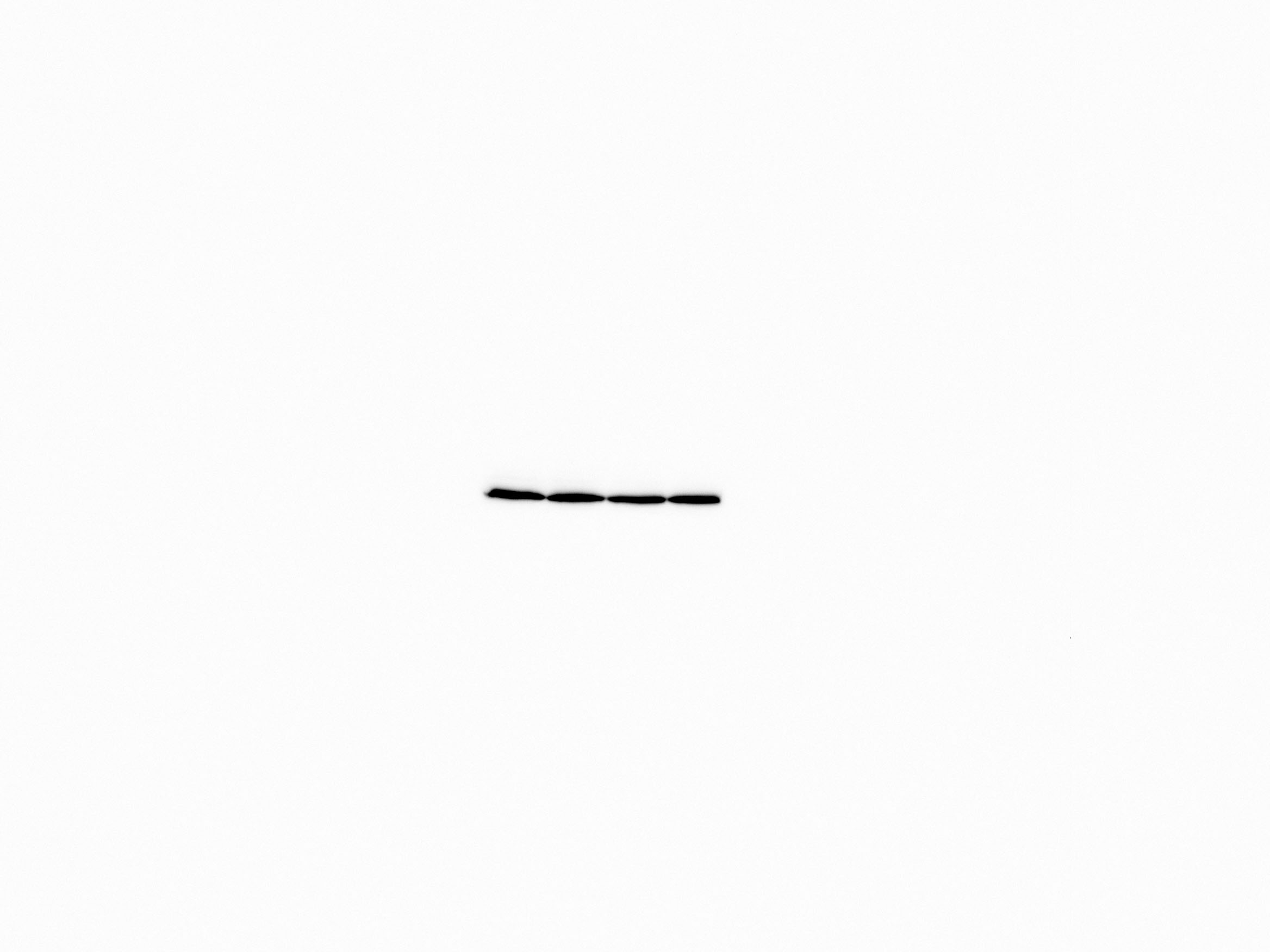

Supplement: Original Images for Blots.zip [file YRER_A_2313366_SM3875.zip › Original Images for Blots/Figure 5/Figure 5D/α-tubulin/α-tubulin.jpg]

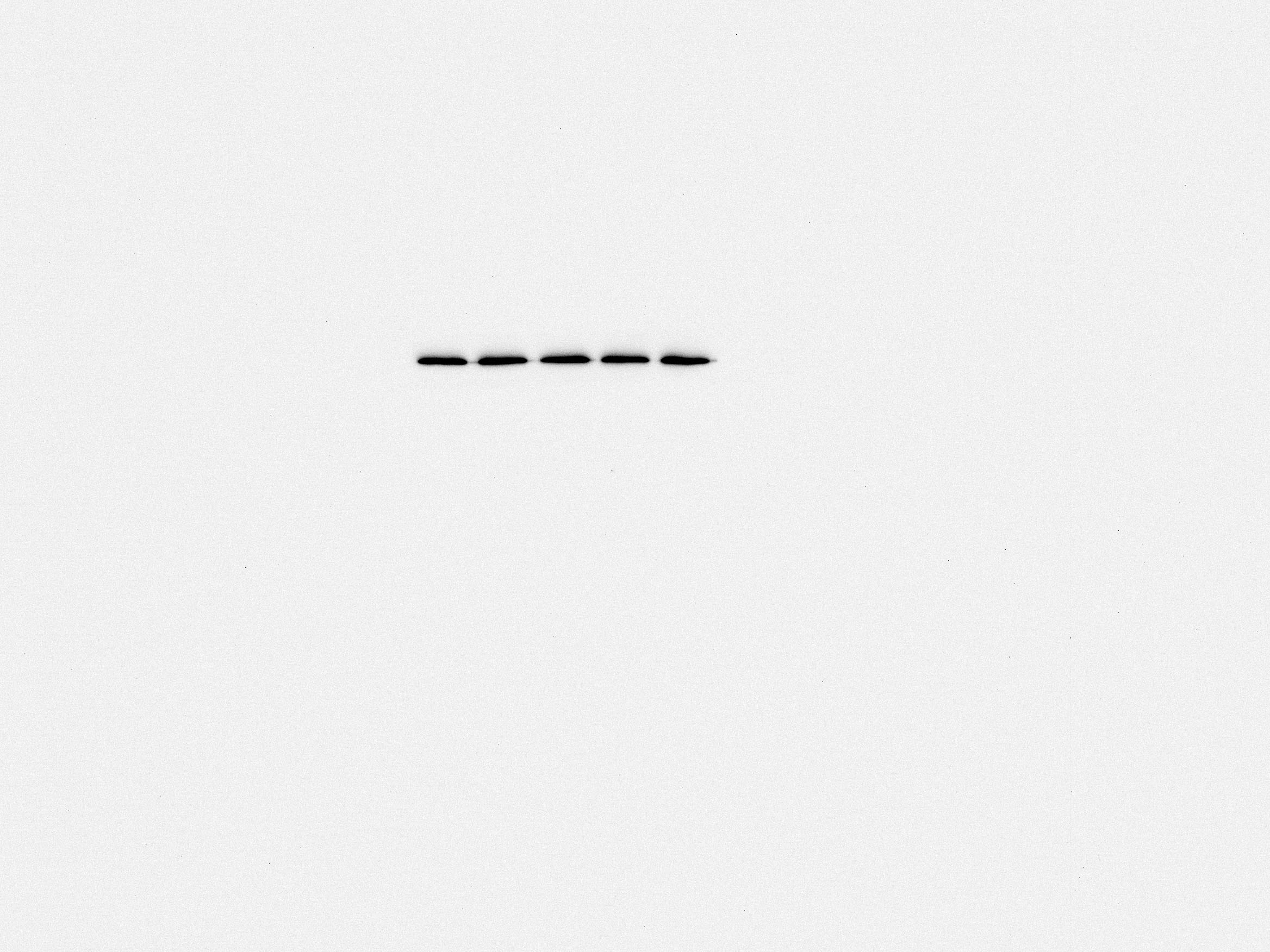

Supplement: Original Images for Blots.zip [file YRER_A_2313366_SM3875.zip › Original Images for Blots/Figure 6/Figure 6C/AKT/AKT.jpg]

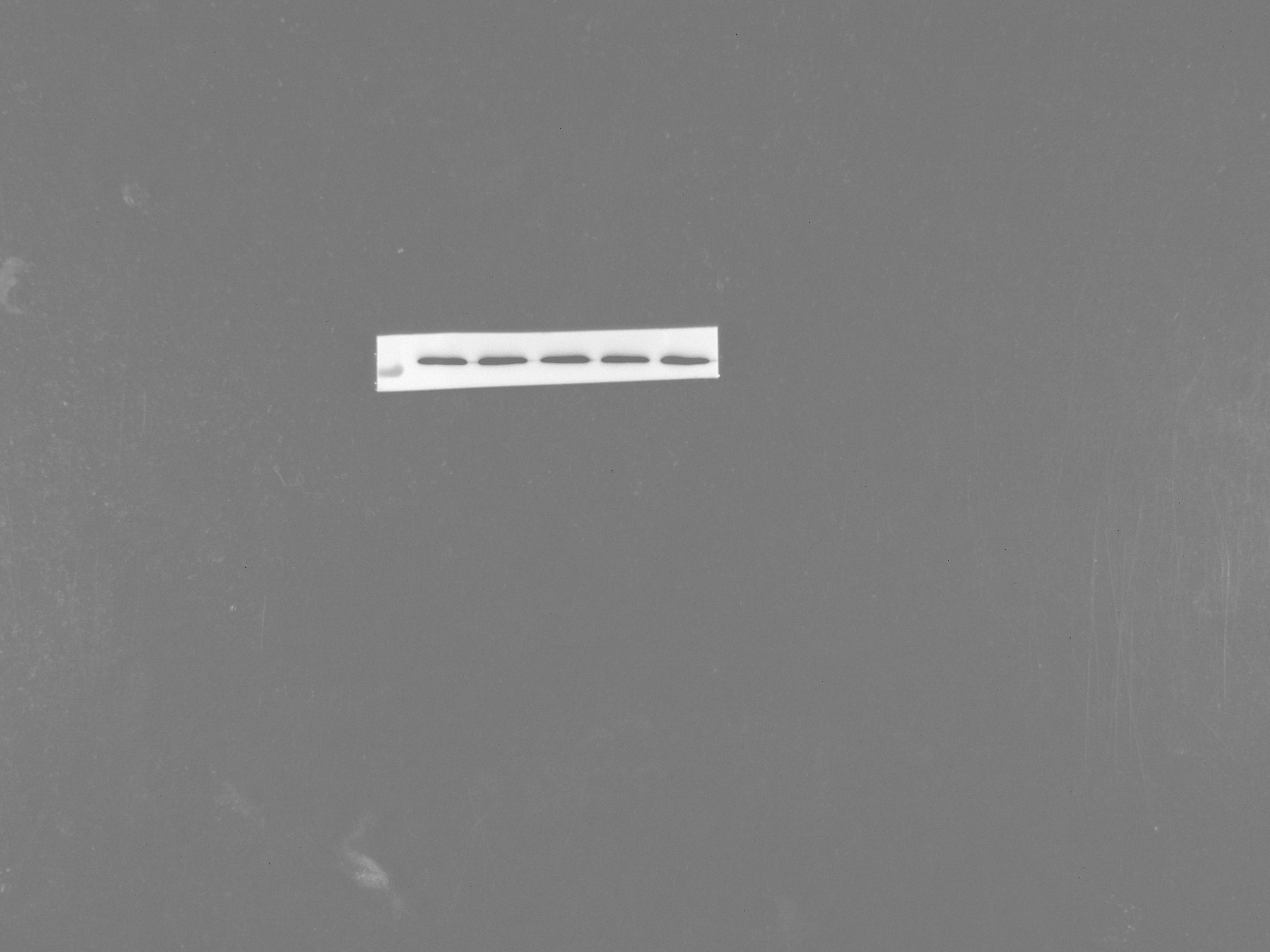

Supplement: Original Images for Blots.zip [file YRER_A_2313366_SM3875.zip › Original Images for Blots/Figure 6/Figure 6C/AKT/Marker+AKT.jpg]

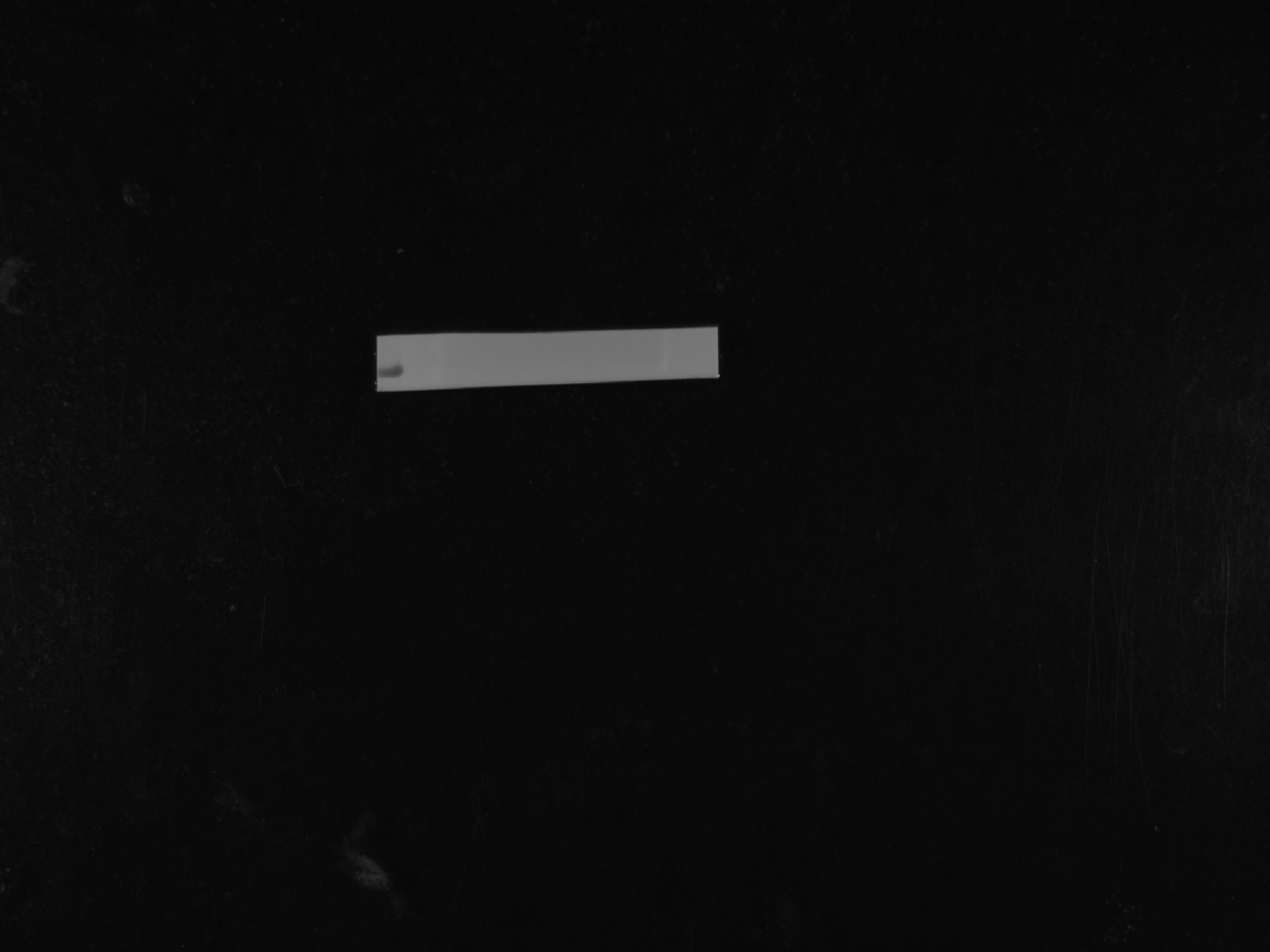

Supplement: Original Images for Blots.zip [file YRER_A_2313366_SM3875.zip › Original Images for Blots/Figure 6/Figure 6C/AKT/Marker.jpg]

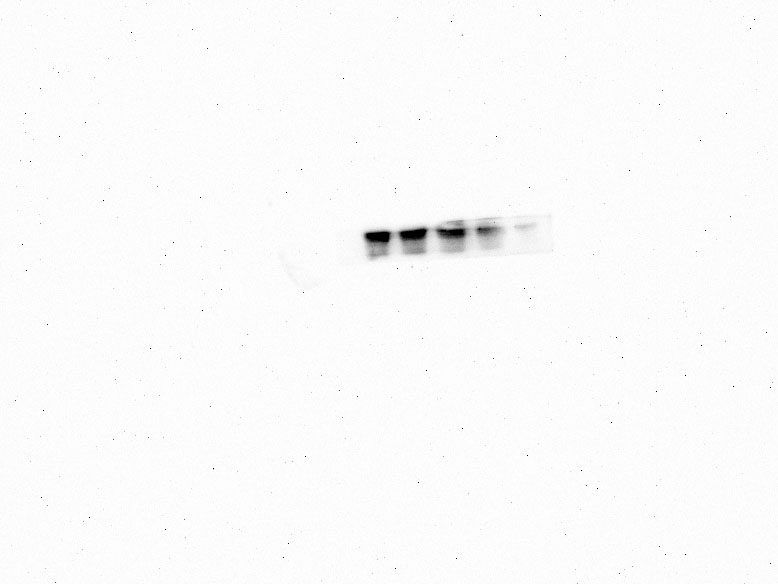

Supplement: Original Images for Blots.zip [file YRER_A_2313366_SM3875.zip › Original Images for Blots/Figure 6/Figure 6C/CDK2/CDK2.jpg]

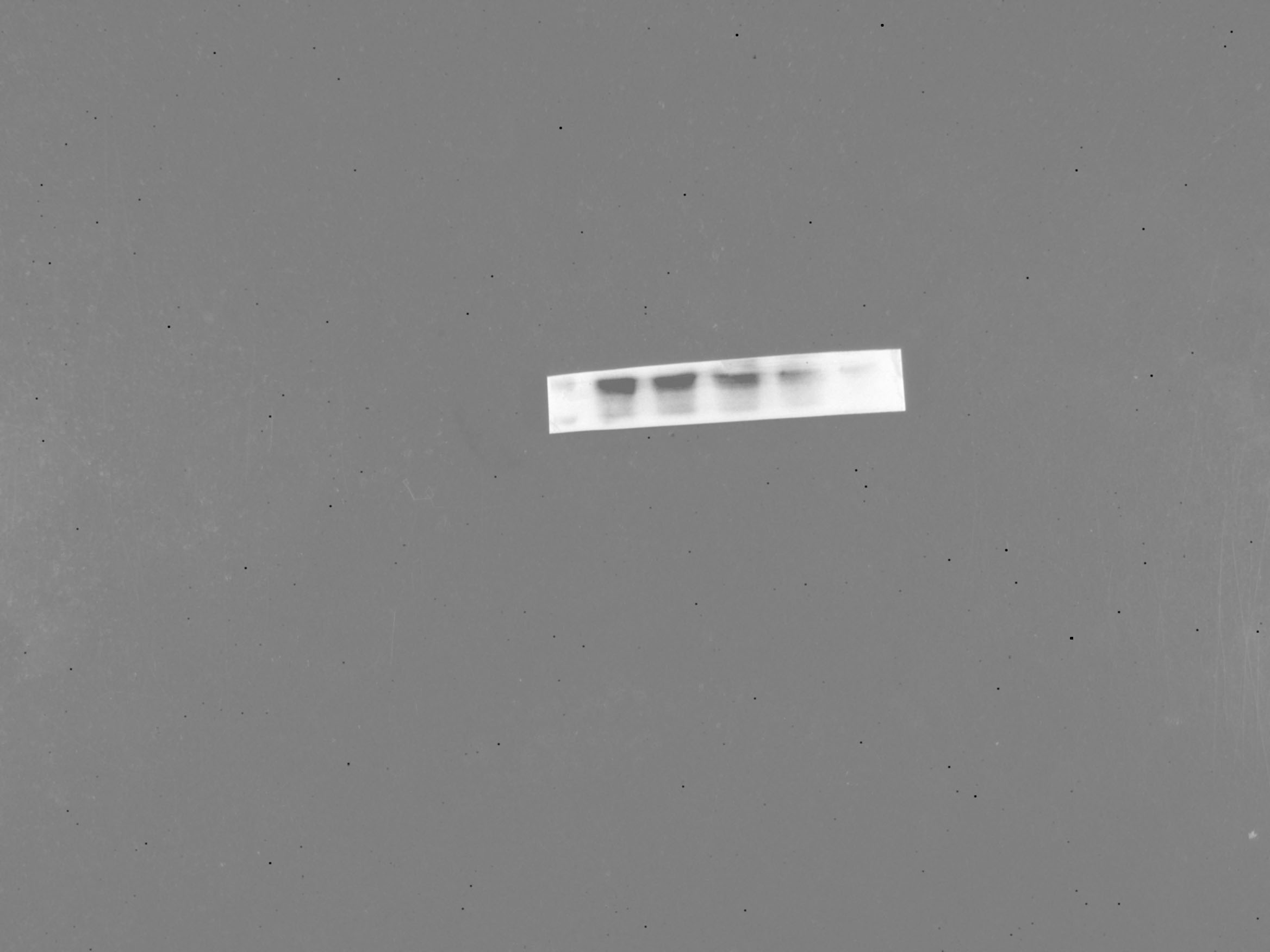

Supplement: Original Images for Blots.zip [file YRER_A_2313366_SM3875.zip › Original Images for Blots/Figure 6/Figure 6C/CDK2/Marker+CDK2.jpg]

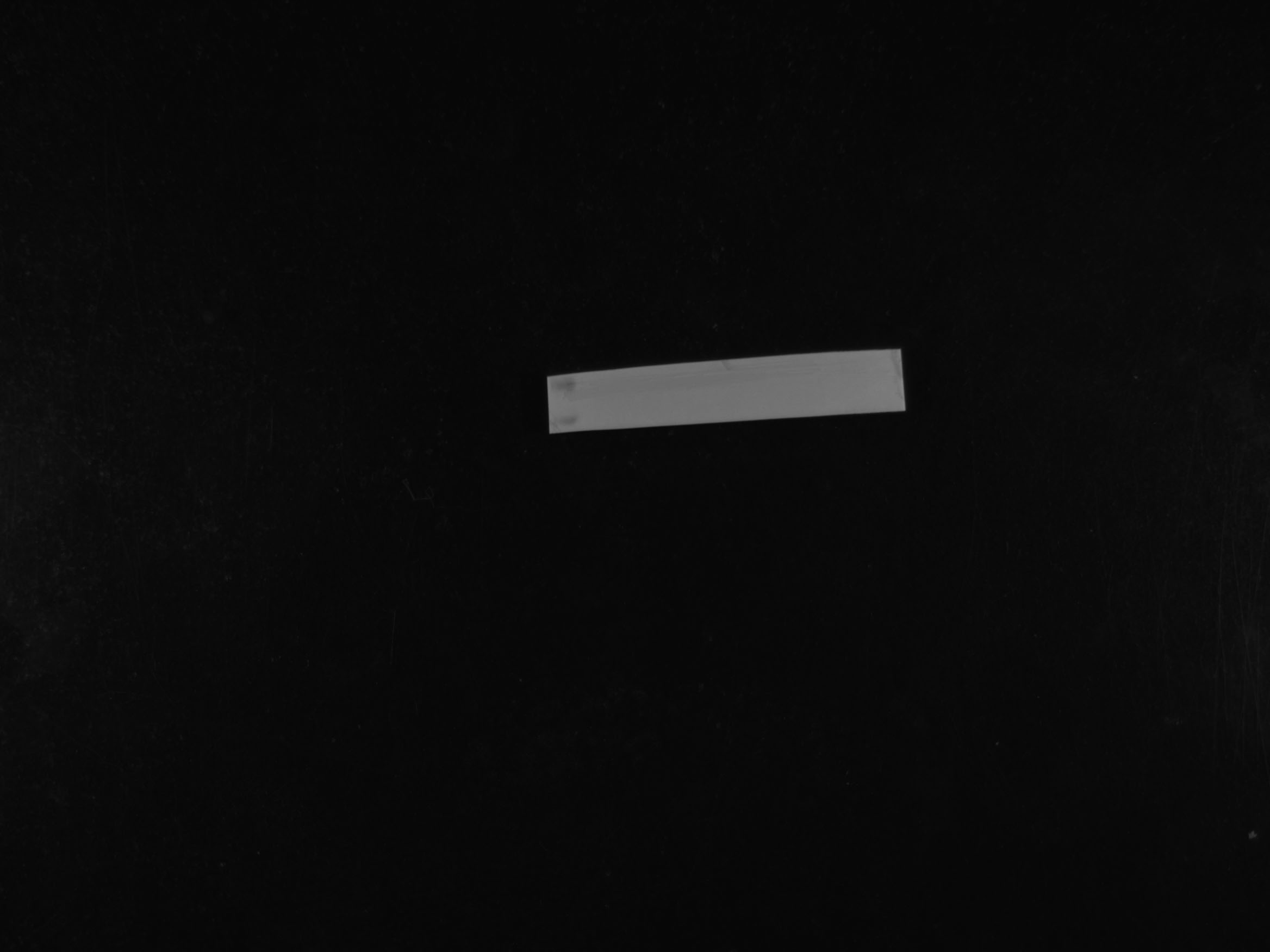

Supplement: Original Images for Blots.zip [file YRER_A_2313366_SM3875.zip › Original Images for Blots/Figure 6/Figure 6C/CDK2/Marker.jpg]

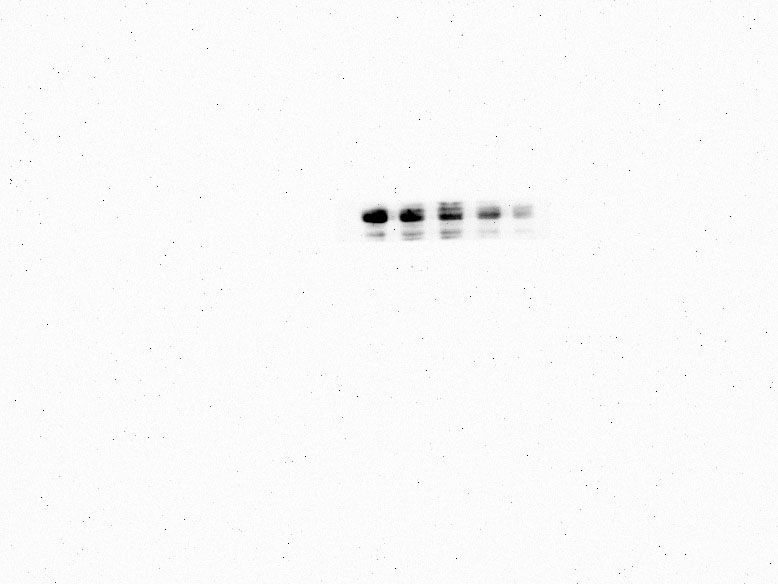

Supplement: Original Images for Blots.zip [file YRER_A_2313366_SM3875.zip › Original Images for Blots/Figure 6/Figure 6C/CDK4/CDK4.jpg]

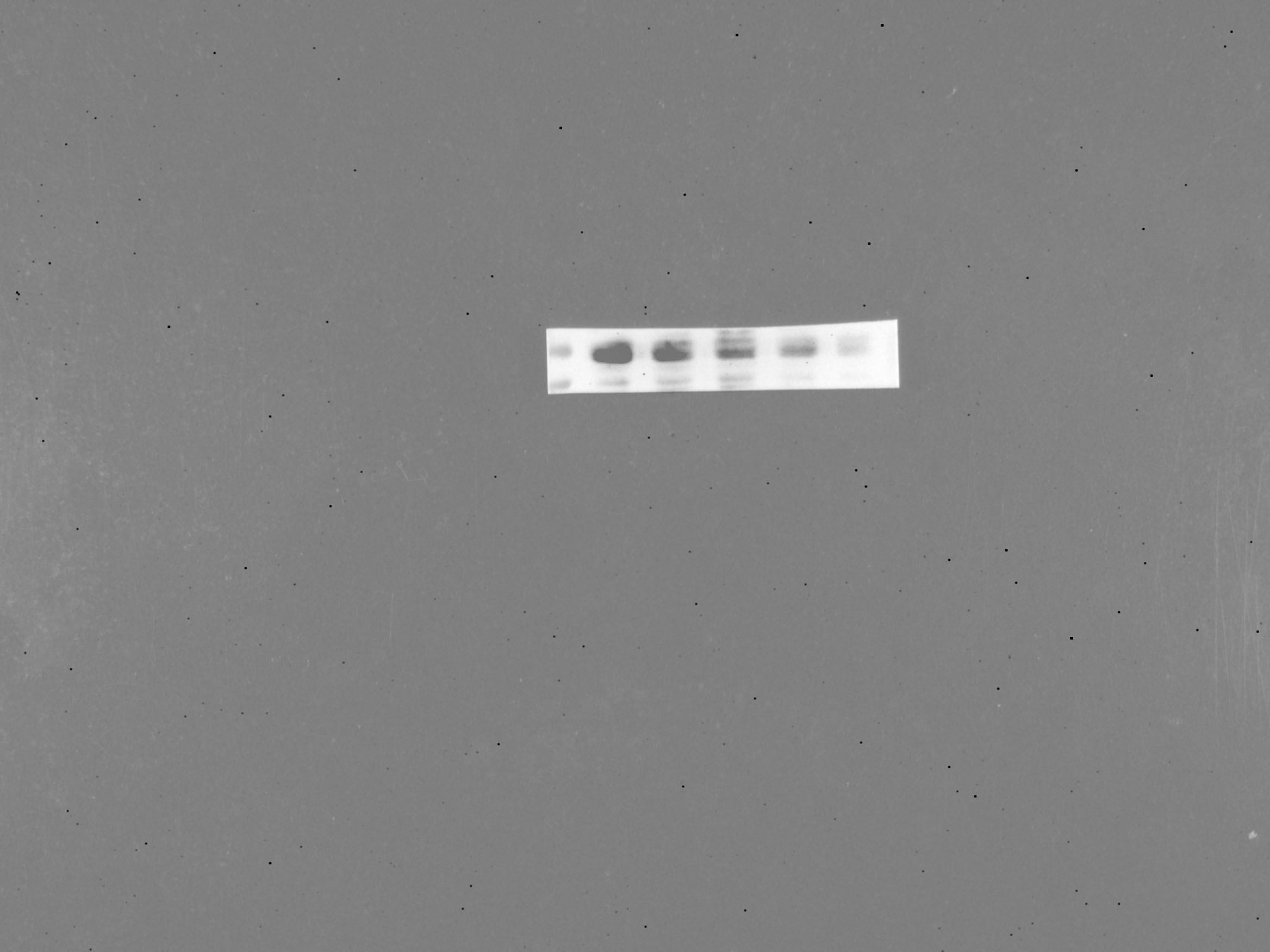

Supplement: Original Images for Blots.zip [file YRER_A_2313366_SM3875.zip › Original Images for Blots/Figure 6/Figure 6C/CDK4/Marker+CDK4.jpg]

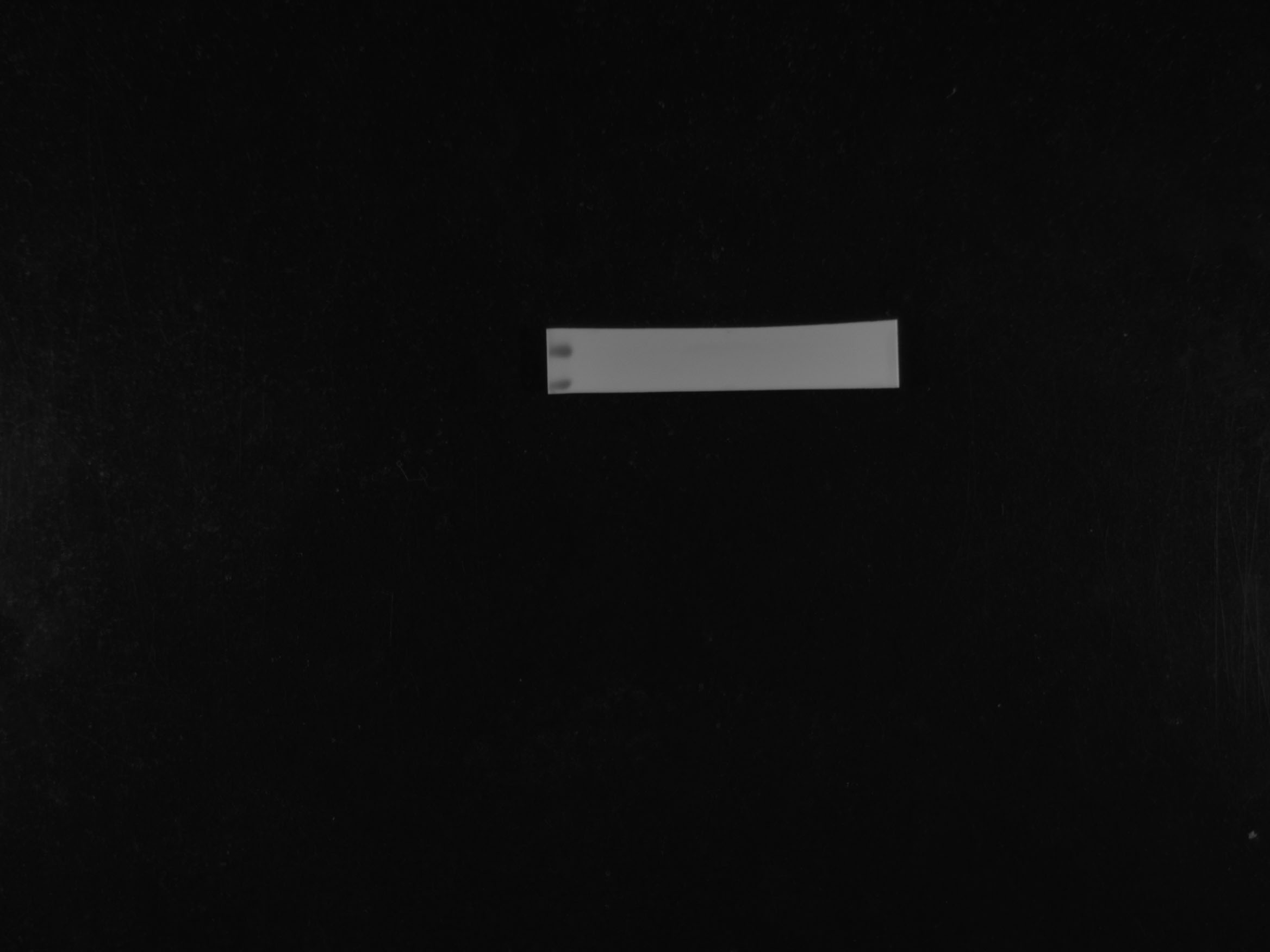

Supplement: Original Images for Blots.zip [file YRER_A_2313366_SM3875.zip › Original Images for Blots/Figure 6/Figure 6C/CDK4/Marker.jpg]

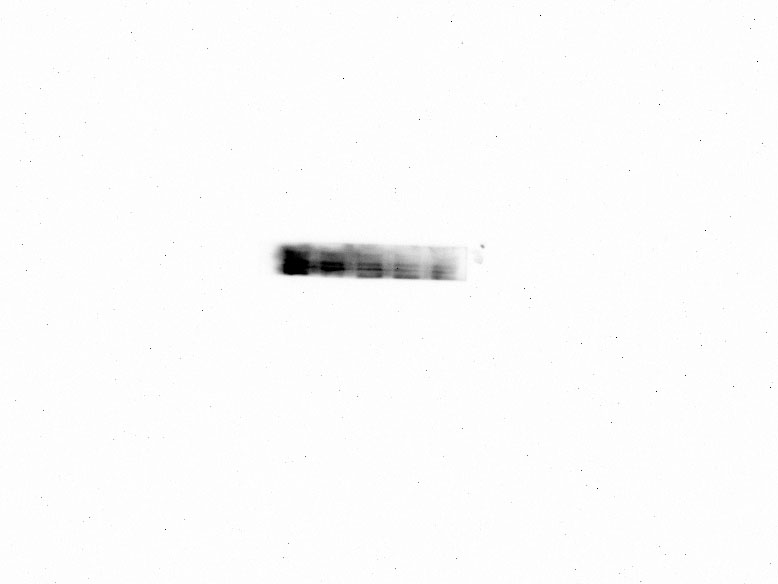

Supplement: Original Images for Blots.zip [file YRER_A_2313366_SM3875.zip › Original Images for Blots/Figure 6/Figure 6C/CDK6/CDK6.jpg]

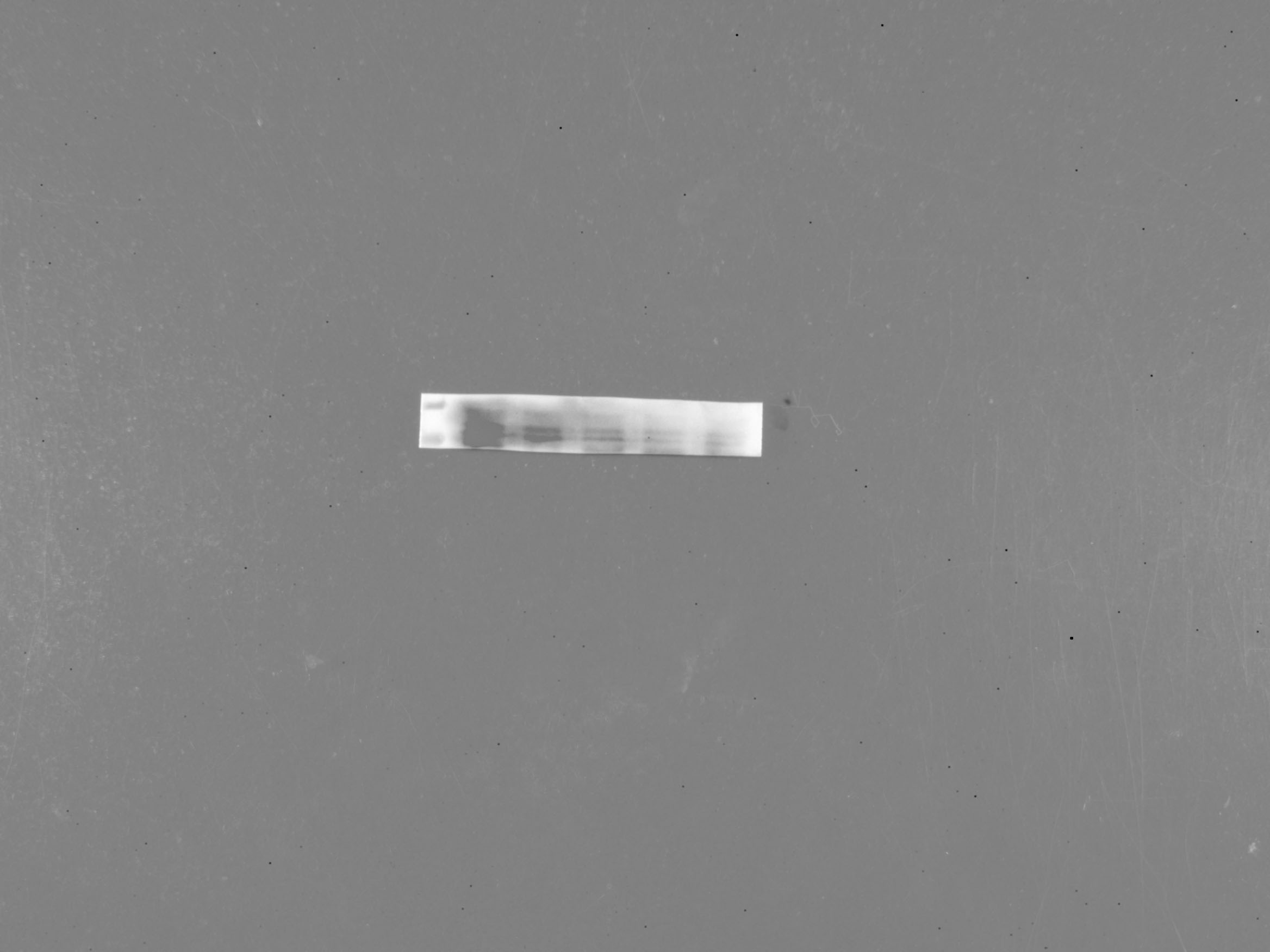

Supplement: Original Images for Blots.zip [file YRER_A_2313366_SM3875.zip › Original Images for Blots/Figure 6/Figure 6C/CDK6/Marker+CDK6.jpg]

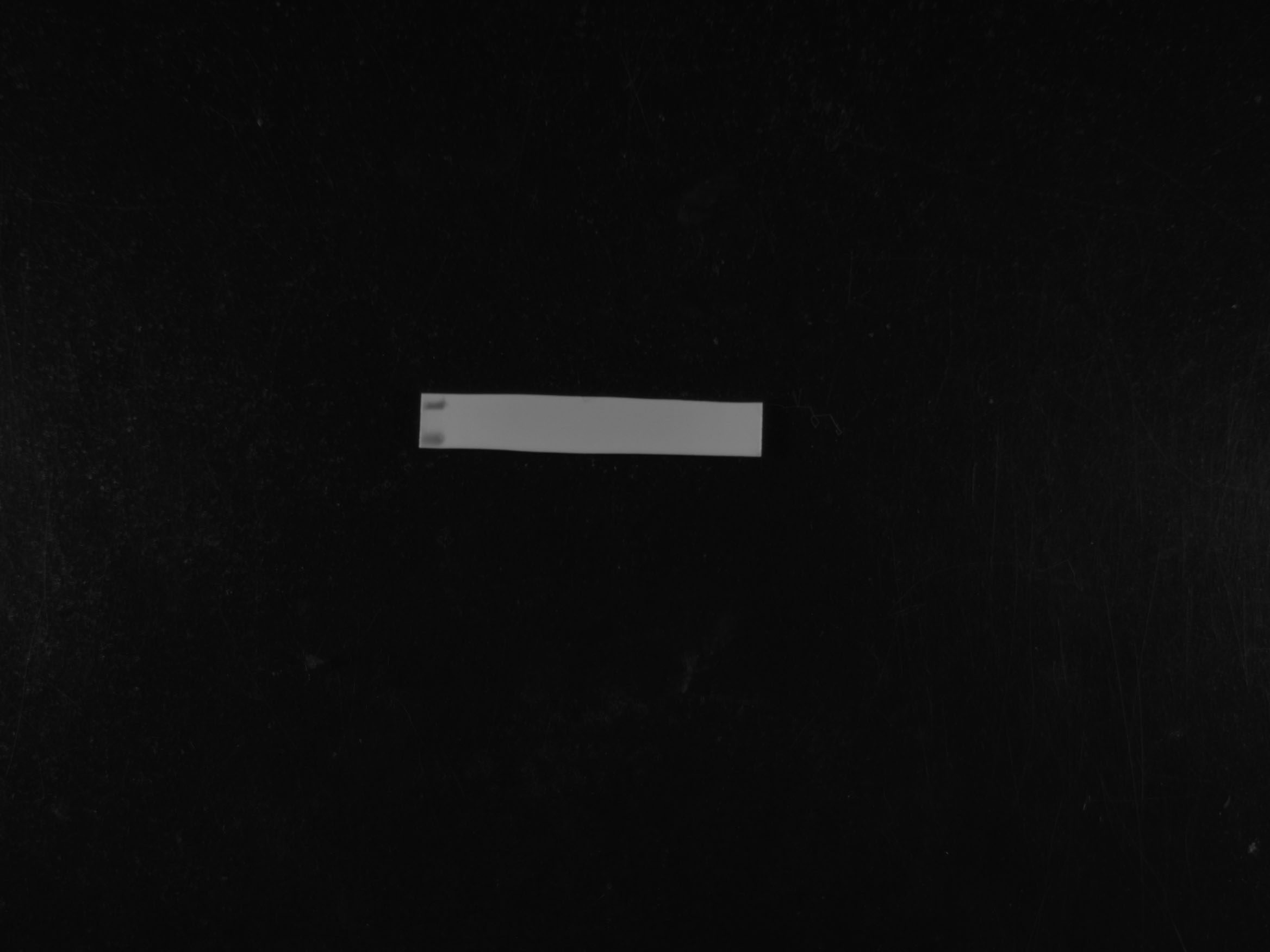

Supplement: Original Images for Blots.zip [file YRER_A_2313366_SM3875.zip › Original Images for Blots/Figure 6/Figure 6C/CDK6/Marker.jpg]

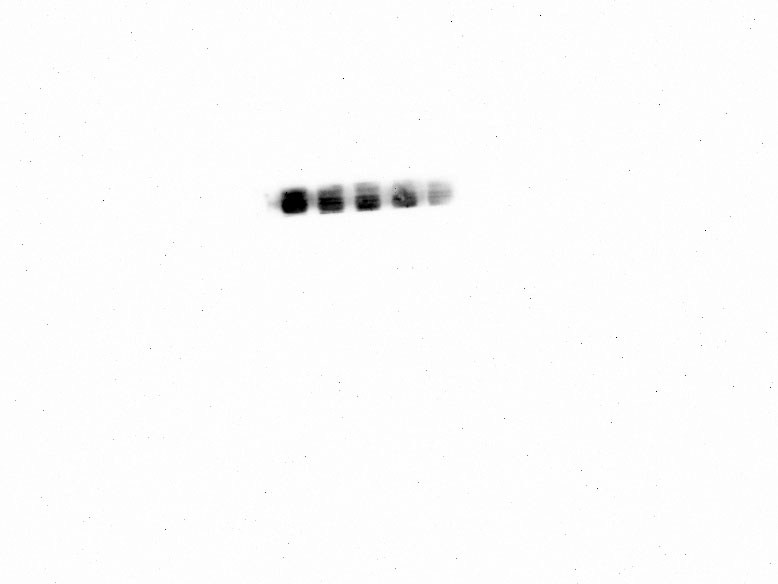

Supplement: Original Images for Blots.zip [file YRER_A_2313366_SM3875.zip › Original Images for Blots/Figure 6/Figure 6C/CyclinD1/CyclinD1.jpg]

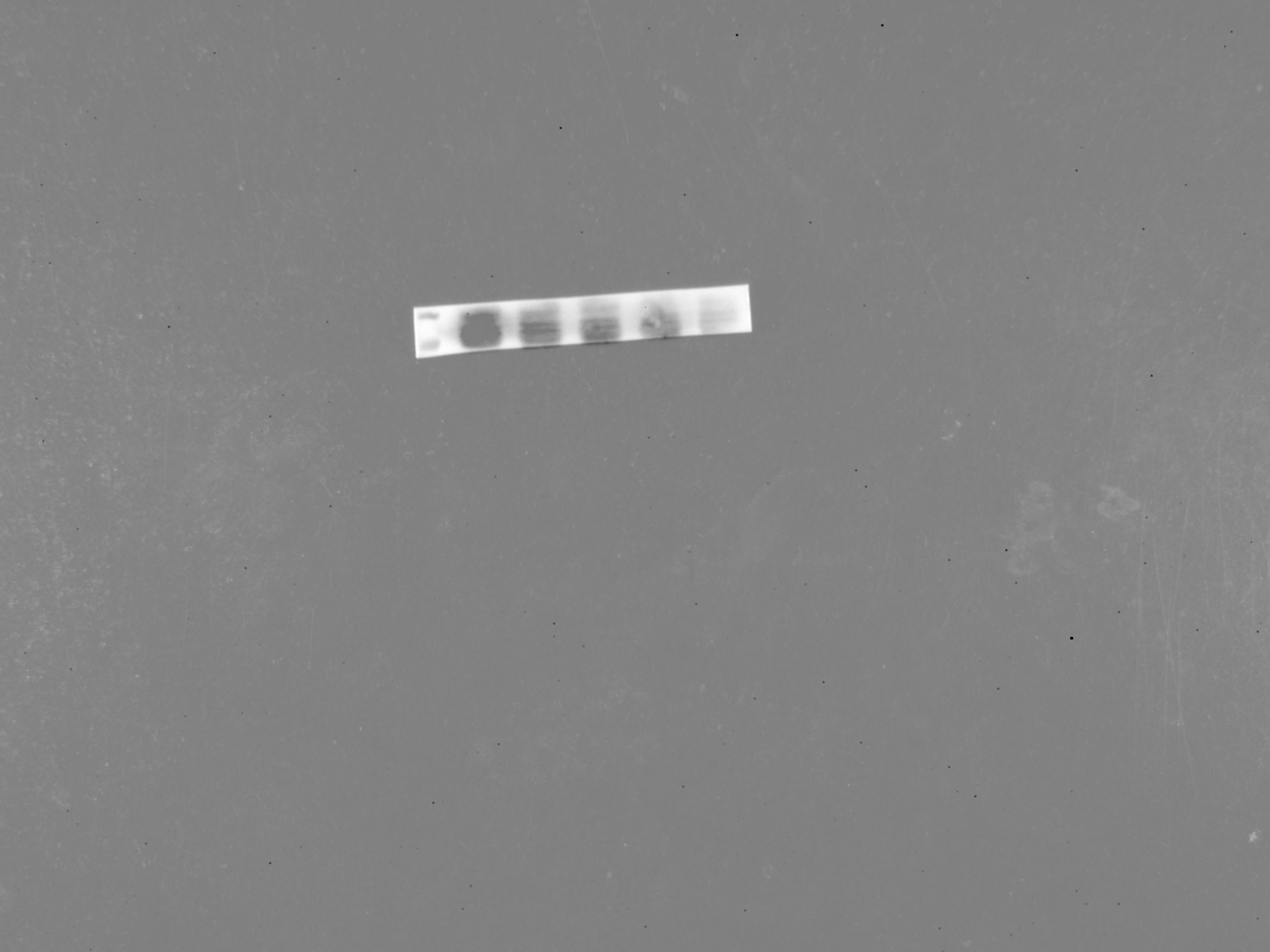

Supplement: Original Images for Blots.zip [file YRER_A_2313366_SM3875.zip › Original Images for Blots/Figure 6/Figure 6C/CyclinD1/Marker+CyclinD1.jpg]

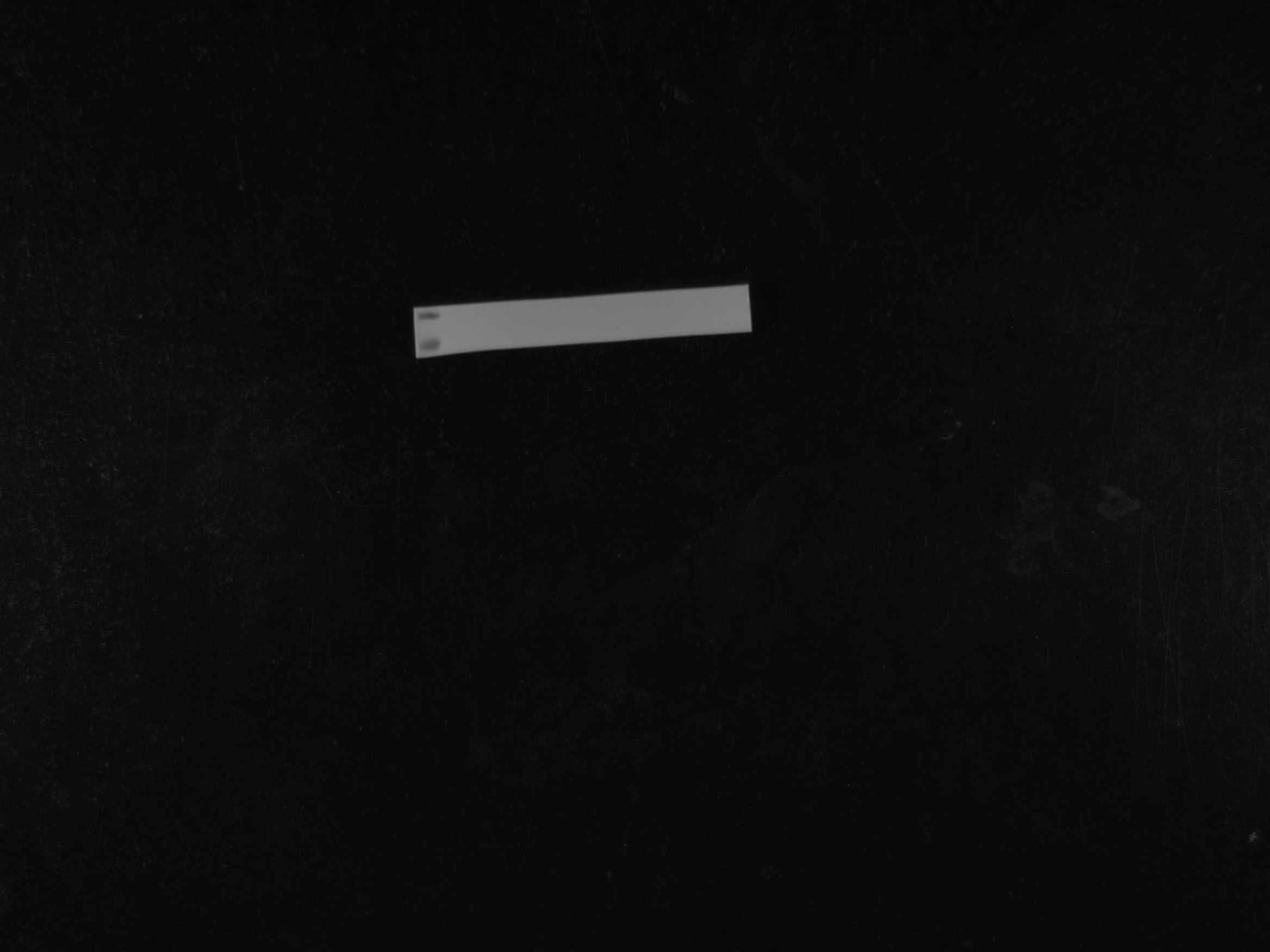

Supplement: Original Images for Blots.zip [file YRER_A_2313366_SM3875.zip › Original Images for Blots/Figure 6/Figure 6C/CyclinD1/Marker.jpg]

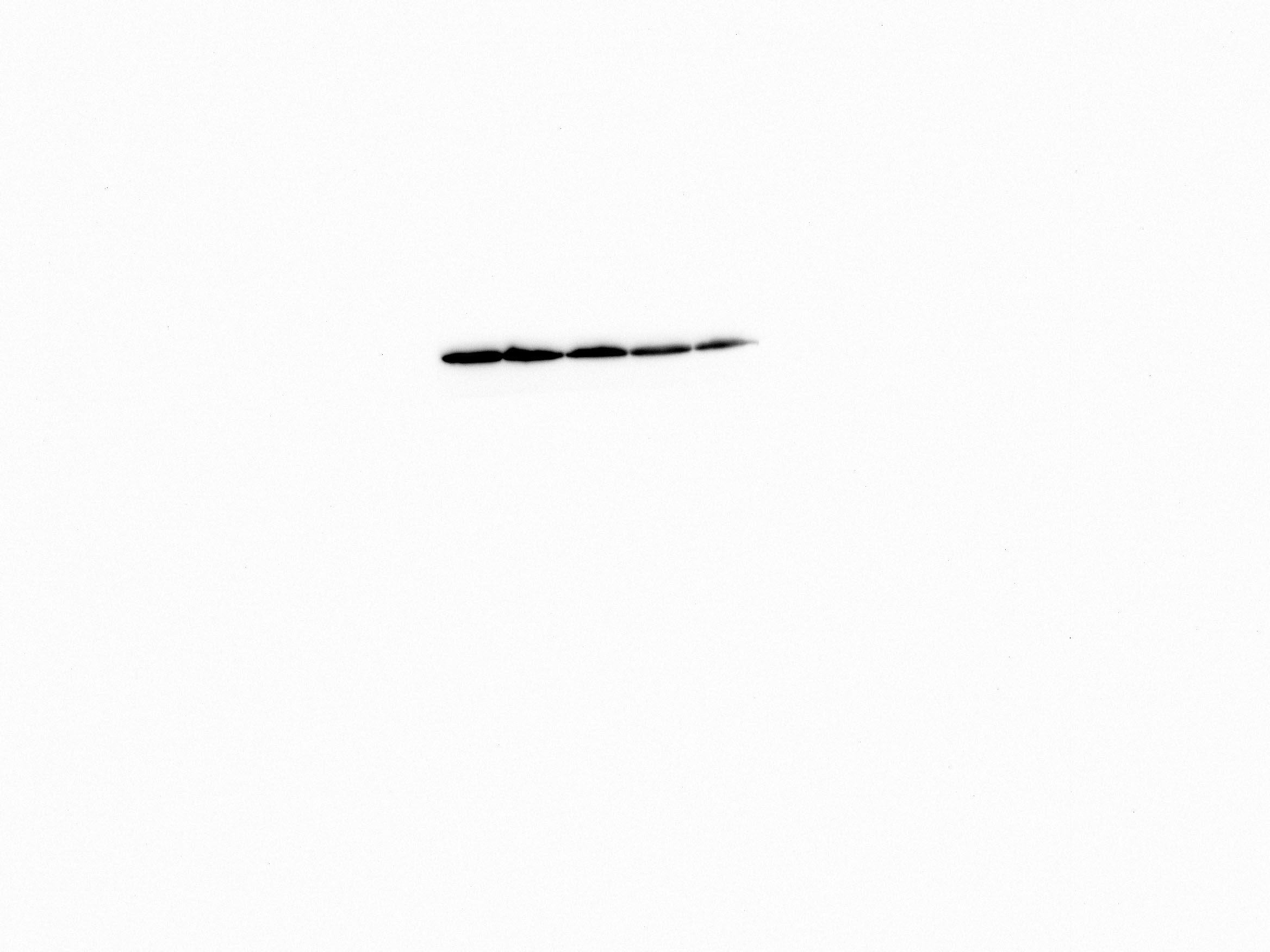

Supplement: Original Images for Blots.zip [file YRER_A_2313366_SM3875.zip › Original Images for Blots/Figure 6/Figure 6C/CyclinE/CyclinE.jpg]

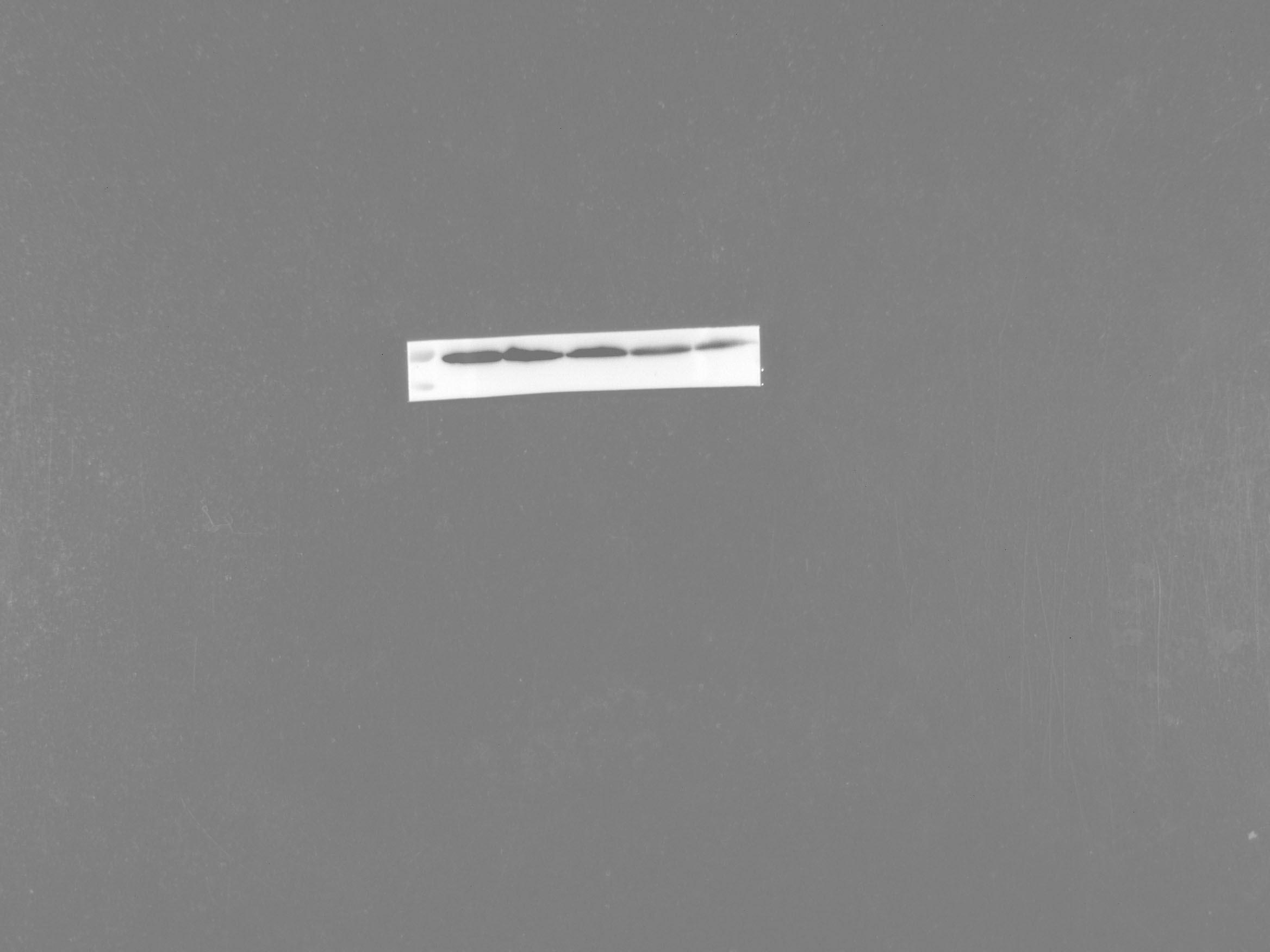

Supplement: Original Images for Blots.zip [file YRER_A_2313366_SM3875.zip › Original Images for Blots/Figure 6/Figure 6C/CyclinE/Marker+CyclinE.jpg]

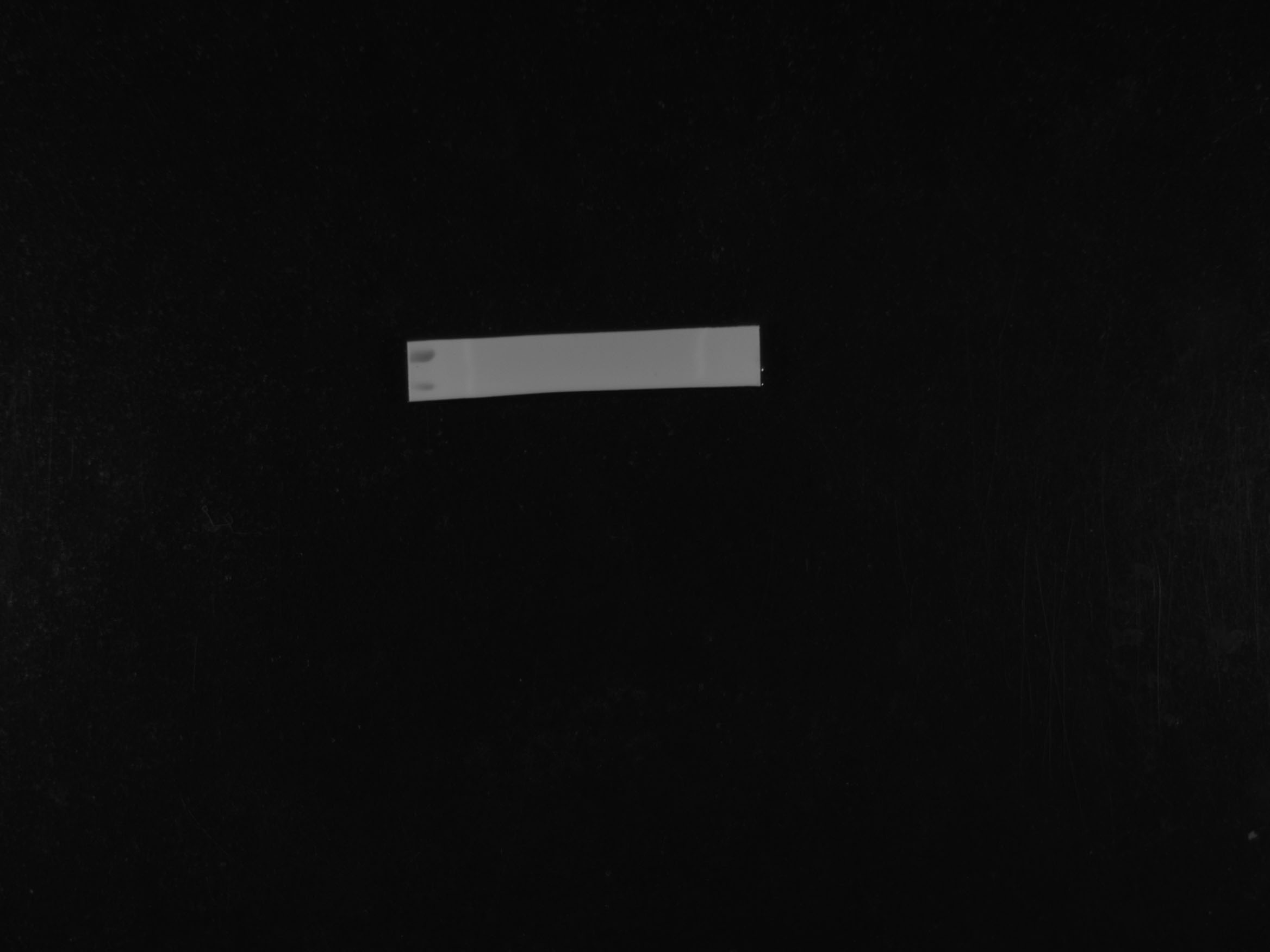

Supplement: Original Images for Blots.zip [file YRER_A_2313366_SM3875.zip › Original Images for Blots/Figure 6/Figure 6C/CyclinE/Marker.jpg]

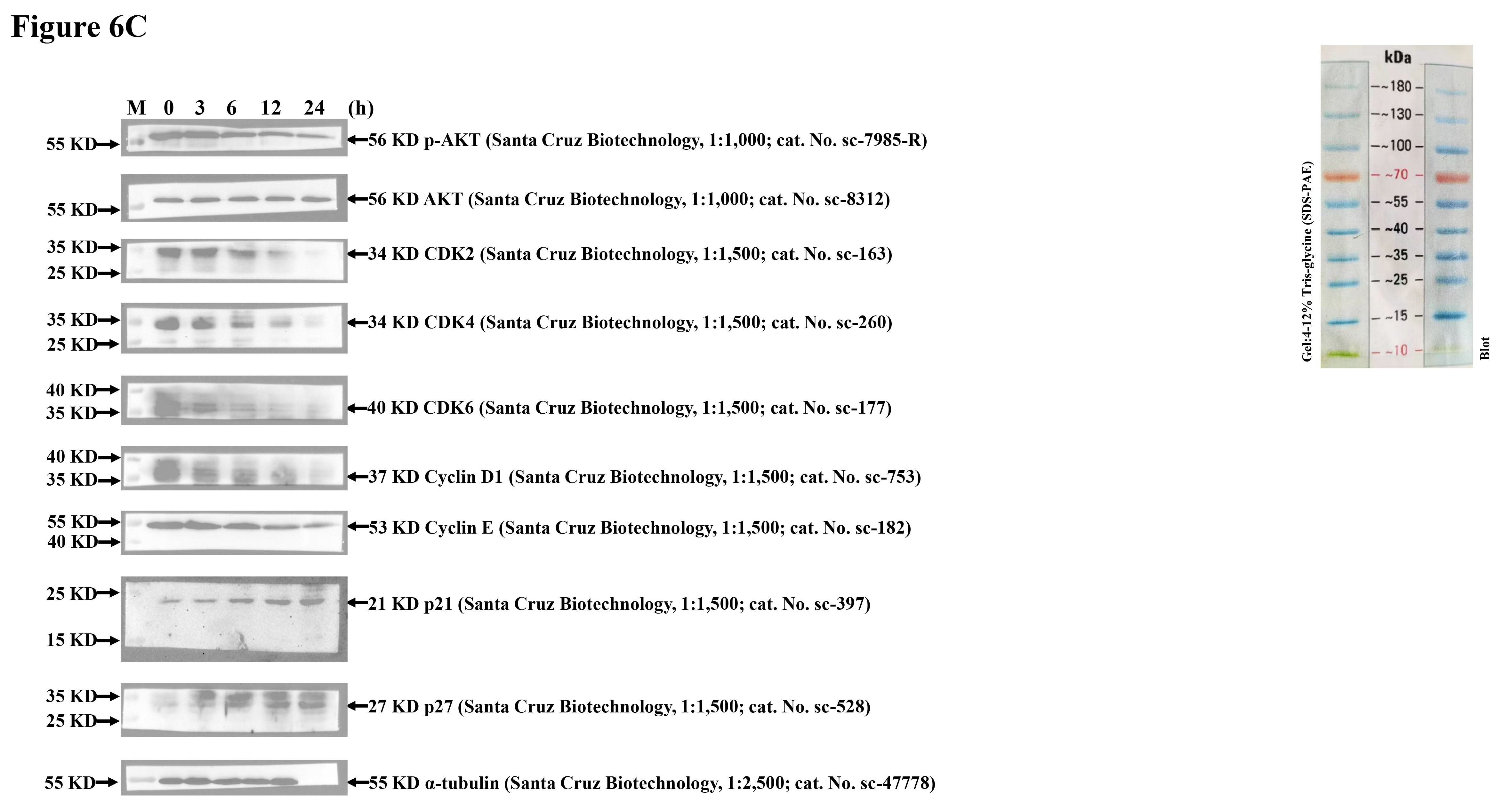

Supplement: Original Images for Blots.zip [file YRER_A_2313366_SM3875.zip › Original Images for Blots/Figure 6/Figure 6C/Figure 6C.jpg]

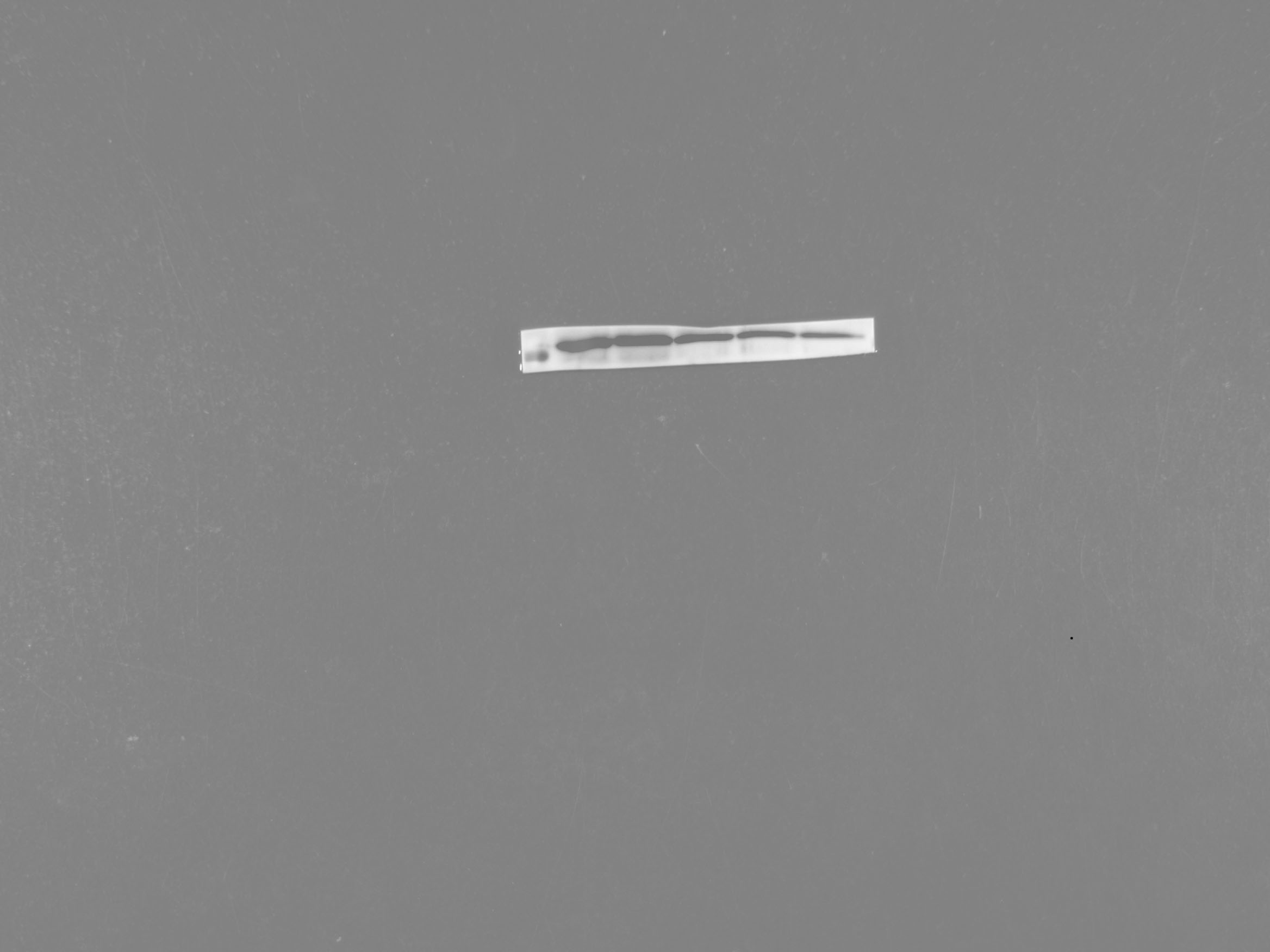

Supplement: Original Images for Blots.zip [file YRER_A_2313366_SM3875.zip › Original Images for Blots/Figure 6/Figure 6C/p-AKT/Marker+p-AKT.jpg]

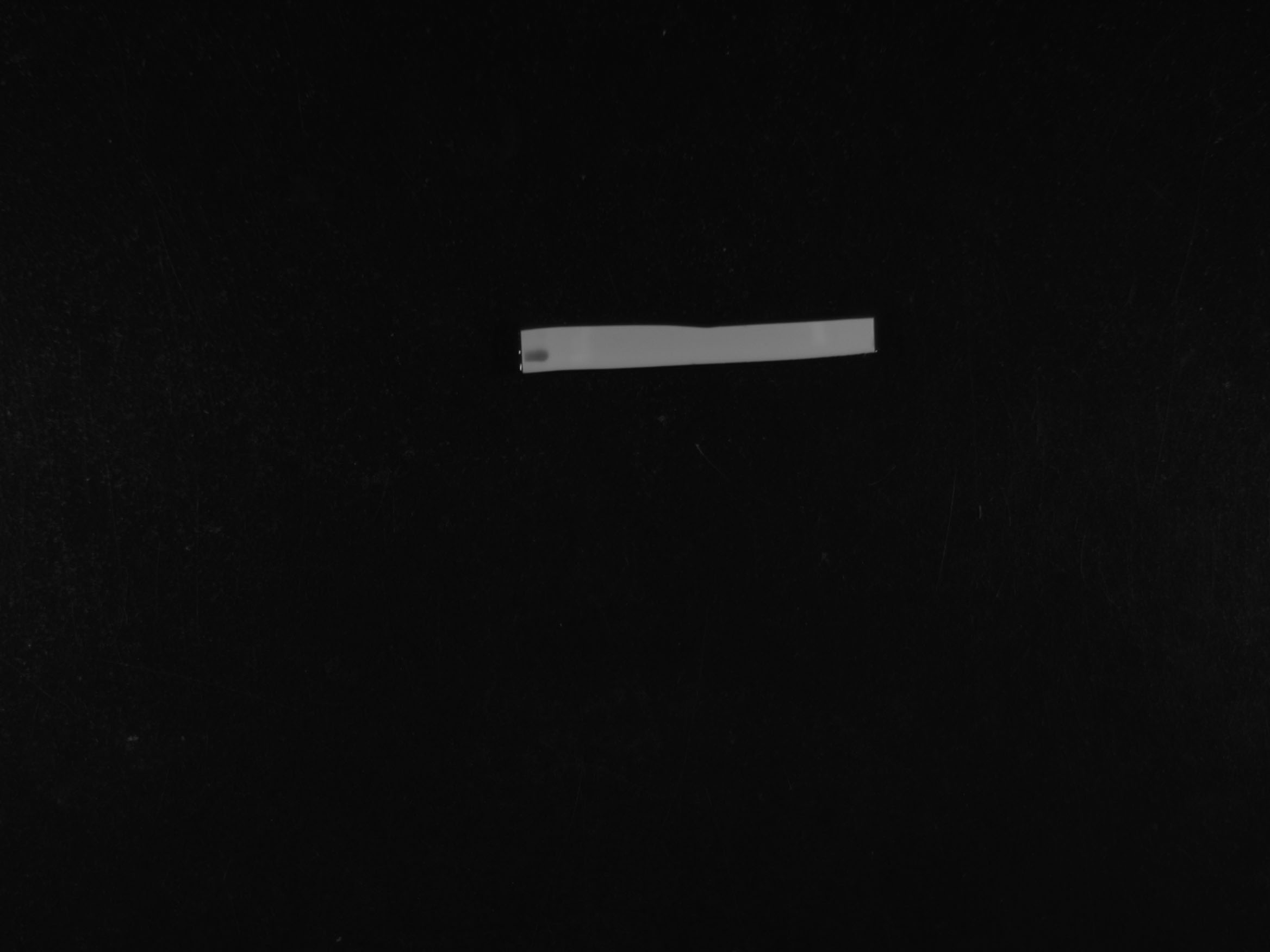

Supplement: Original Images for Blots.zip [file YRER_A_2313366_SM3875.zip › Original Images for Blots/Figure 6/Figure 6C/p-AKT/Marker.jpg]

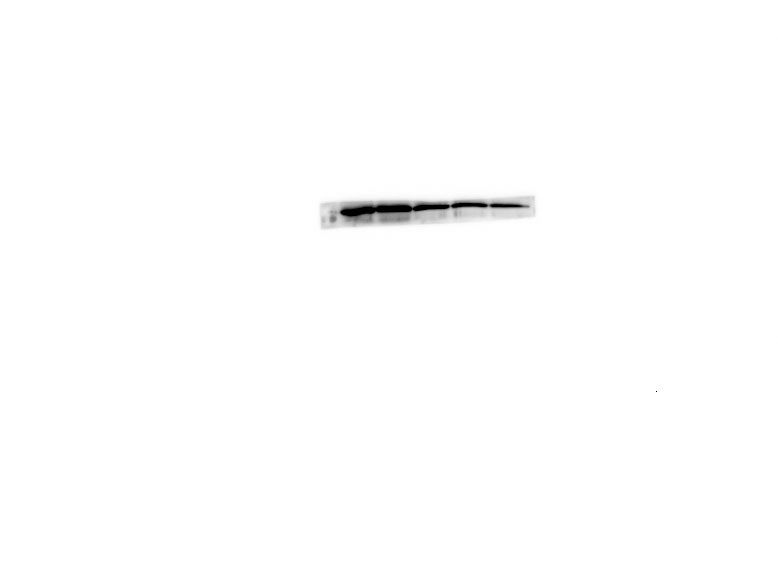

Supplement: Original Images for Blots.zip [file YRER_A_2313366_SM3875.zip › Original Images for Blots/Figure 6/Figure 6C/p-AKT/p-AKT.jpg]

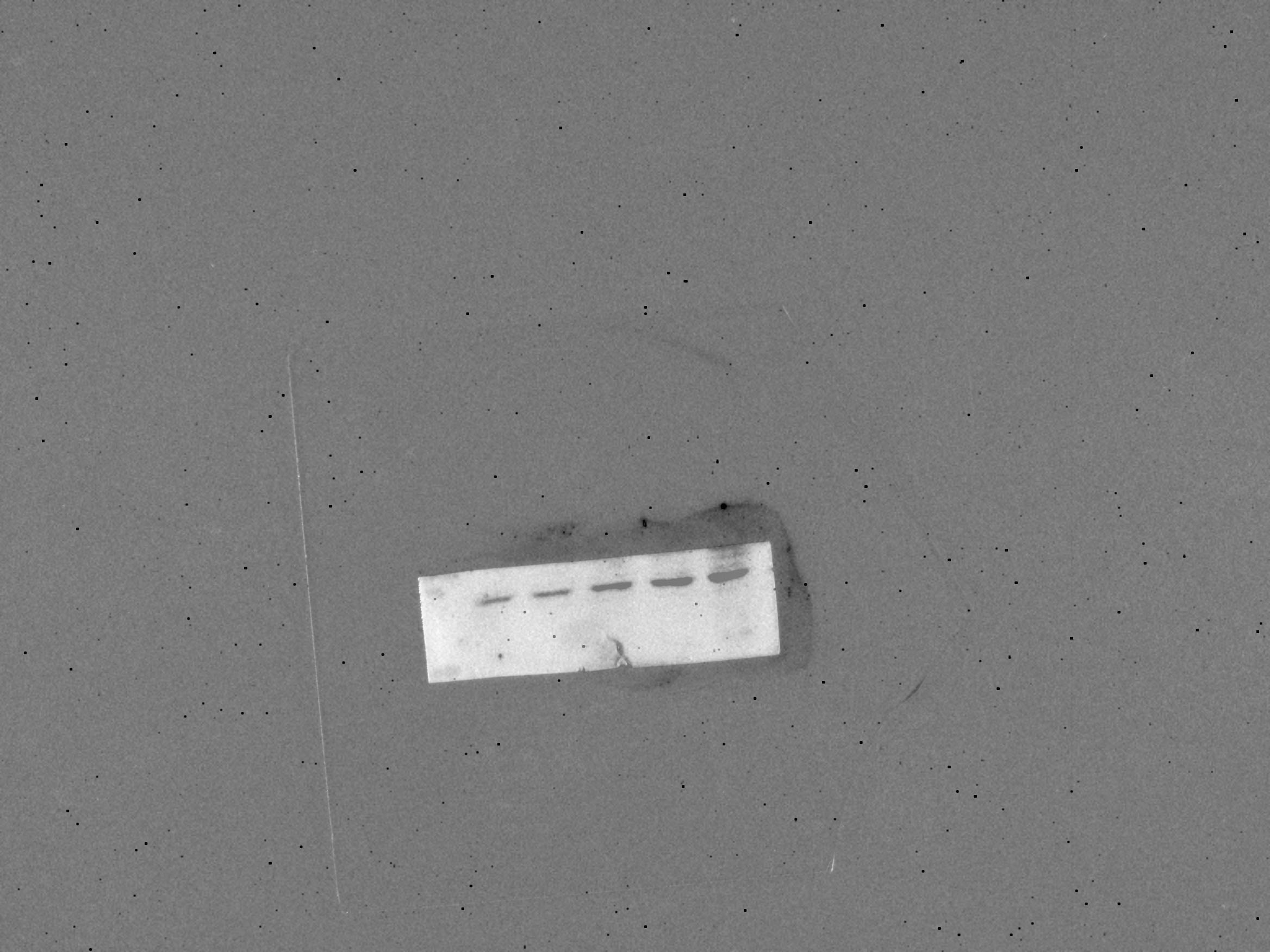

Supplement: Original Images for Blots.zip [file YRER_A_2313366_SM3875.zip › Original Images for Blots/Figure 6/Figure 6C/P21/Marker+p21.jpg]

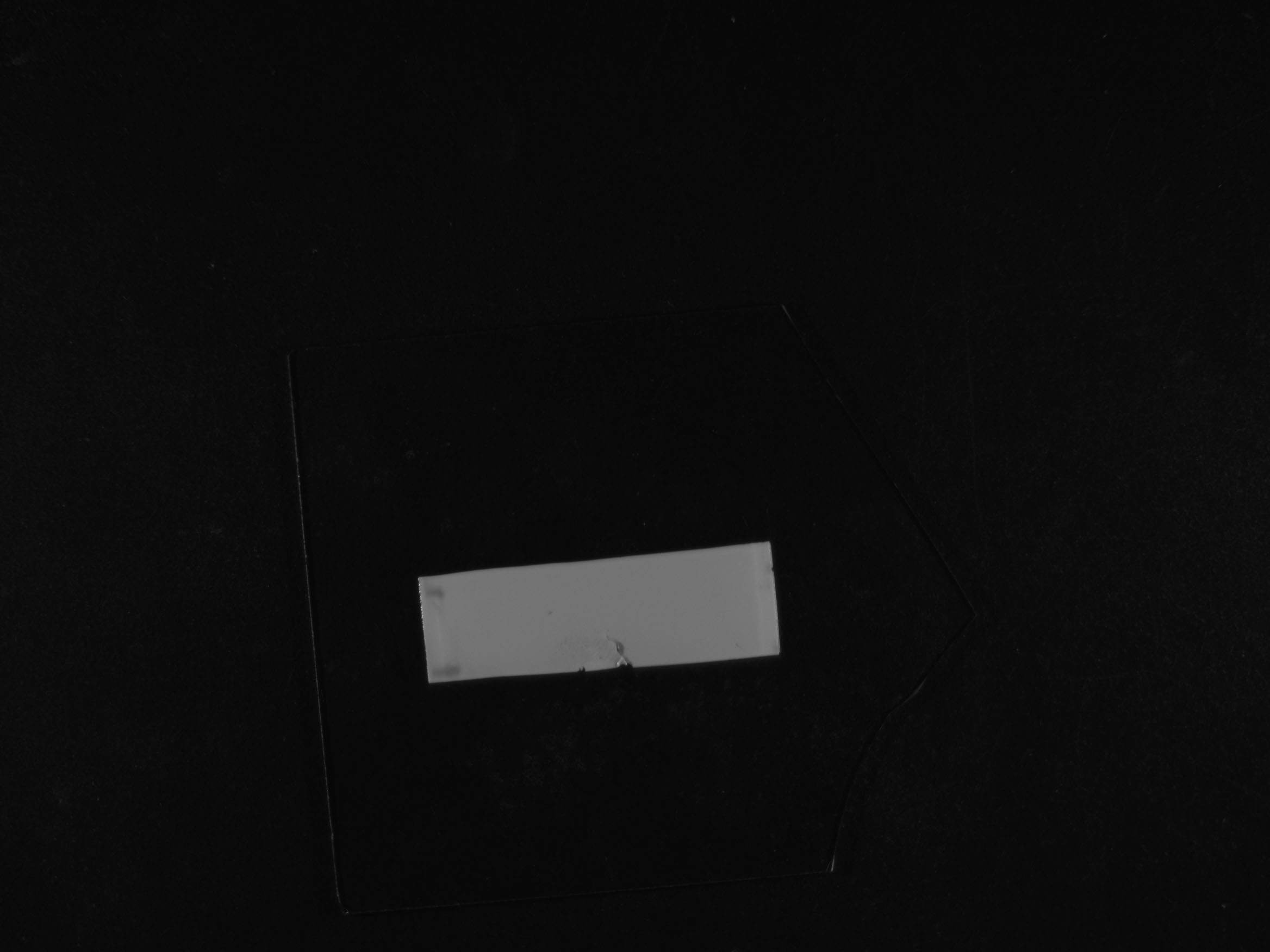

Supplement: Original Images for Blots.zip [file YRER_A_2313366_SM3875.zip › Original Images for Blots/Figure 6/Figure 6C/P21/Marker.jpg]

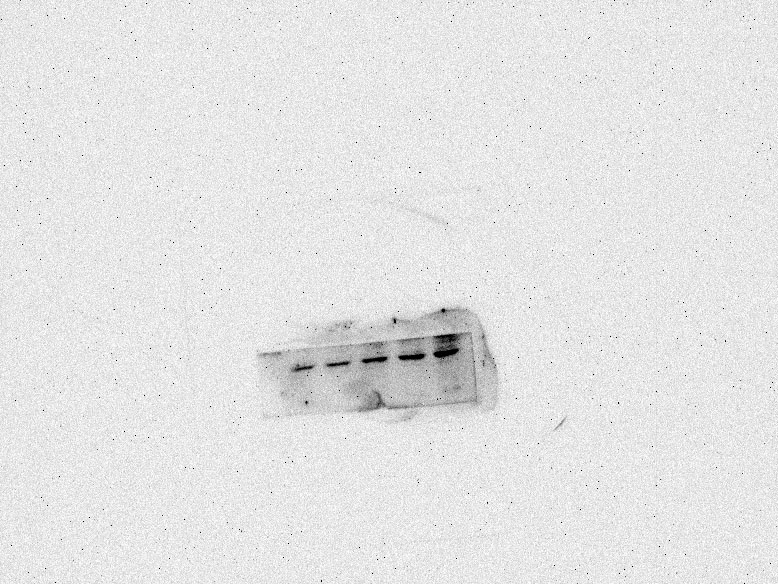

Supplement: Original Images for Blots.zip [file YRER_A_2313366_SM3875.zip › Original Images for Blots/Figure 6/Figure 6C/P21/p21.jpg]

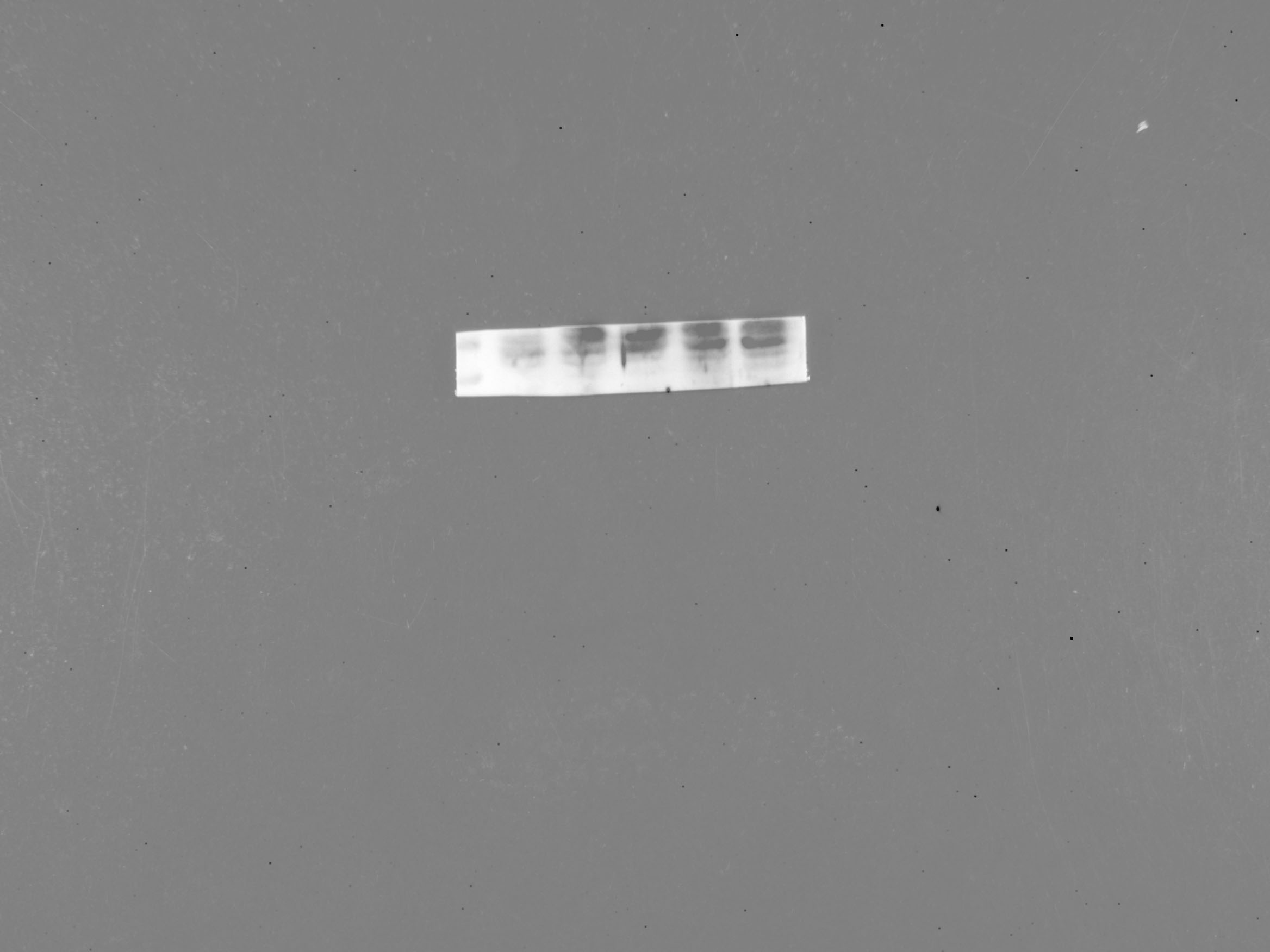

Supplement: Original Images for Blots.zip [file YRER_A_2313366_SM3875.zip › Original Images for Blots/Figure 6/Figure 6C/p27/Marker+p27.jpg]
