# Supplementary figures and images for: Jaceosidin induces apoptosis and inhibits migration in AGS gastric cancer cells by regulating ROS-mediated signaling pathways (part 1 of 2)
Source: Redox Rep. 2024 Feb 6;29(1):2313366. doi: 10.1080/13510002.2024.2313366 (PMC10854459; doi:10.1080/13510002.2024.2313366)

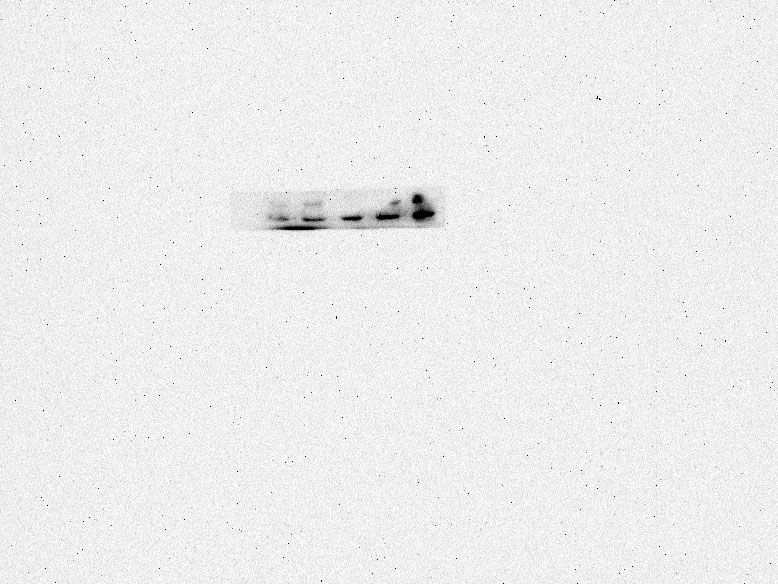

Supplement: Original Images for Blots.zip [file YRER_A_2313366_SM3875.zip › Original Images for Blots/Figure 2/Figure 2E/Bad/Bad.jpg]

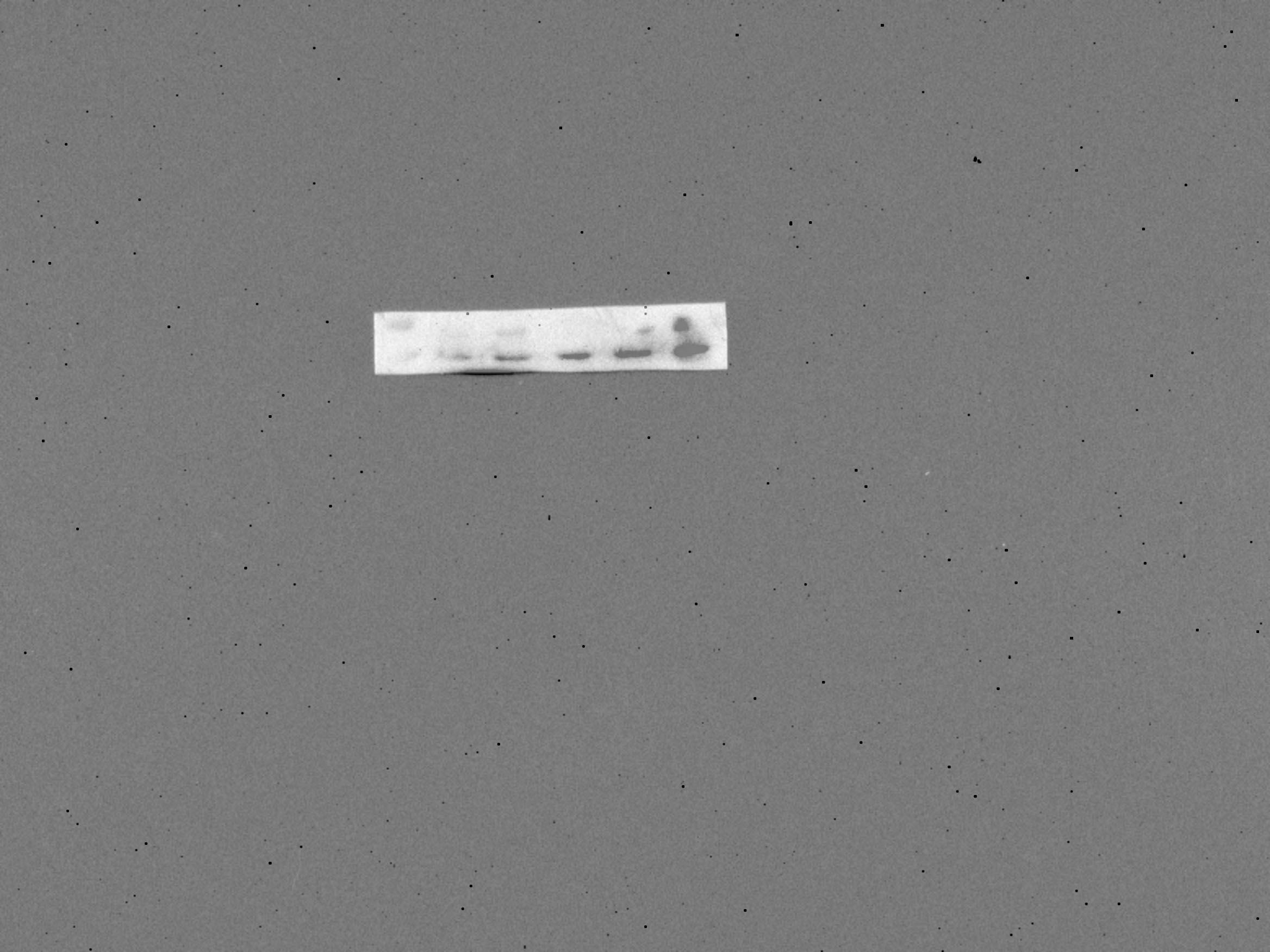

Supplement: Original Images for Blots.zip [file YRER_A_2313366_SM3875.zip › Original Images for Blots/Figure 2/Figure 2E/Bad/Marker+Bad.jpg]

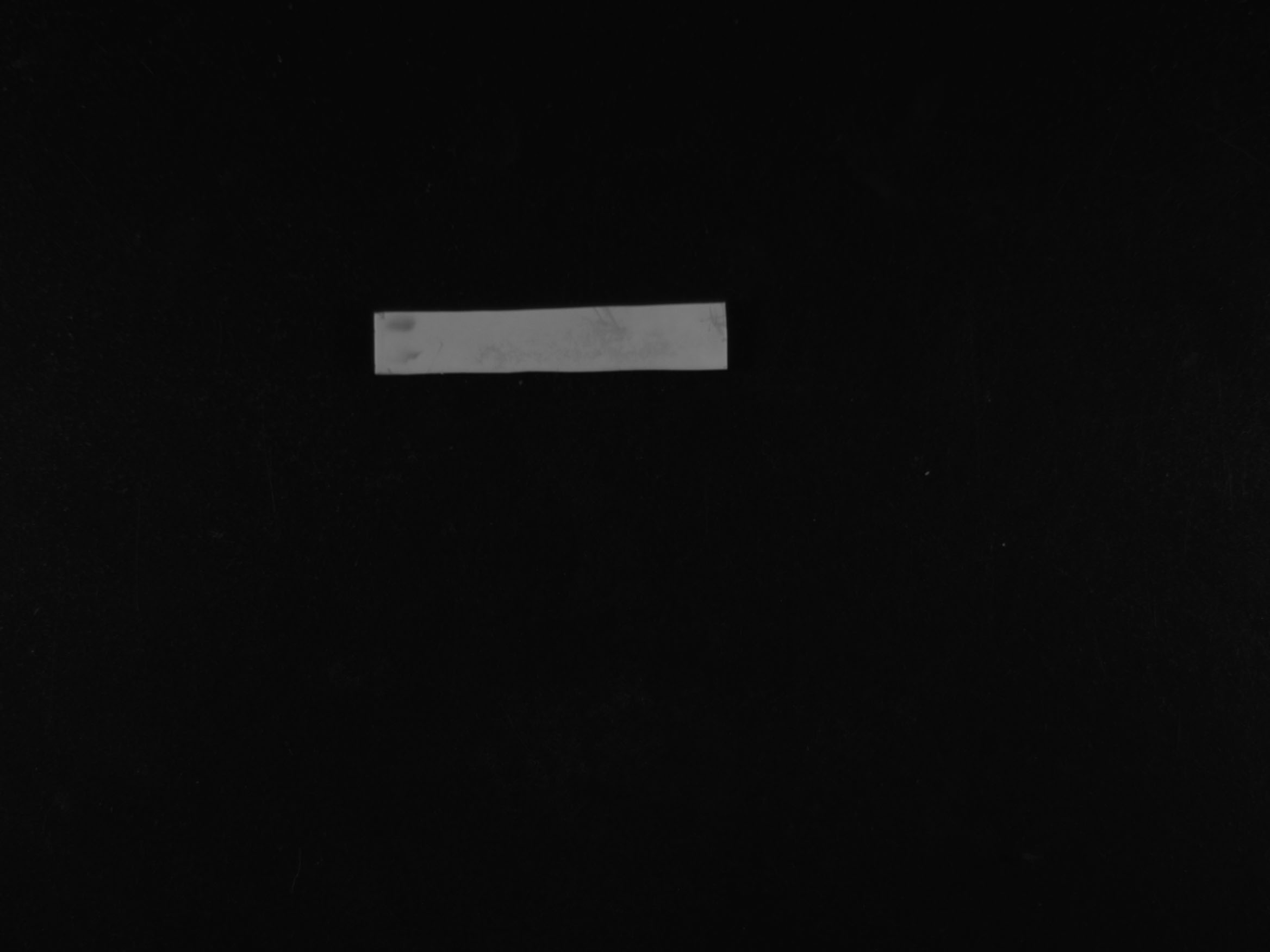

Supplement: Original Images for Blots.zip [file YRER_A_2313366_SM3875.zip › Original Images for Blots/Figure 2/Figure 2E/Bad/Marker.jpg]

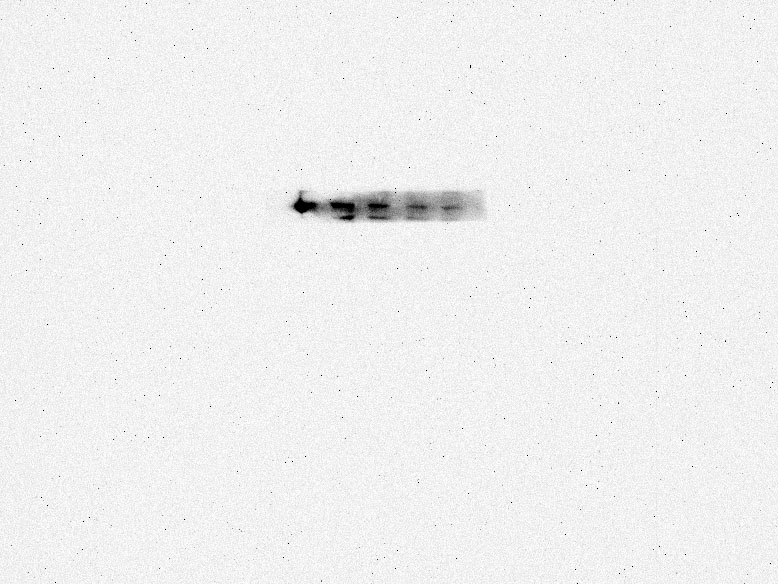

Supplement: Original Images for Blots.zip [file YRER_A_2313366_SM3875.zip › Original Images for Blots/Figure 2/Figure 2E/Bcl-2/Bcl-2.jpg]

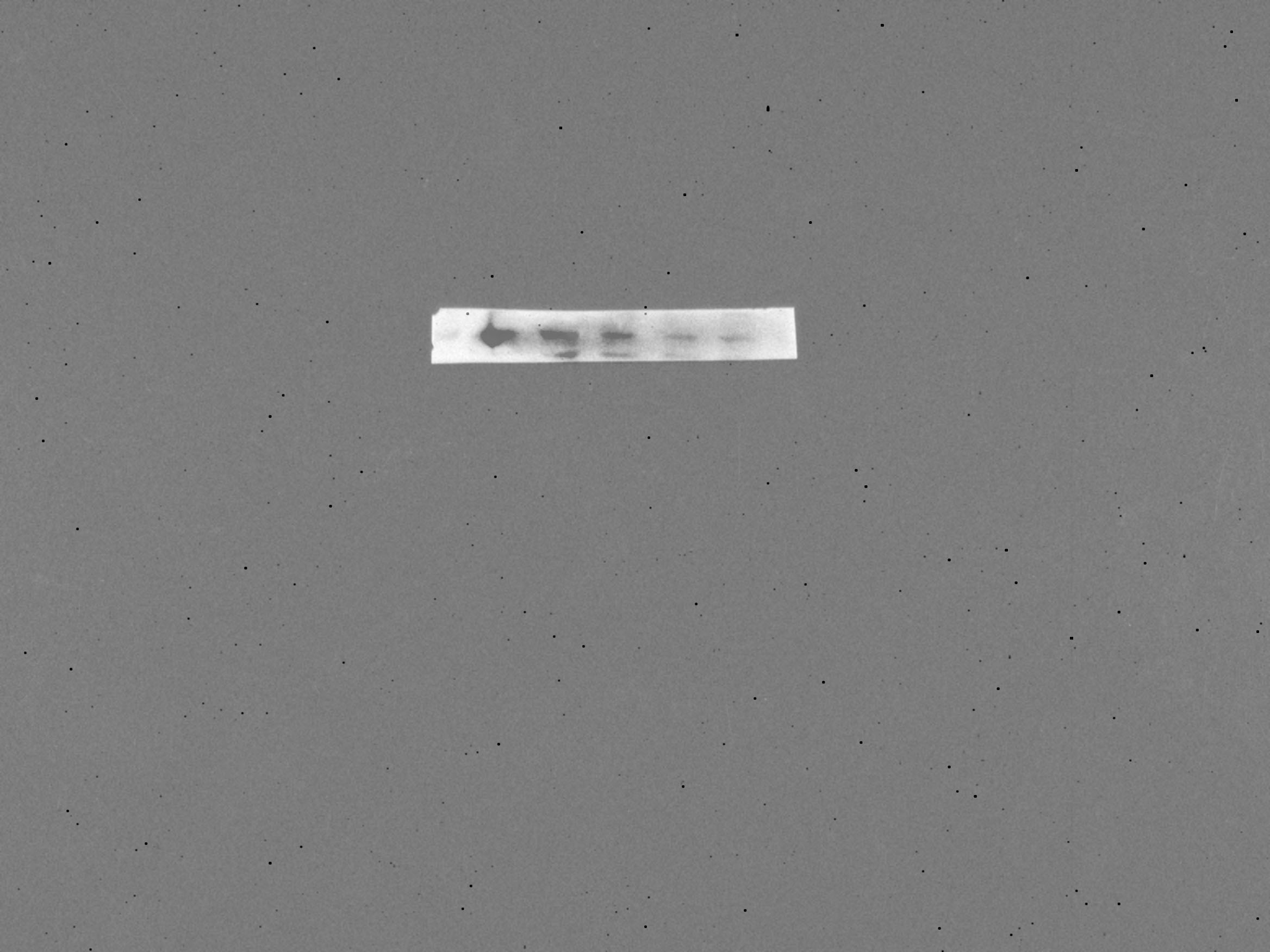

Supplement: Original Images for Blots.zip [file YRER_A_2313366_SM3875.zip › Original Images for Blots/Figure 2/Figure 2E/Bcl-2/Marker+Bcl-2.jpg]

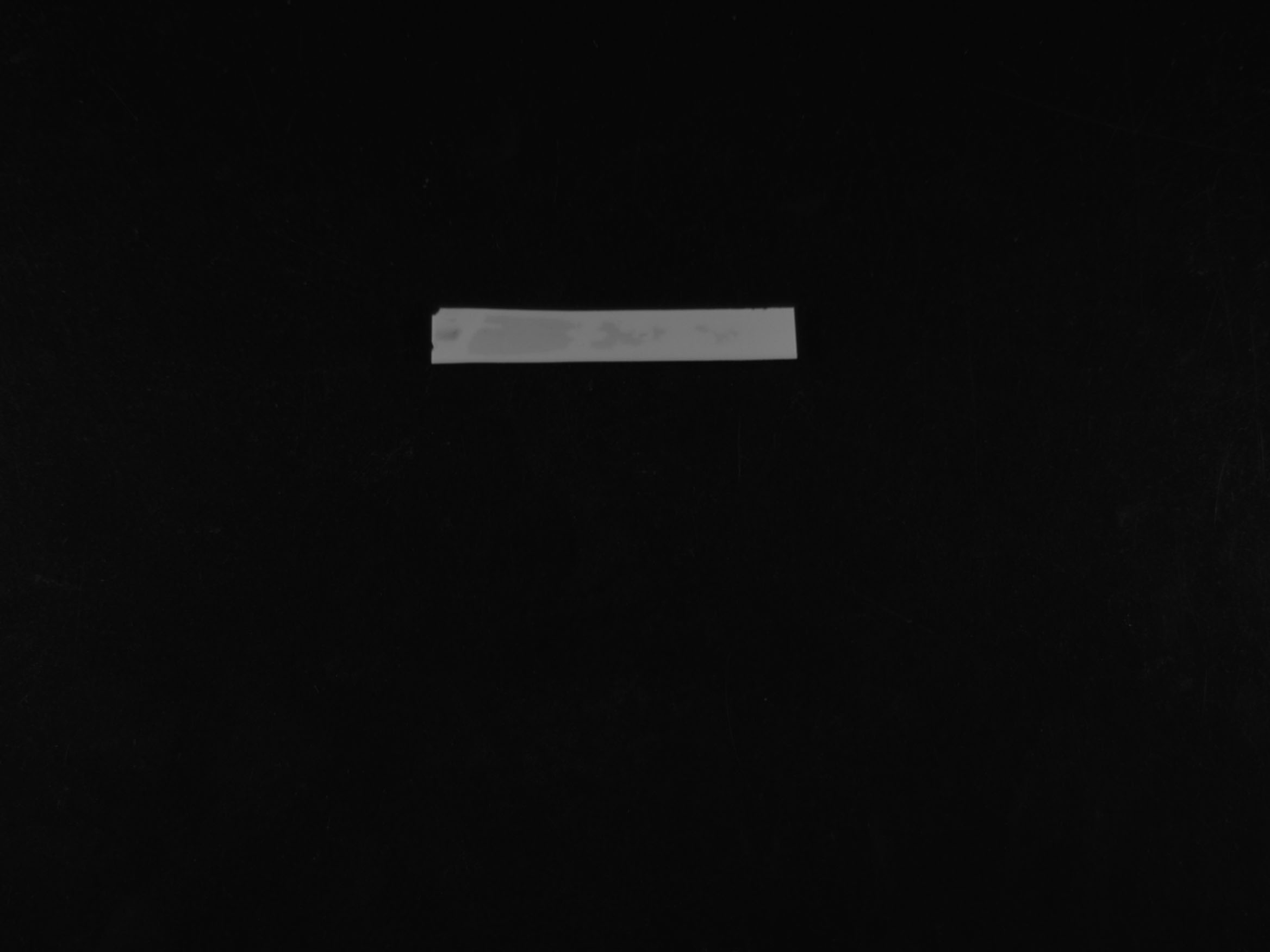

Supplement: Original Images for Blots.zip [file YRER_A_2313366_SM3875.zip › Original Images for Blots/Figure 2/Figure 2E/Bcl-2/Marker.jpg]

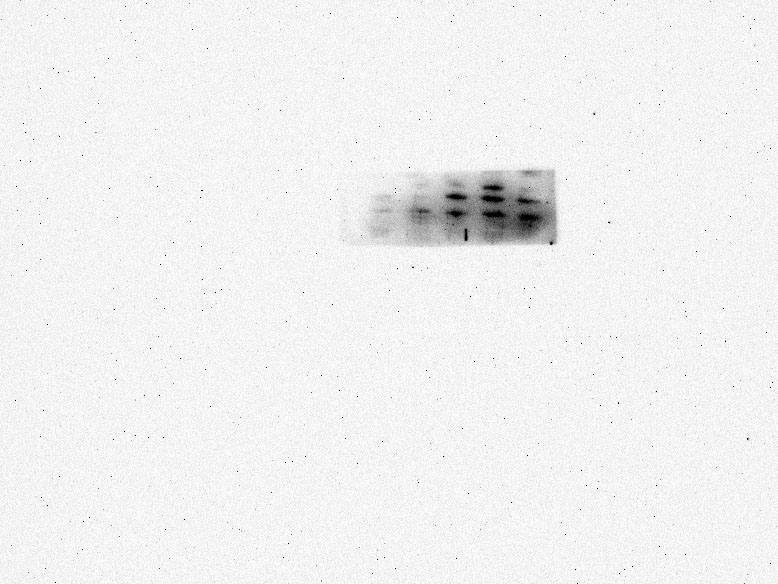

Supplement: Original Images for Blots.zip [file YRER_A_2313366_SM3875.zip › Original Images for Blots/Figure 2/Figure 2E/cle-caspase-3/cle-caspase-3.jpg]

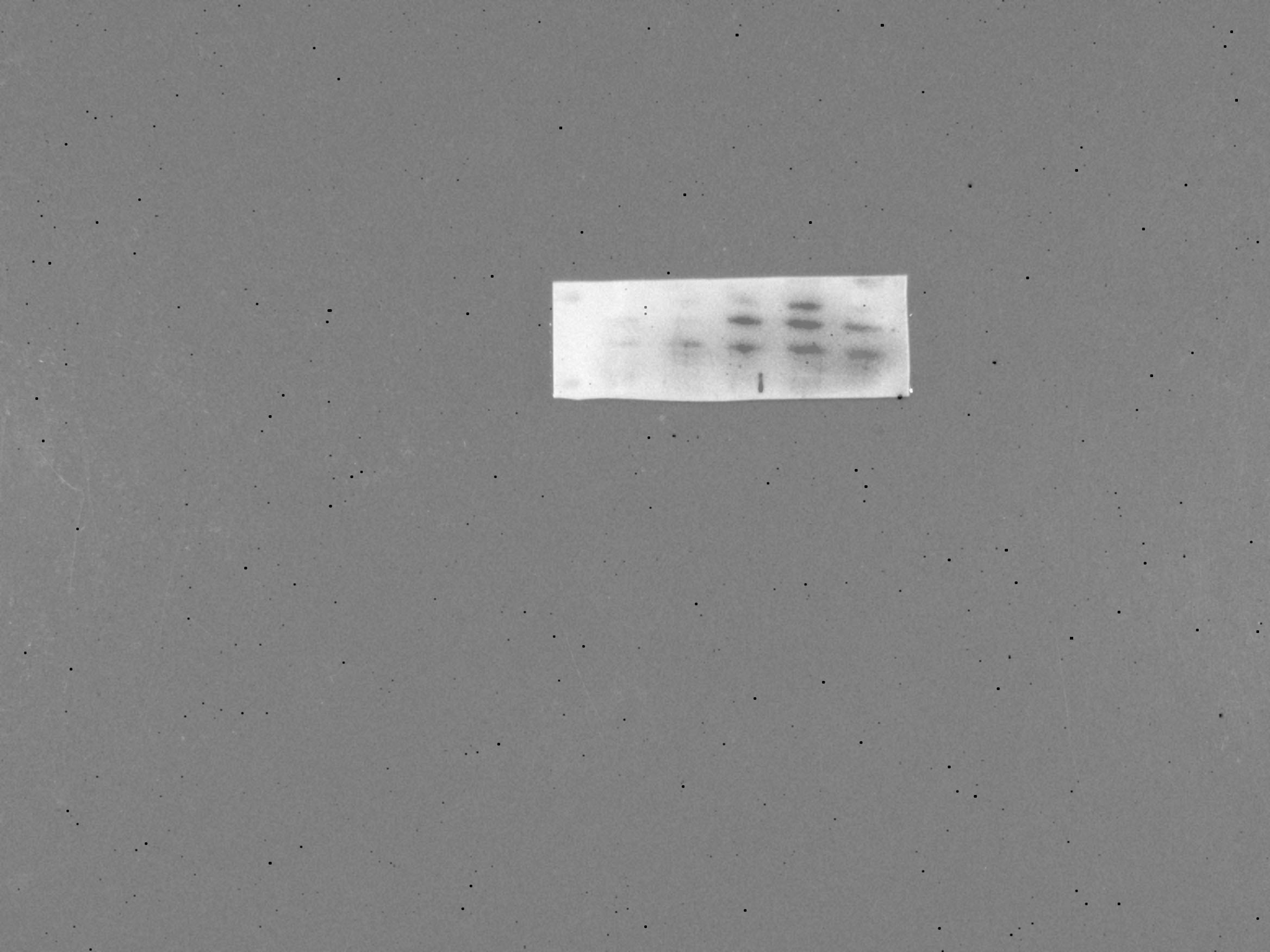

Supplement: Original Images for Blots.zip [file YRER_A_2313366_SM3875.zip › Original Images for Blots/Figure 2/Figure 2E/cle-caspase-3/Marker+cle-caspase-3.jpg]

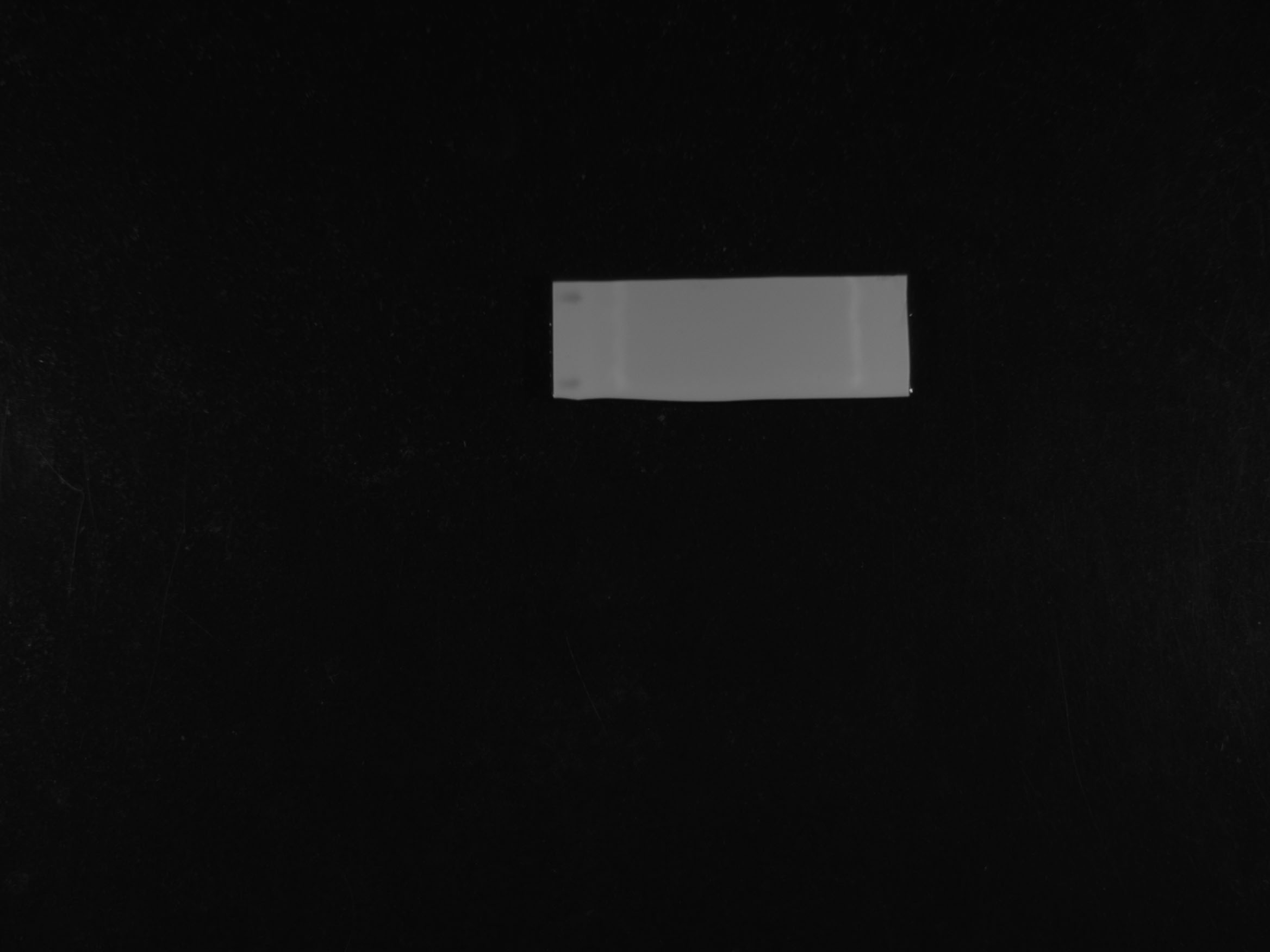

Supplement: Original Images for Blots.zip [file YRER_A_2313366_SM3875.zip › Original Images for Blots/Figure 2/Figure 2E/cle-caspase-3/Marker.jpg]

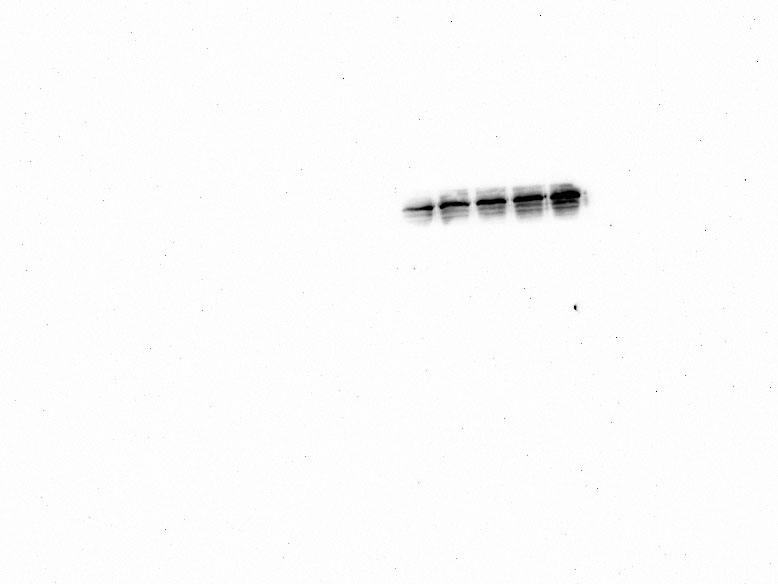

Supplement: Original Images for Blots.zip [file YRER_A_2313366_SM3875.zip › Original Images for Blots/Figure 2/Figure 2E/cle-PARP/cle-PARP.jpg]

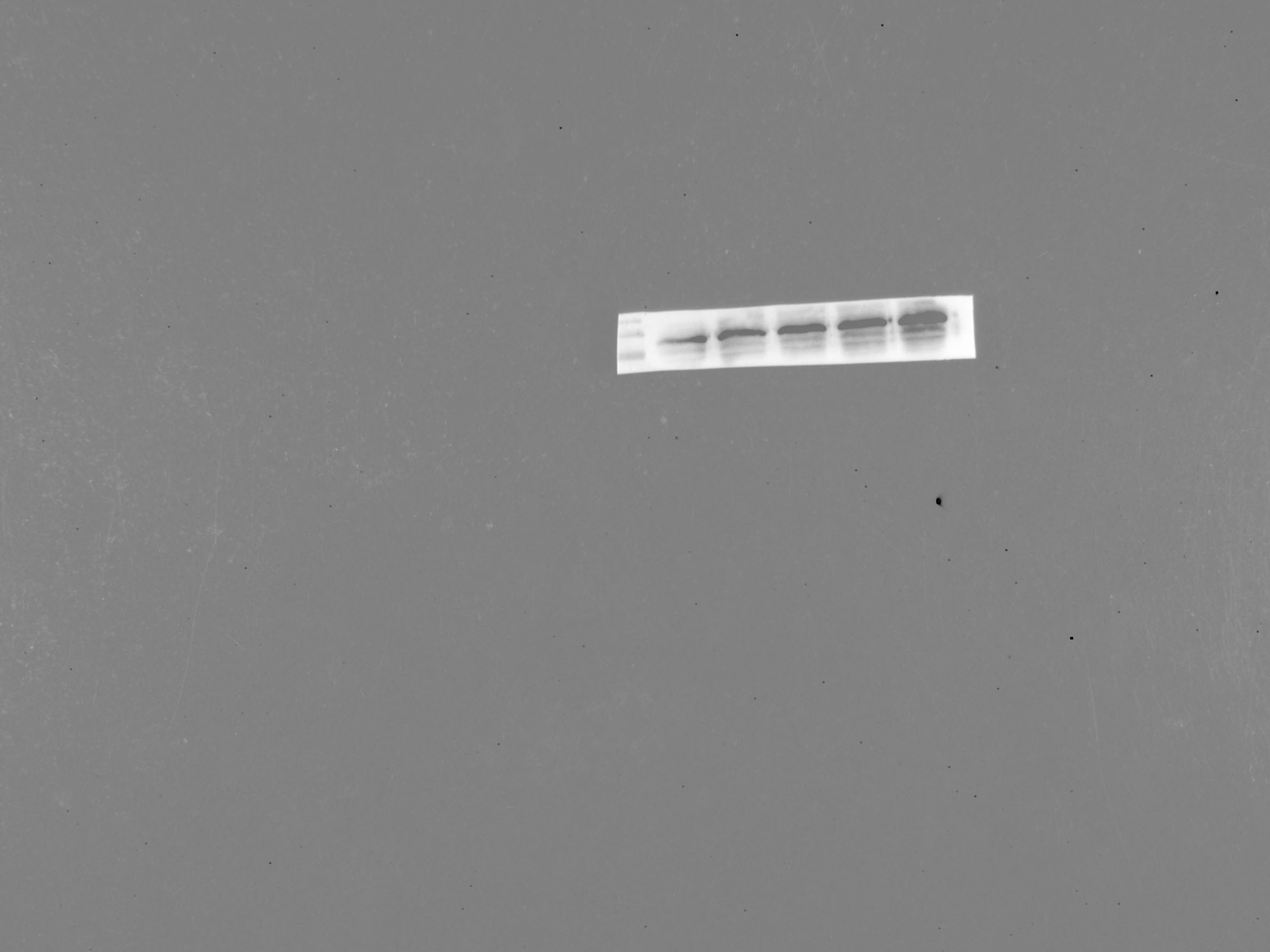

Supplement: Original Images for Blots.zip [file YRER_A_2313366_SM3875.zip › Original Images for Blots/Figure 2/Figure 2E/cle-PARP/Marker+cle-PARP.jpg]

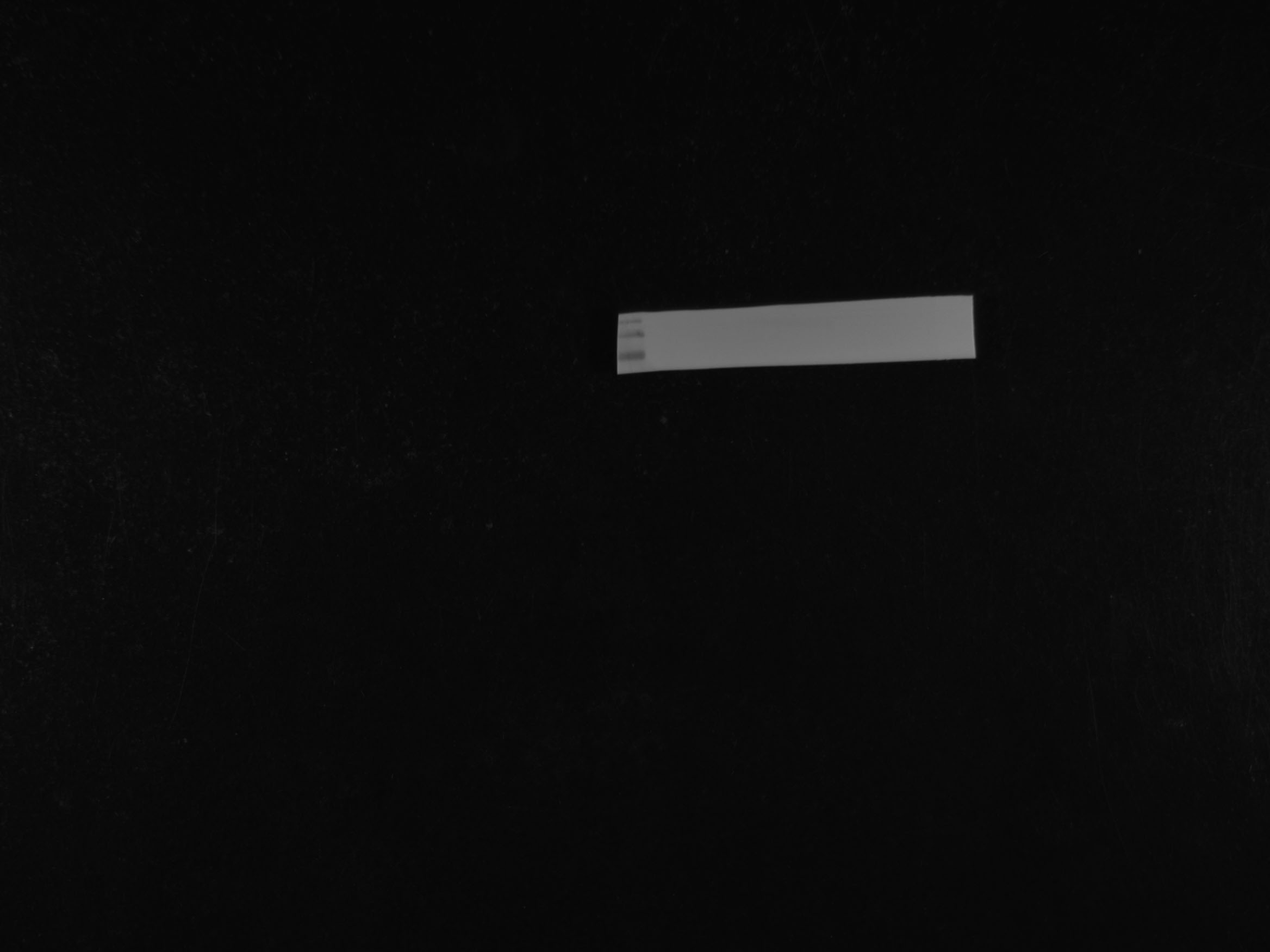

Supplement: Original Images for Blots.zip [file YRER_A_2313366_SM3875.zip › Original Images for Blots/Figure 2/Figure 2E/cle-PARP/Marker.jpg]

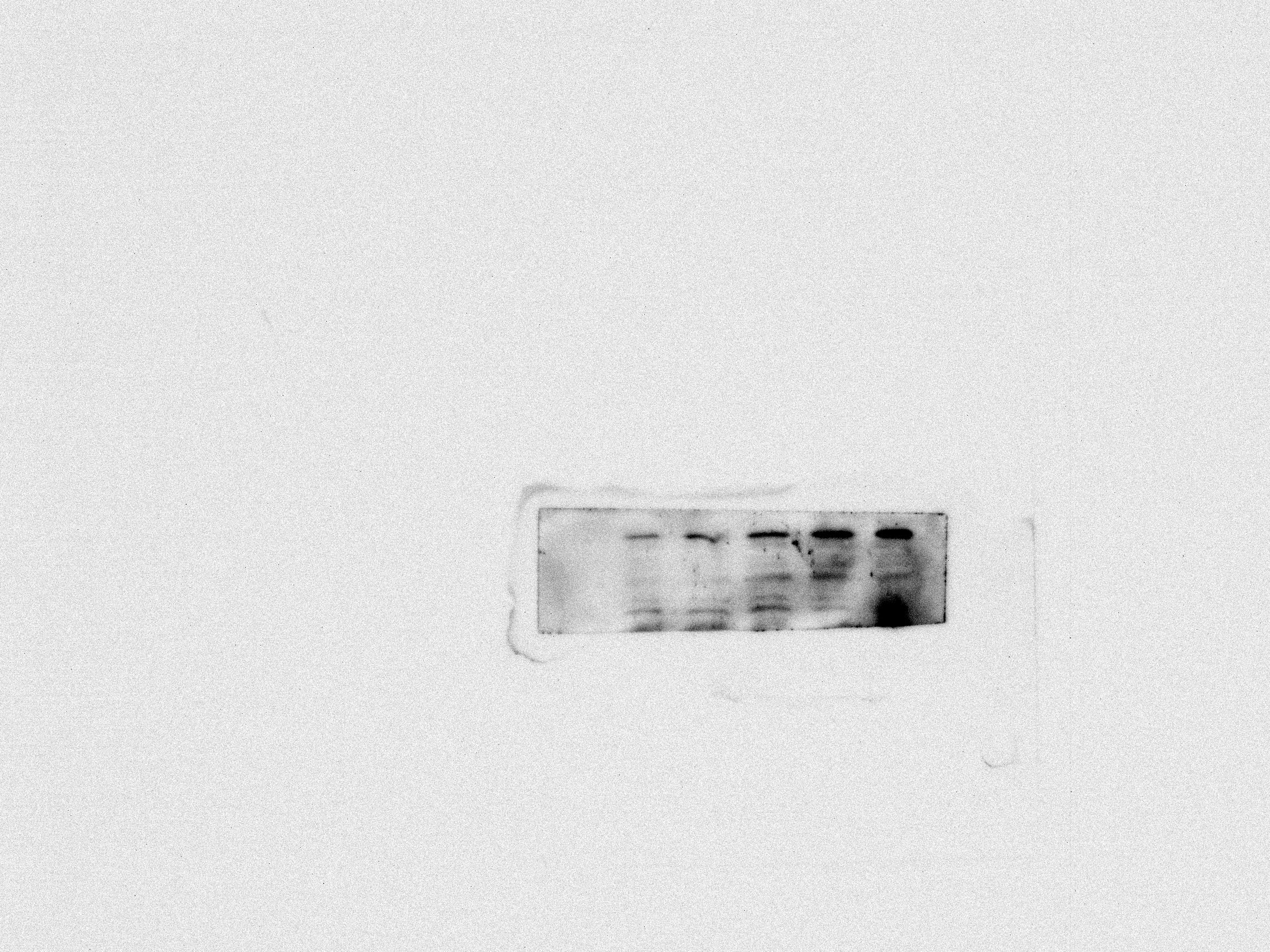

Supplement: Original Images for Blots.zip [file YRER_A_2313366_SM3875.zip › Original Images for Blots/Figure 2/Figure 2E/Cyto-c/Cyto-c.jpg]

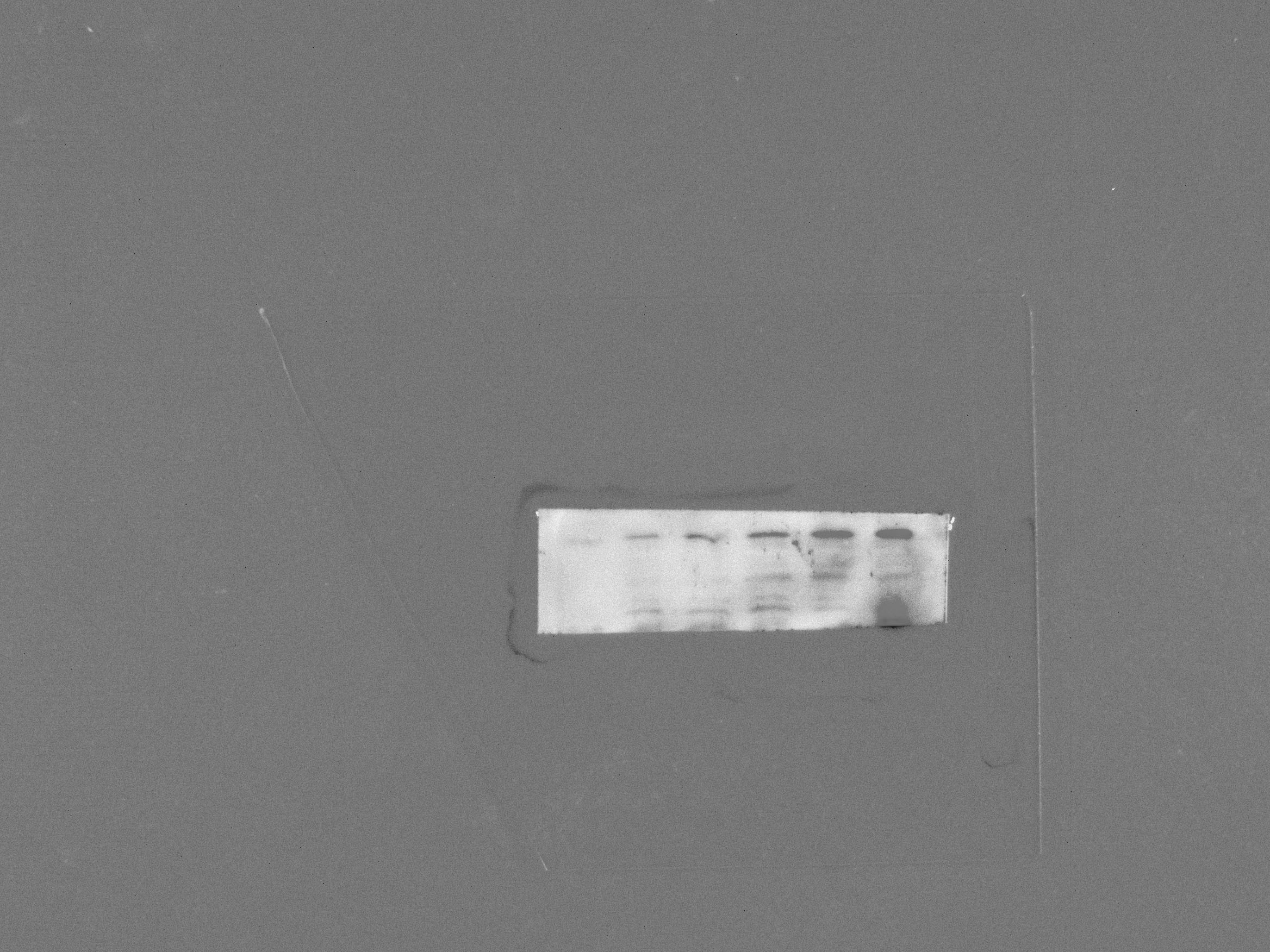

Supplement: Original Images for Blots.zip [file YRER_A_2313366_SM3875.zip › Original Images for Blots/Figure 2/Figure 2E/Cyto-c/Marker+Cyto-c.jpg]

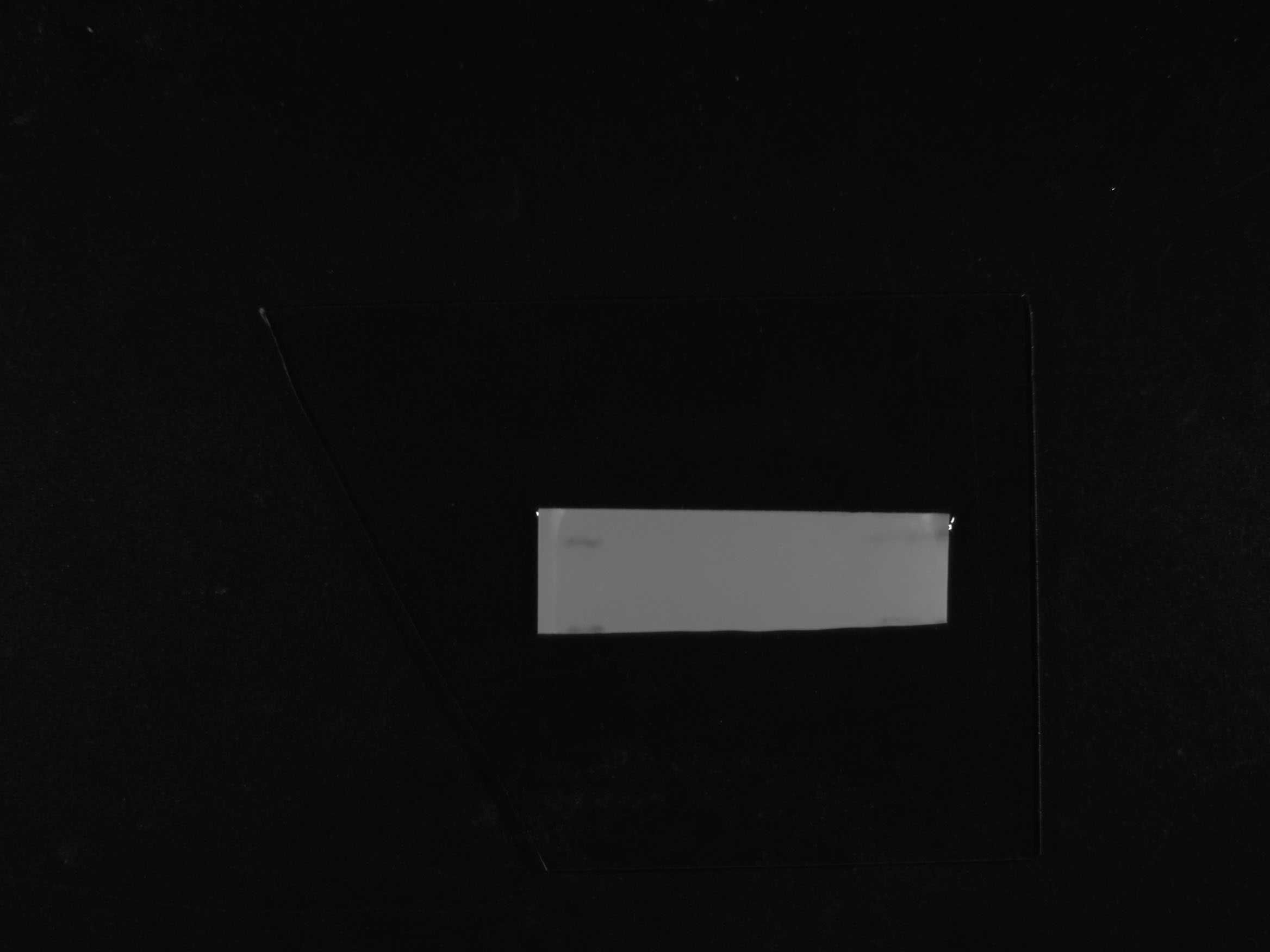

Supplement: Original Images for Blots.zip [file YRER_A_2313366_SM3875.zip › Original Images for Blots/Figure 2/Figure 2E/Cyto-c/Marker.jpg]

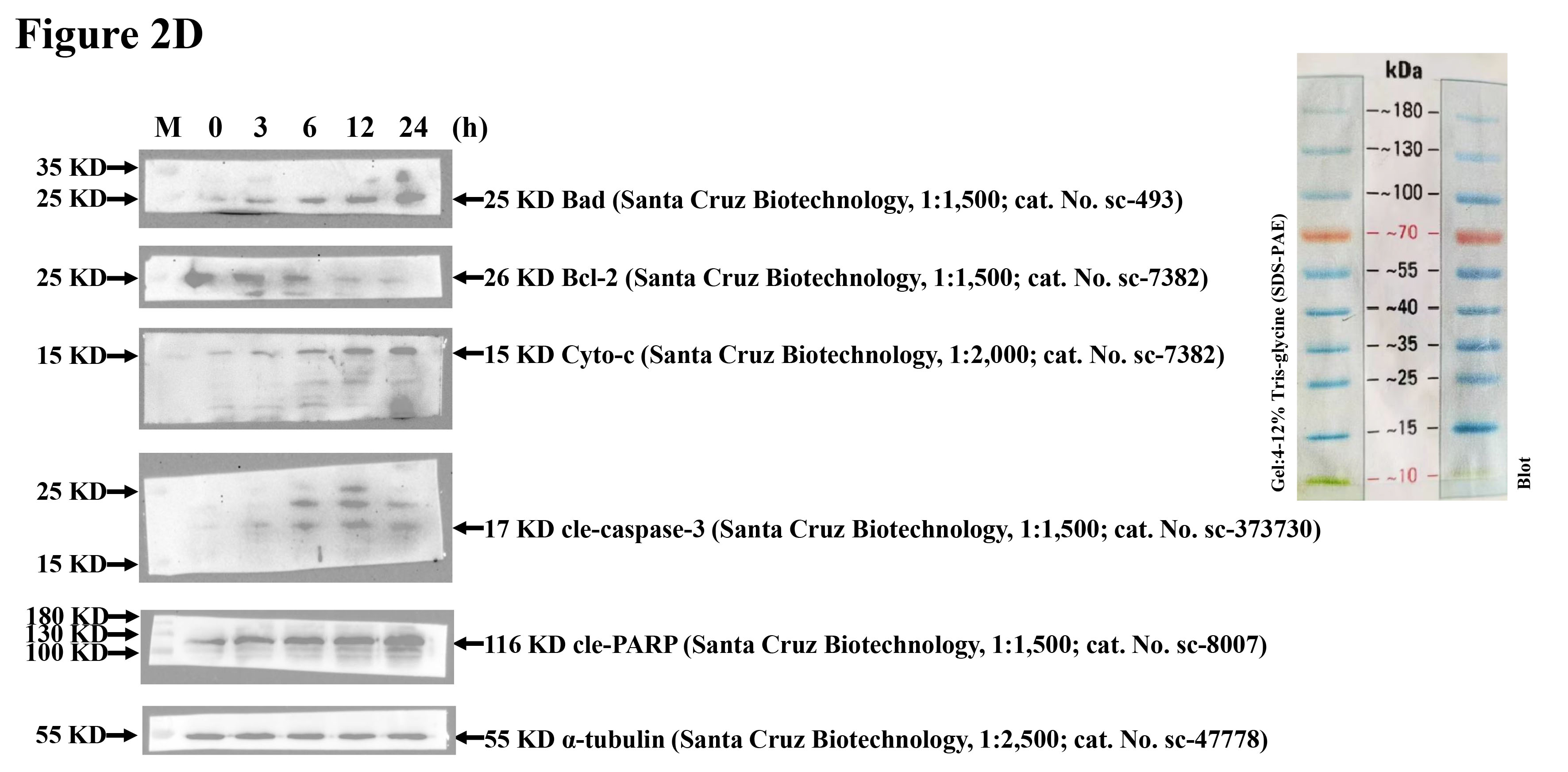

Supplement: Original Images for Blots.zip [file YRER_A_2313366_SM3875.zip › Original Images for Blots/Figure 2/Figure 2E/Figure 2E.jpg]

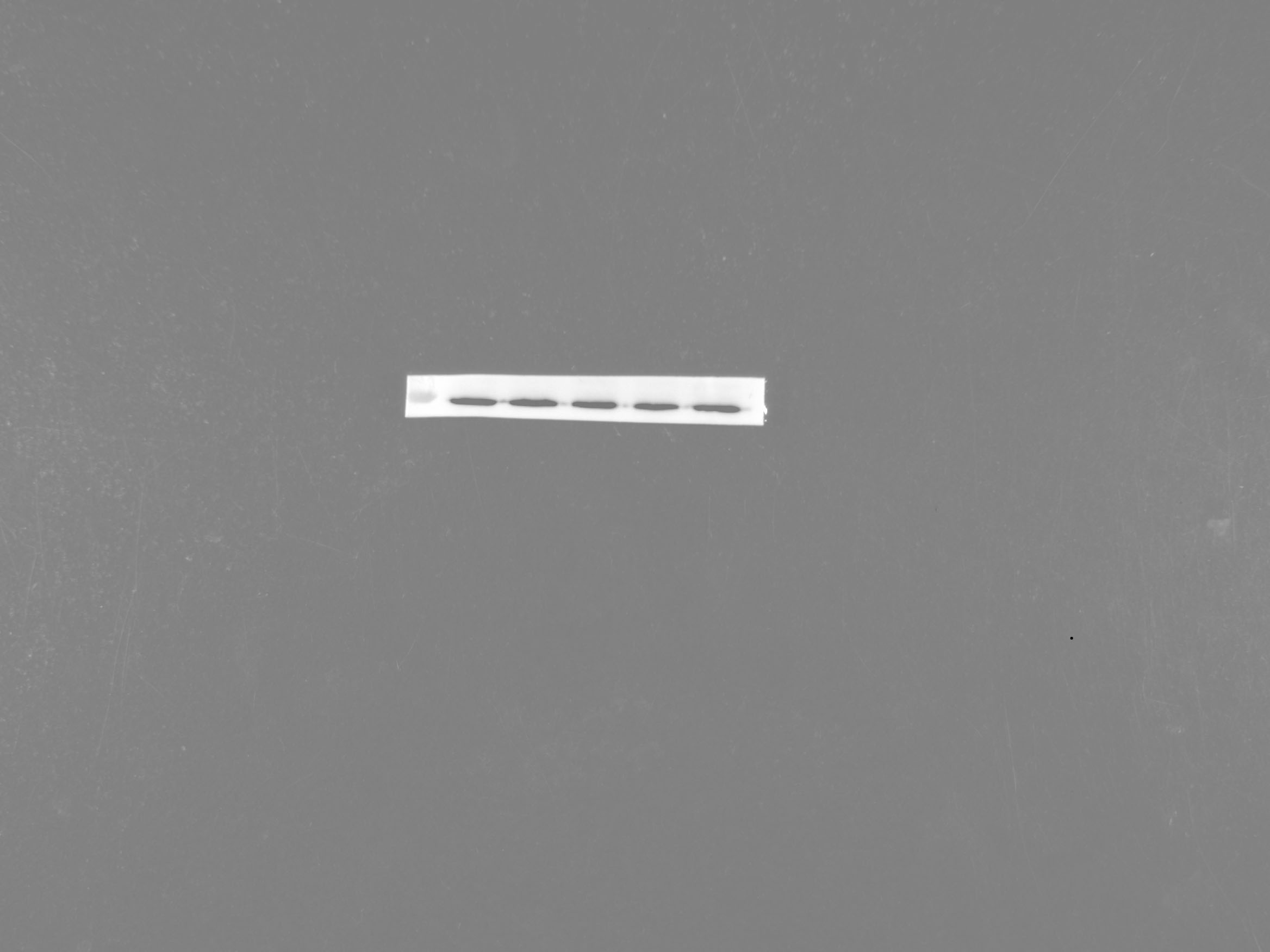

Supplement: Original Images for Blots.zip [file YRER_A_2313366_SM3875.zip › Original Images for Blots/Figure 2/Figure 2E/α-tubulin/Marker+α-tubulin.jpg]

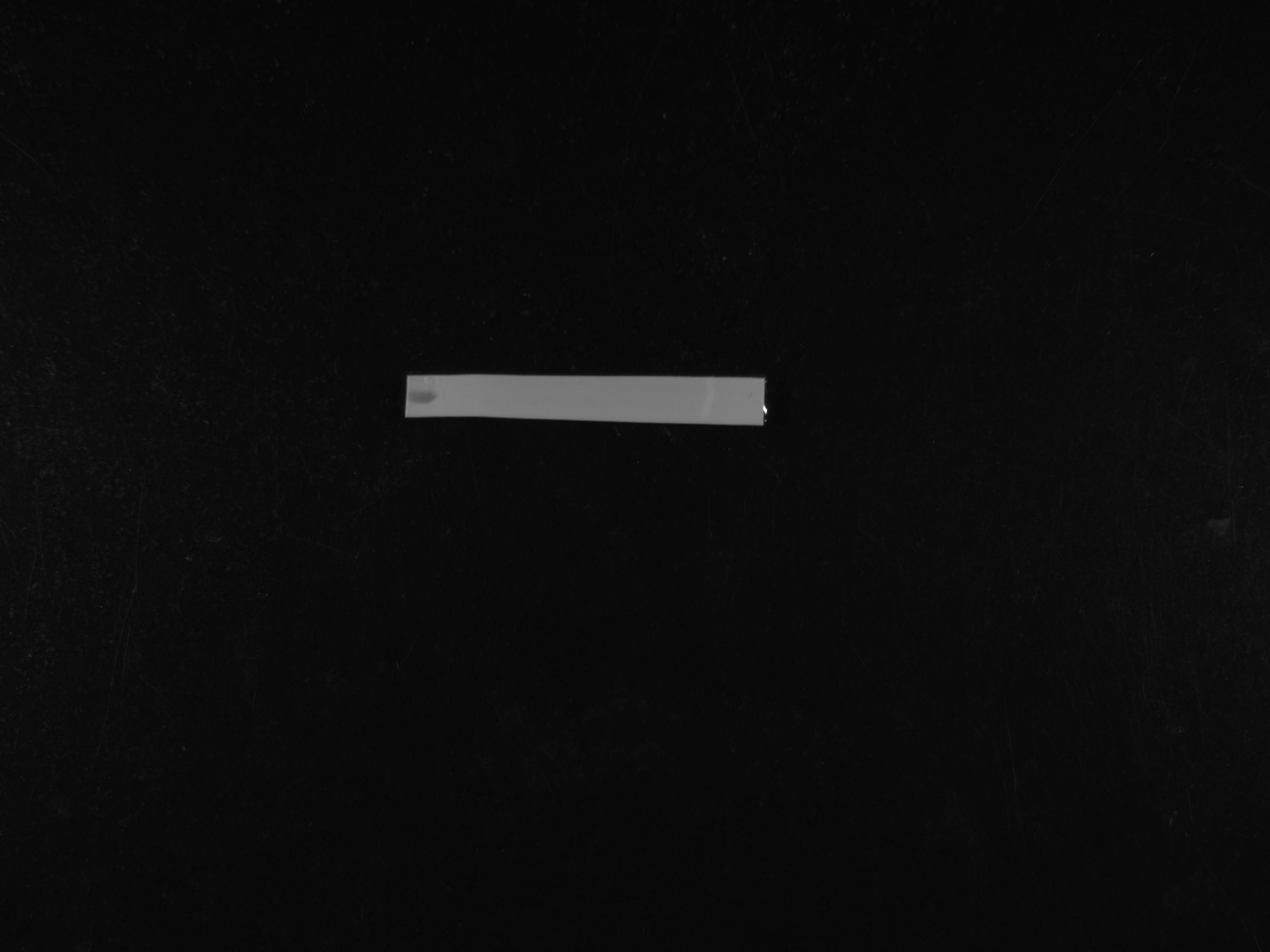

Supplement: Original Images for Blots.zip [file YRER_A_2313366_SM3875.zip › Original Images for Blots/Figure 2/Figure 2E/α-tubulin/Marker.jpg]

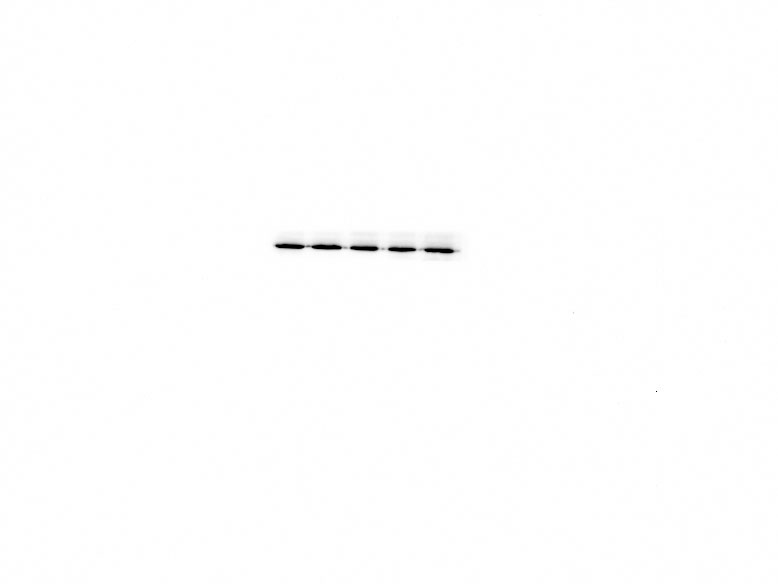

Supplement: Original Images for Blots.zip [file YRER_A_2313366_SM3875.zip › Original Images for Blots/Figure 2/Figure 2E/α-tubulin/α-tubulin.jpg]

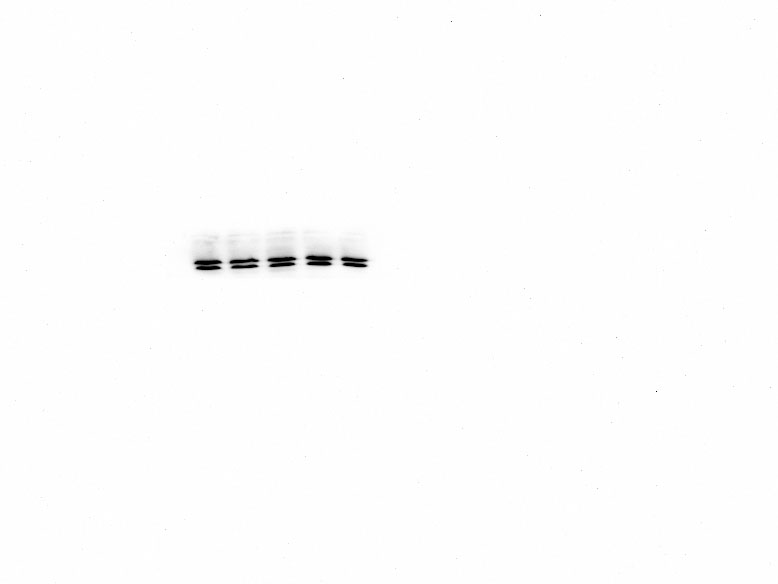

Supplement: Original Images for Blots.zip [file YRER_A_2313366_SM3875.zip › Original Images for Blots/Figure 4/Figure 4A/ERK signaling pathway/ERK/ERK.jpg]

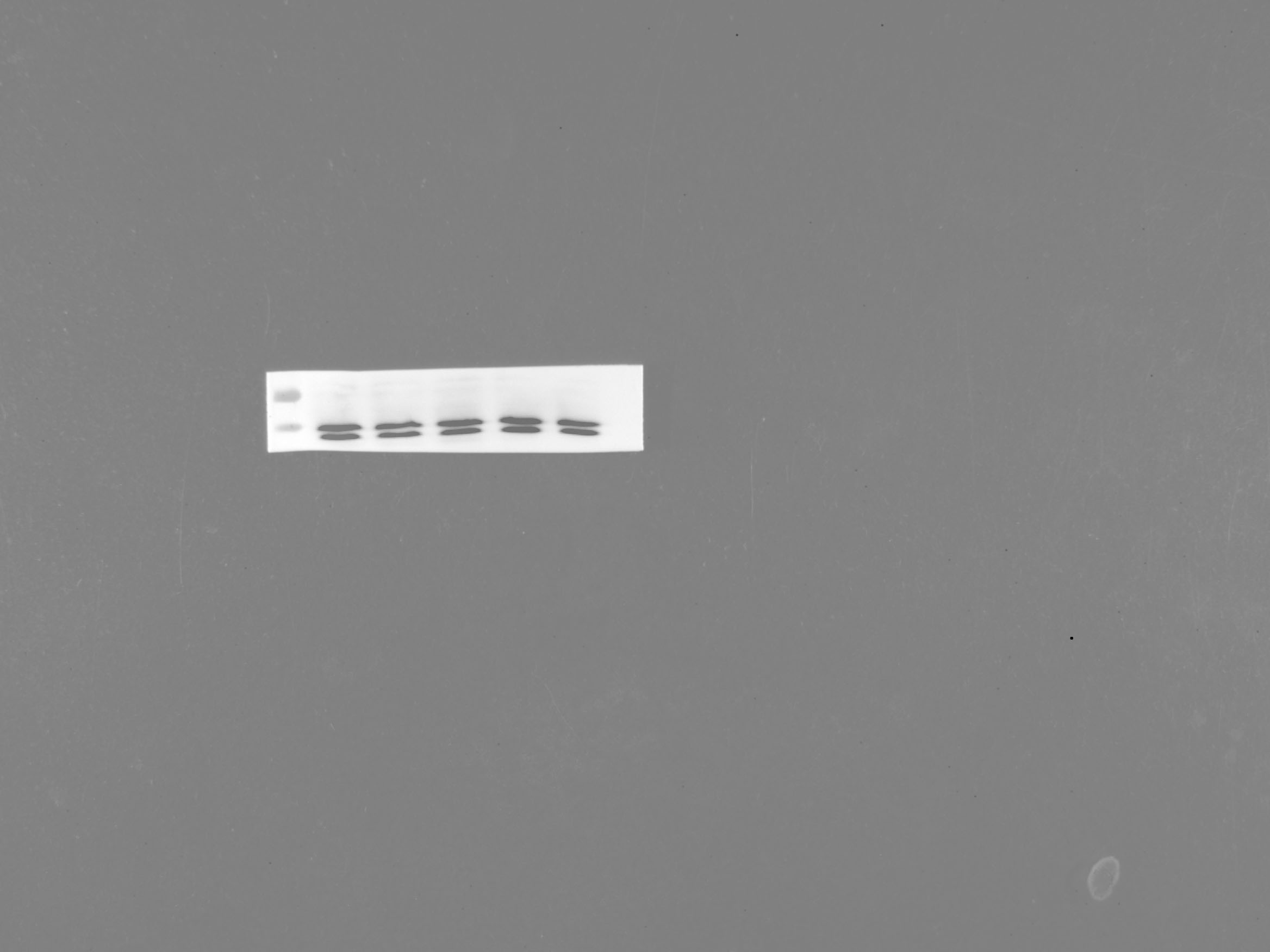

Supplement: Original Images for Blots.zip [file YRER_A_2313366_SM3875.zip › Original Images for Blots/Figure 4/Figure 4A/ERK signaling pathway/ERK/Marker+ERK.jpg]

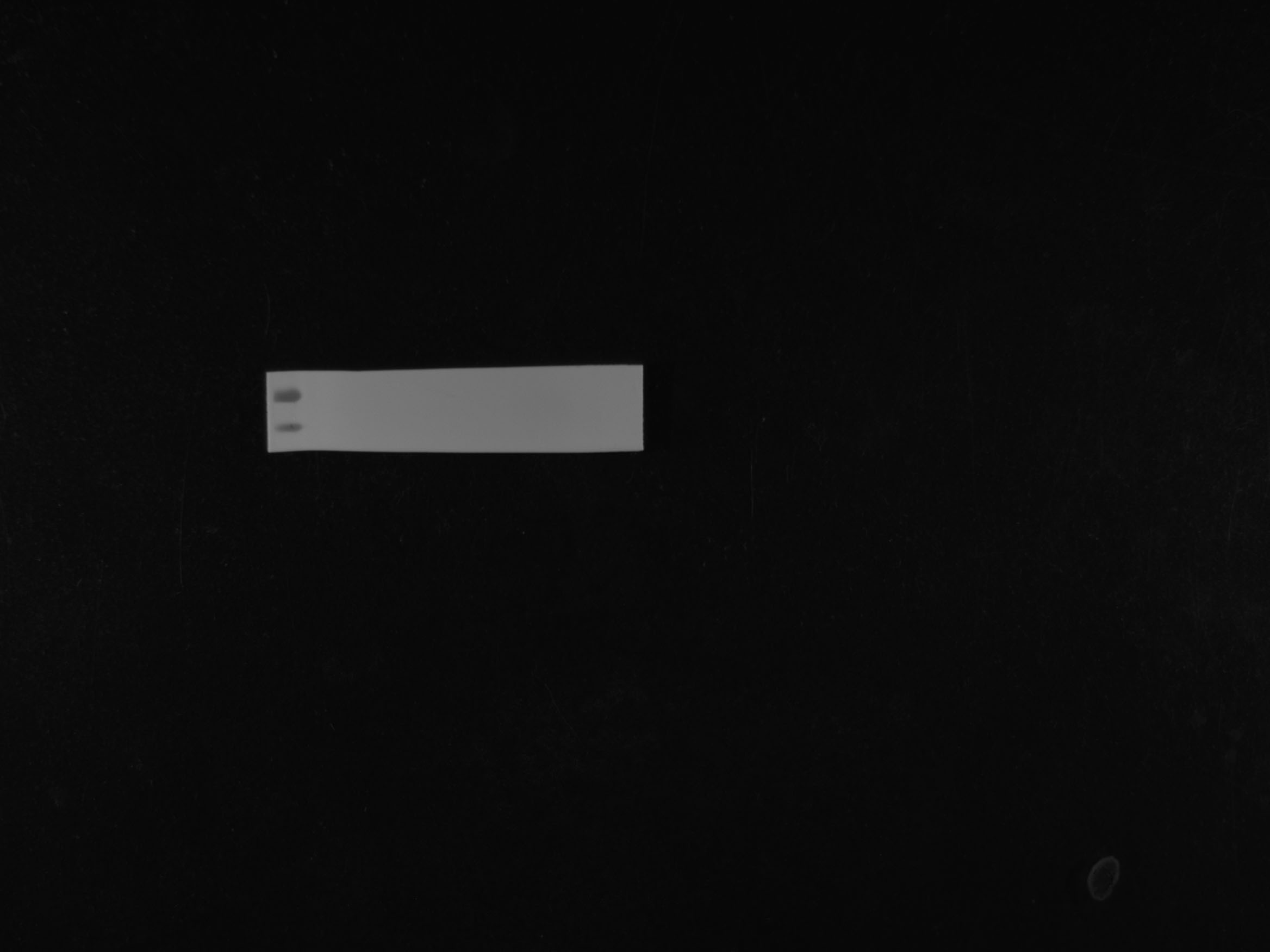

Supplement: Original Images for Blots.zip [file YRER_A_2313366_SM3875.zip › Original Images for Blots/Figure 4/Figure 4A/ERK signaling pathway/ERK/Marker.jpg]

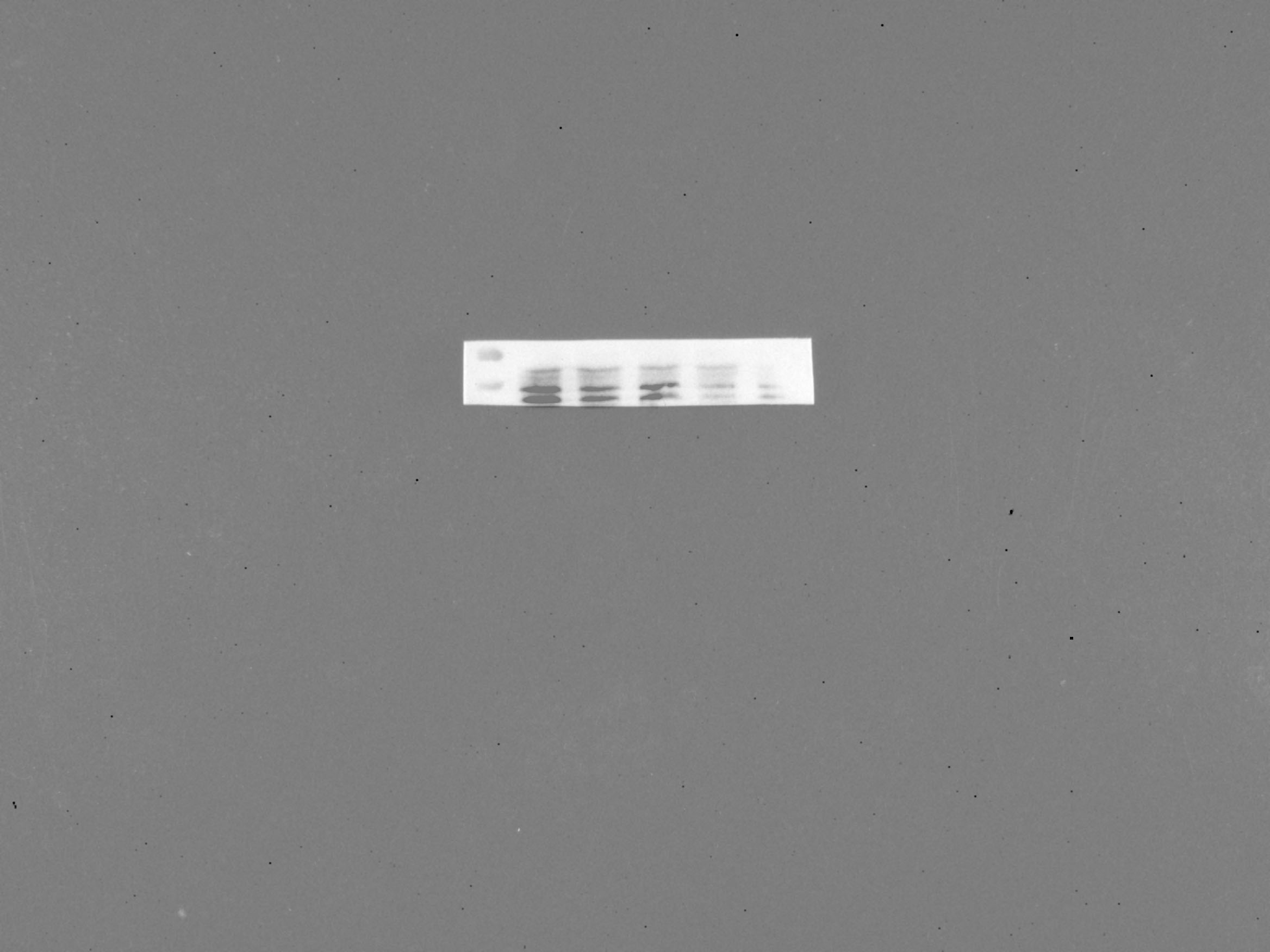

Supplement: Original Images for Blots.zip [file YRER_A_2313366_SM3875.zip › Original Images for Blots/Figure 4/Figure 4A/ERK signaling pathway/p-ERK/Marker+p-ERK.jpg]

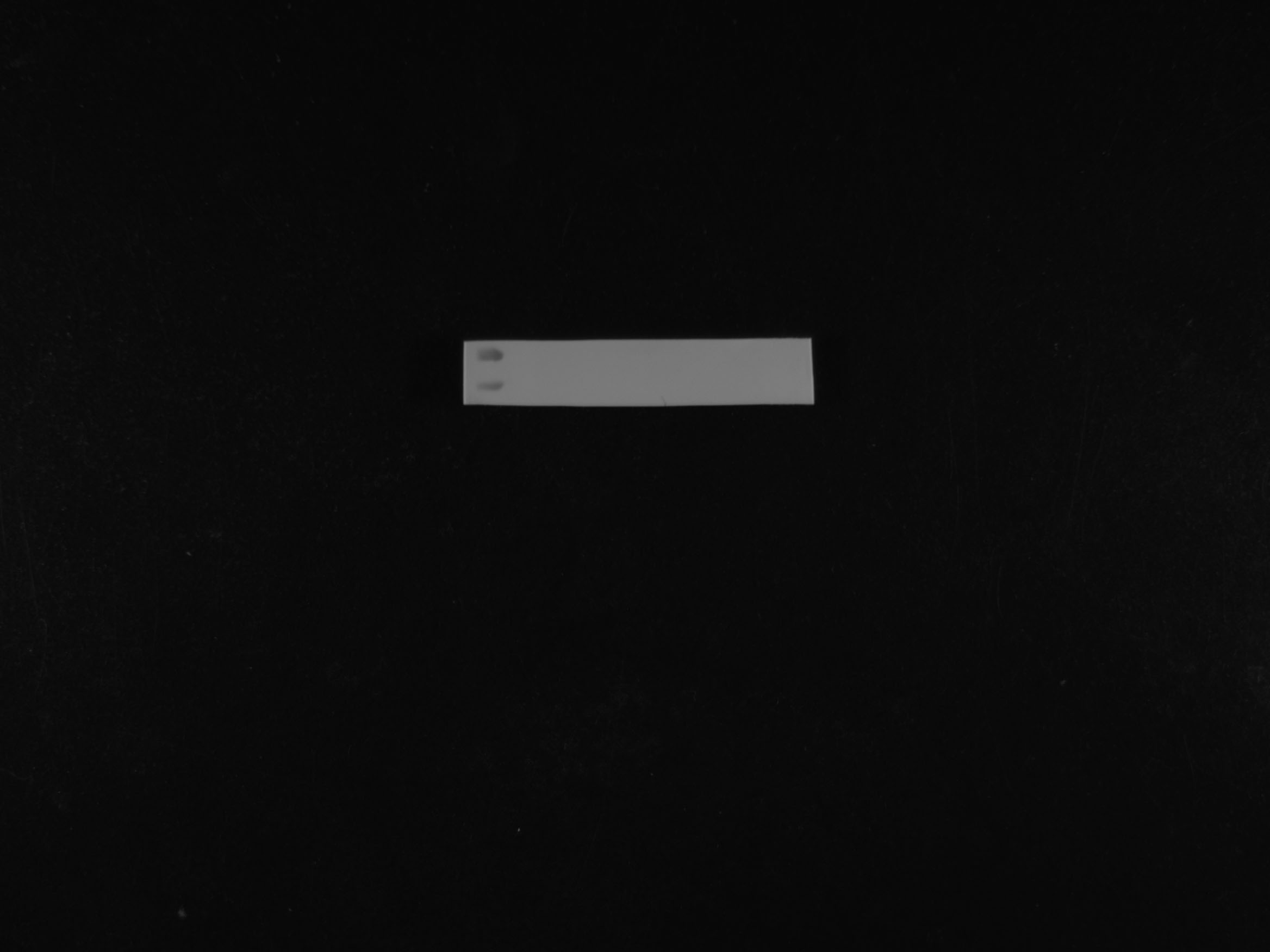

Supplement: Original Images for Blots.zip [file YRER_A_2313366_SM3875.zip › Original Images for Blots/Figure 4/Figure 4A/ERK signaling pathway/p-ERK/Marker.jpg]

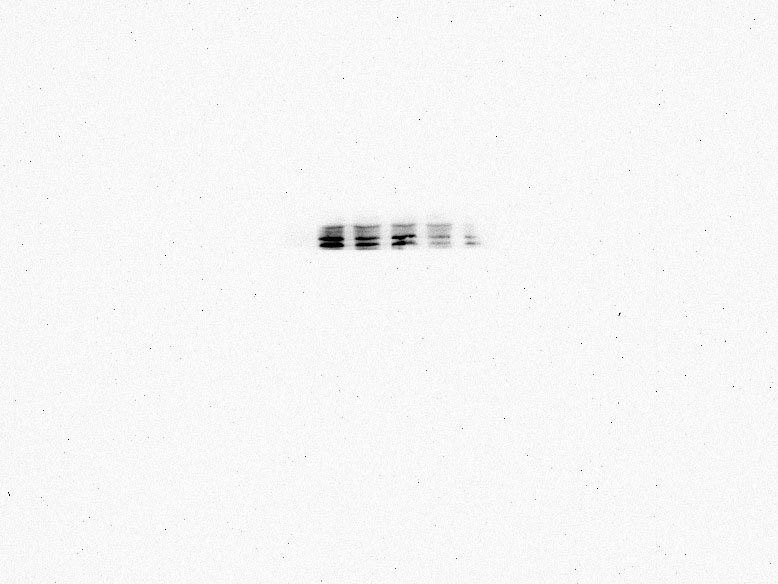

Supplement: Original Images for Blots.zip [file YRER_A_2313366_SM3875.zip › Original Images for Blots/Figure 4/Figure 4A/ERK signaling pathway/p-ERK/p-ERK.jpg]

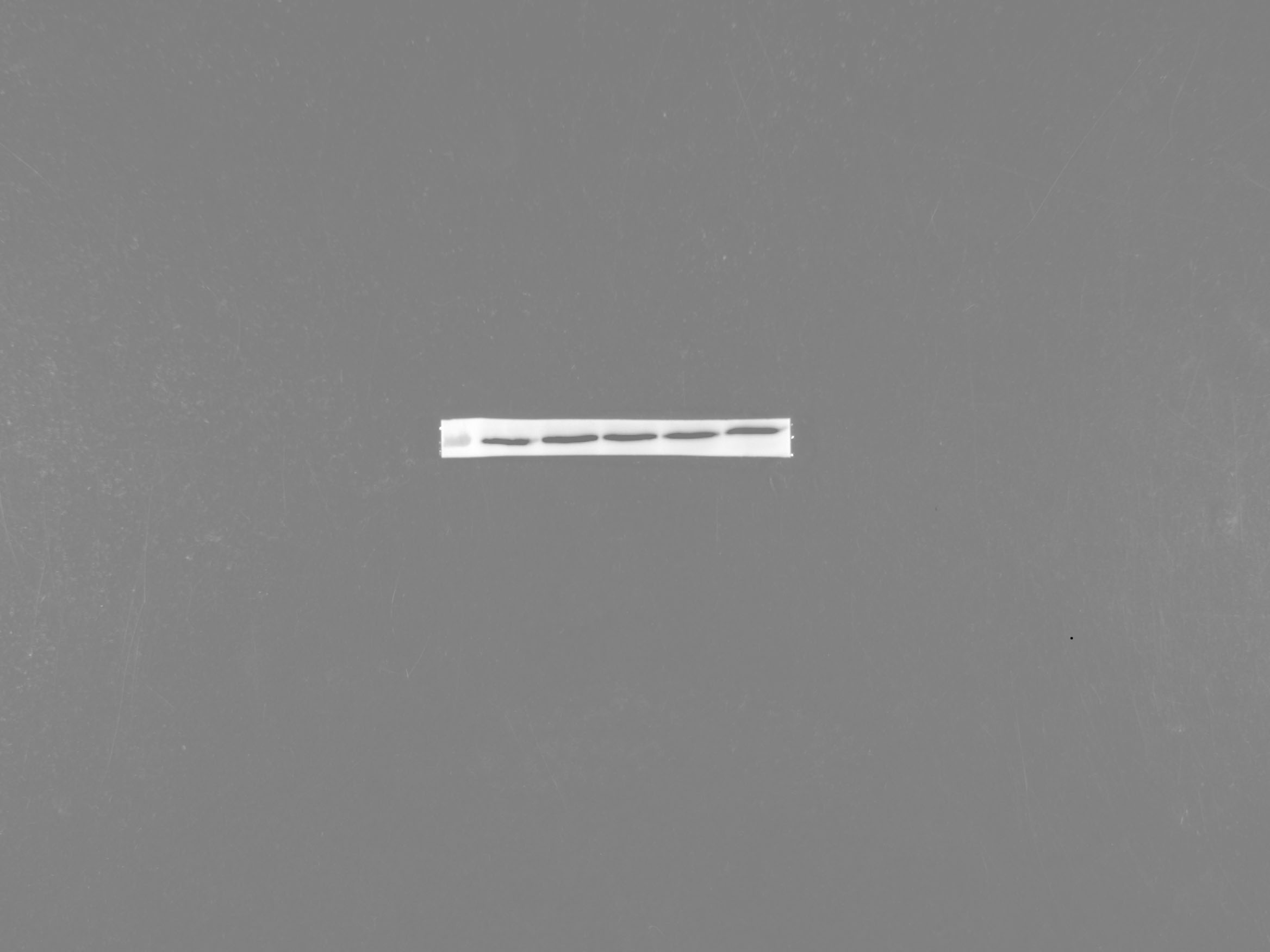

Supplement: Original Images for Blots.zip [file YRER_A_2313366_SM3875.zip › Original Images for Blots/Figure 4/Figure 4A/ERK signaling pathway/α-tubulin/Marker+α-tubulin.jpg]

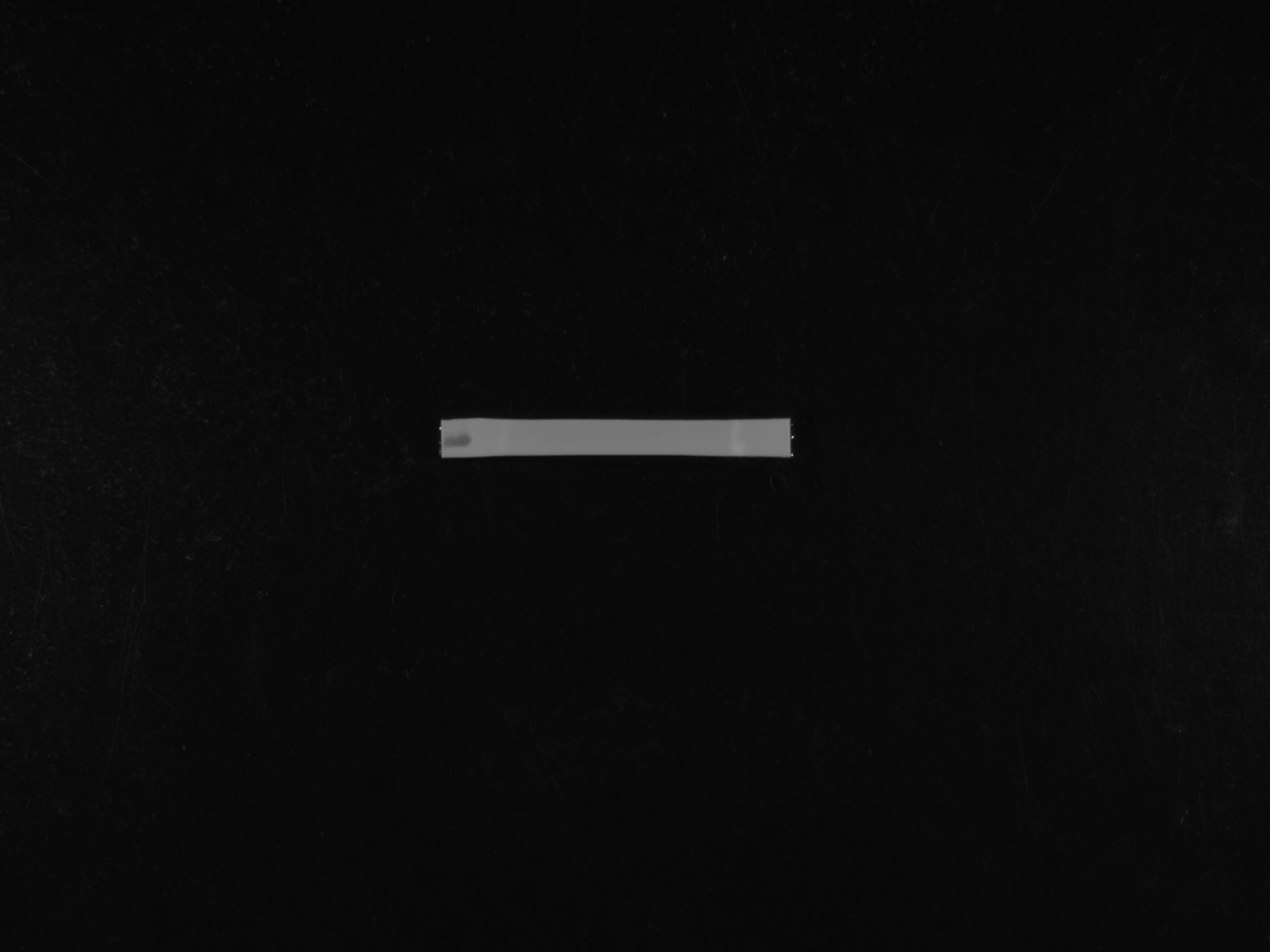

Supplement: Original Images for Blots.zip [file YRER_A_2313366_SM3875.zip › Original Images for Blots/Figure 4/Figure 4A/ERK signaling pathway/α-tubulin/Marker.jpg]

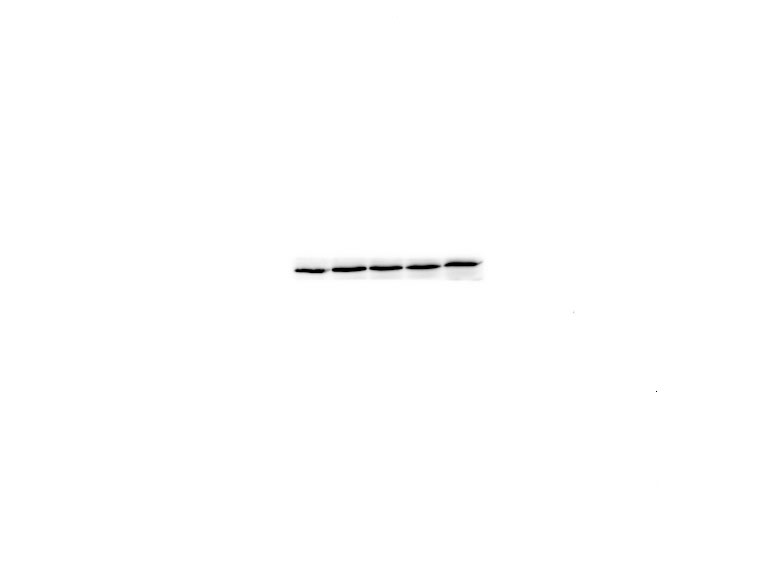

Supplement: Original Images for Blots.zip [file YRER_A_2313366_SM3875.zip › Original Images for Blots/Figure 4/Figure 4A/ERK signaling pathway/α-tubulin/α-tubulin.jpg]

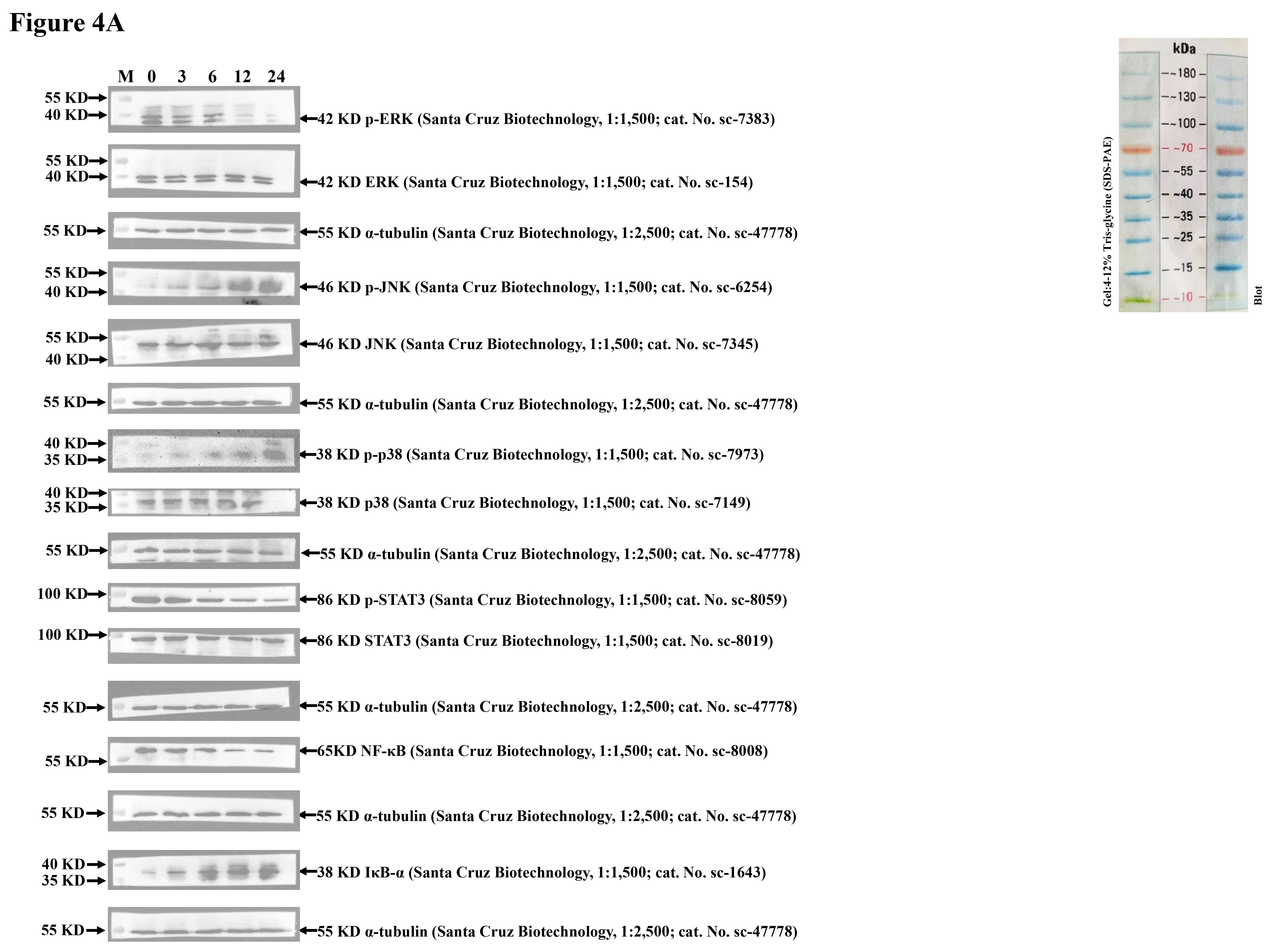

Supplement: Original Images for Blots.zip [file YRER_A_2313366_SM3875.zip › Original Images for Blots/Figure 4/Figure 4A/Figure 4A.jpg]

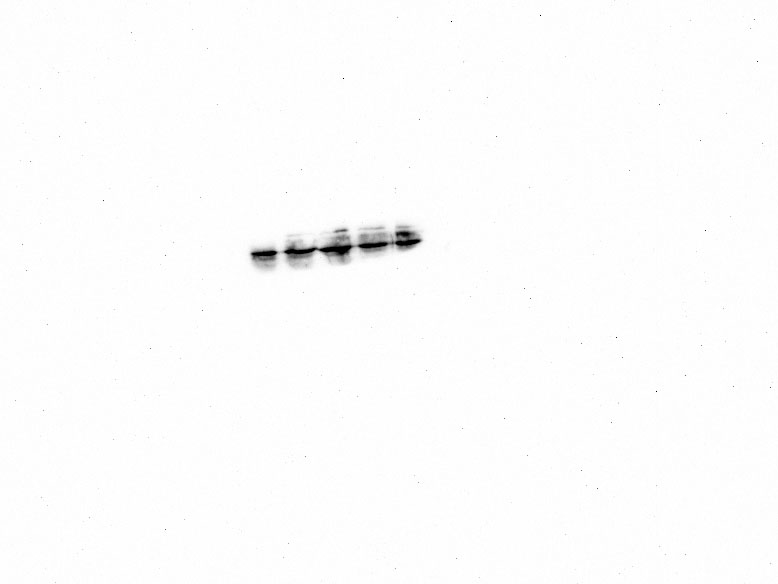

Supplement: Original Images for Blots.zip [file YRER_A_2313366_SM3875.zip › Original Images for Blots/Figure 4/Figure 4A/JNK signaling pathway/JNK/JNK.jpg]

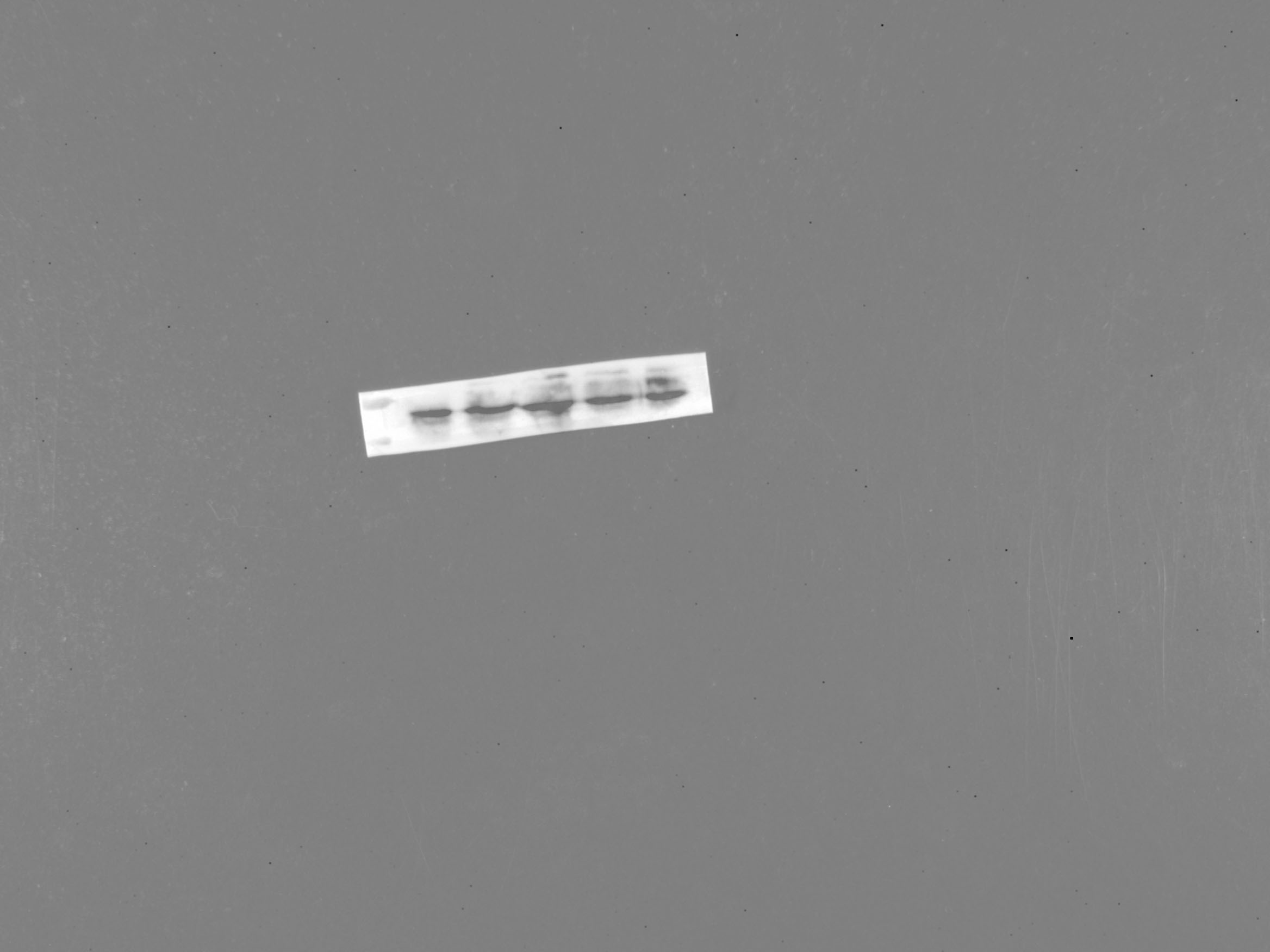

Supplement: Original Images for Blots.zip [file YRER_A_2313366_SM3875.zip › Original Images for Blots/Figure 4/Figure 4A/JNK signaling pathway/JNK/Marker+JNK.jpg]

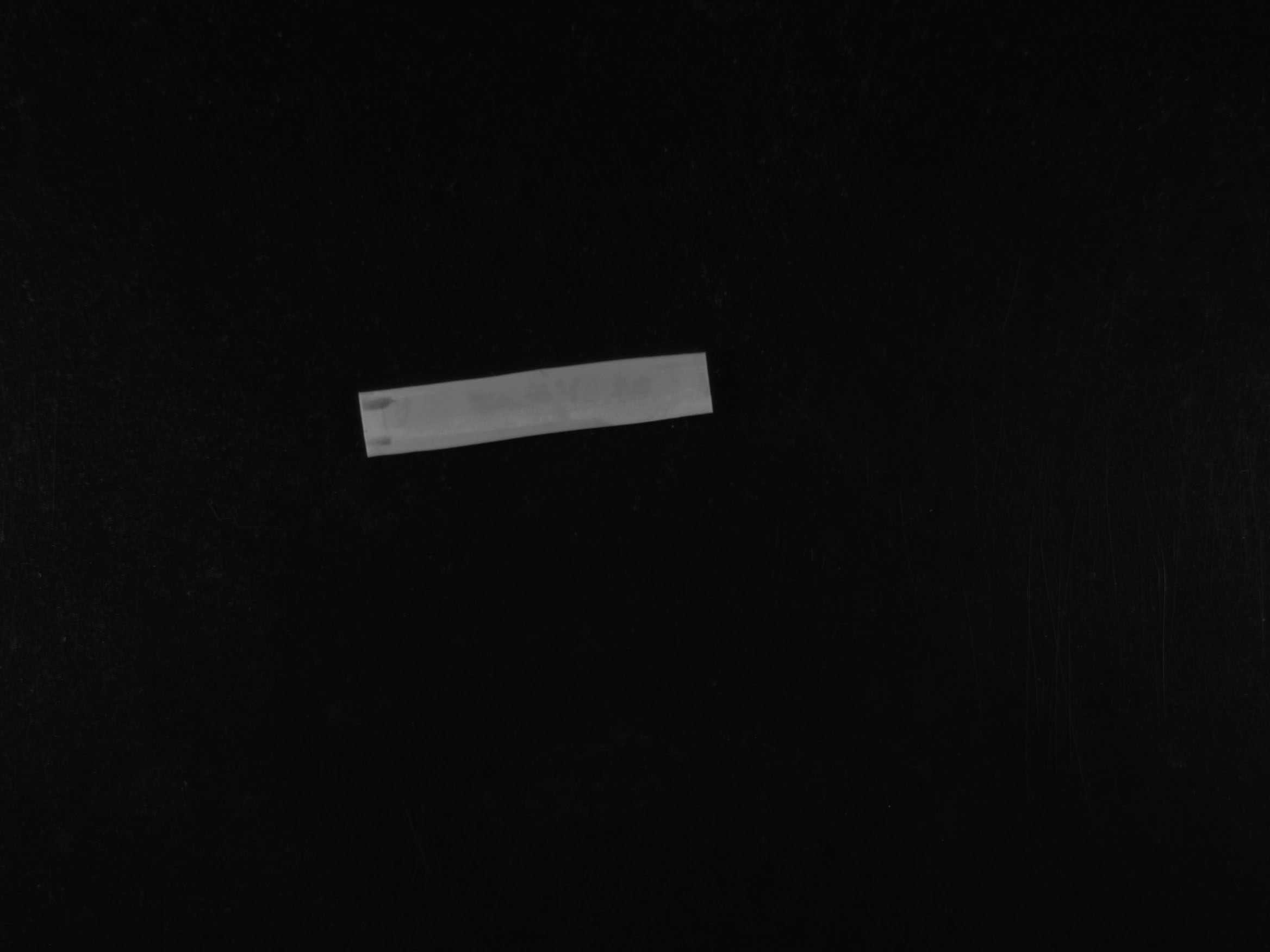

Supplement: Original Images for Blots.zip [file YRER_A_2313366_SM3875.zip › Original Images for Blots/Figure 4/Figure 4A/JNK signaling pathway/JNK/Marker.jpg]

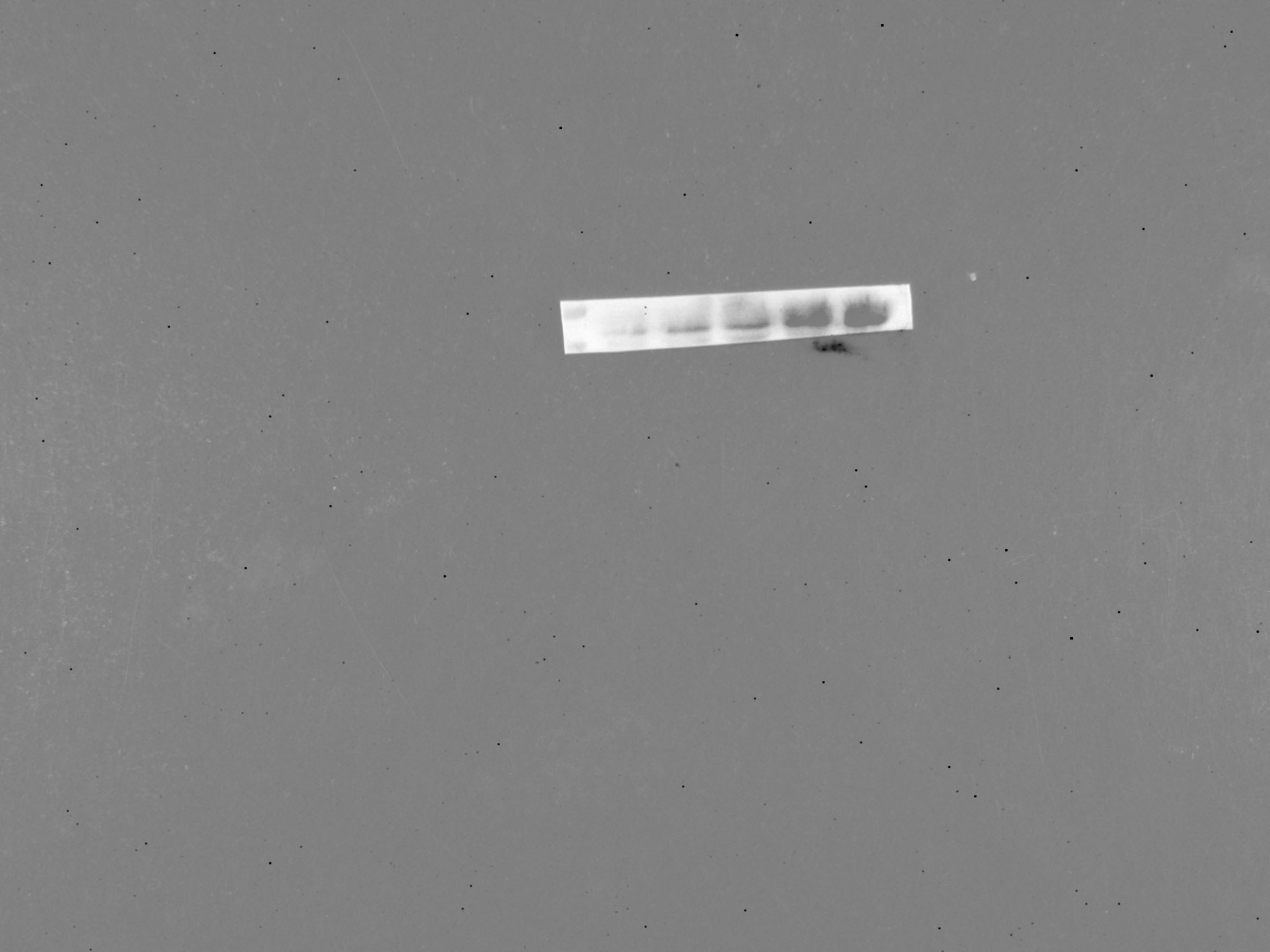

Supplement: Original Images for Blots.zip [file YRER_A_2313366_SM3875.zip › Original Images for Blots/Figure 4/Figure 4A/JNK signaling pathway/p-JNK/Marker+p-JNK.jpg]

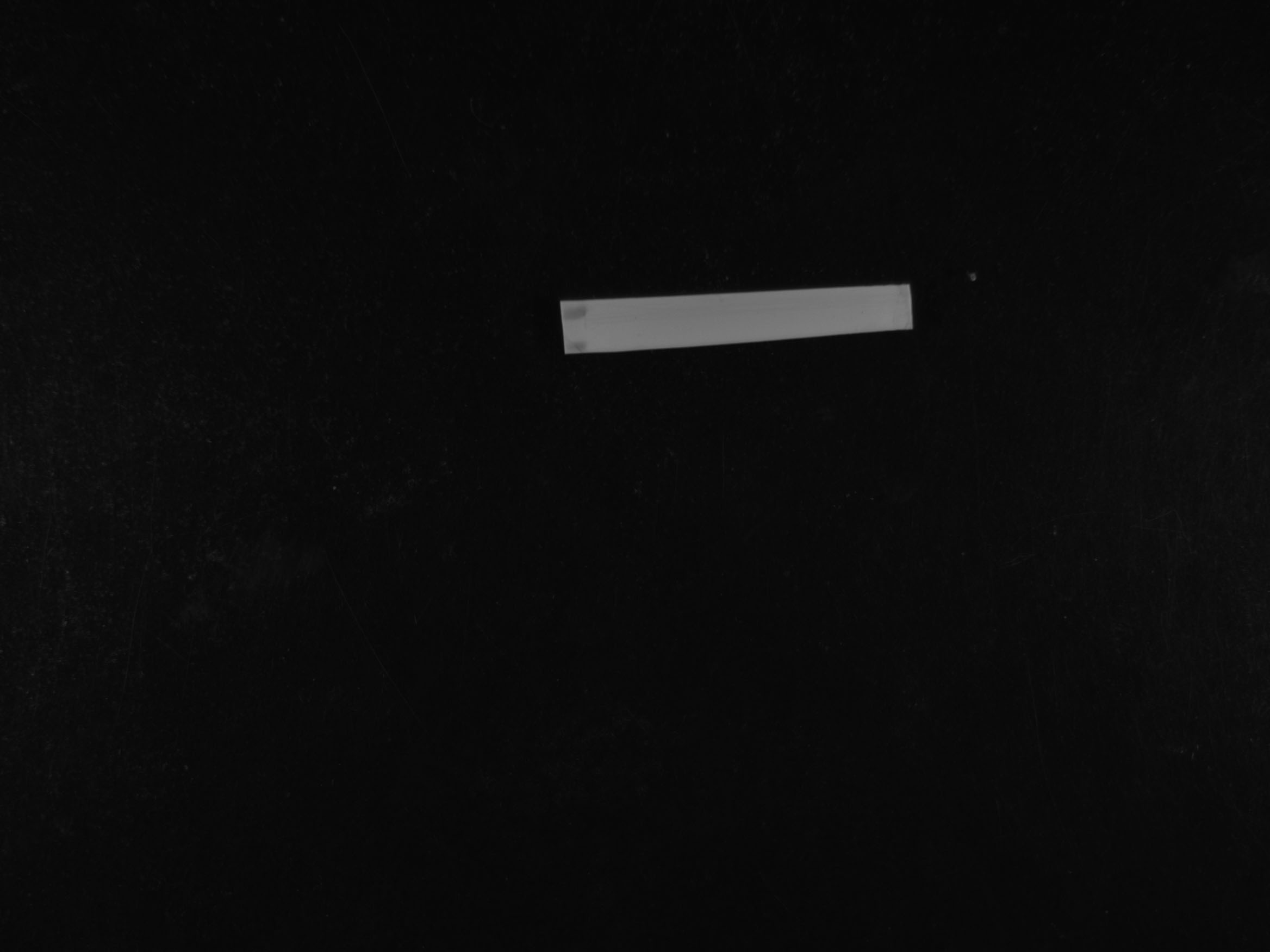

Supplement: Original Images for Blots.zip [file YRER_A_2313366_SM3875.zip › Original Images for Blots/Figure 4/Figure 4A/JNK signaling pathway/p-JNK/Marker.jpg]

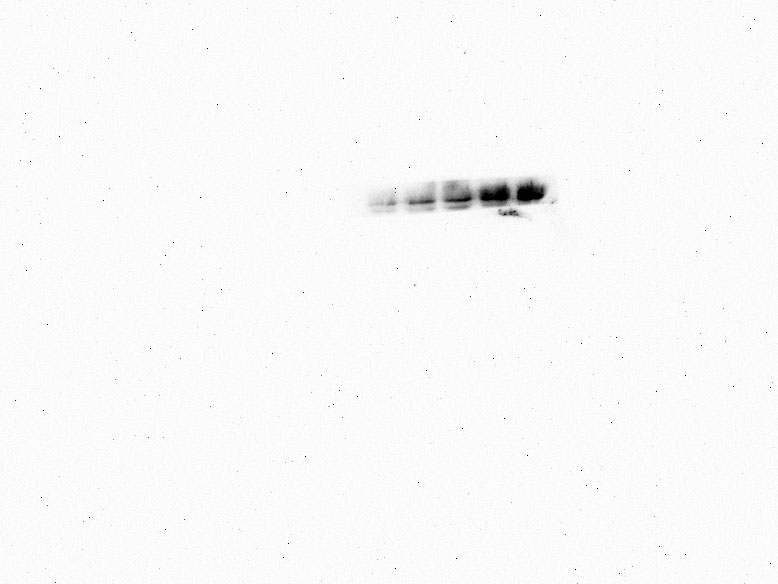

Supplement: Original Images for Blots.zip [file YRER_A_2313366_SM3875.zip › Original Images for Blots/Figure 4/Figure 4A/JNK signaling pathway/p-JNK/p-JNK.jpg]

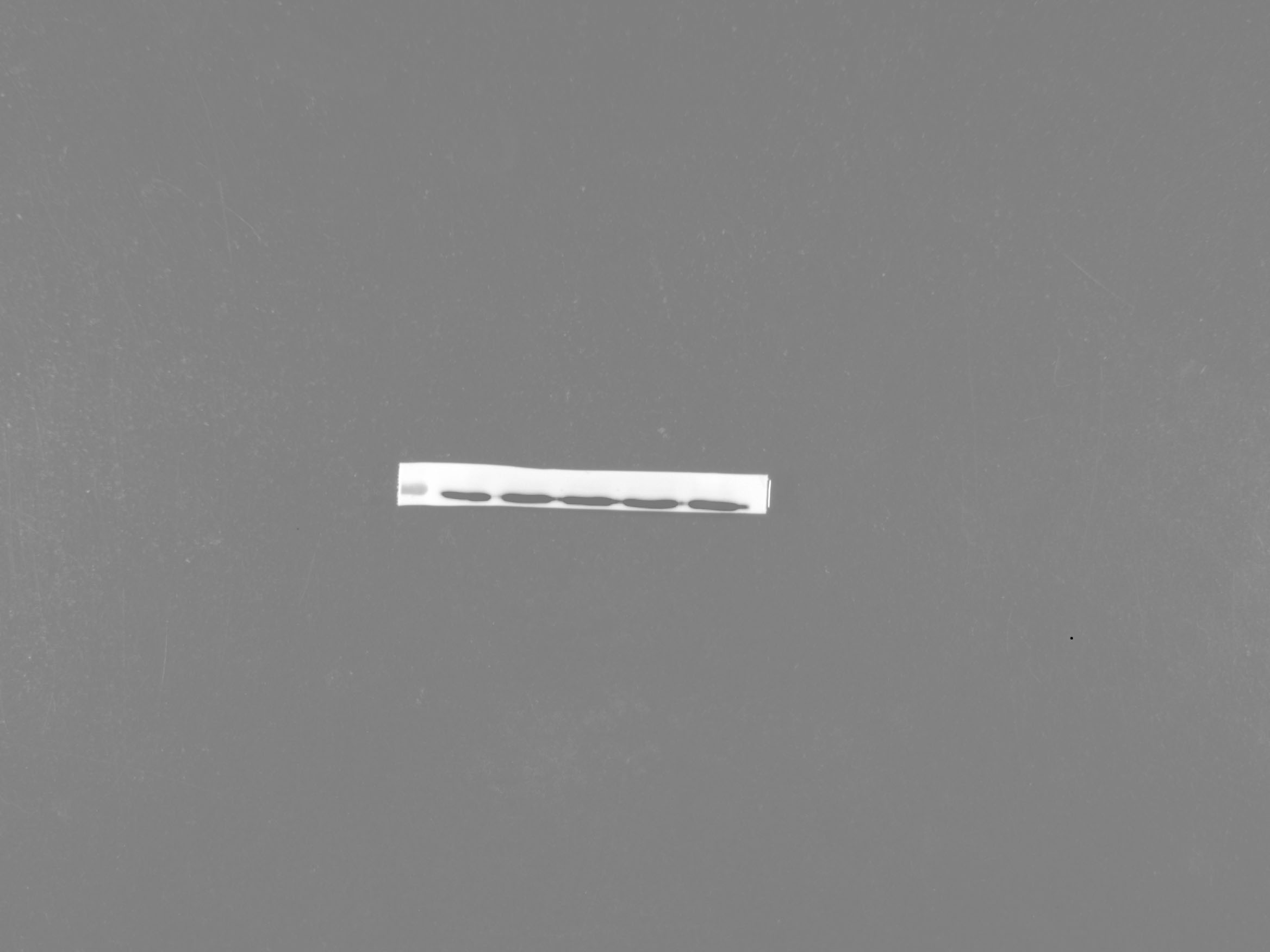

Supplement: Original Images for Blots.zip [file YRER_A_2313366_SM3875.zip › Original Images for Blots/Figure 4/Figure 4A/JNK signaling pathway/α-tubulin/Marker+α-tubulin.jpg]

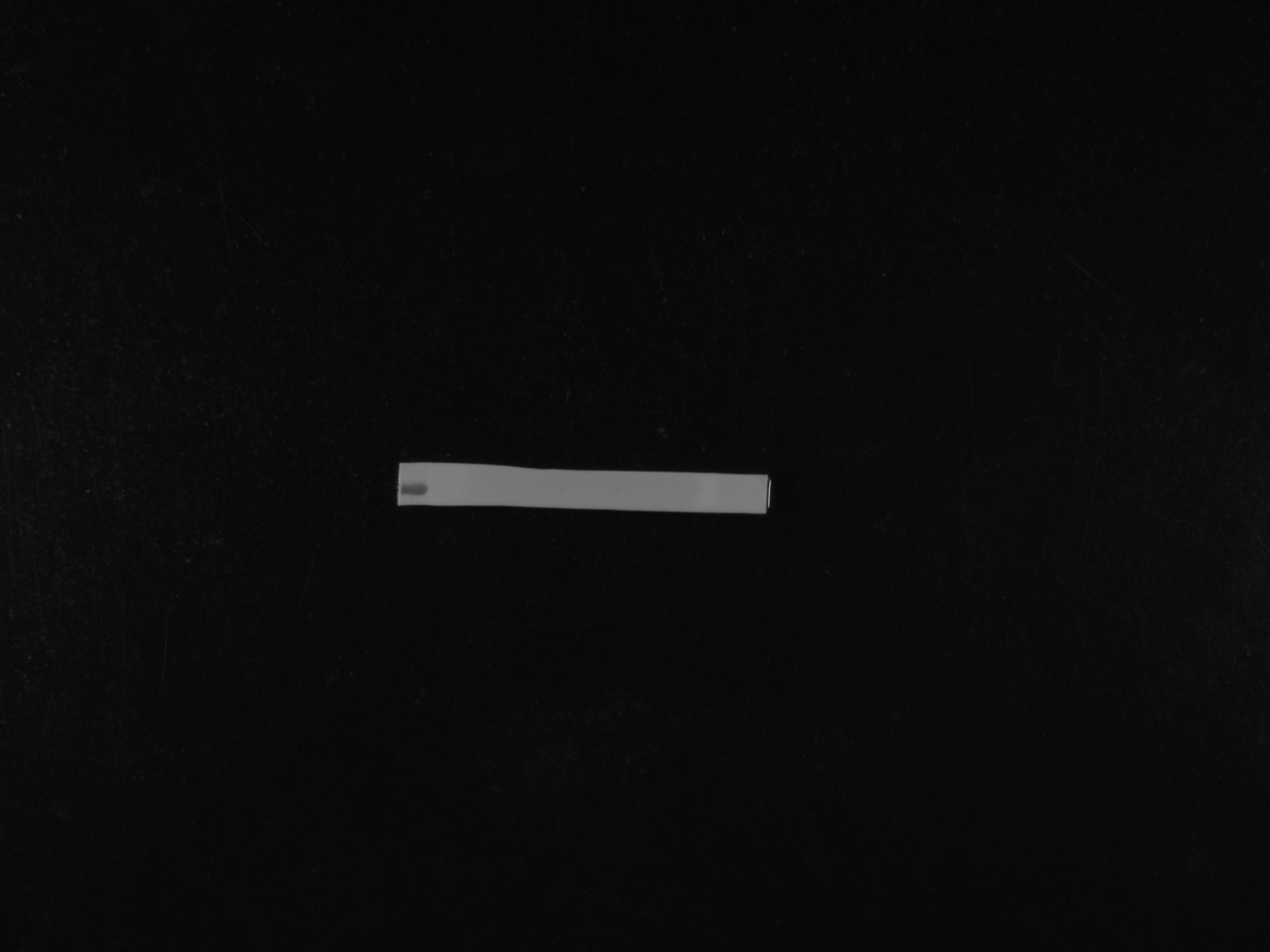

Supplement: Original Images for Blots.zip [file YRER_A_2313366_SM3875.zip › Original Images for Blots/Figure 4/Figure 4A/JNK signaling pathway/α-tubulin/Marker.jpg]

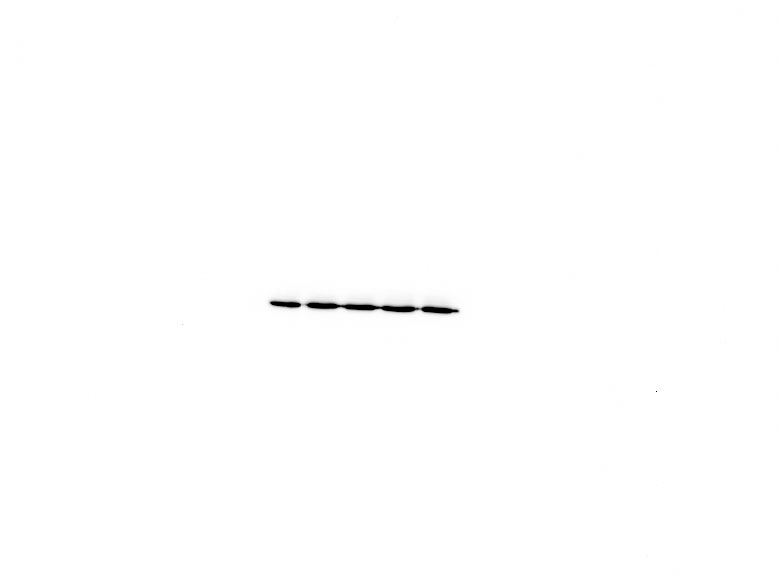

Supplement: Original Images for Blots.zip [file YRER_A_2313366_SM3875.zip › Original Images for Blots/Figure 4/Figure 4A/JNK signaling pathway/α-tubulin/α-tubulin.jpg]

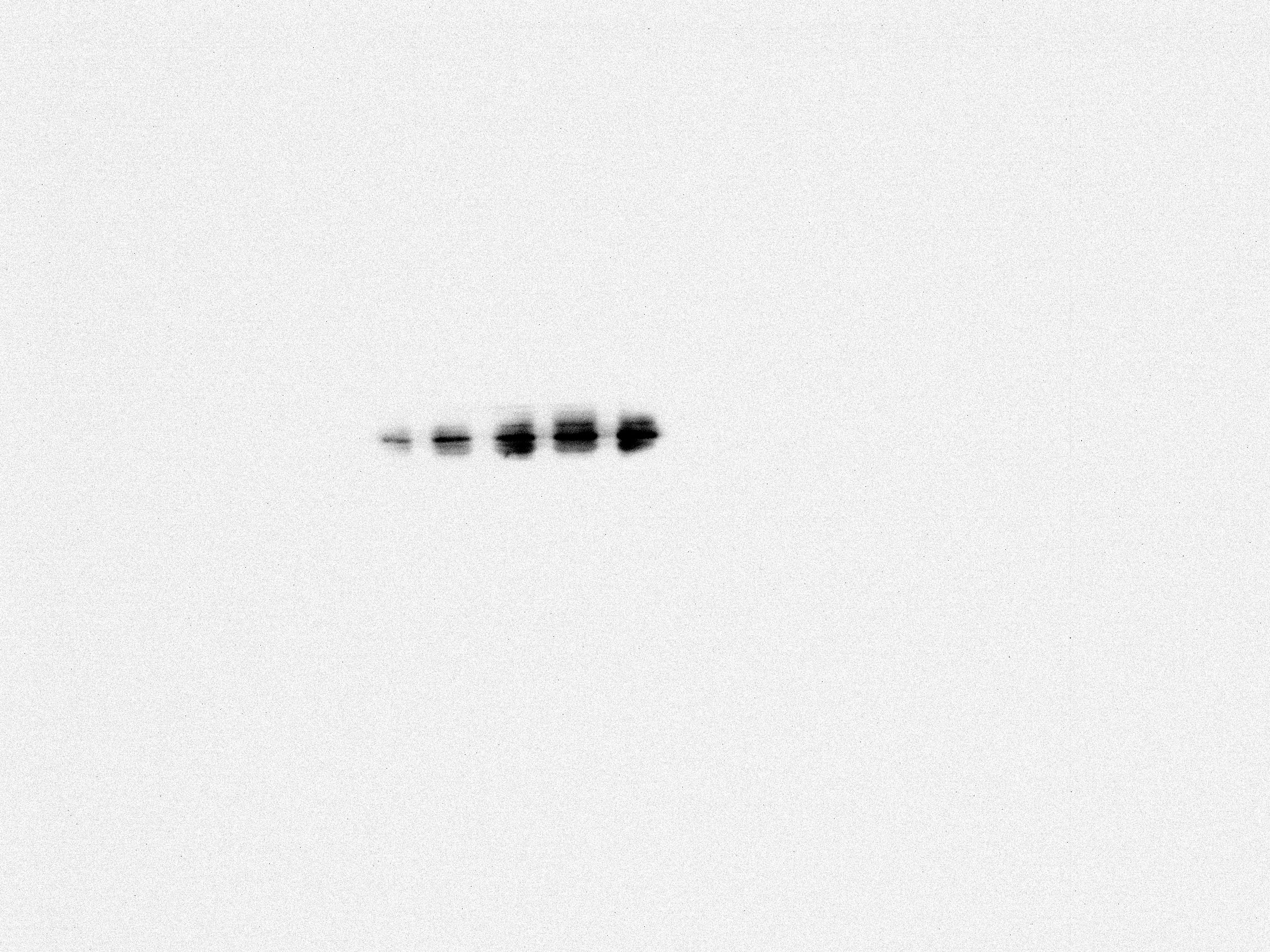

Supplement: Original Images for Blots.zip [file YRER_A_2313366_SM3875.zip › Original Images for Blots/Figure 4/Figure 4A/NF-kb signaling pathway/IκB-α/IκB-α.jpg]

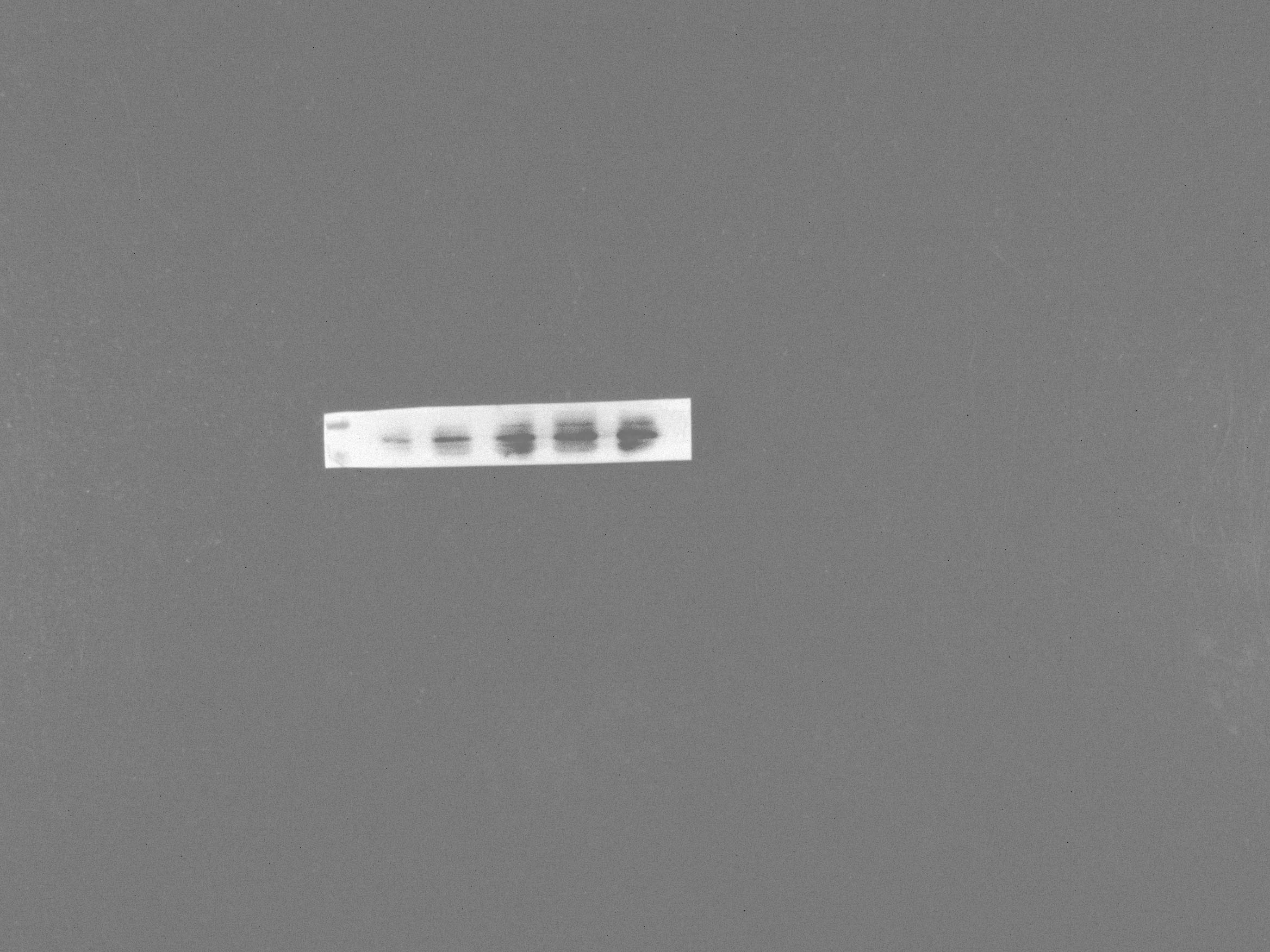

Supplement: Original Images for Blots.zip [file YRER_A_2313366_SM3875.zip › Original Images for Blots/Figure 4/Figure 4A/NF-kb signaling pathway/IκB-α/Marker+IκB-α.jpg]

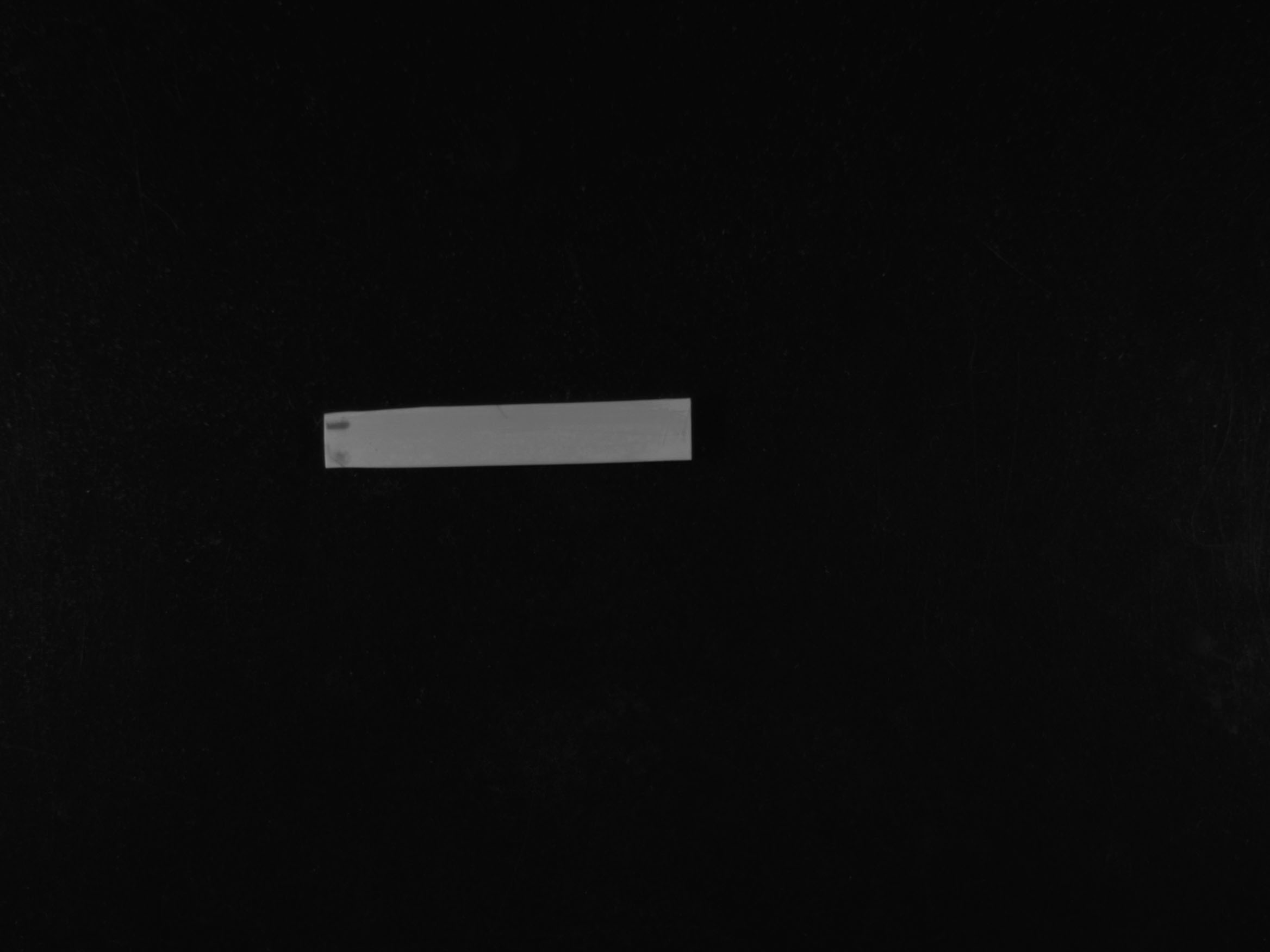

Supplement: Original Images for Blots.zip [file YRER_A_2313366_SM3875.zip › Original Images for Blots/Figure 4/Figure 4A/NF-kb signaling pathway/IκB-α/Marker.jpg]

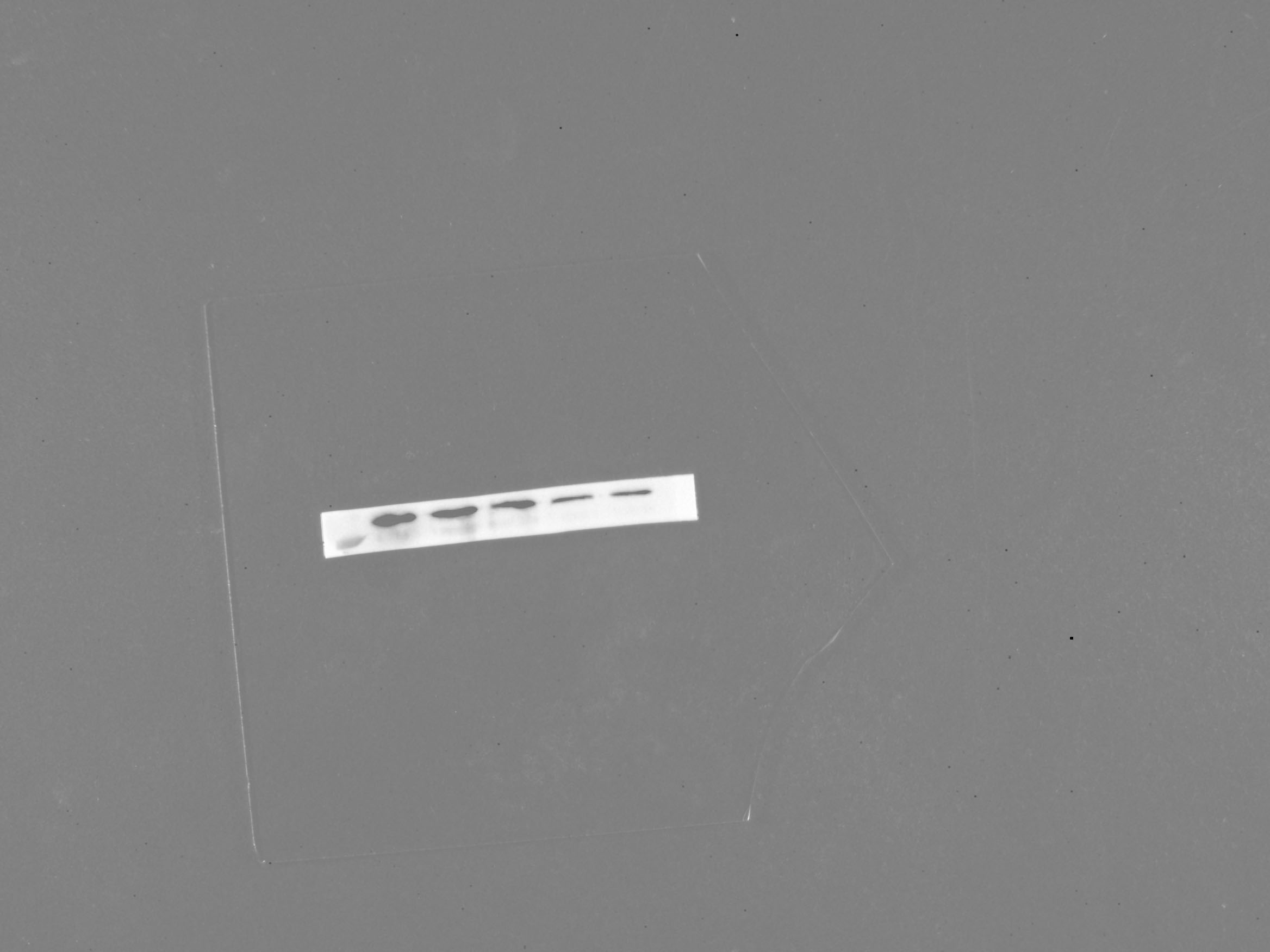

Supplement: Original Images for Blots.zip [file YRER_A_2313366_SM3875.zip › Original Images for Blots/Figure 4/Figure 4A/NF-kb signaling pathway/NF-κB/Marker+NF-κB.jpg]

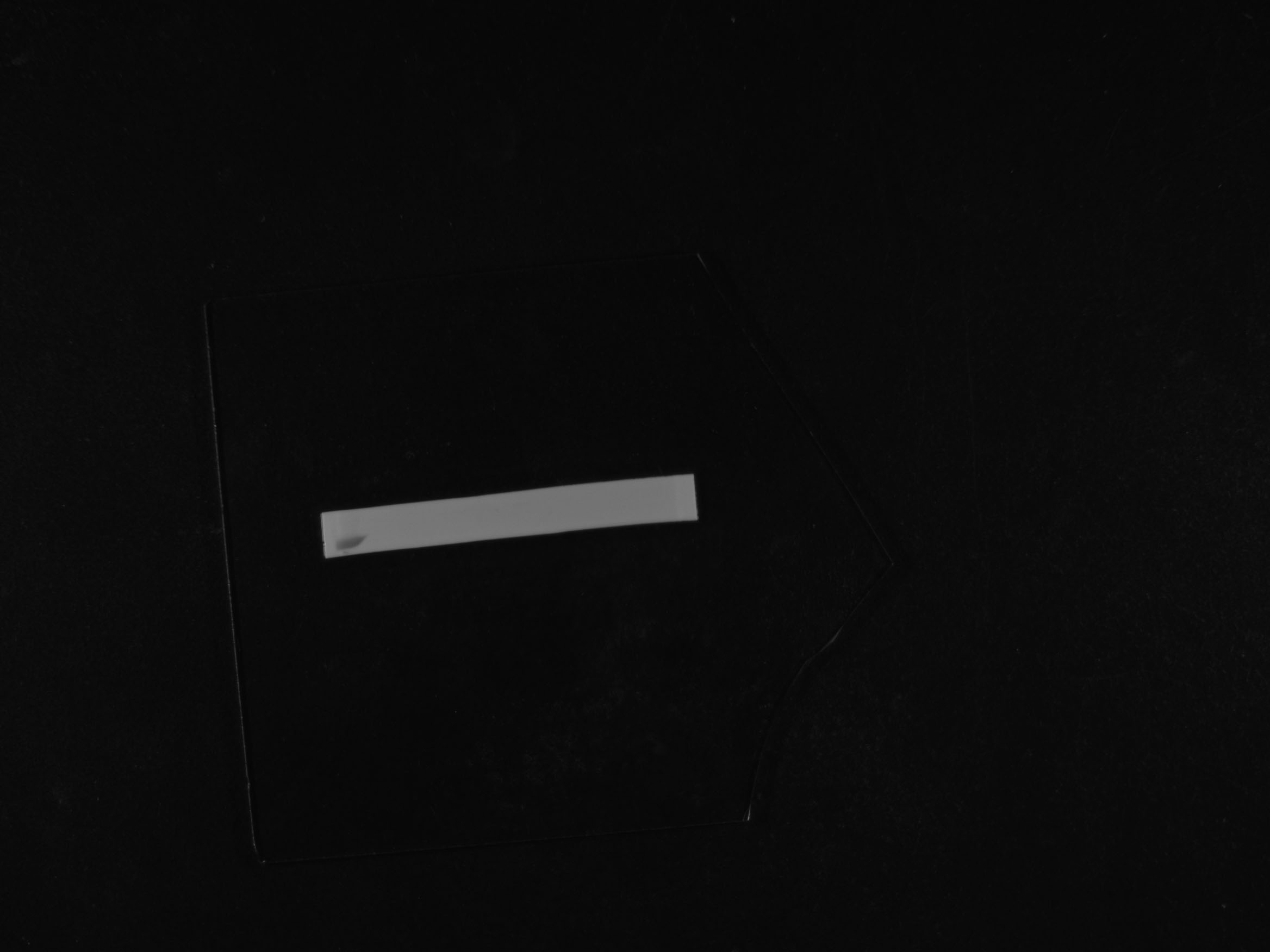

Supplement: Original Images for Blots.zip [file YRER_A_2313366_SM3875.zip › Original Images for Blots/Figure 4/Figure 4A/NF-kb signaling pathway/NF-κB/Marker.jpg]

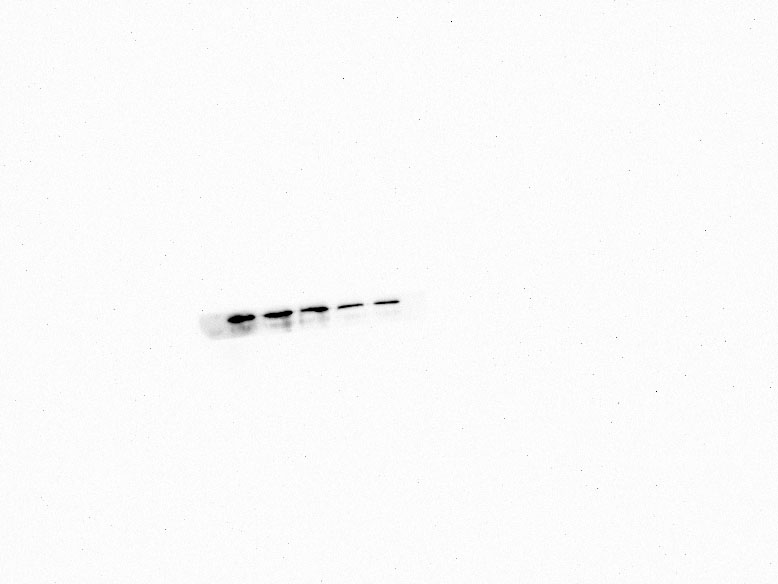

Supplement: Original Images for Blots.zip [file YRER_A_2313366_SM3875.zip › Original Images for Blots/Figure 4/Figure 4A/NF-kb signaling pathway/NF-κB/NF-κB.jpg]

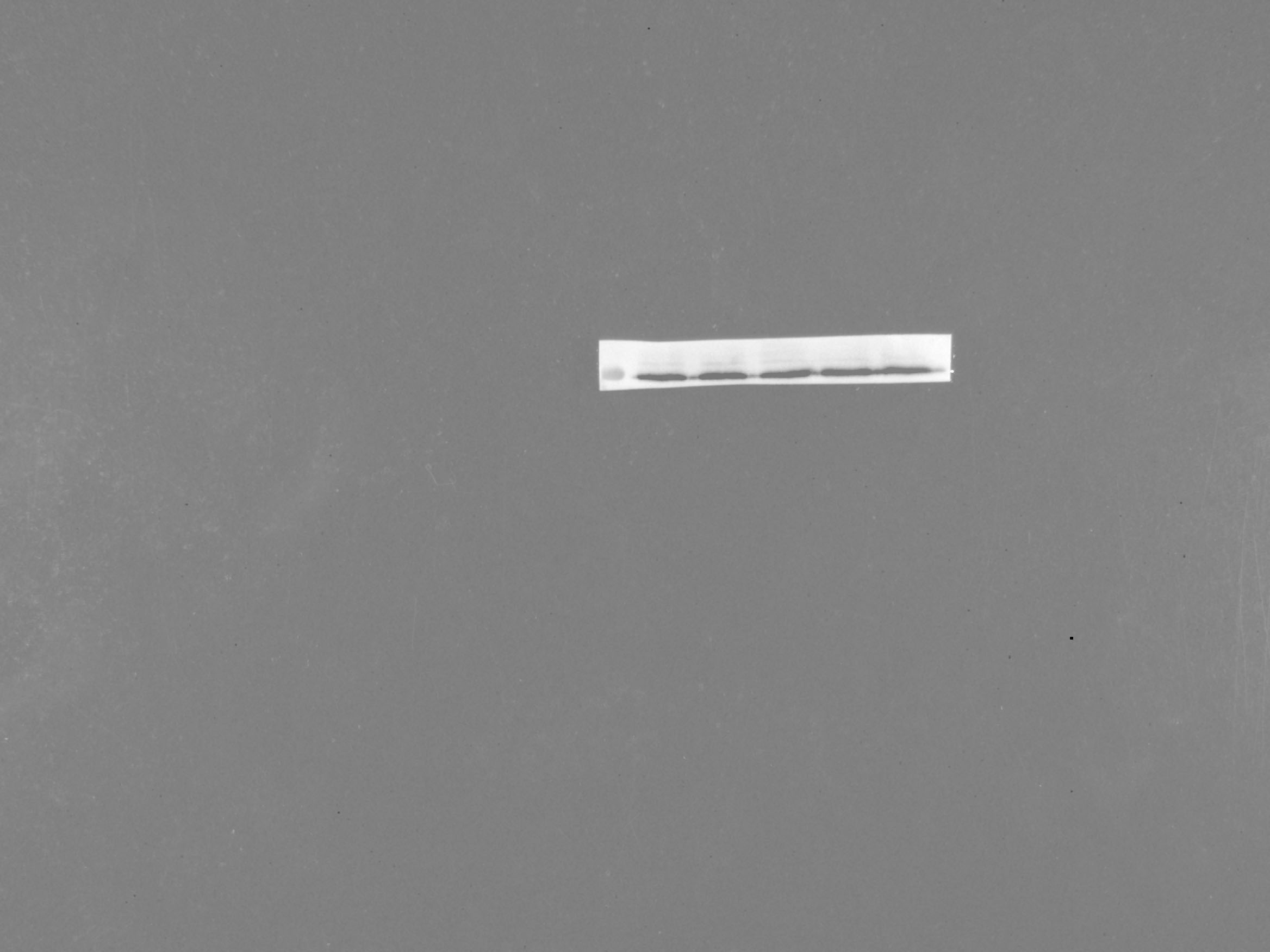

Supplement: Original Images for Blots.zip [file YRER_A_2313366_SM3875.zip › Original Images for Blots/Figure 4/Figure 4A/NF-kb signaling pathway/α-tubulin(IκB-α)/Marker+α-tubulin.jpg]

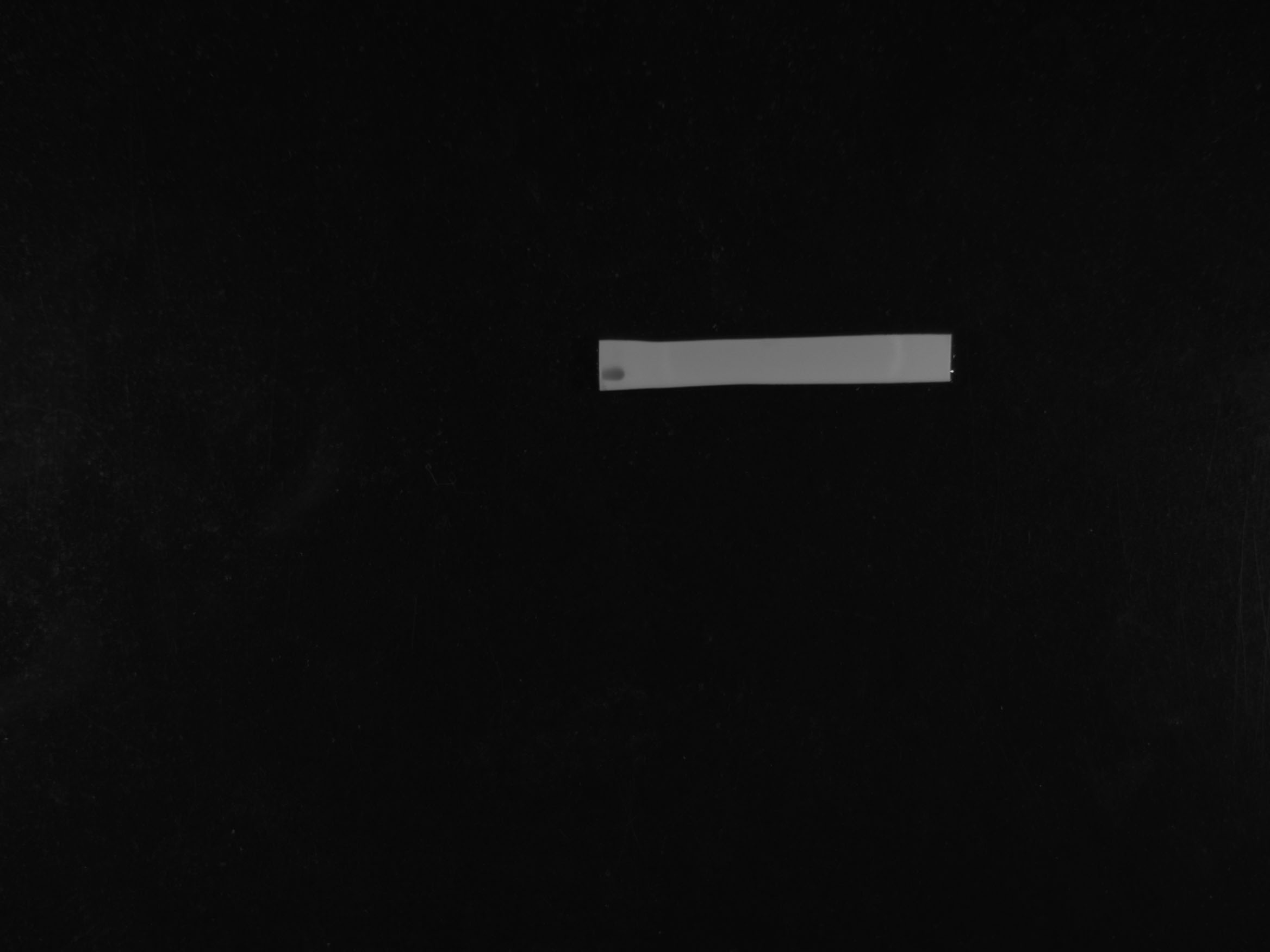

Supplement: Original Images for Blots.zip [file YRER_A_2313366_SM3875.zip › Original Images for Blots/Figure 4/Figure 4A/NF-kb signaling pathway/α-tubulin(IκB-α)/Marker.jpg]

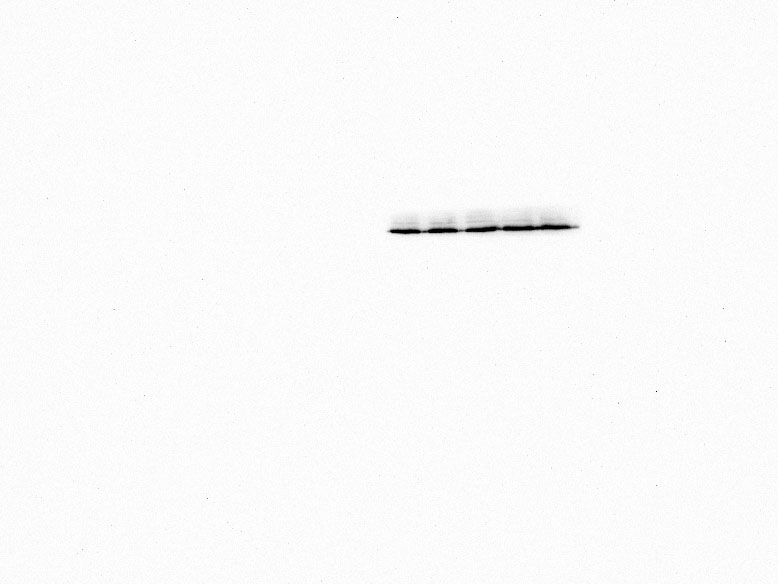

Supplement: Original Images for Blots.zip [file YRER_A_2313366_SM3875.zip › Original Images for Blots/Figure 4/Figure 4A/NF-kb signaling pathway/α-tubulin(IκB-α)/α-tubulin.jpg]

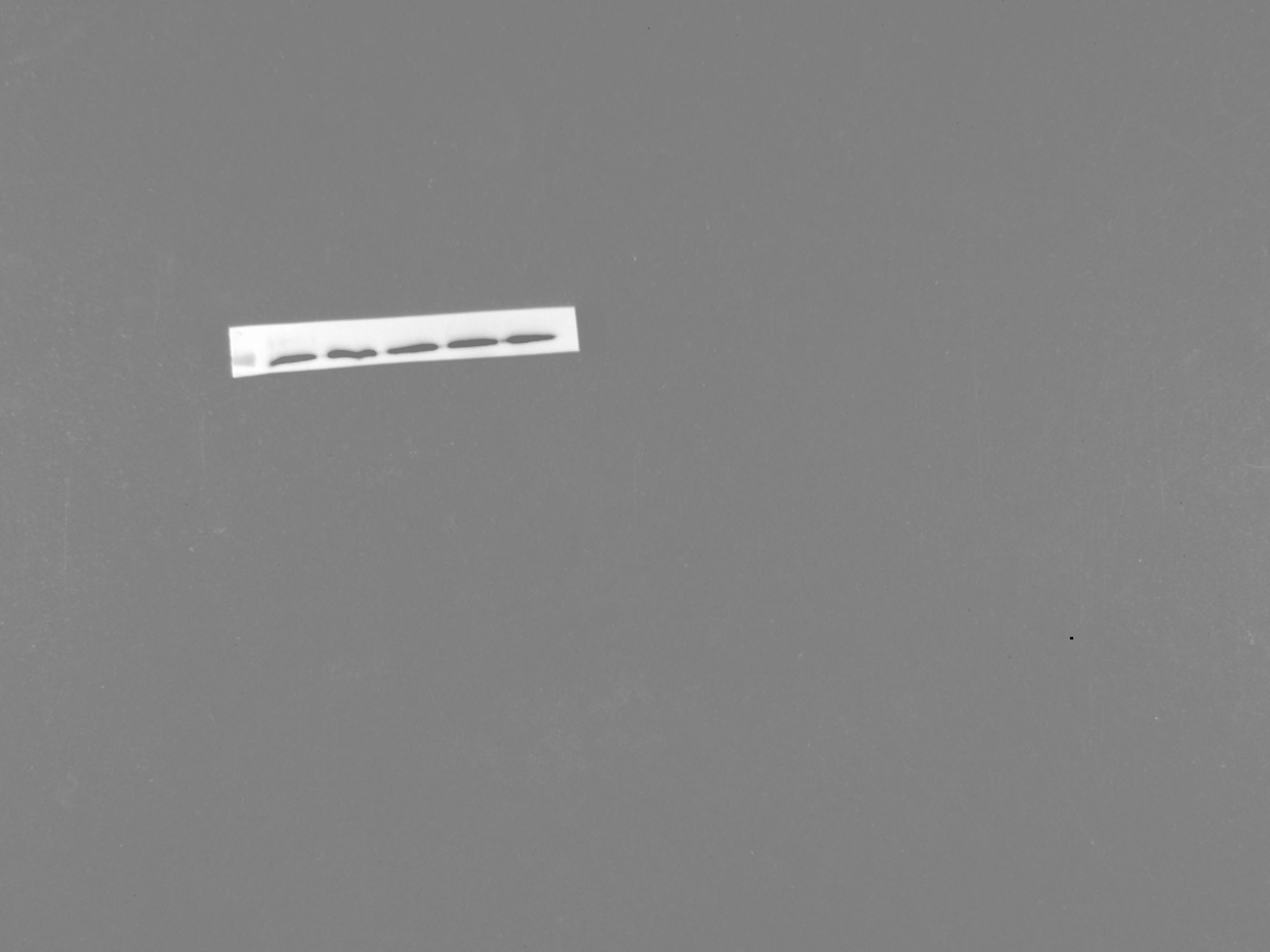

Supplement: Original Images for Blots.zip [file YRER_A_2313366_SM3875.zip › Original Images for Blots/Figure 4/Figure 4A/NF-kb signaling pathway/α-tubulin(NF-κB)/Marker+α-tubulin.jpg]

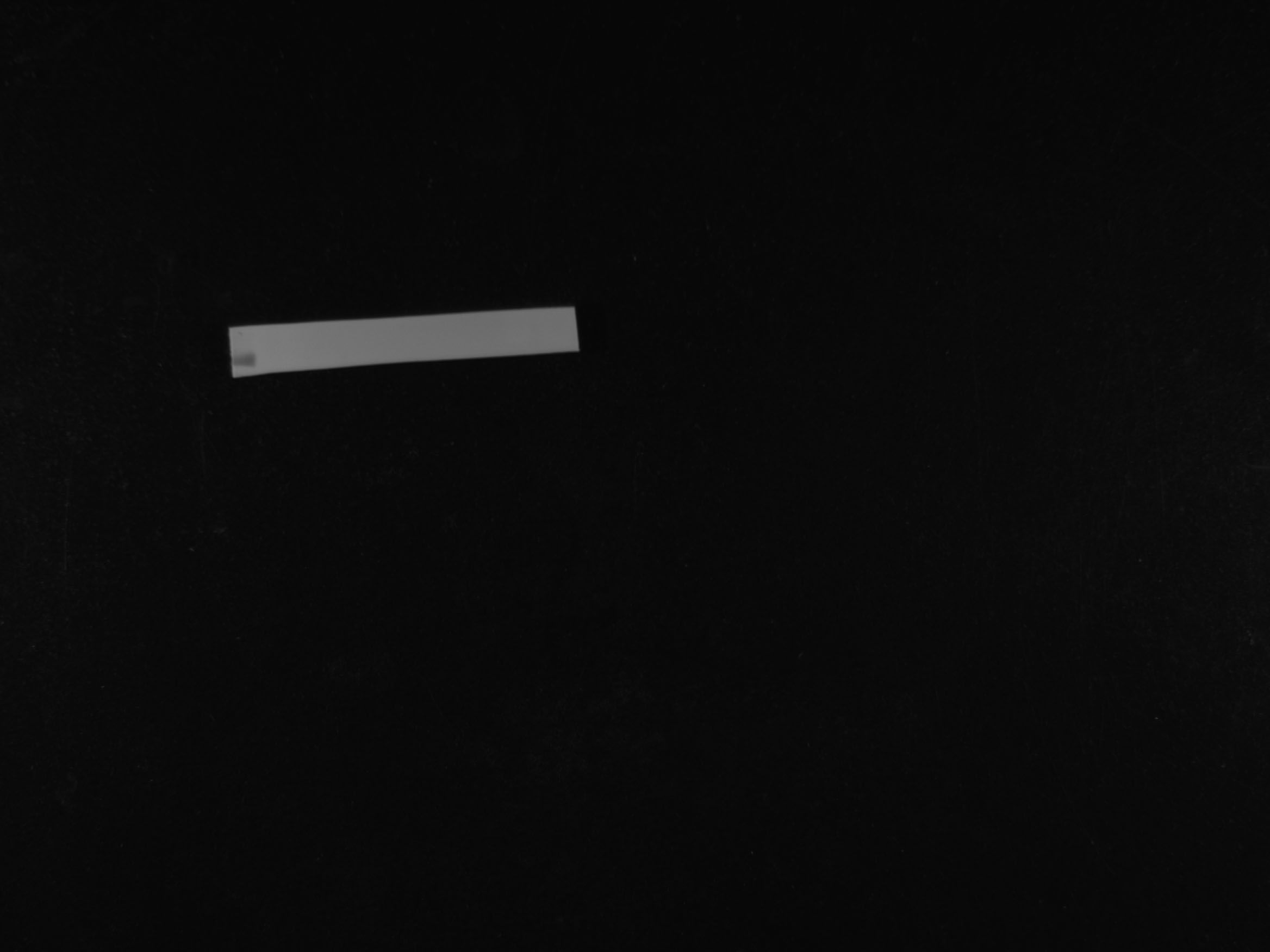

Supplement: Original Images for Blots.zip [file YRER_A_2313366_SM3875.zip › Original Images for Blots/Figure 4/Figure 4A/NF-kb signaling pathway/α-tubulin(NF-κB)/Marker.jpg]

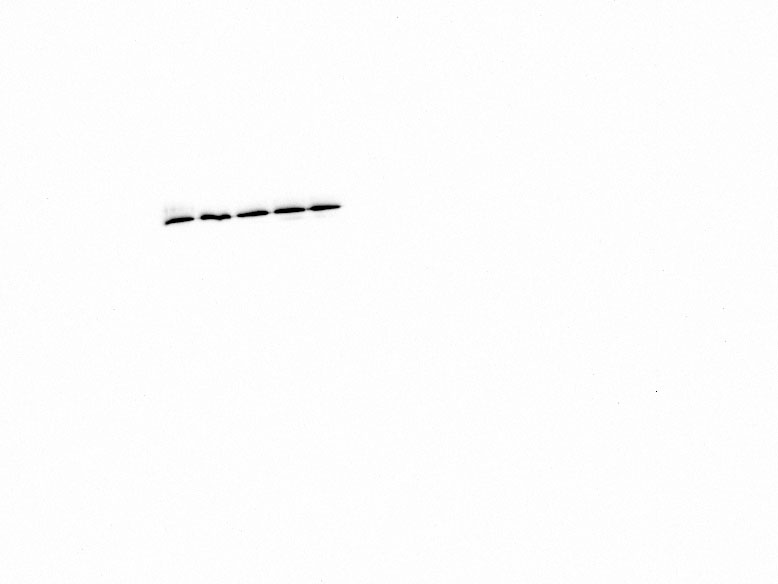

Supplement: Original Images for Blots.zip [file YRER_A_2313366_SM3875.zip › Original Images for Blots/Figure 4/Figure 4A/NF-kb signaling pathway/α-tubulin(NF-κB)/α-tubulin.jpg]

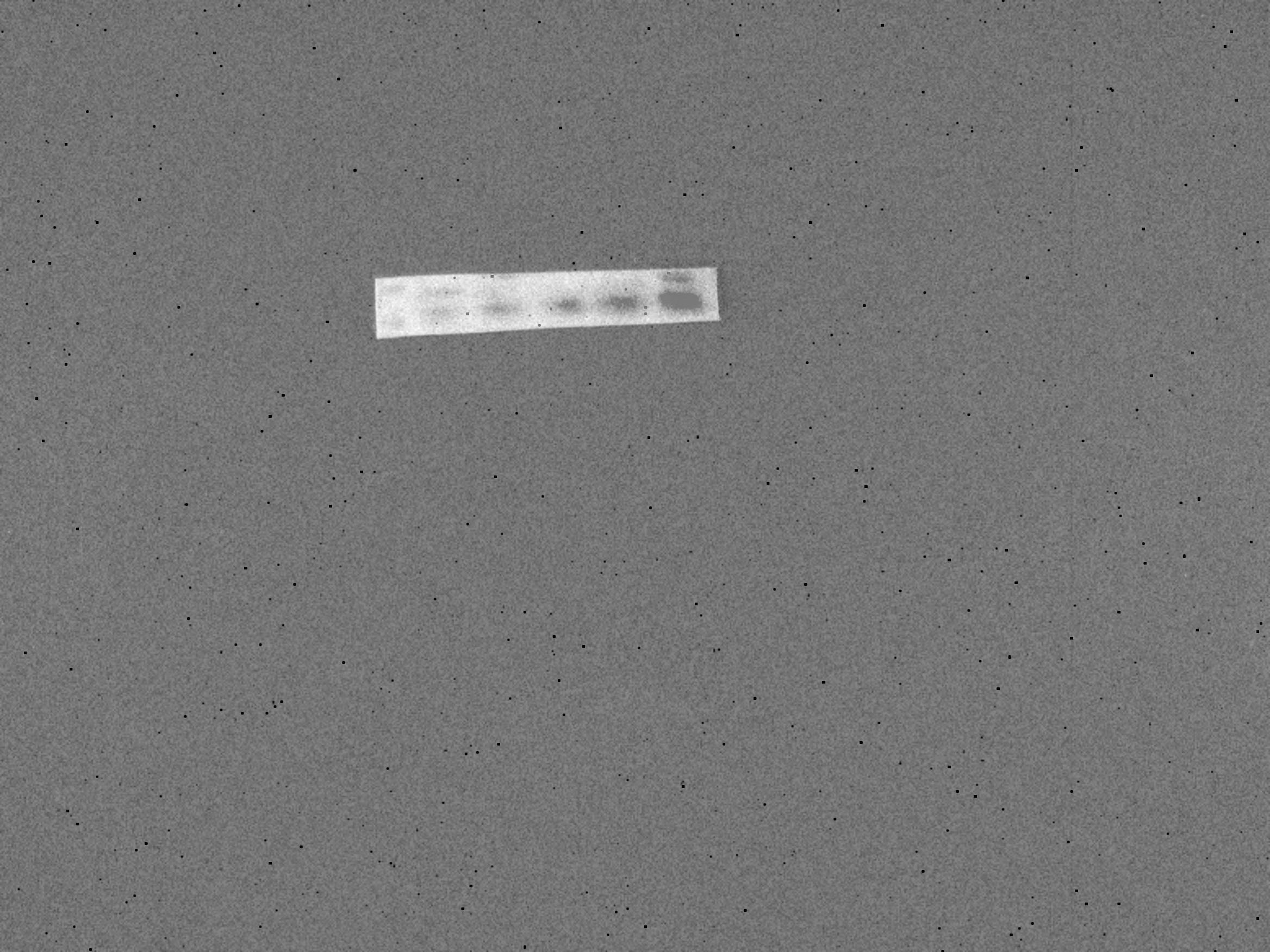

Supplement: Original Images for Blots.zip [file YRER_A_2313366_SM3875.zip › Original Images for Blots/Figure 4/Figure 4A/p38 signaling pathway/p-p38/Marker+p-p38.jpg]

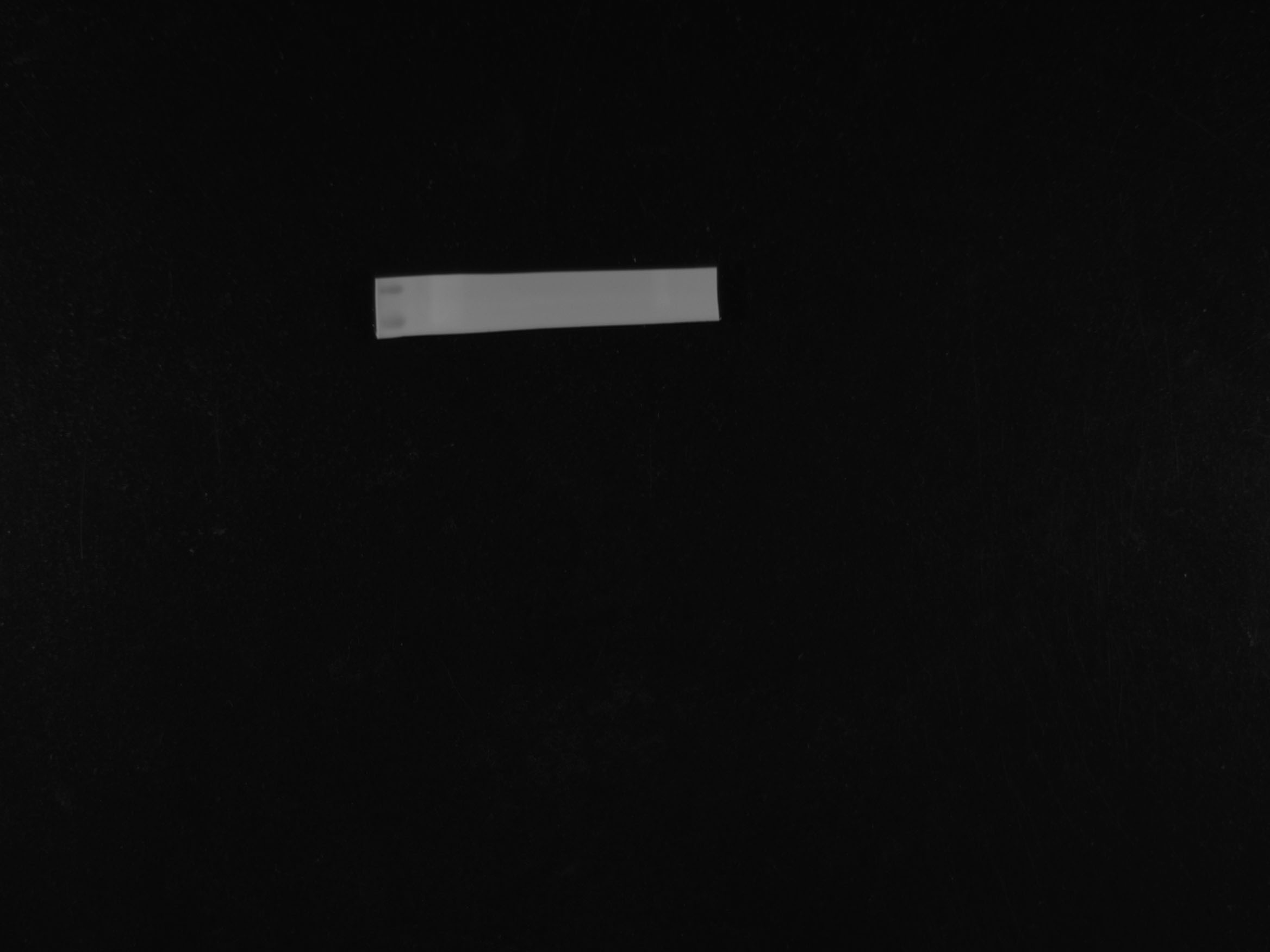

Supplement: Original Images for Blots.zip [file YRER_A_2313366_SM3875.zip › Original Images for Blots/Figure 4/Figure 4A/p38 signaling pathway/p-p38/Marker.jpg]

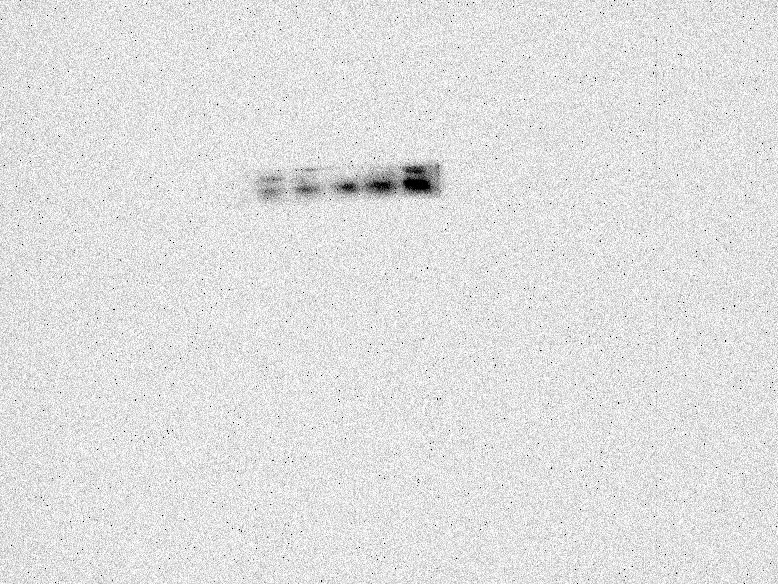

Supplement: Original Images for Blots.zip [file YRER_A_2313366_SM3875.zip › Original Images for Blots/Figure 4/Figure 4A/p38 signaling pathway/p-p38/p-p38.jpg]

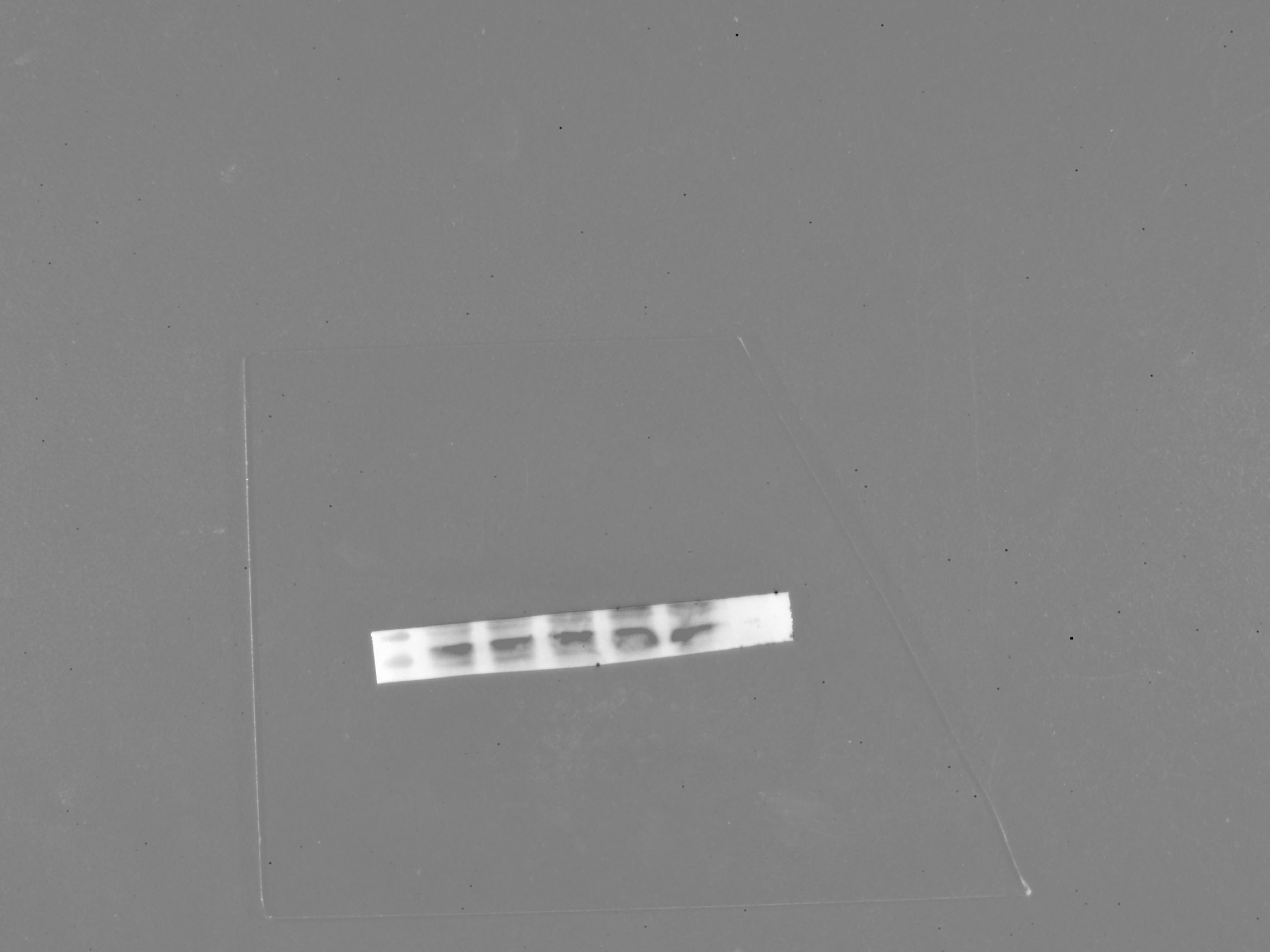

Supplement: Original Images for Blots.zip [file YRER_A_2313366_SM3875.zip › Original Images for Blots/Figure 4/Figure 4A/p38 signaling pathway/p38/Marker+p38.jpg]

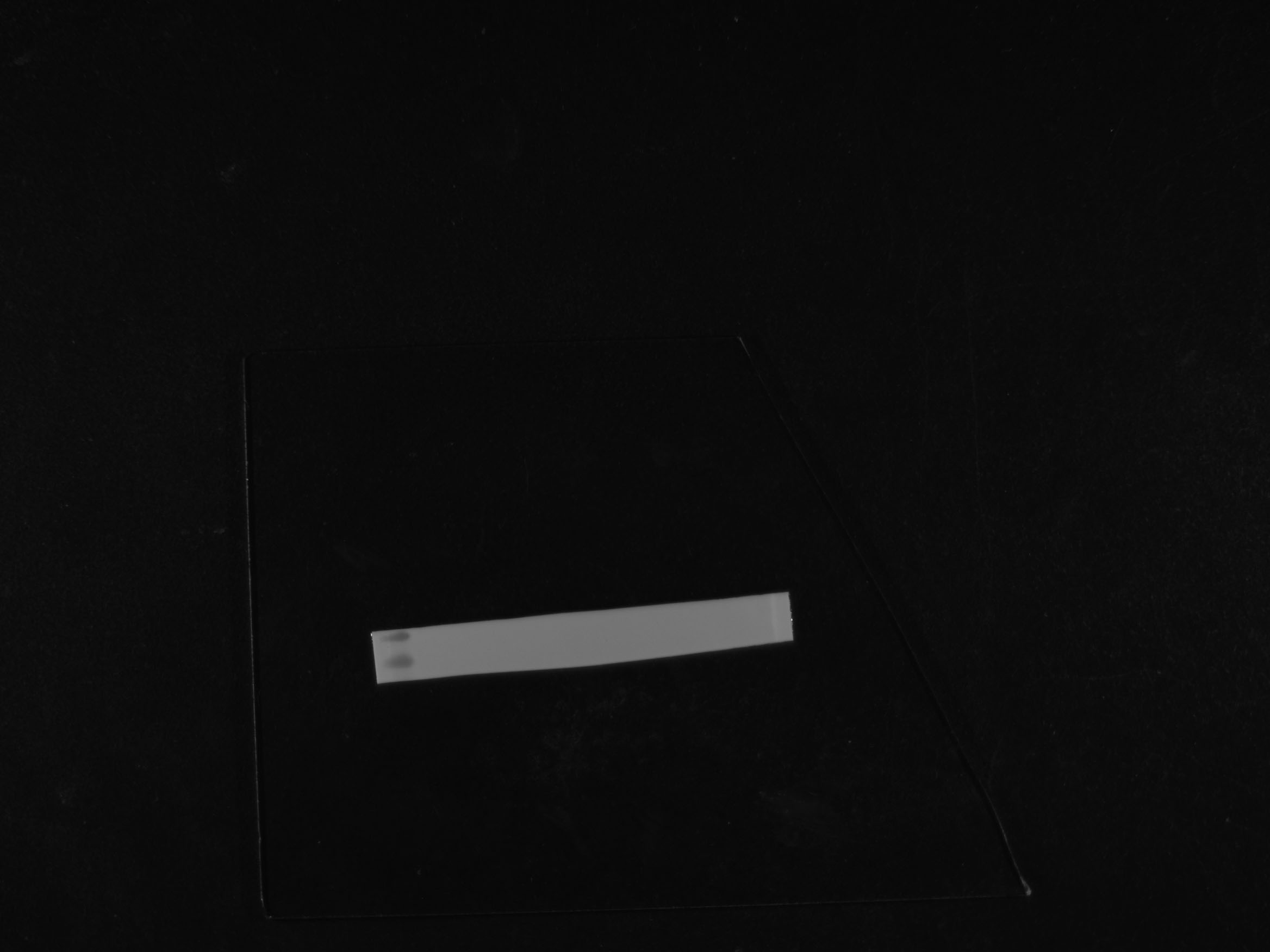

Supplement: Original Images for Blots.zip [file YRER_A_2313366_SM3875.zip › Original Images for Blots/Figure 4/Figure 4A/p38 signaling pathway/p38/Marker.jpg]

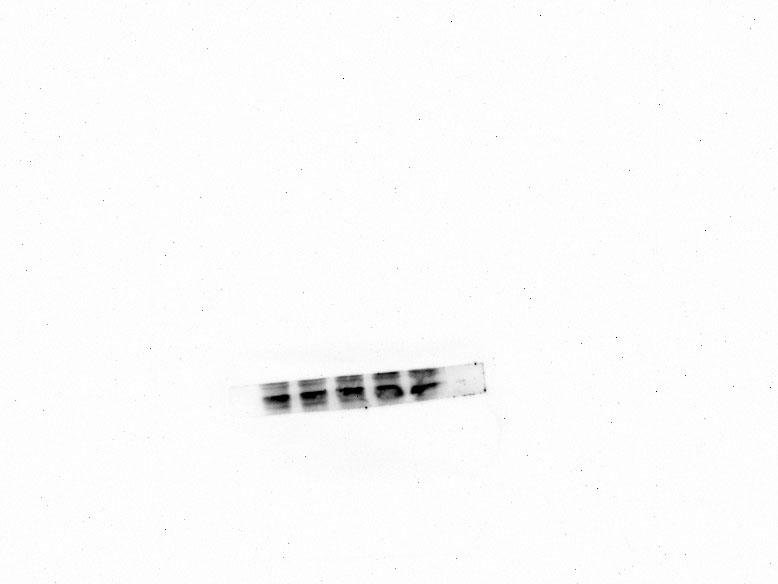

Supplement: Original Images for Blots.zip [file YRER_A_2313366_SM3875.zip › Original Images for Blots/Figure 4/Figure 4A/p38 signaling pathway/p38/p38.jpg]

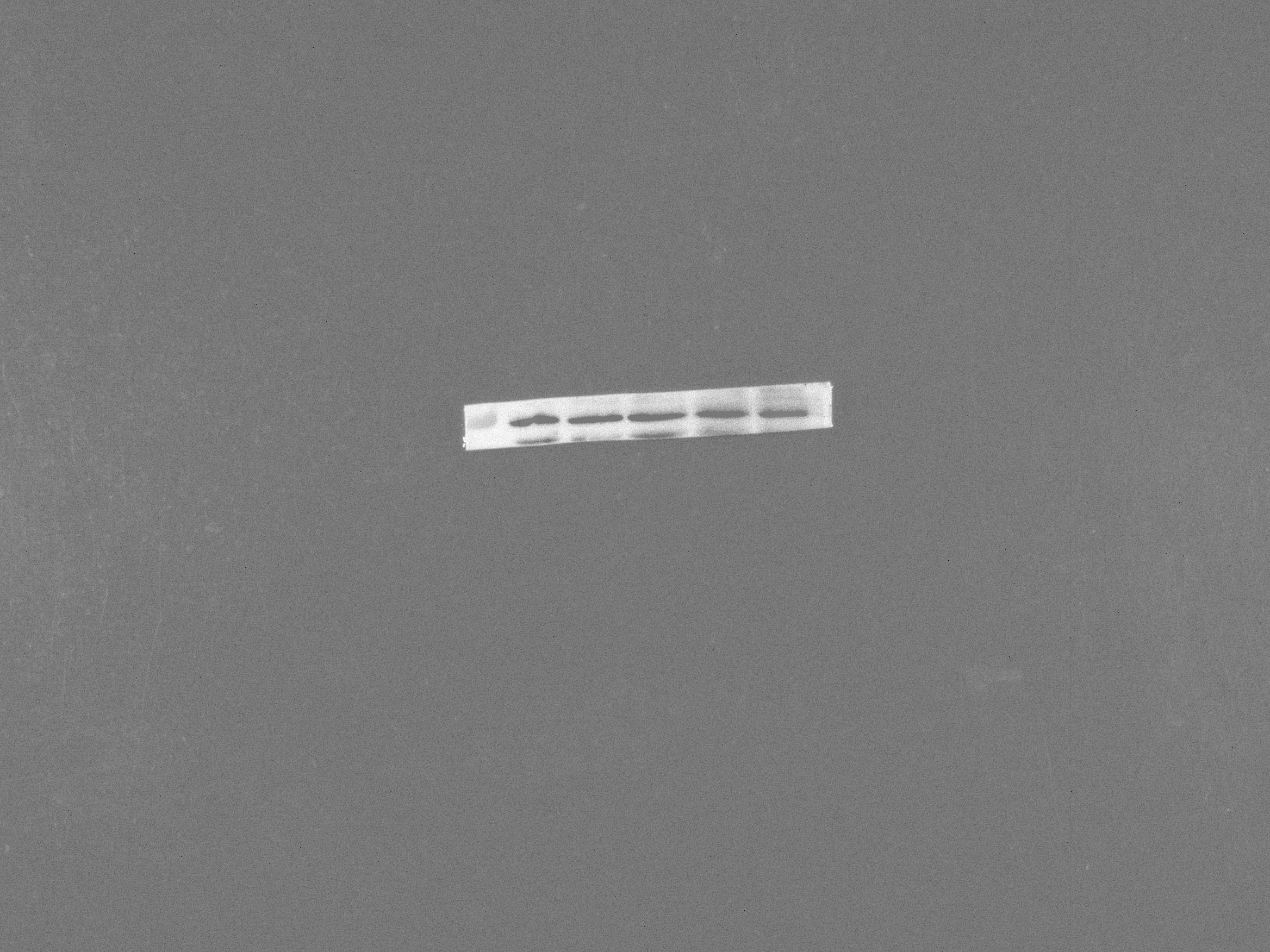

Supplement: Original Images for Blots.zip [file YRER_A_2313366_SM3875.zip › Original Images for Blots/Figure 4/Figure 4A/p38 signaling pathway/α-tubulin/Marker+α-tubulin.jpg]

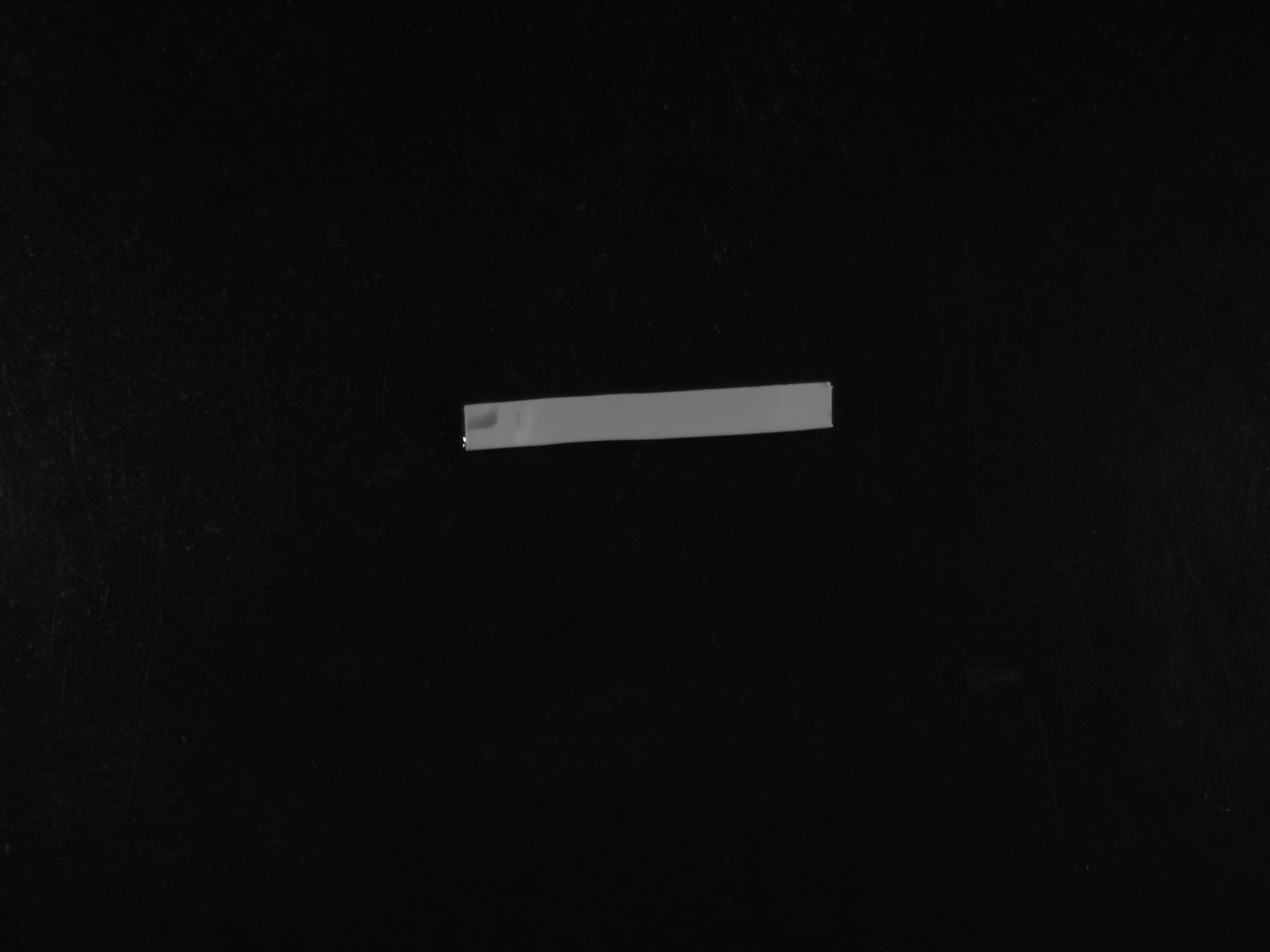

Supplement: Original Images for Blots.zip [file YRER_A_2313366_SM3875.zip › Original Images for Blots/Figure 4/Figure 4A/p38 signaling pathway/α-tubulin/Marker.jpg]

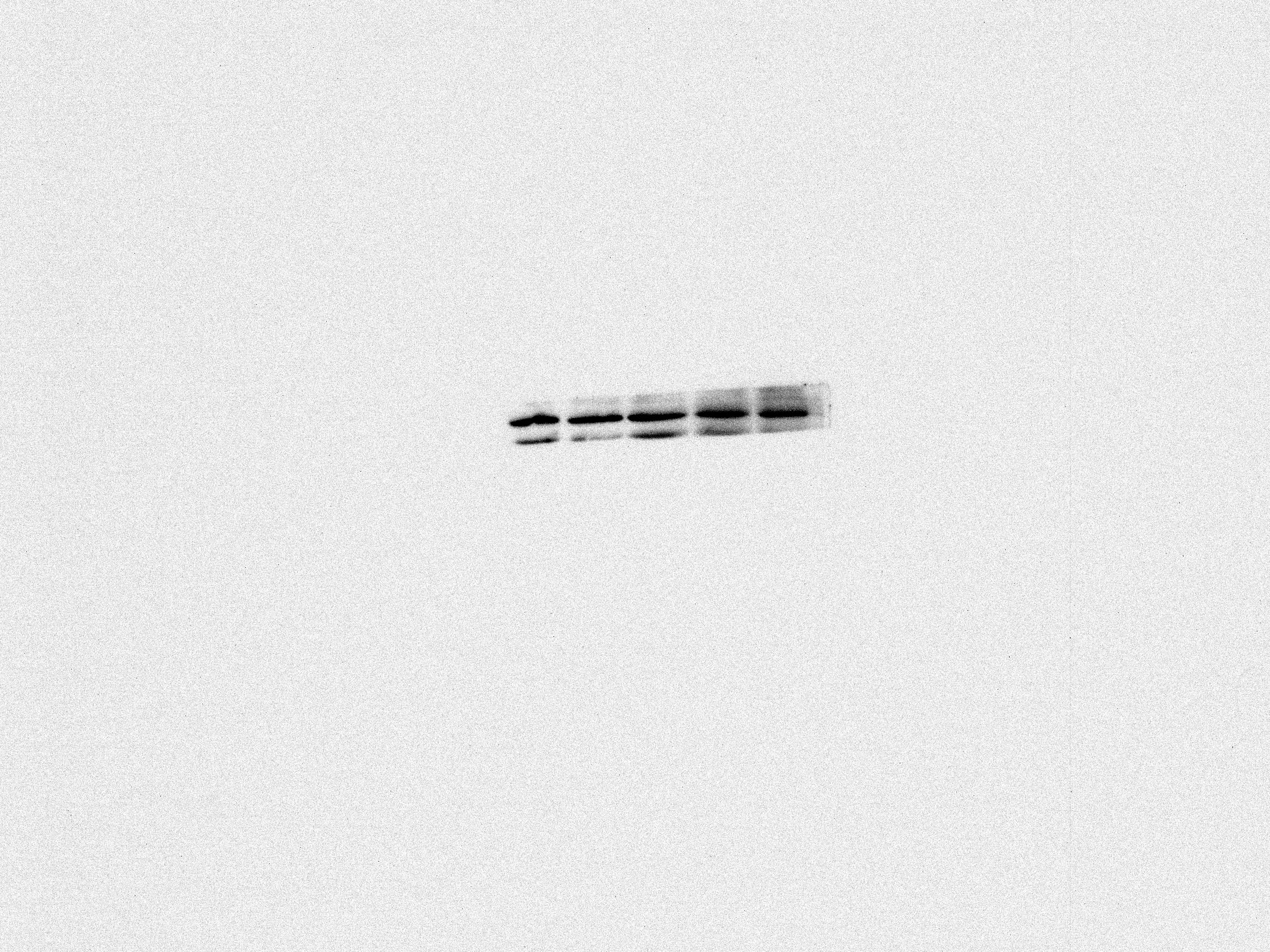

Supplement: Original Images for Blots.zip [file YRER_A_2313366_SM3875.zip › Original Images for Blots/Figure 4/Figure 4A/p38 signaling pathway/α-tubulin/α-tubulin.jpg]

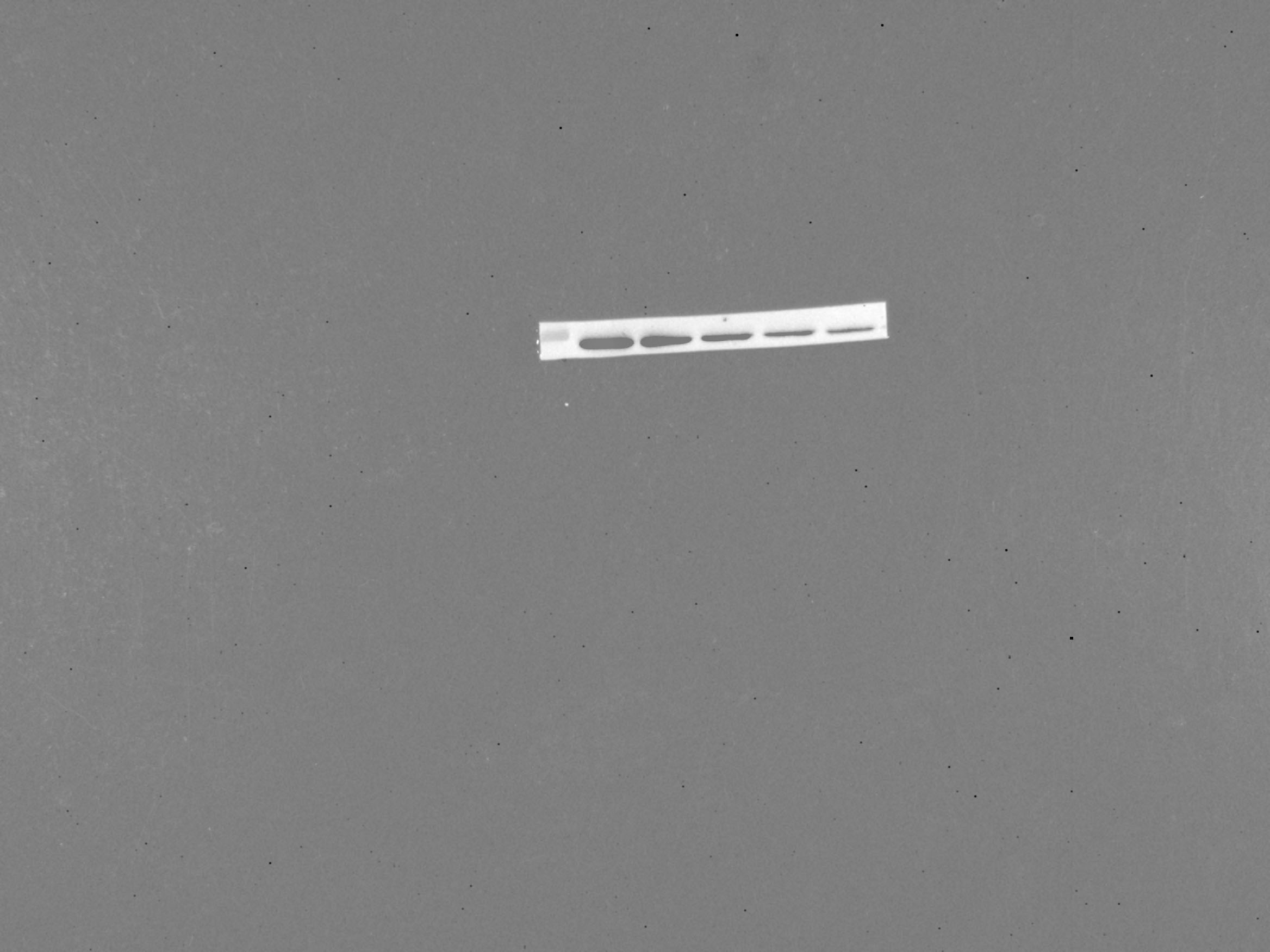

Supplement: Original Images for Blots.zip [file YRER_A_2313366_SM3875.zip › Original Images for Blots/Figure 4/Figure 4A/STAT3 signaling pathway/p-STAT3/Marker+p-STAT3.jpg]

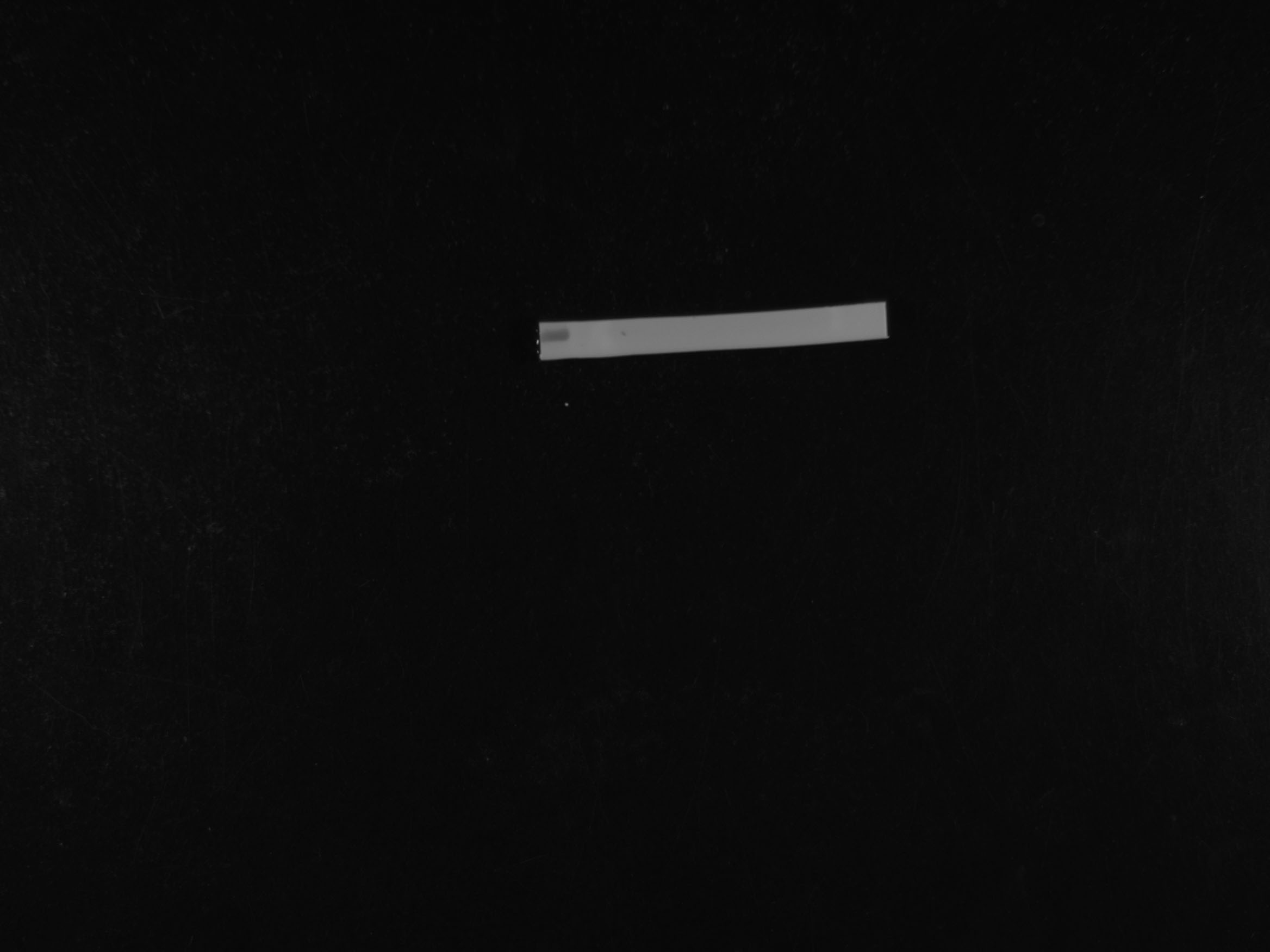

Supplement: Original Images for Blots.zip [file YRER_A_2313366_SM3875.zip › Original Images for Blots/Figure 4/Figure 4A/STAT3 signaling pathway/p-STAT3/Marker.jpg]

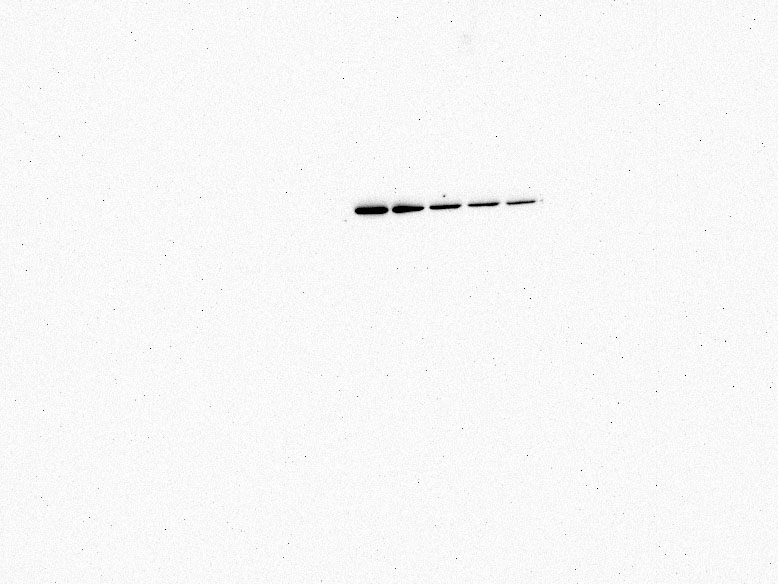

Supplement: Original Images for Blots.zip [file YRER_A_2313366_SM3875.zip › Original Images for Blots/Figure 4/Figure 4A/STAT3 signaling pathway/p-STAT3/p-STAT3.jpg]

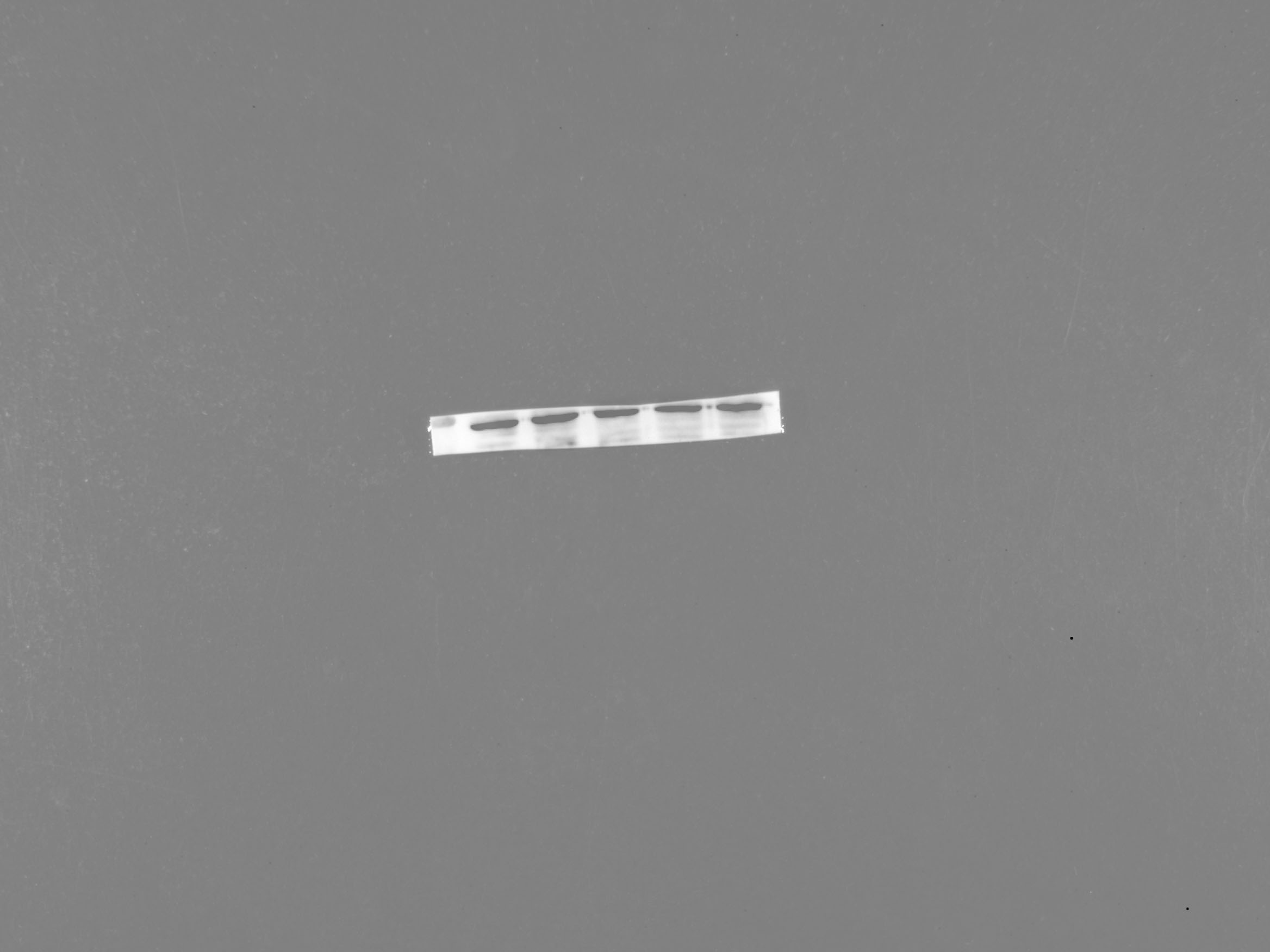

Supplement: Original Images for Blots.zip [file YRER_A_2313366_SM3875.zip › Original Images for Blots/Figure 4/Figure 4A/STAT3 signaling pathway/STAT3/Marker+STAT3.jpg]

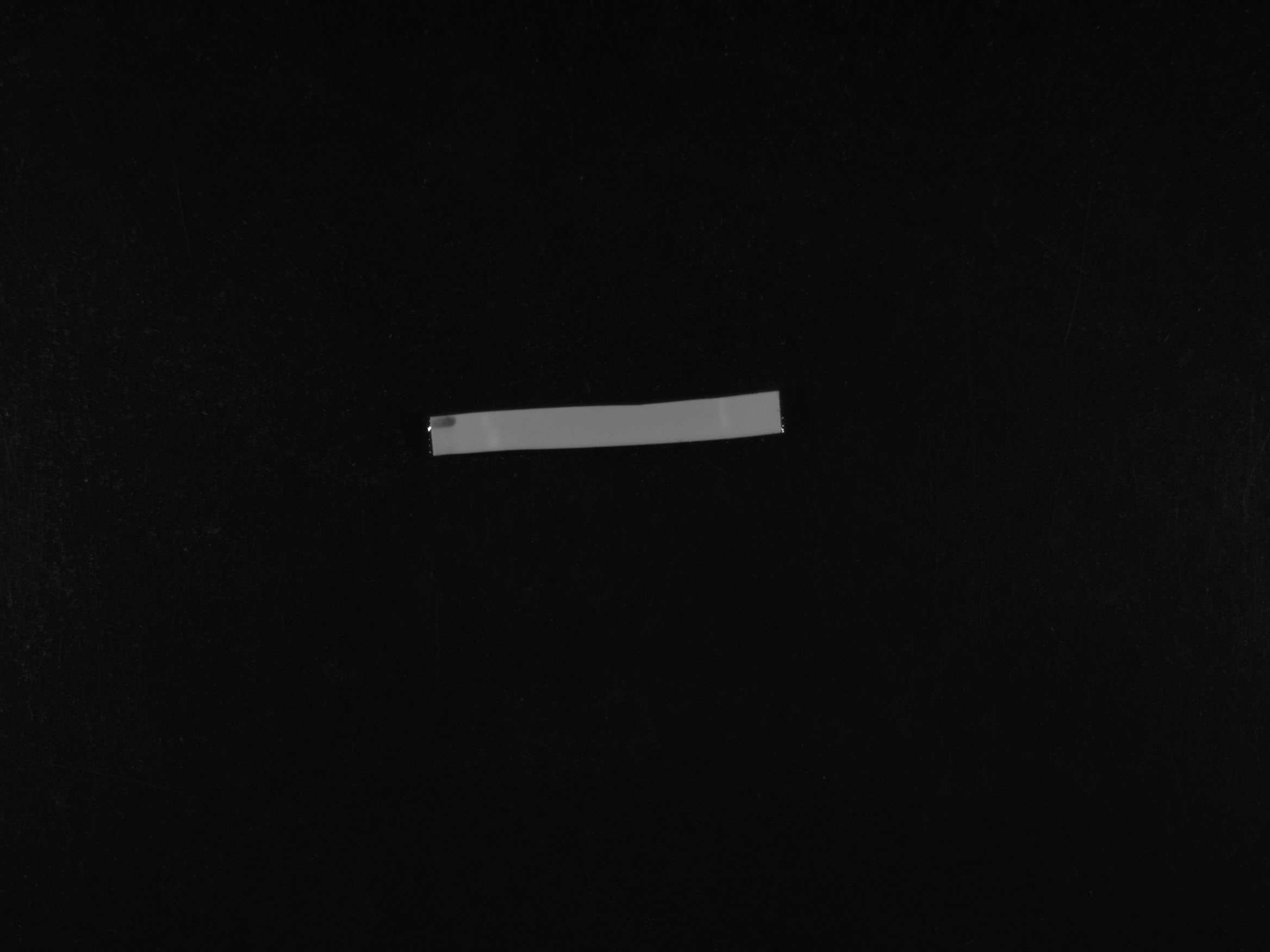

Supplement: Original Images for Blots.zip [file YRER_A_2313366_SM3875.zip › Original Images for Blots/Figure 4/Figure 4A/STAT3 signaling pathway/STAT3/Marker.jpg]

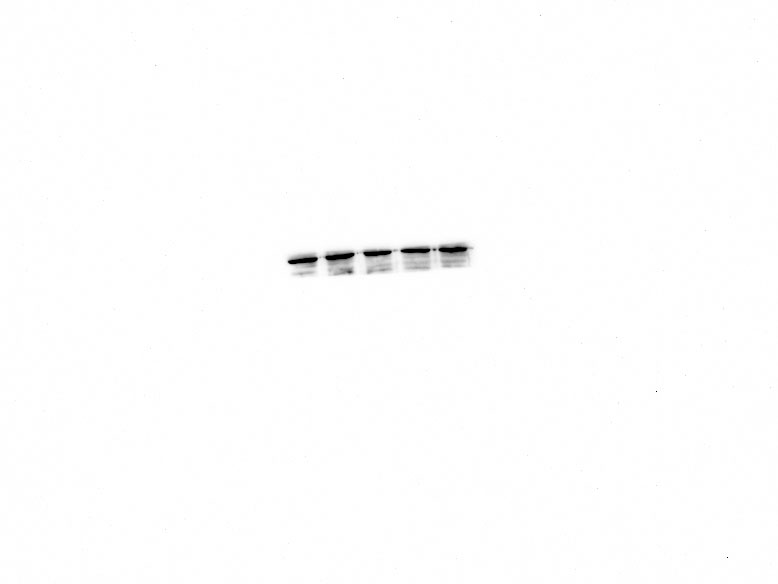

Supplement: Original Images for Blots.zip [file YRER_A_2313366_SM3875.zip › Original Images for Blots/Figure 4/Figure 4A/STAT3 signaling pathway/STAT3/STAT3.jpg]

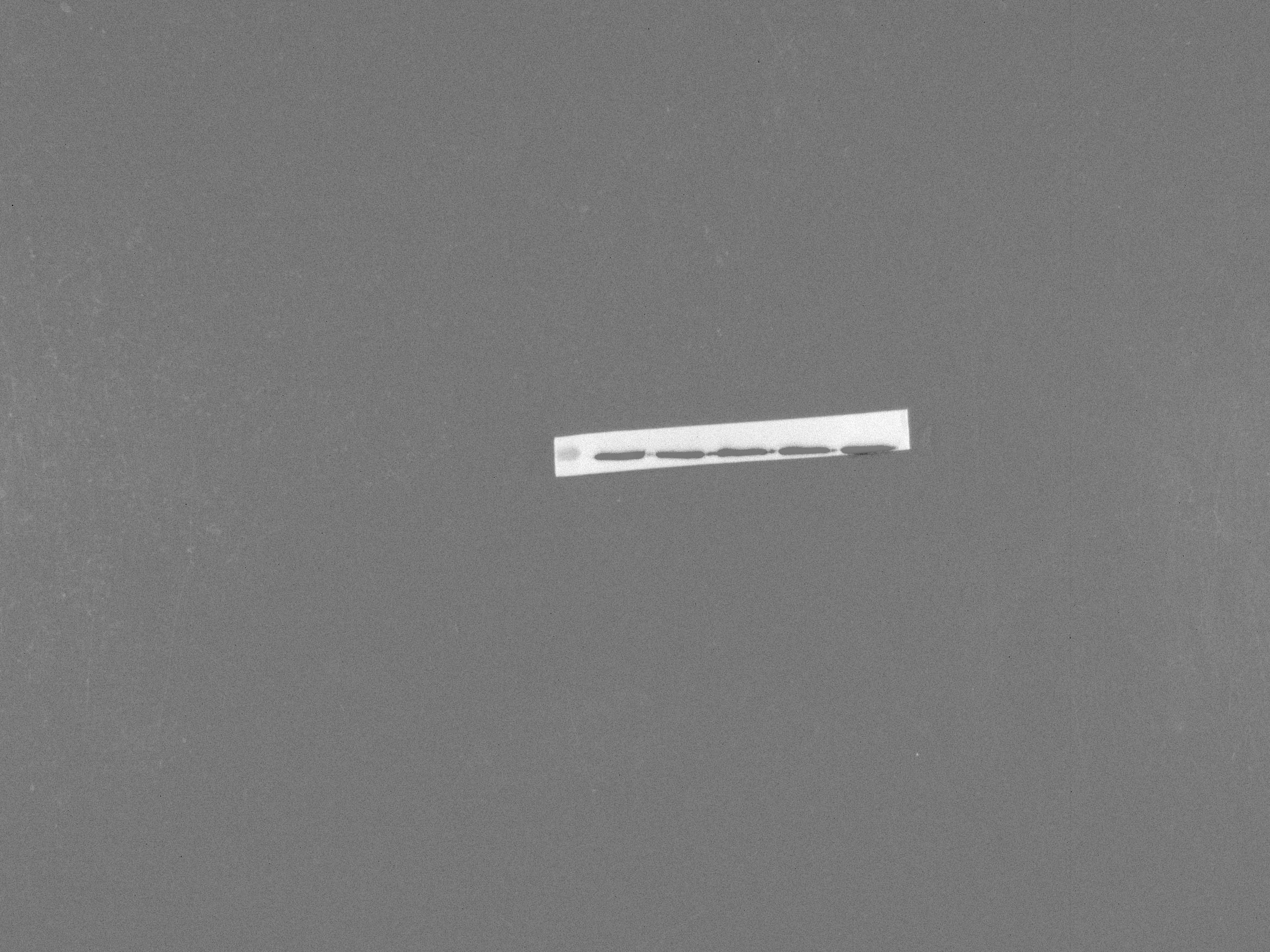

Supplement: Original Images for Blots.zip [file YRER_A_2313366_SM3875.zip › Original Images for Blots/Figure 4/Figure 4A/STAT3 signaling pathway/α-tubulin/Marker+α-tubulin.jpg]

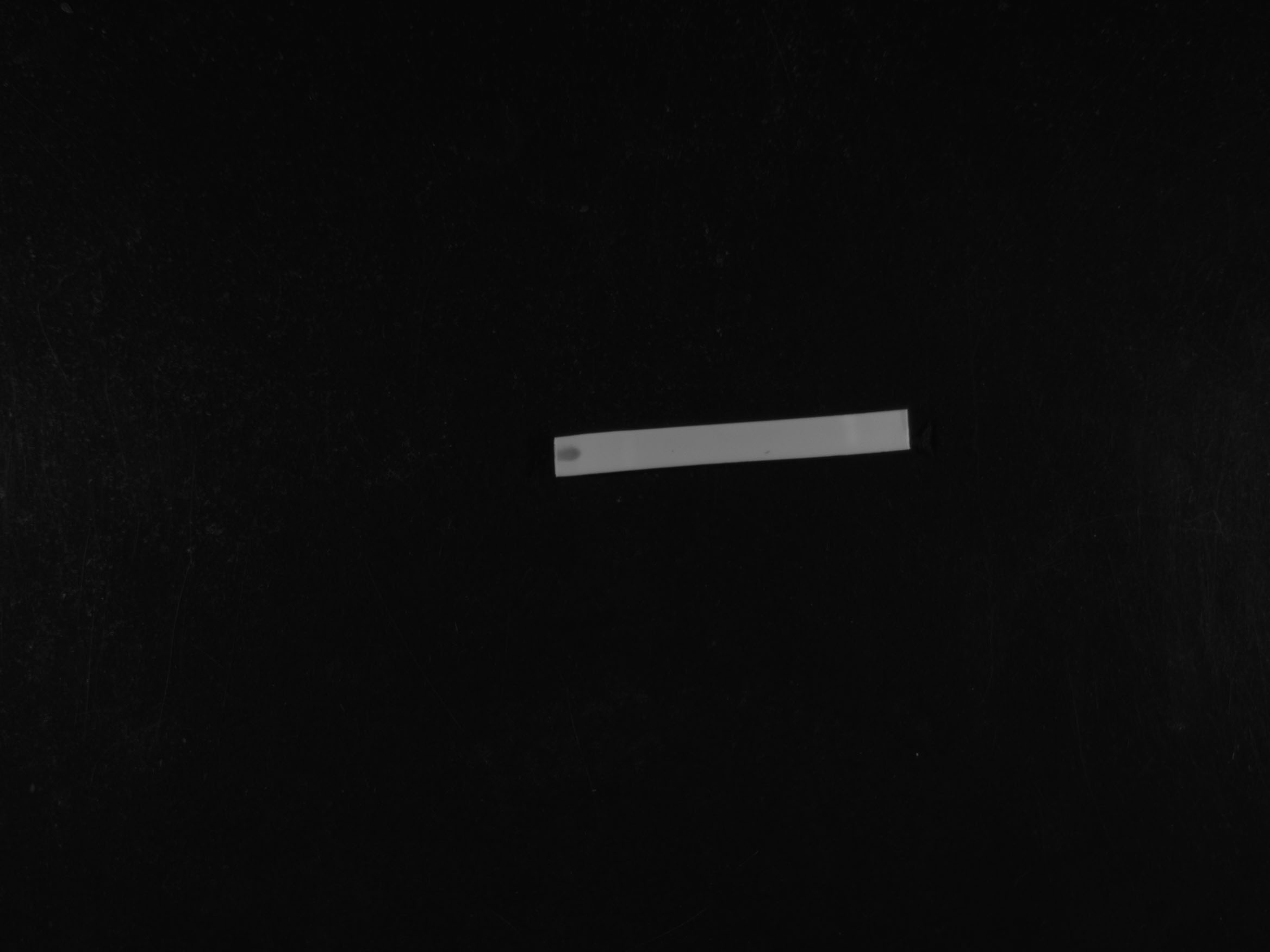

Supplement: Original Images for Blots.zip [file YRER_A_2313366_SM3875.zip › Original Images for Blots/Figure 4/Figure 4A/STAT3 signaling pathway/α-tubulin/Marker.jpg]

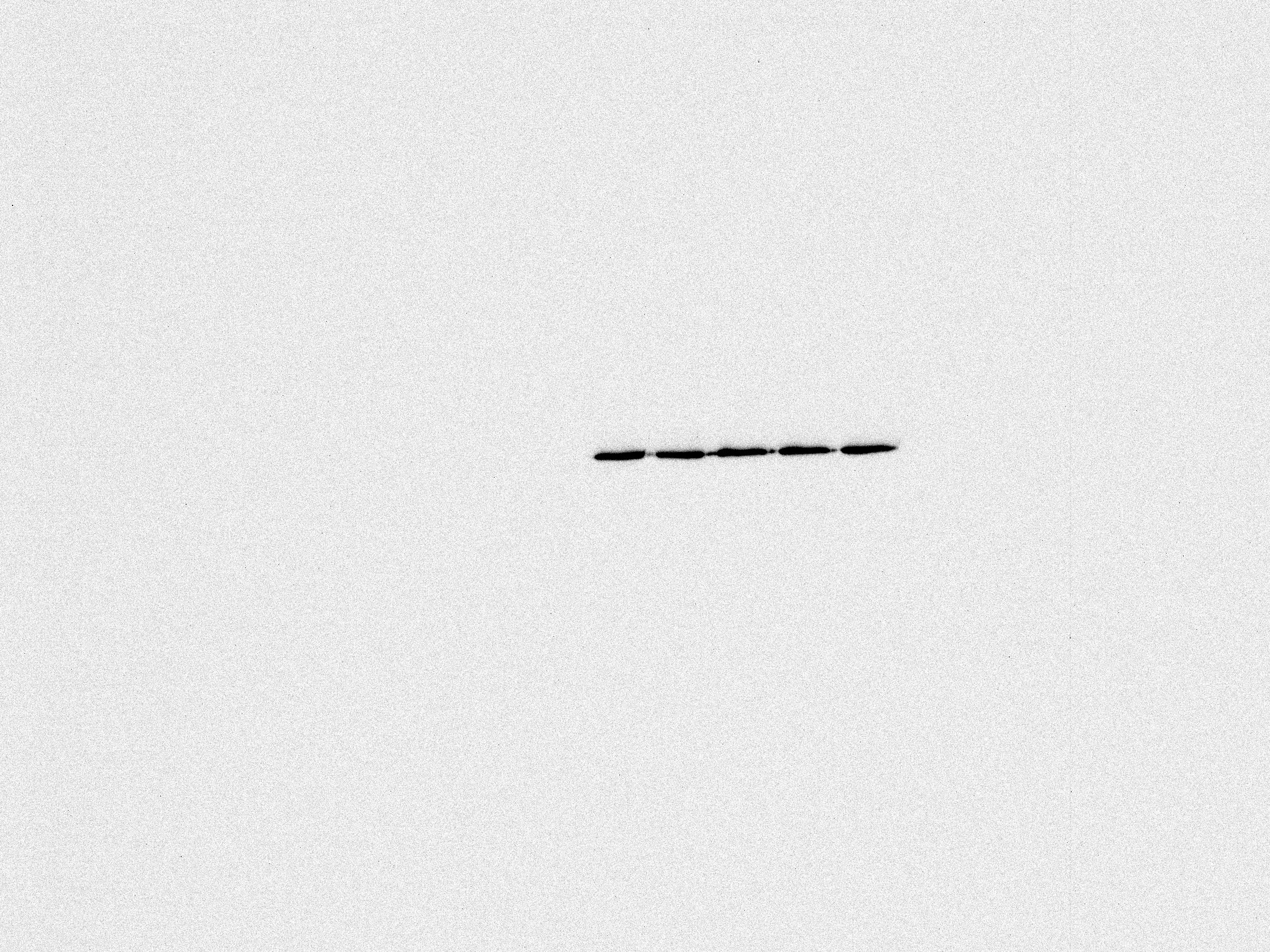

Supplement: Original Images for Blots.zip [file YRER_A_2313366_SM3875.zip › Original Images for Blots/Figure 4/Figure 4A/STAT3 signaling pathway/α-tubulin/α-tubulin.jpg]

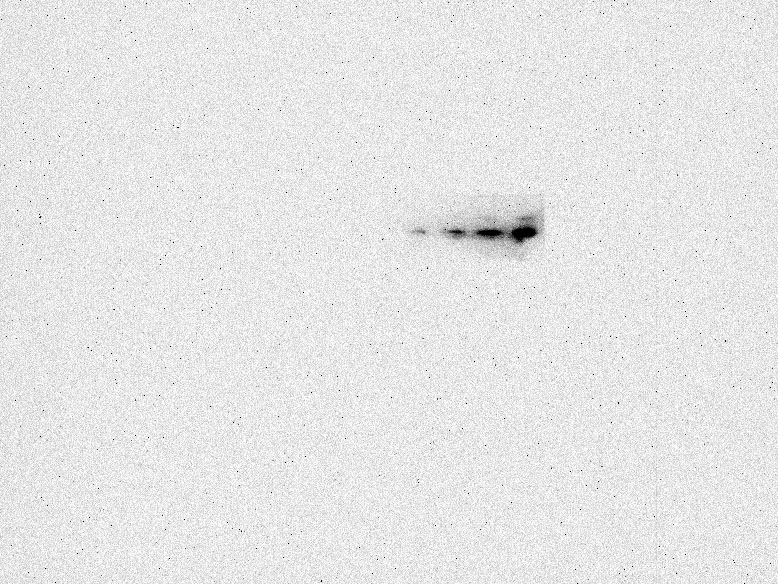

Supplement: Original Images for Blots.zip [file YRER_A_2313366_SM3875.zip › Original Images for Blots/Figure 4/Figure 4B/ERK signaling pathway/cle-caspase-3/cle-caspase-3.jpg]

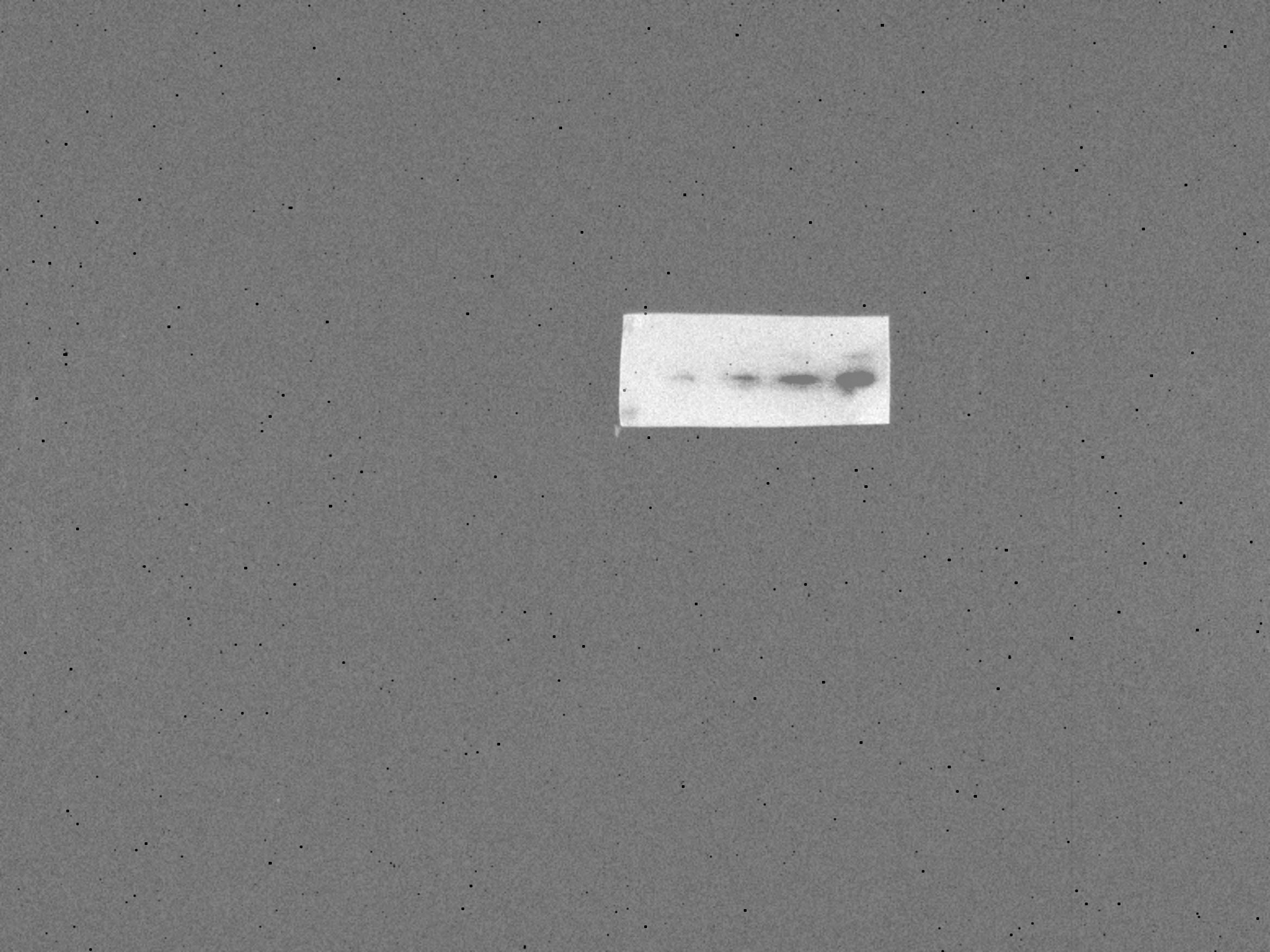

Supplement: Original Images for Blots.zip [file YRER_A_2313366_SM3875.zip › Original Images for Blots/Figure 4/Figure 4B/ERK signaling pathway/cle-caspase-3/Marker+cle-caspase-3.jpg]

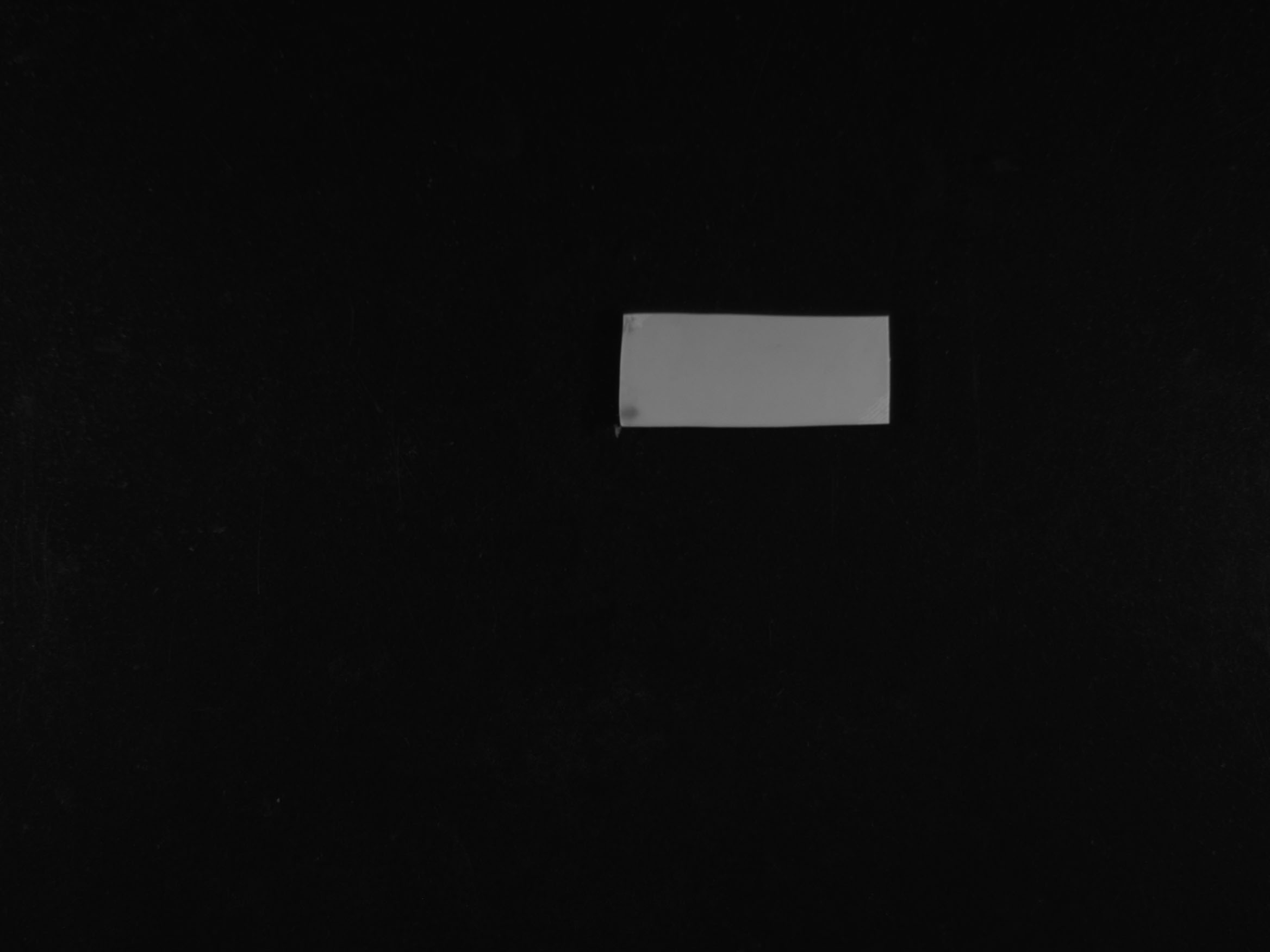

Supplement: Original Images for Blots.zip [file YRER_A_2313366_SM3875.zip › Original Images for Blots/Figure 4/Figure 4B/ERK signaling pathway/cle-caspase-3/Marker.jpg]

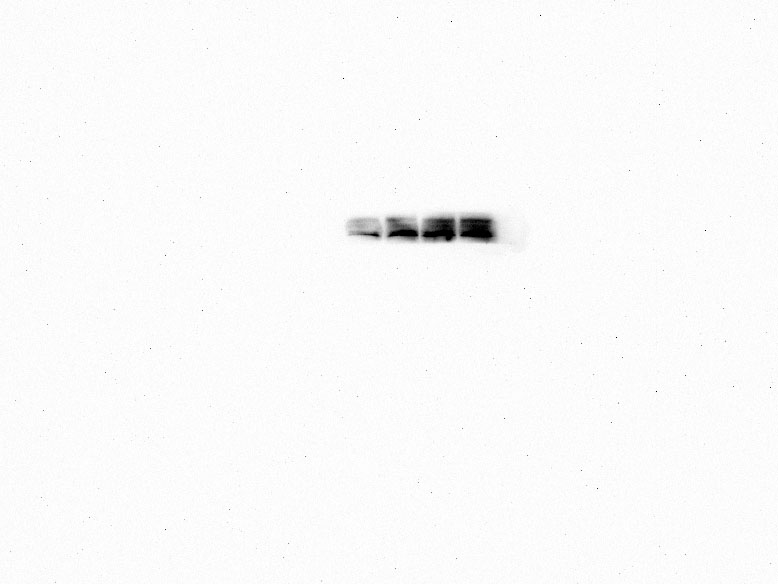

Supplement: Original Images for Blots.zip [file YRER_A_2313366_SM3875.zip › Original Images for Blots/Figure 4/Figure 4B/ERK signaling pathway/cle-PARP/cle-PARP.jpg]

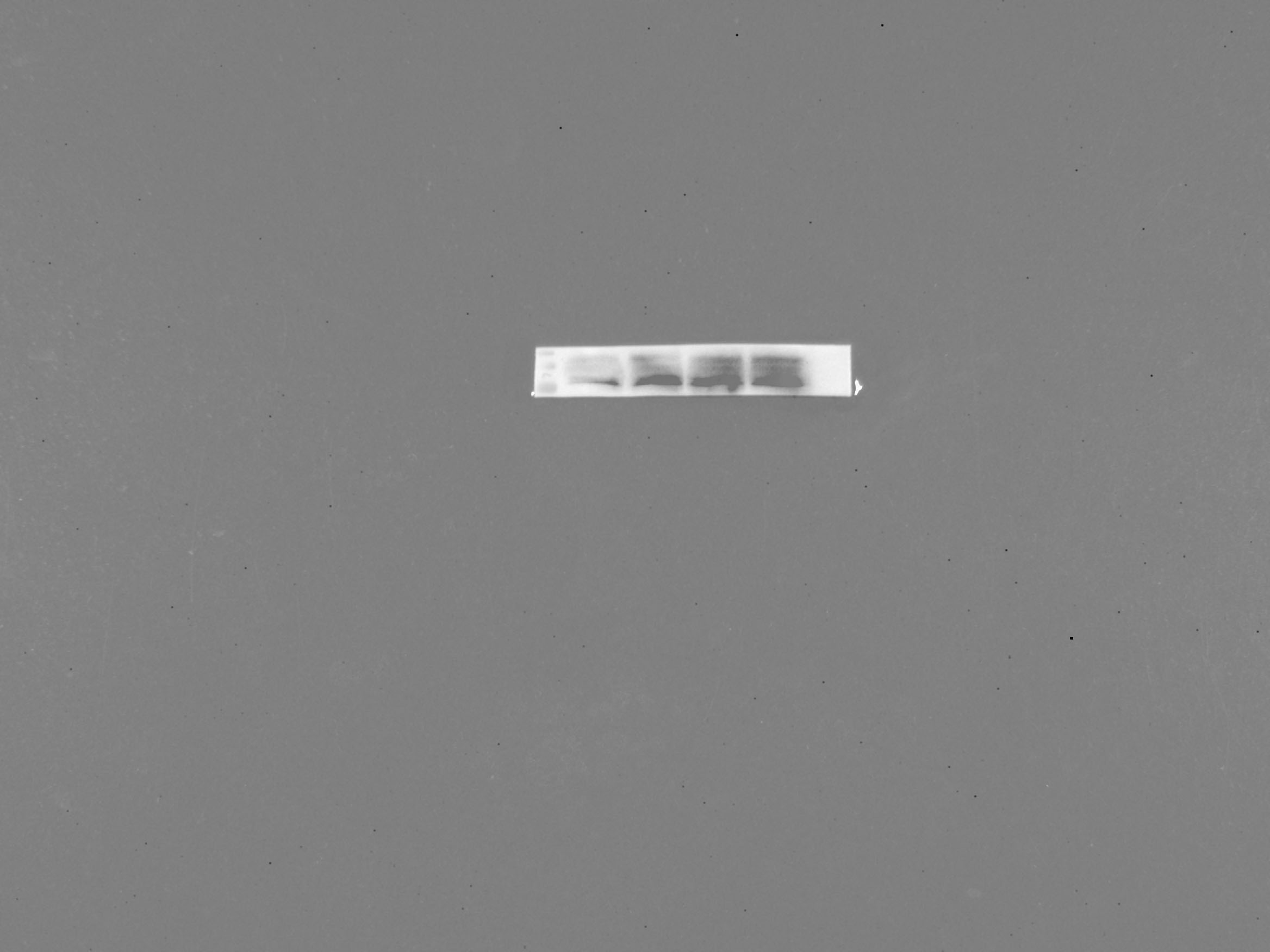

Supplement: Original Images for Blots.zip [file YRER_A_2313366_SM3875.zip › Original Images for Blots/Figure 4/Figure 4B/ERK signaling pathway/cle-PARP/Marker+cle-PARP.jpg]

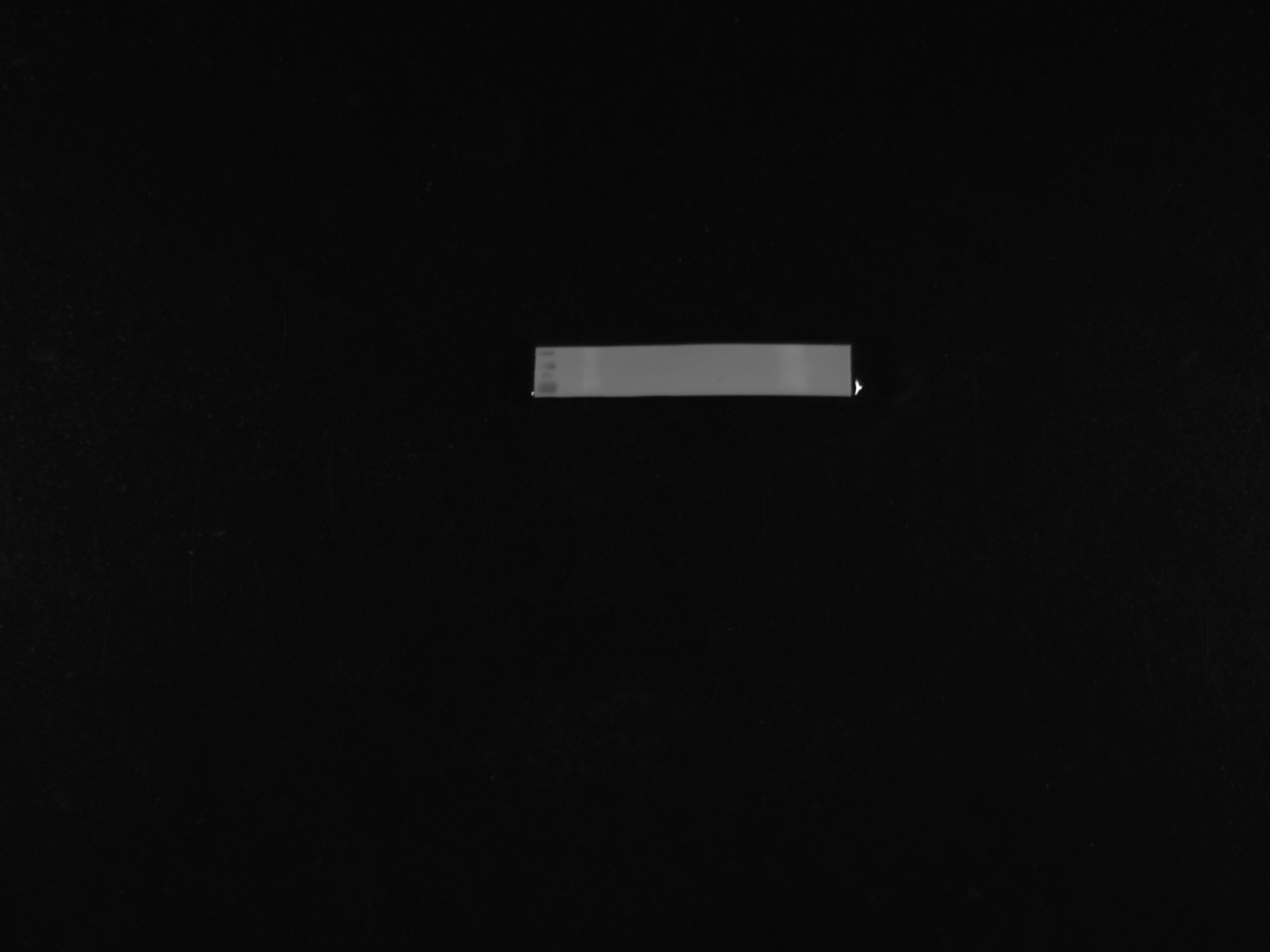

Supplement: Original Images for Blots.zip [file YRER_A_2313366_SM3875.zip › Original Images for Blots/Figure 4/Figure 4B/ERK signaling pathway/cle-PARP/Marker.jpg]

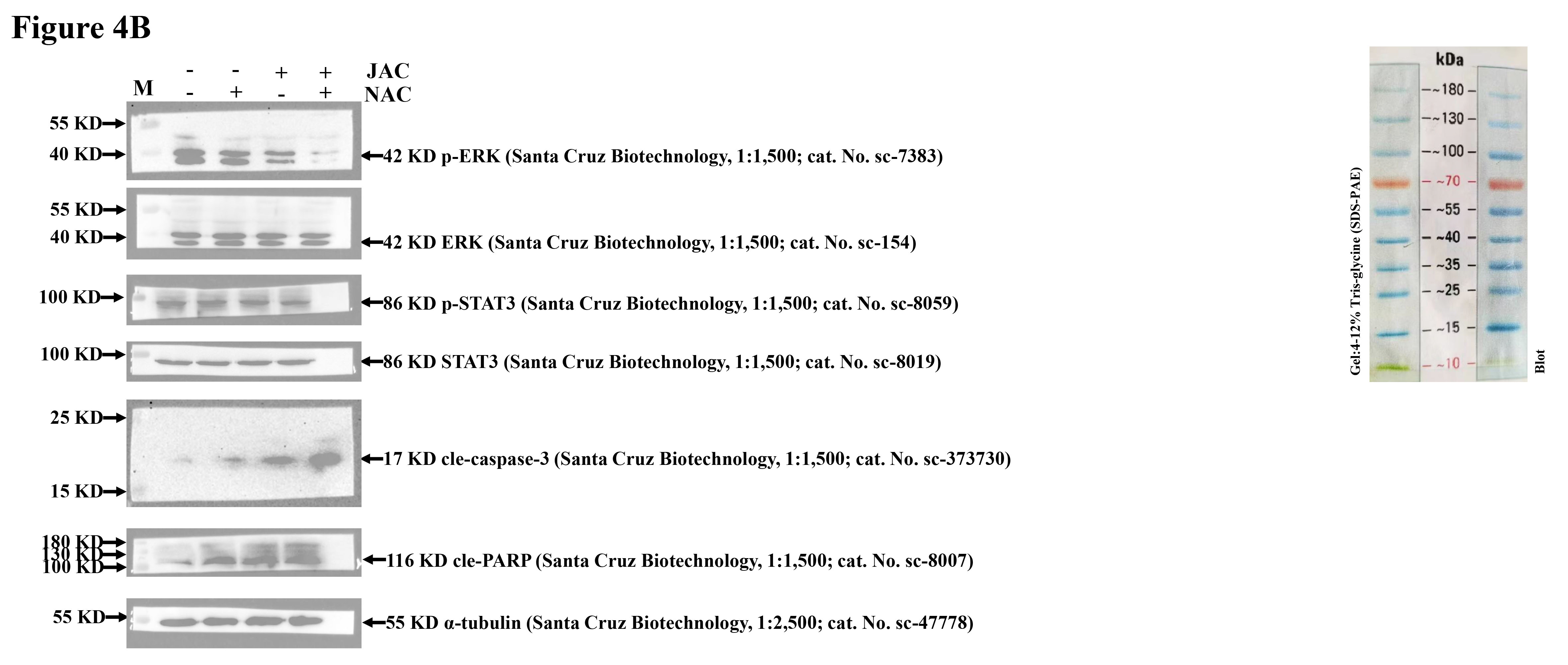

Supplement: Original Images for Blots.zip [file YRER_A_2313366_SM3875.zip › Original Images for Blots/Figure 4/Figure 4B/ERK signaling pathway/ERK signaling pathway.jpg]

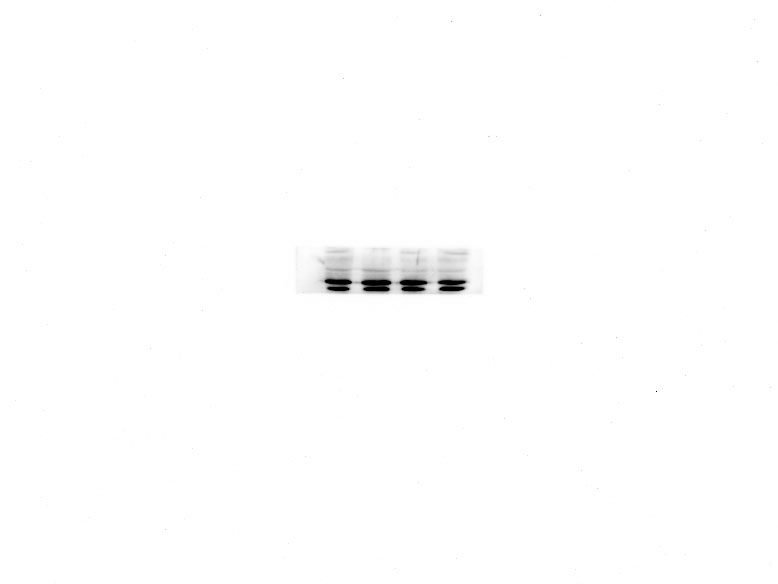

Supplement: Original Images for Blots.zip [file YRER_A_2313366_SM3875.zip › Original Images for Blots/Figure 4/Figure 4B/ERK signaling pathway/ERK/ERK.jpg]

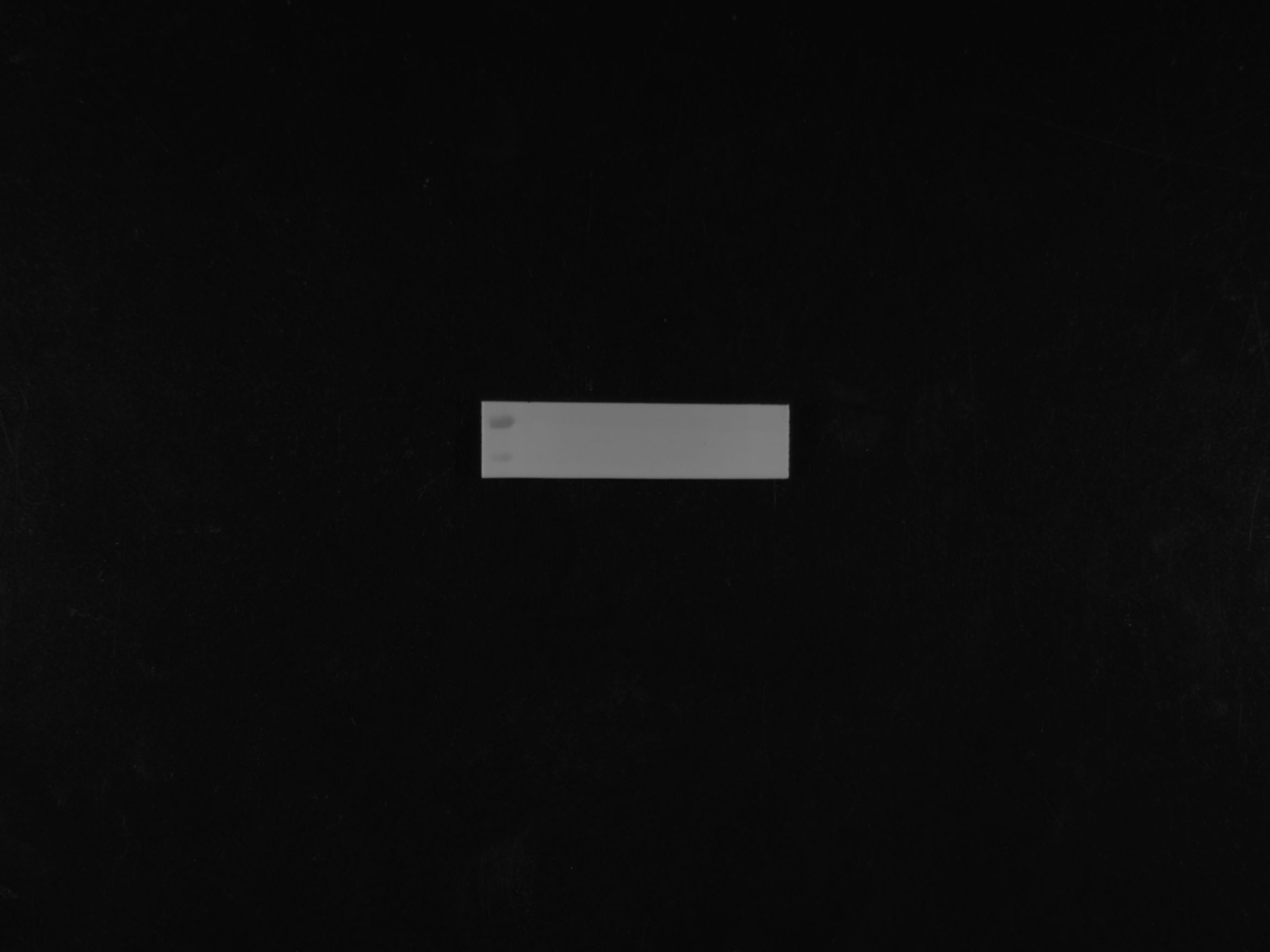

Supplement: Original Images for Blots.zip [file YRER_A_2313366_SM3875.zip › Original Images for Blots/Figure 4/Figure 4B/ERK signaling pathway/ERK/Marker .jpg]

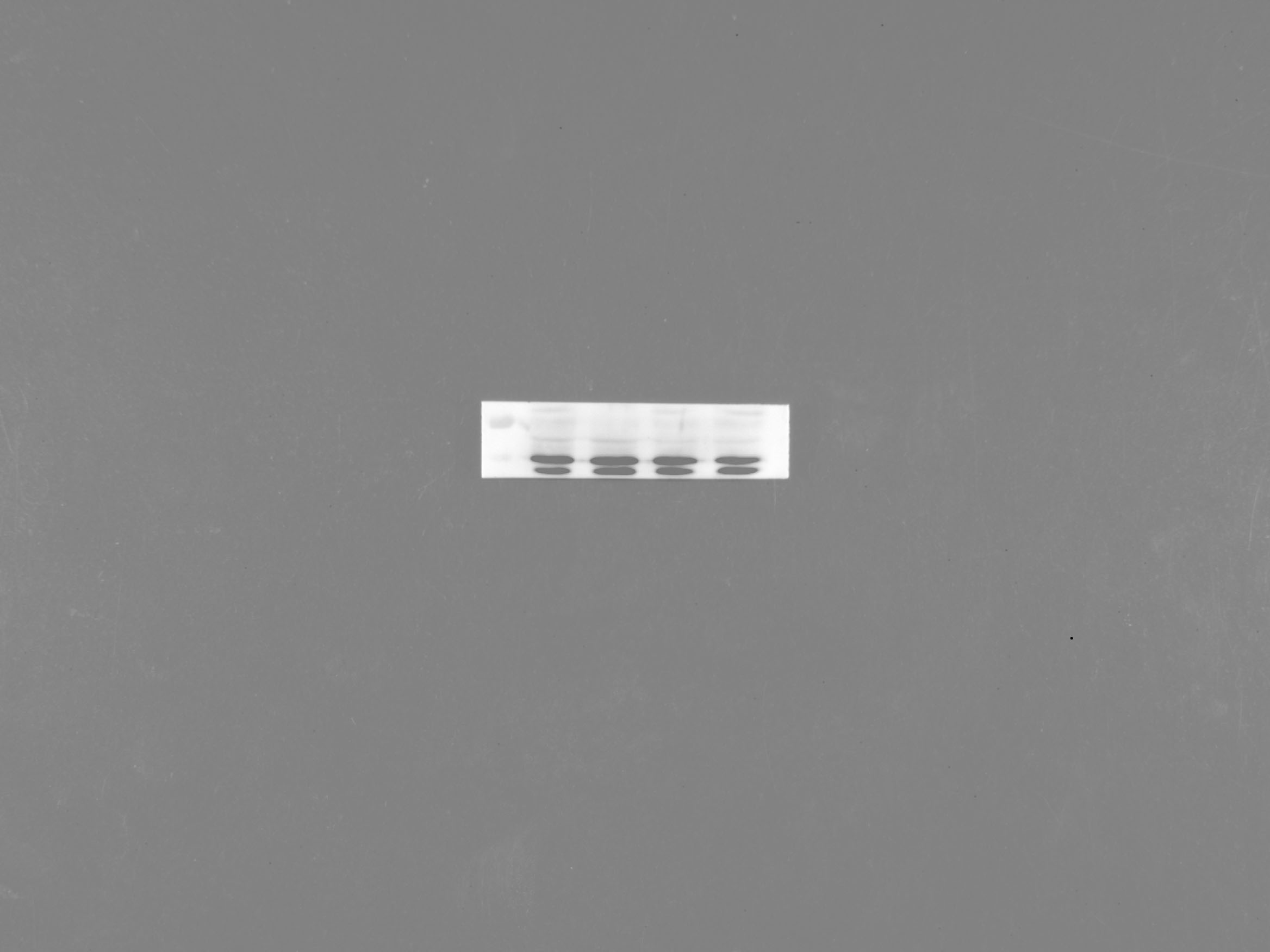

Supplement: Original Images for Blots.zip [file YRER_A_2313366_SM3875.zip › Original Images for Blots/Figure 4/Figure 4B/ERK signaling pathway/ERK/Marker+ERK.jpg]

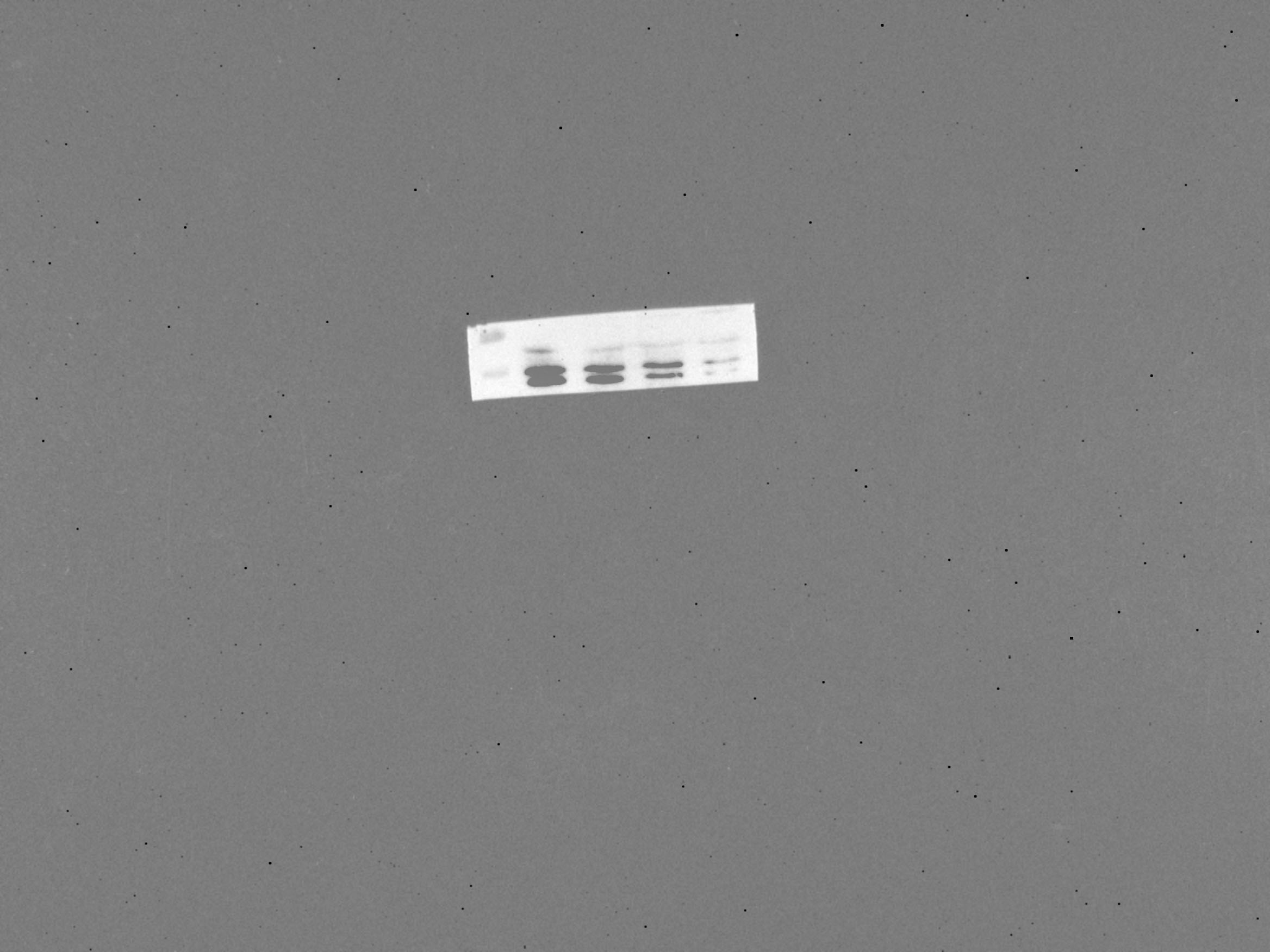

Supplement: Original Images for Blots.zip [file YRER_A_2313366_SM3875.zip › Original Images for Blots/Figure 4/Figure 4B/ERK signaling pathway/p-ERK/Marker+p-ERK.jpg]

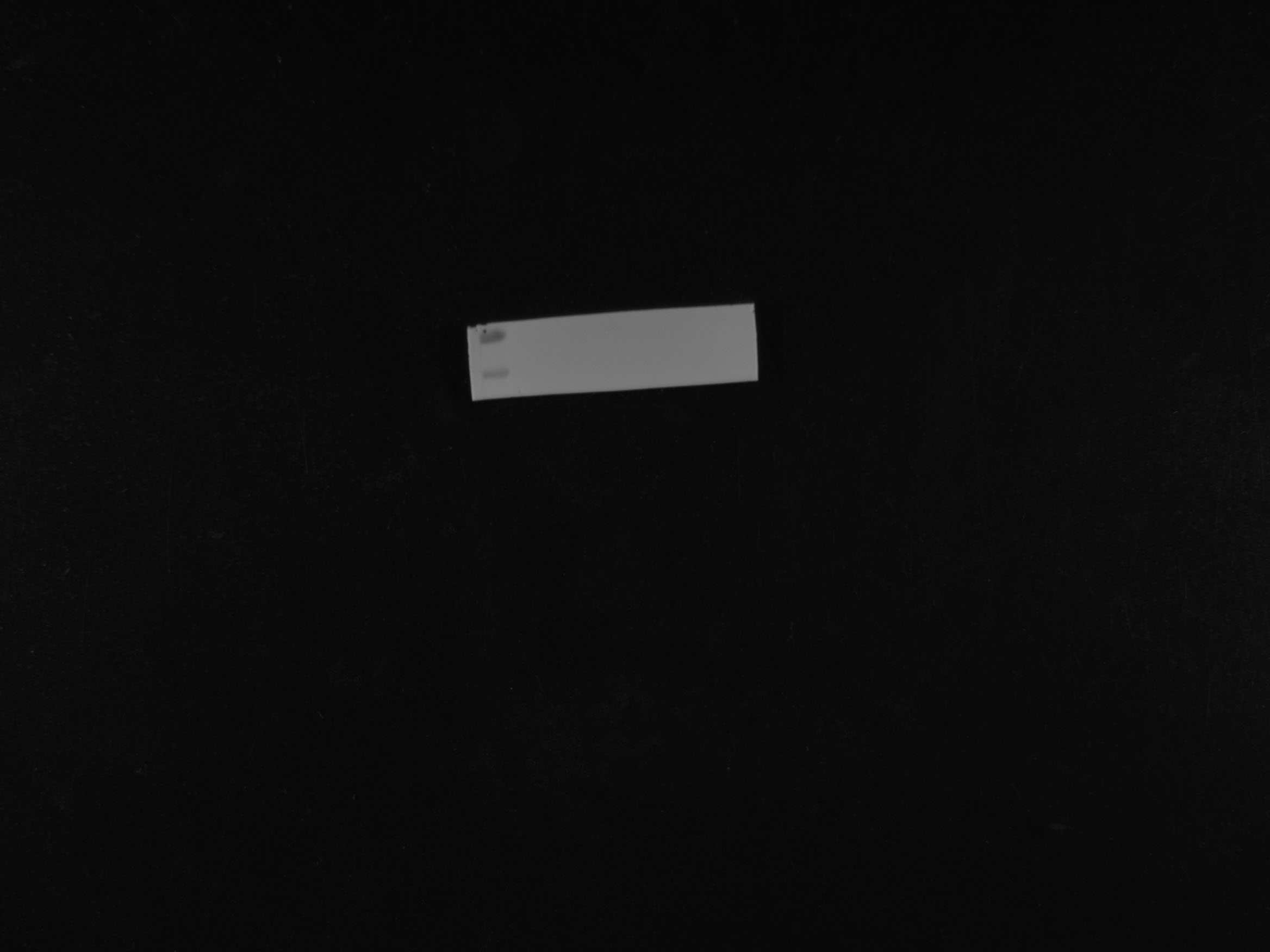

Supplement: Original Images for Blots.zip [file YRER_A_2313366_SM3875.zip › Original Images for Blots/Figure 4/Figure 4B/ERK signaling pathway/p-ERK/Marker.jpg]

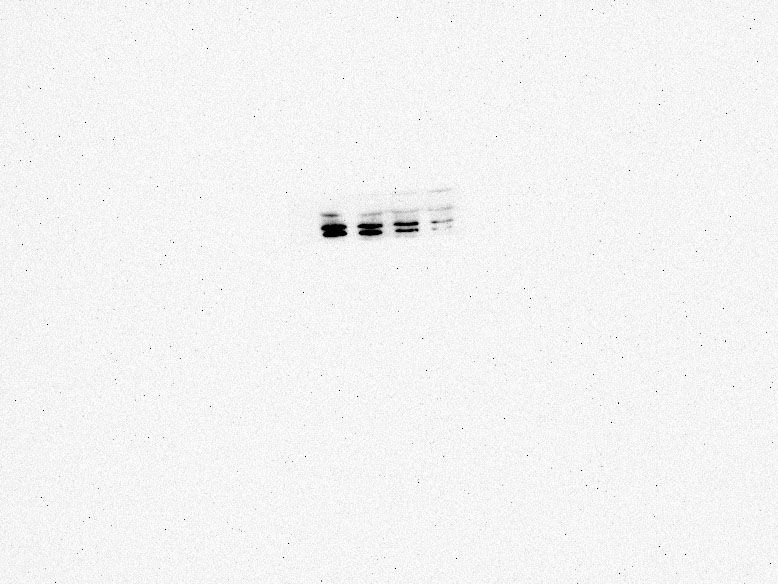

Supplement: Original Images for Blots.zip [file YRER_A_2313366_SM3875.zip › Original Images for Blots/Figure 4/Figure 4B/ERK signaling pathway/p-ERK/p-ERK.jpg]

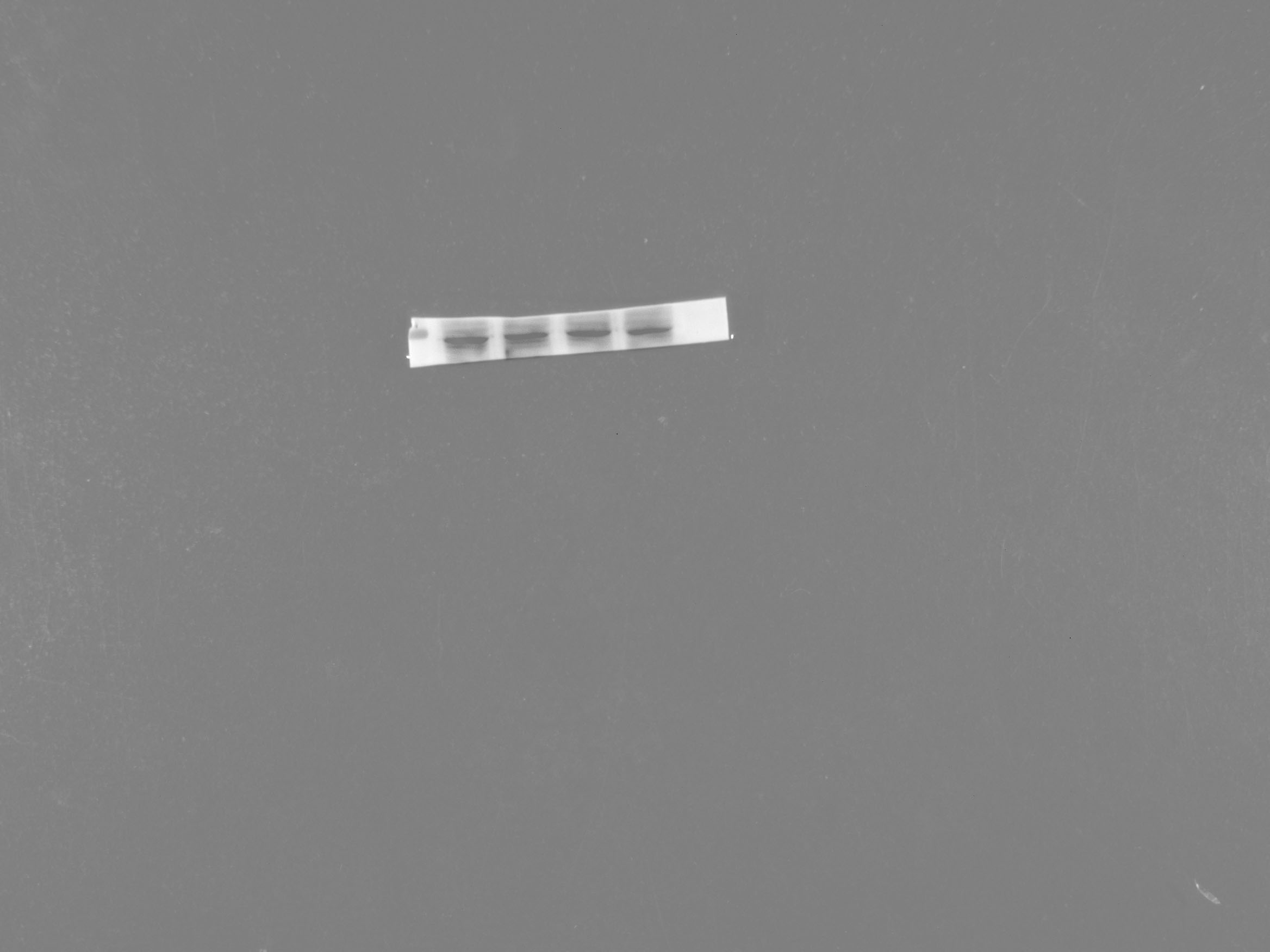

Supplement: Original Images for Blots.zip [file YRER_A_2313366_SM3875.zip › Original Images for Blots/Figure 4/Figure 4B/ERK signaling pathway/p-STAT3/Marker+p-STAT3.jpg]

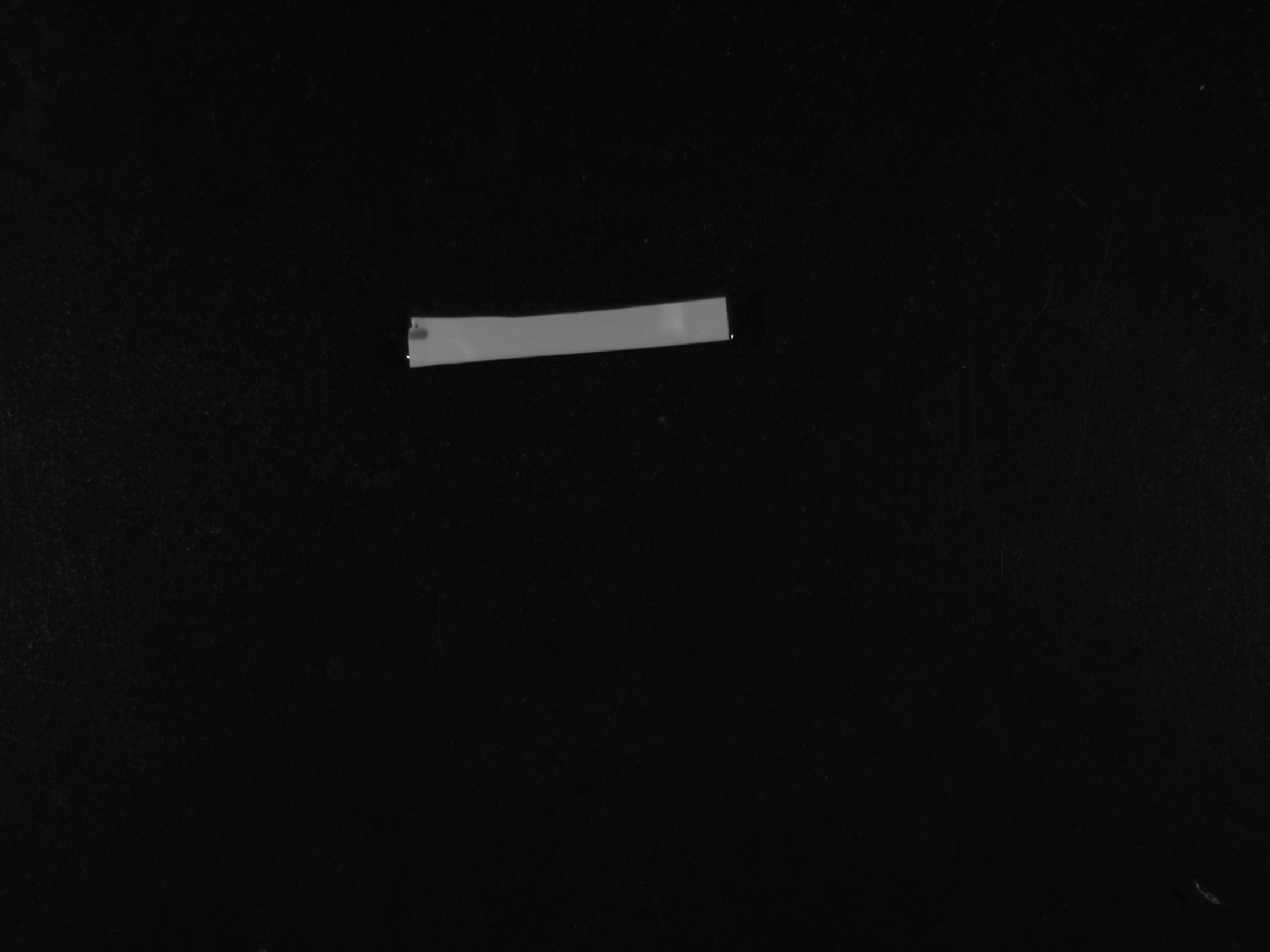

Supplement: Original Images for Blots.zip [file YRER_A_2313366_SM3875.zip › Original Images for Blots/Figure 4/Figure 4B/ERK signaling pathway/p-STAT3/Marker.jpg]

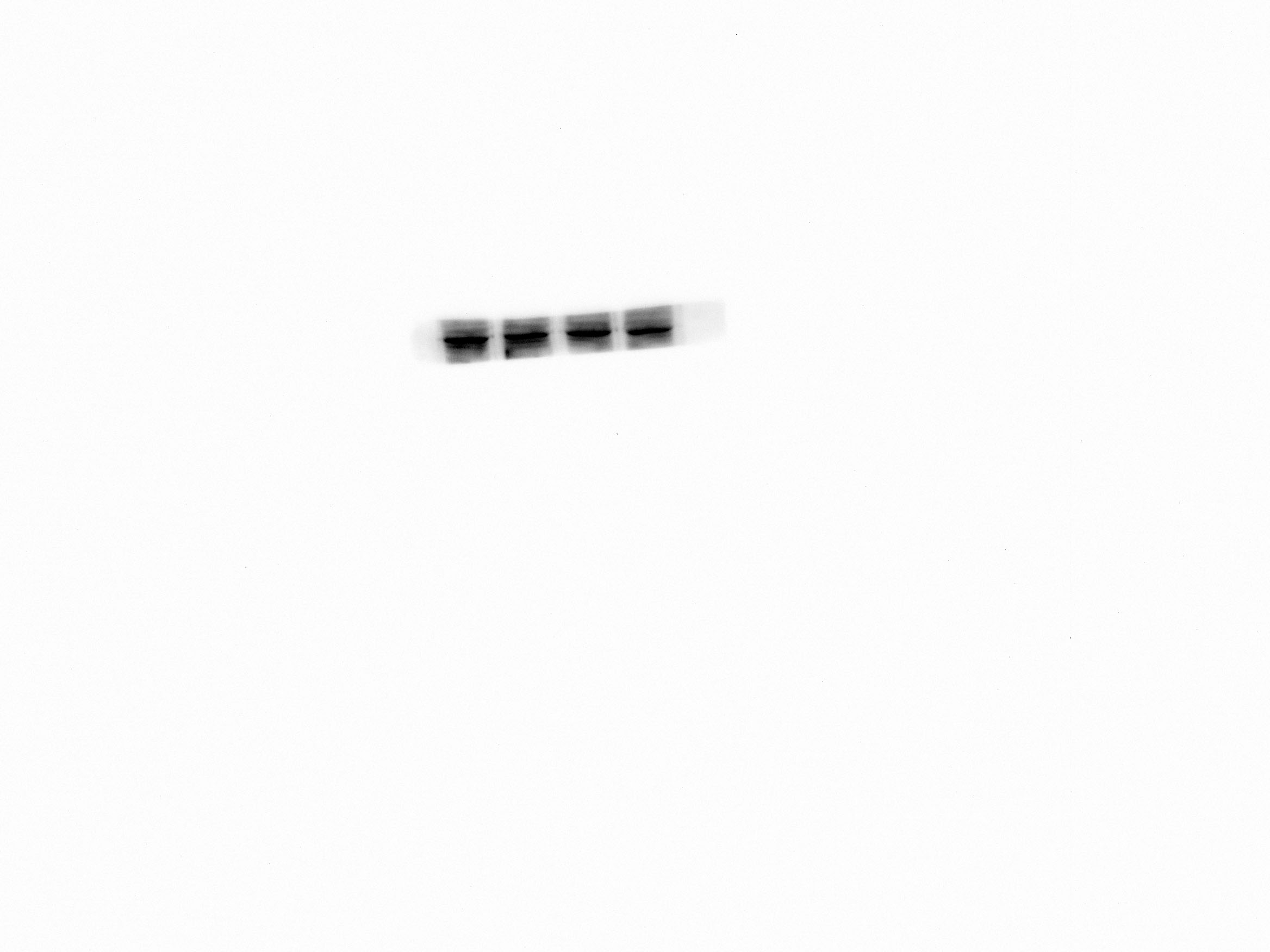

Supplement: Original Images for Blots.zip [file YRER_A_2313366_SM3875.zip › Original Images for Blots/Figure 4/Figure 4B/ERK signaling pathway/p-STAT3/p-STAT3.jpg]

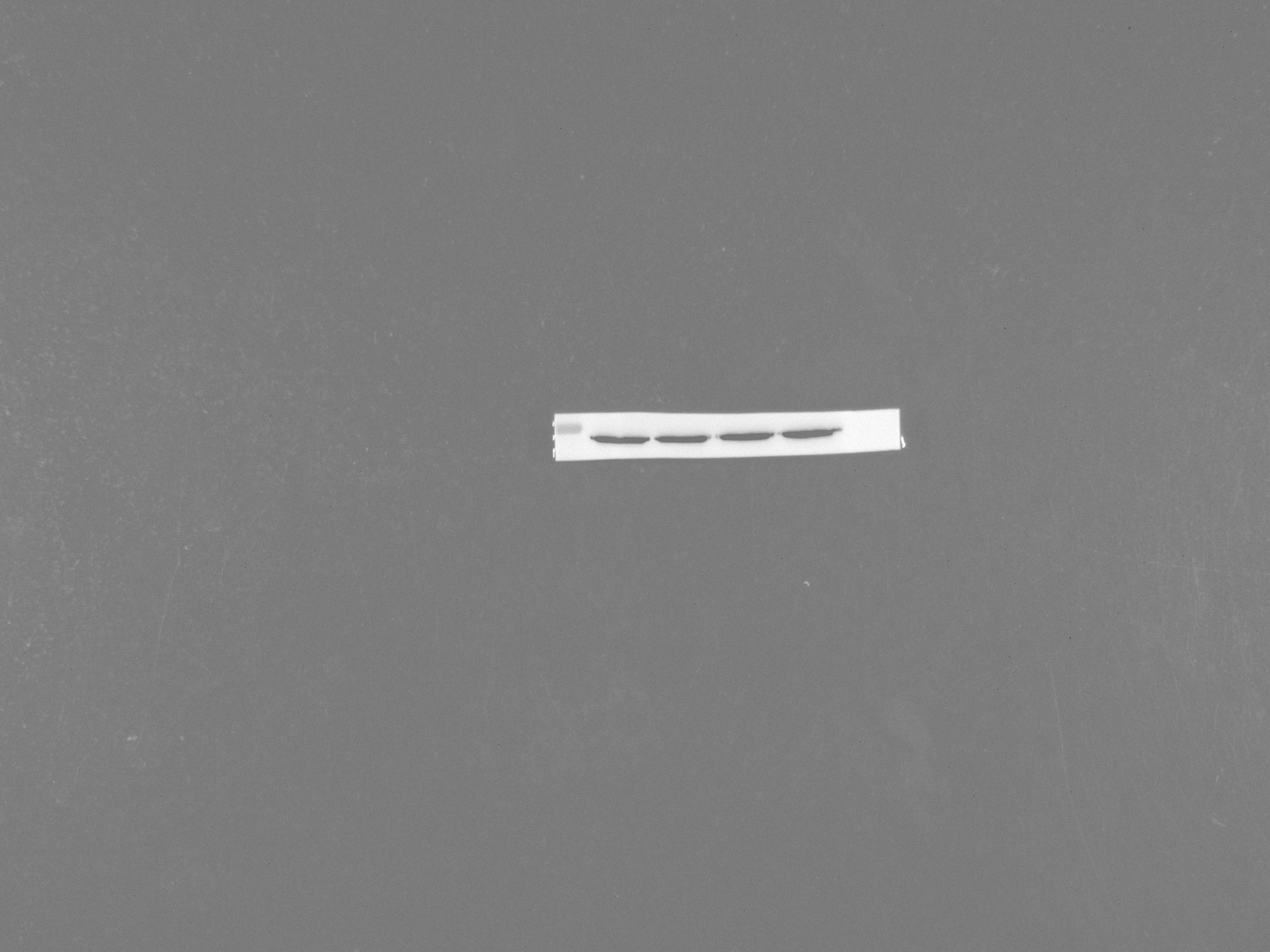

Supplement: Original Images for Blots.zip [file YRER_A_2313366_SM3875.zip › Original Images for Blots/Figure 4/Figure 4B/ERK signaling pathway/STAT3/Marker+STAT3.jpg]

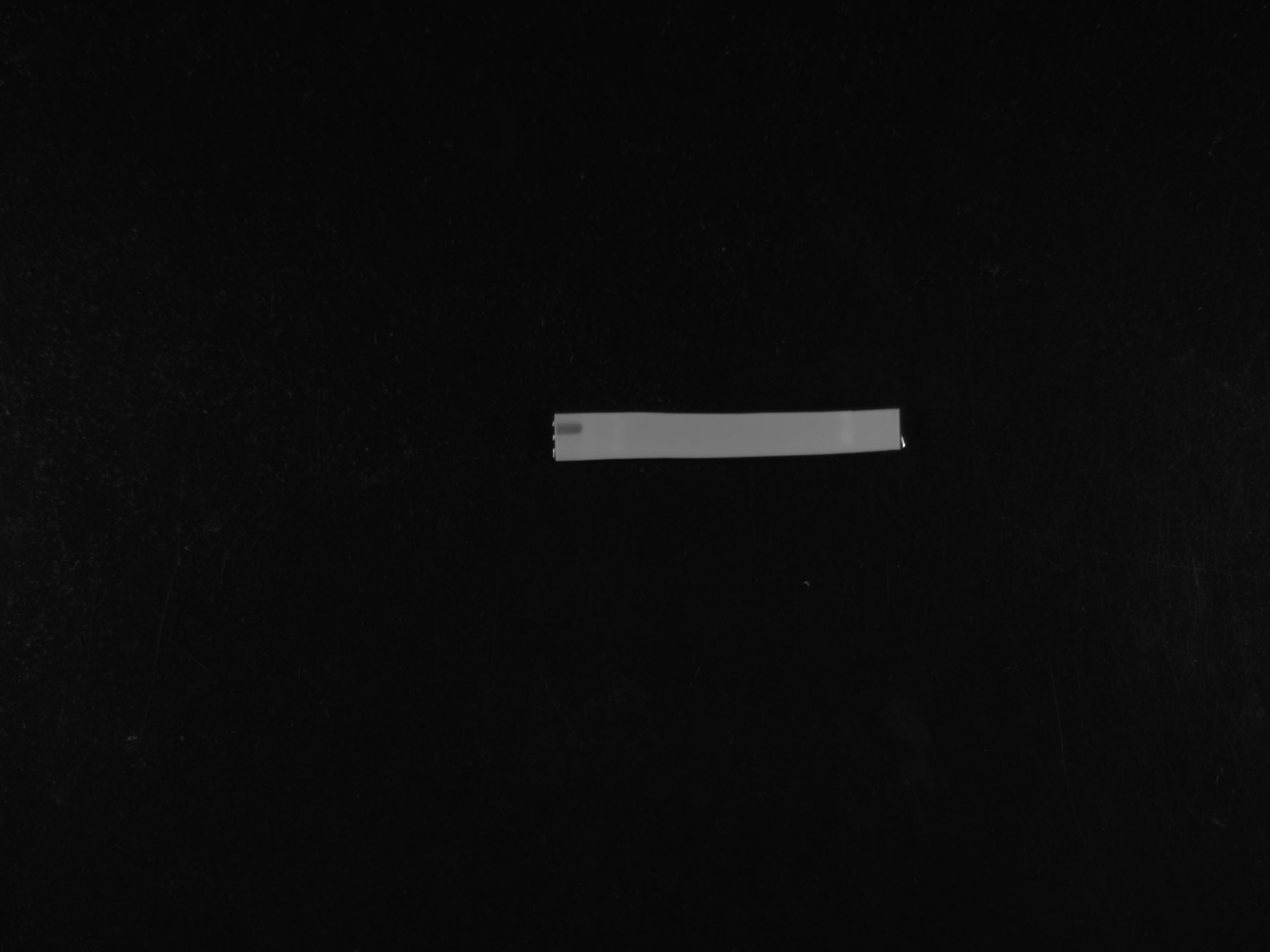

Supplement: Original Images for Blots.zip [file YRER_A_2313366_SM3875.zip › Original Images for Blots/Figure 4/Figure 4B/ERK signaling pathway/STAT3/Marker.jpg]

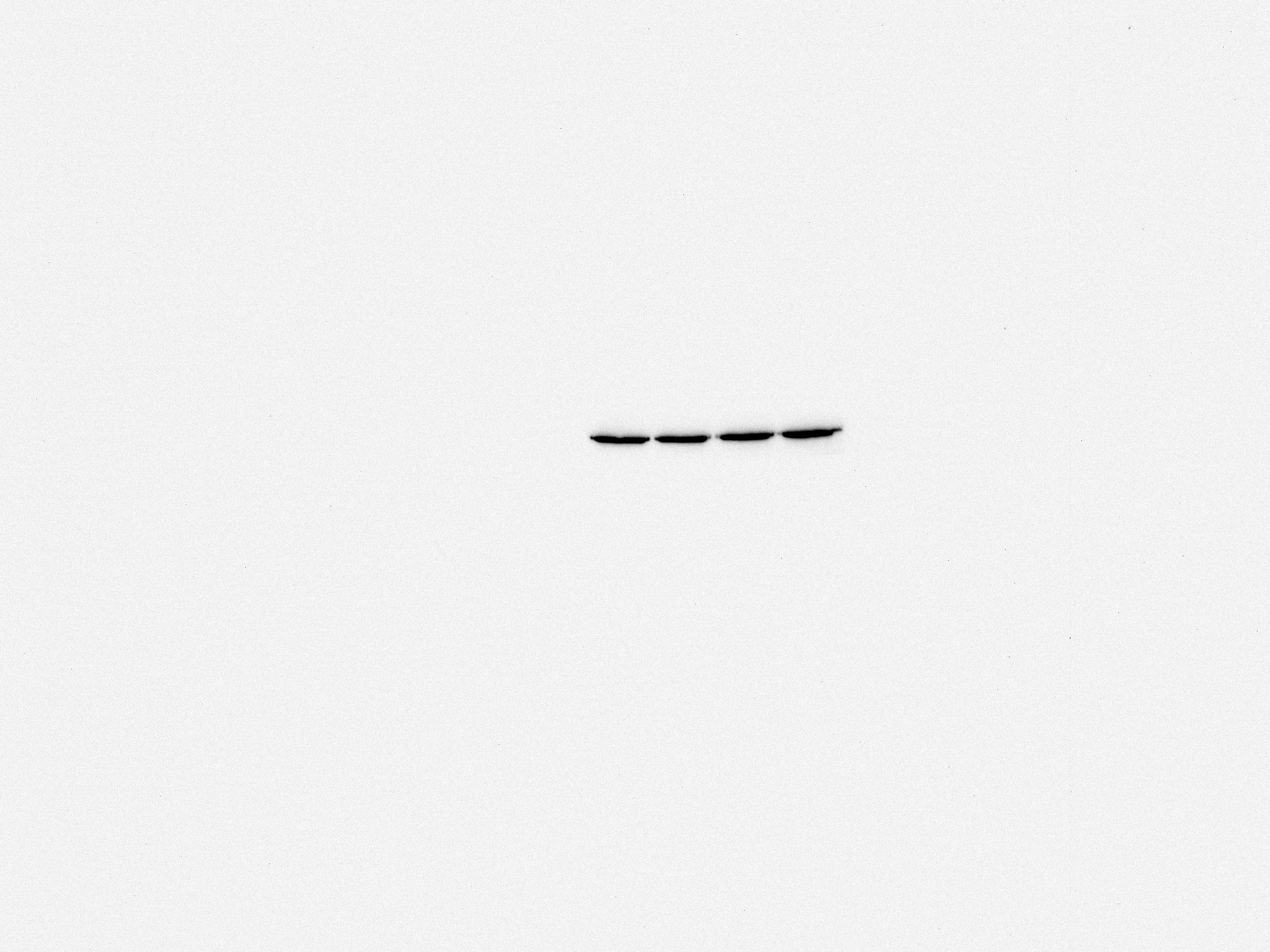

Supplement: Original Images for Blots.zip [file YRER_A_2313366_SM3875.zip › Original Images for Blots/Figure 4/Figure 4B/ERK signaling pathway/STAT3/STAT3.jpg]

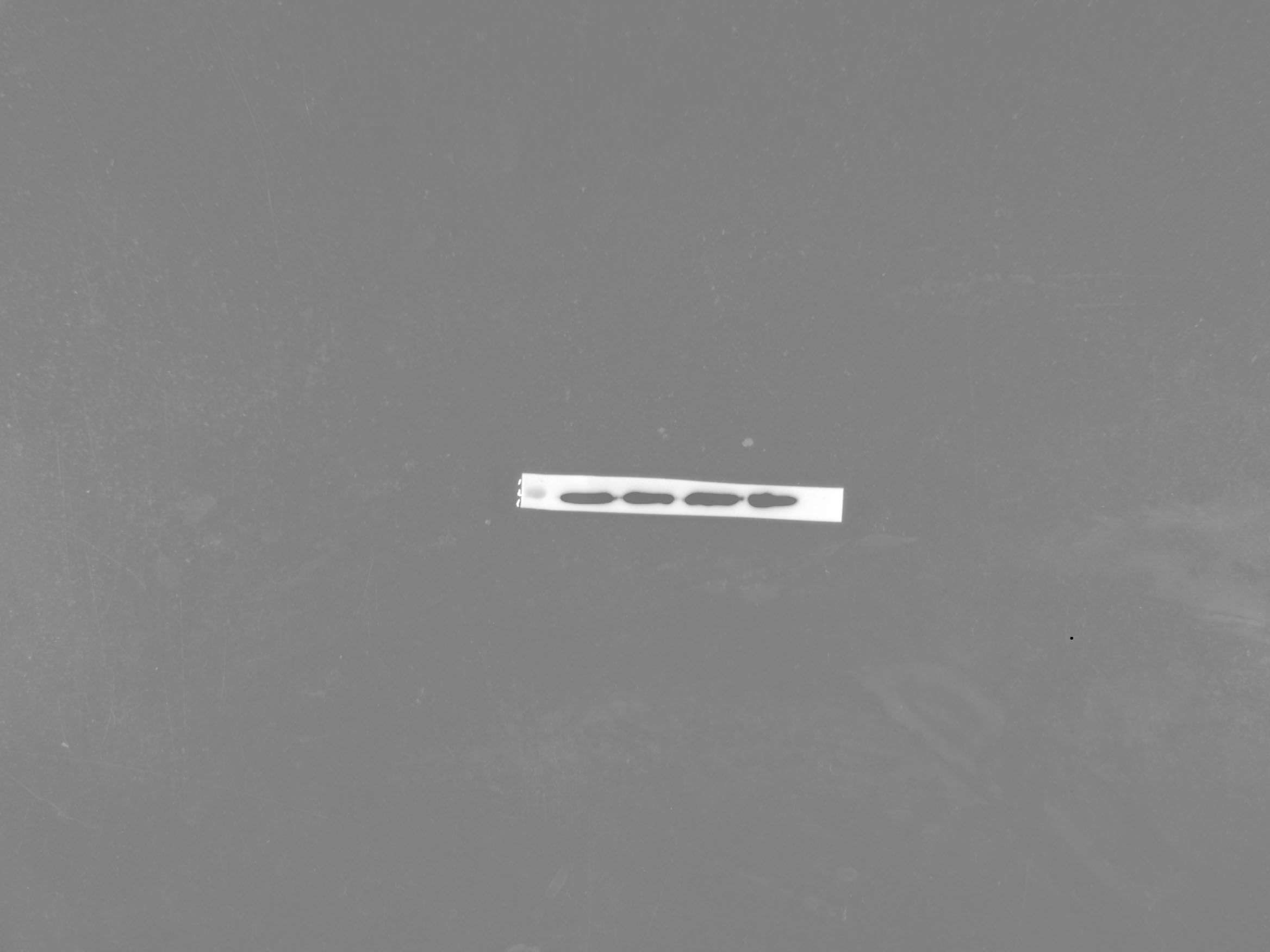

Supplement: Original Images for Blots.zip [file YRER_A_2313366_SM3875.zip › Original Images for Blots/Figure 4/Figure 4B/ERK signaling pathway/α-tubulin/Marker+α-tubulin.jpg]

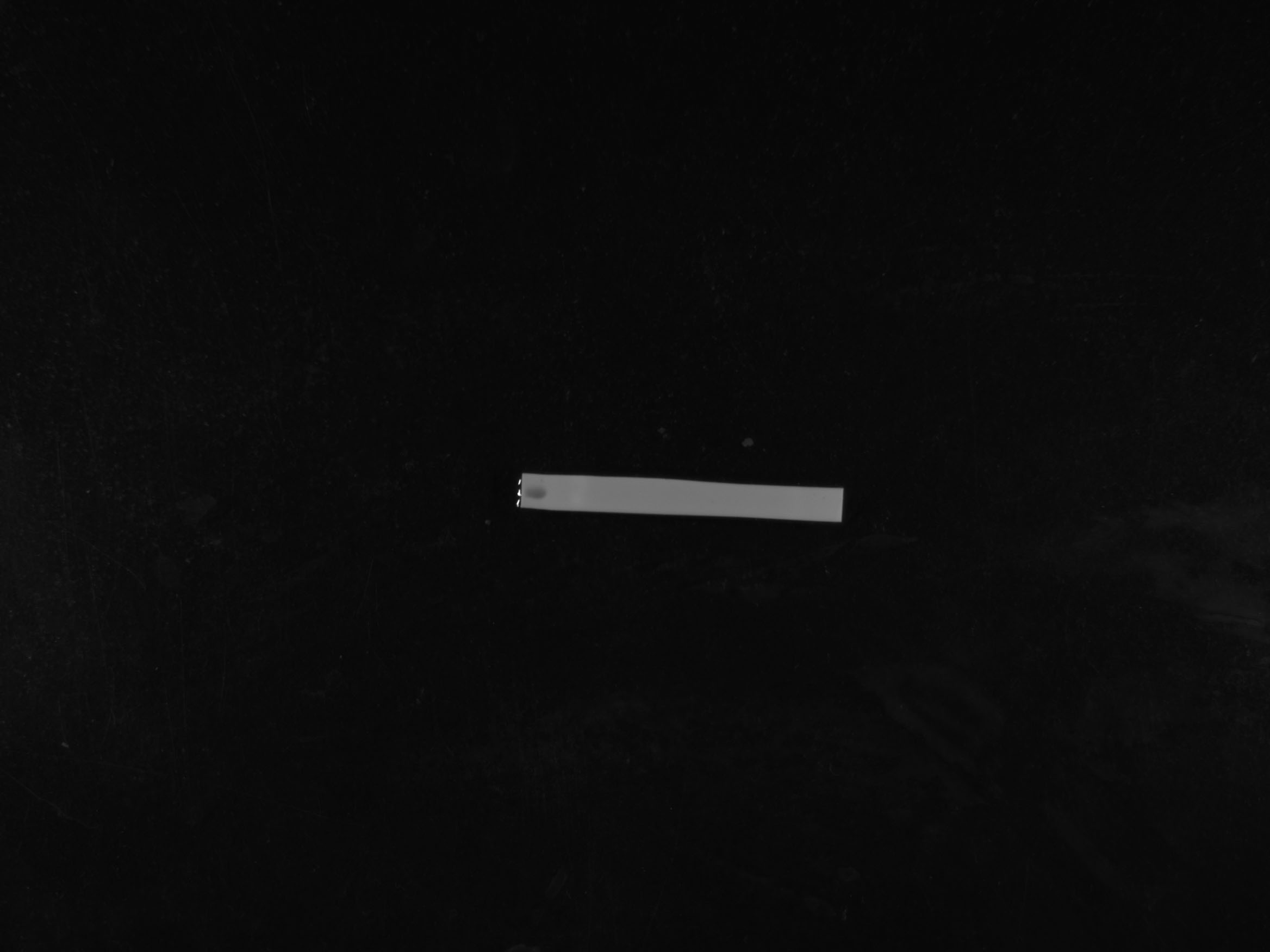

Supplement: Original Images for Blots.zip [file YRER_A_2313366_SM3875.zip › Original Images for Blots/Figure 4/Figure 4B/ERK signaling pathway/α-tubulin/Marker.jpg]

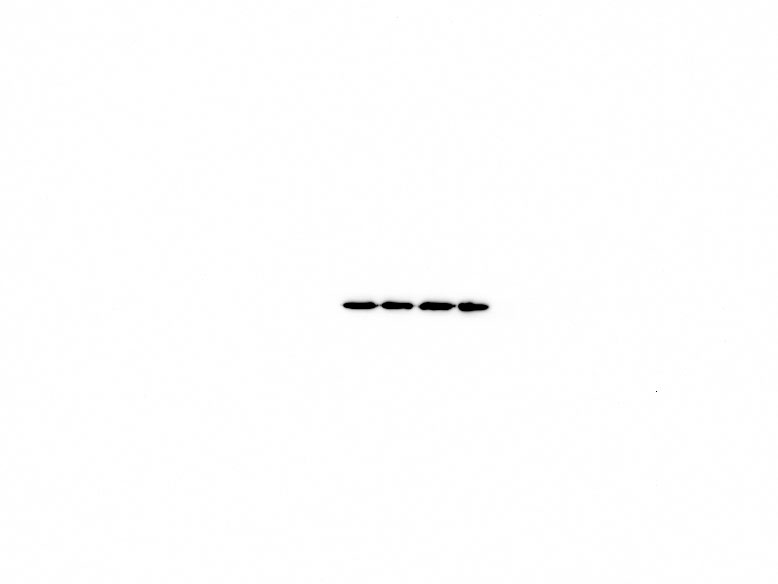

Supplement: Original Images for Blots.zip [file YRER_A_2313366_SM3875.zip › Original Images for Blots/Figure 4/Figure 4B/ERK signaling pathway/α-tubulin/α-tubulin.jpg]

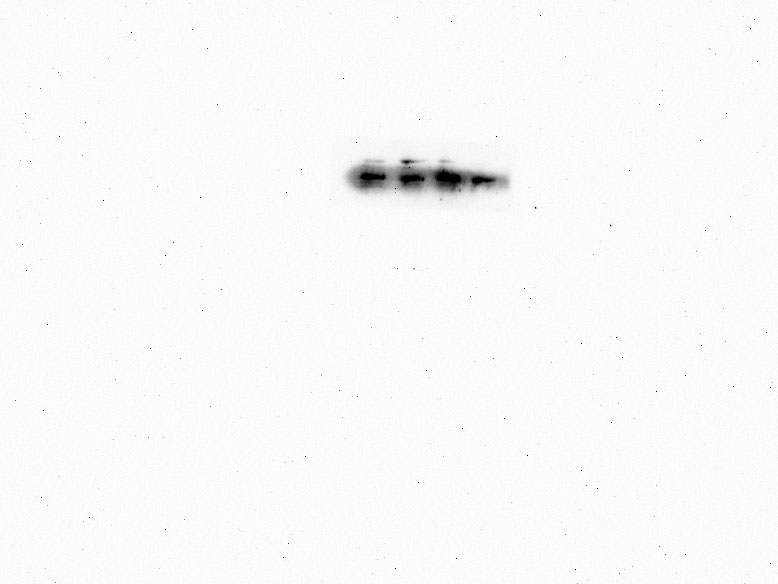

Supplement: Original Images for Blots.zip [file YRER_A_2313366_SM3875.zip › Original Images for Blots/Figure 4/Figure 4B/JNK signaling pathway/cle-caspase-3/cle-caspase-3.jpg]

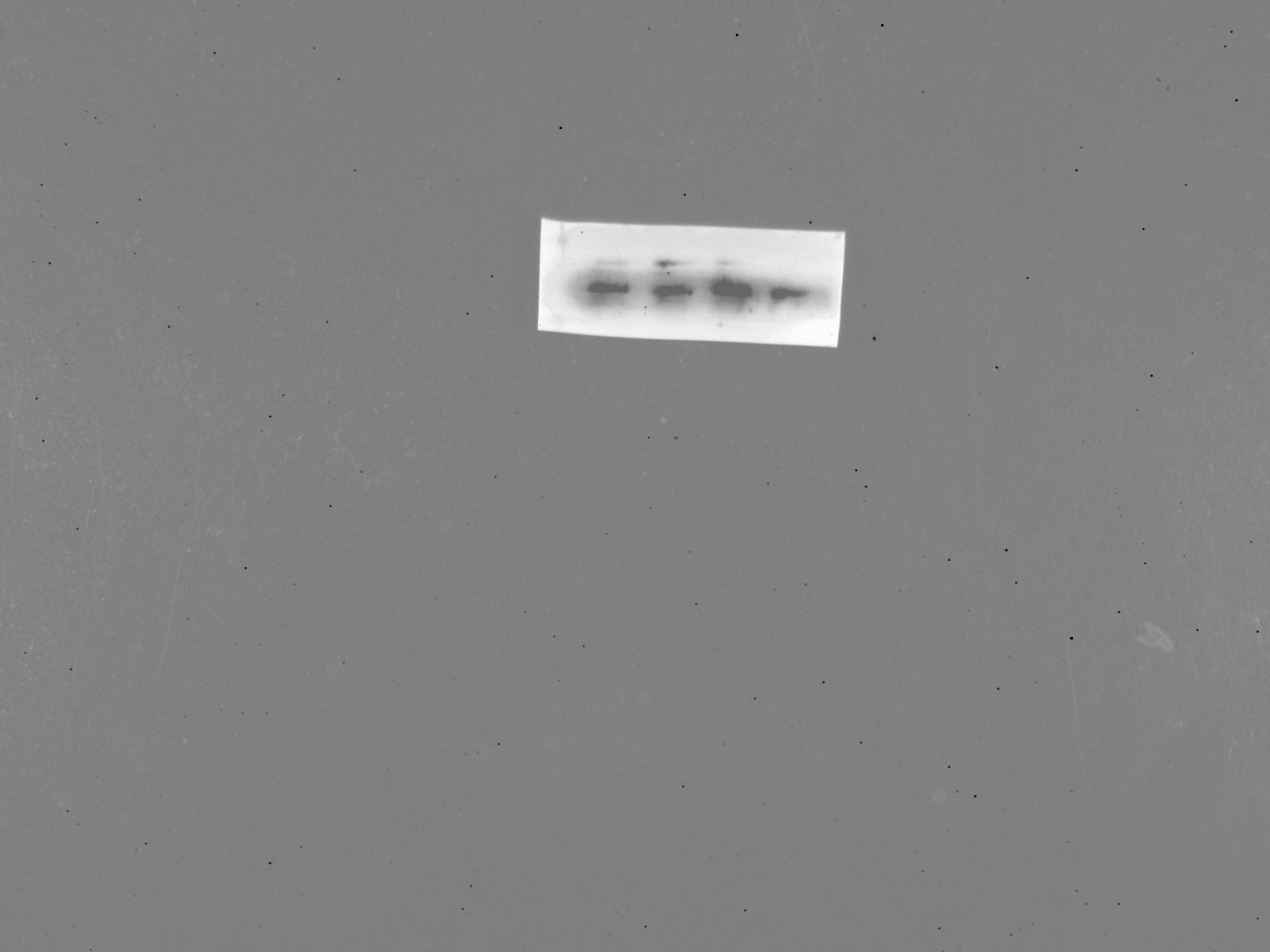

Supplement: Original Images for Blots.zip [file YRER_A_2313366_SM3875.zip › Original Images for Blots/Figure 4/Figure 4B/JNK signaling pathway/cle-caspase-3/Marker+cle-caspase-3.jpg]

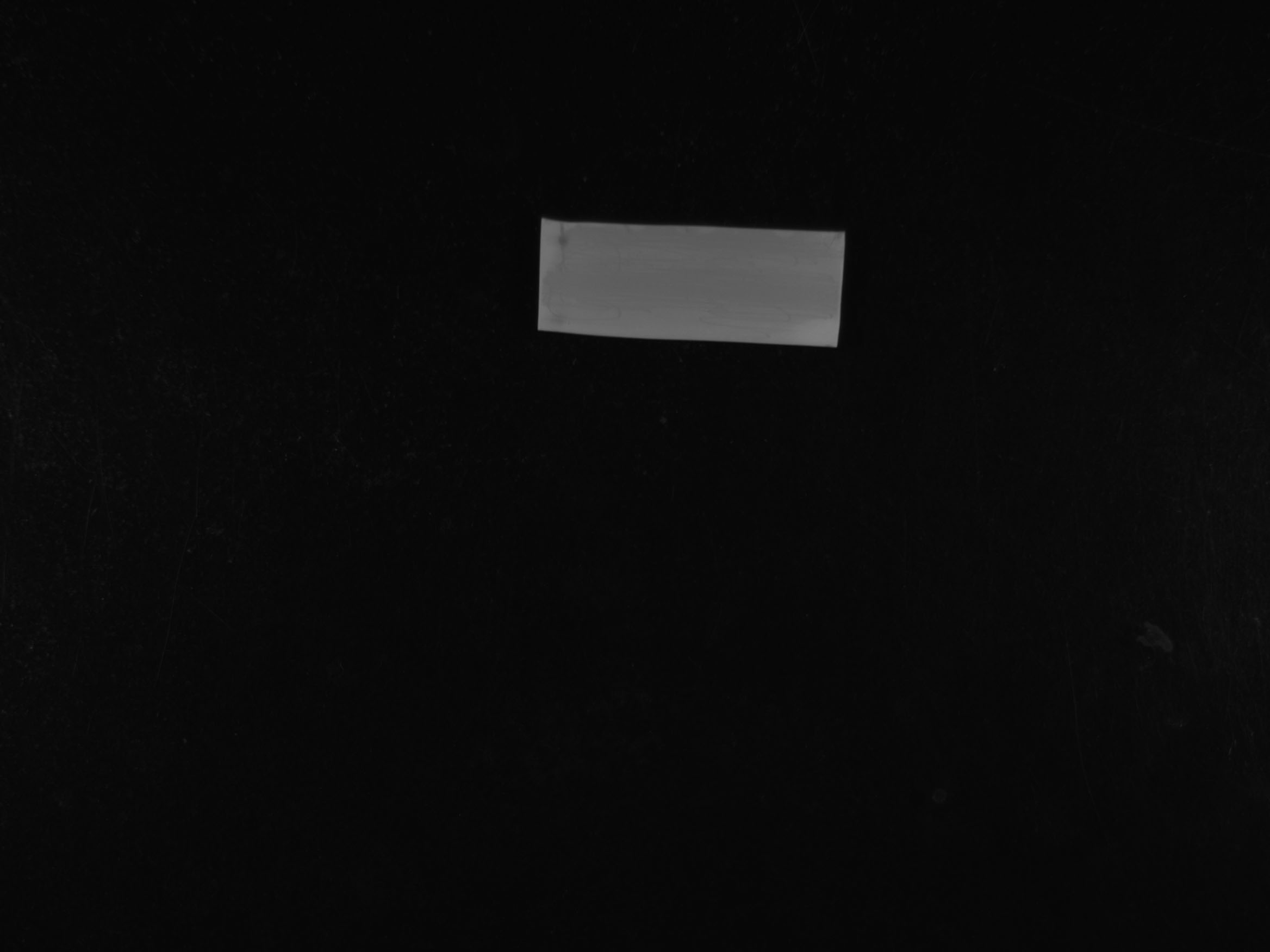

Supplement: Original Images for Blots.zip [file YRER_A_2313366_SM3875.zip › Original Images for Blots/Figure 4/Figure 4B/JNK signaling pathway/cle-caspase-3/Marker.jpg]

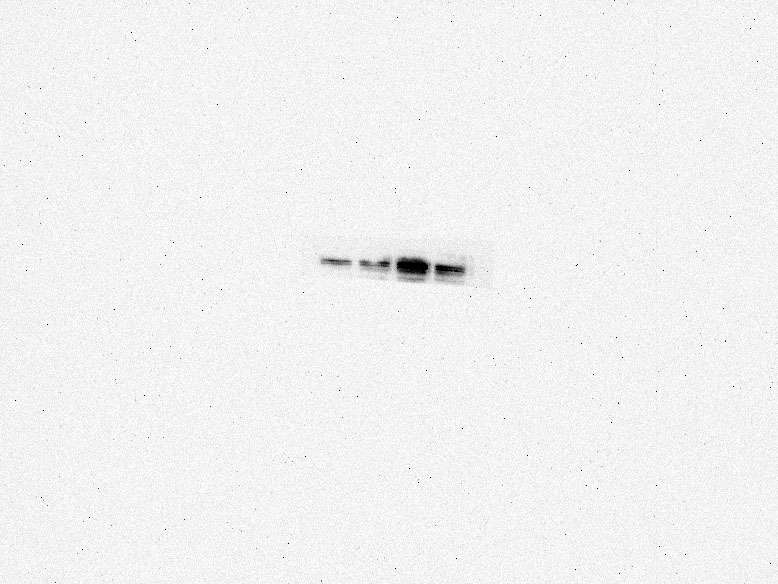

Supplement: Original Images for Blots.zip [file YRER_A_2313366_SM3875.zip › Original Images for Blots/Figure 4/Figure 4B/JNK signaling pathway/cle-PARP/cle-PARP.jpg]

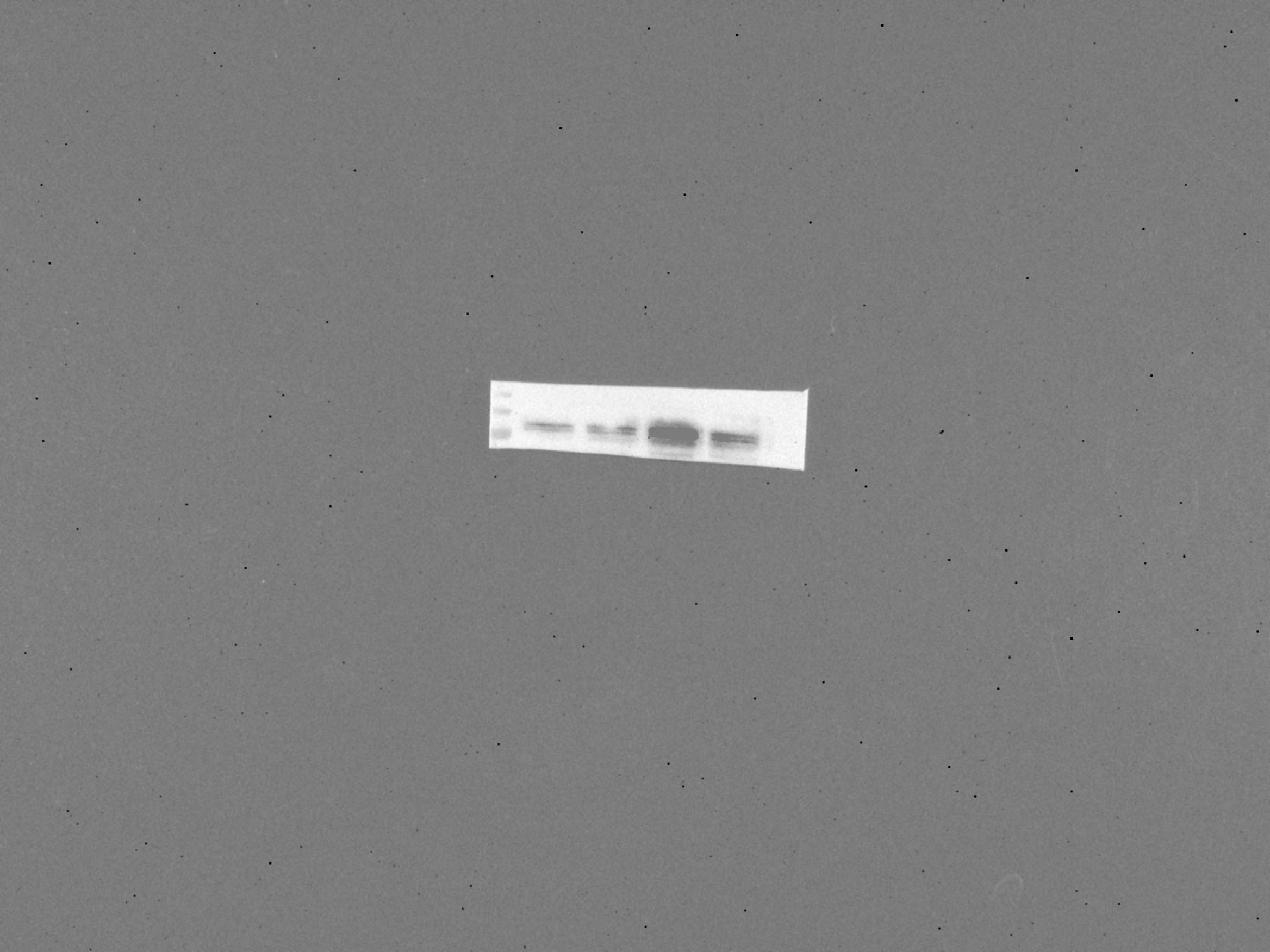

Supplement: Original Images for Blots.zip [file YRER_A_2313366_SM3875.zip › Original Images for Blots/Figure 4/Figure 4B/JNK signaling pathway/cle-PARP/Marker+cle-PARP.jpg]

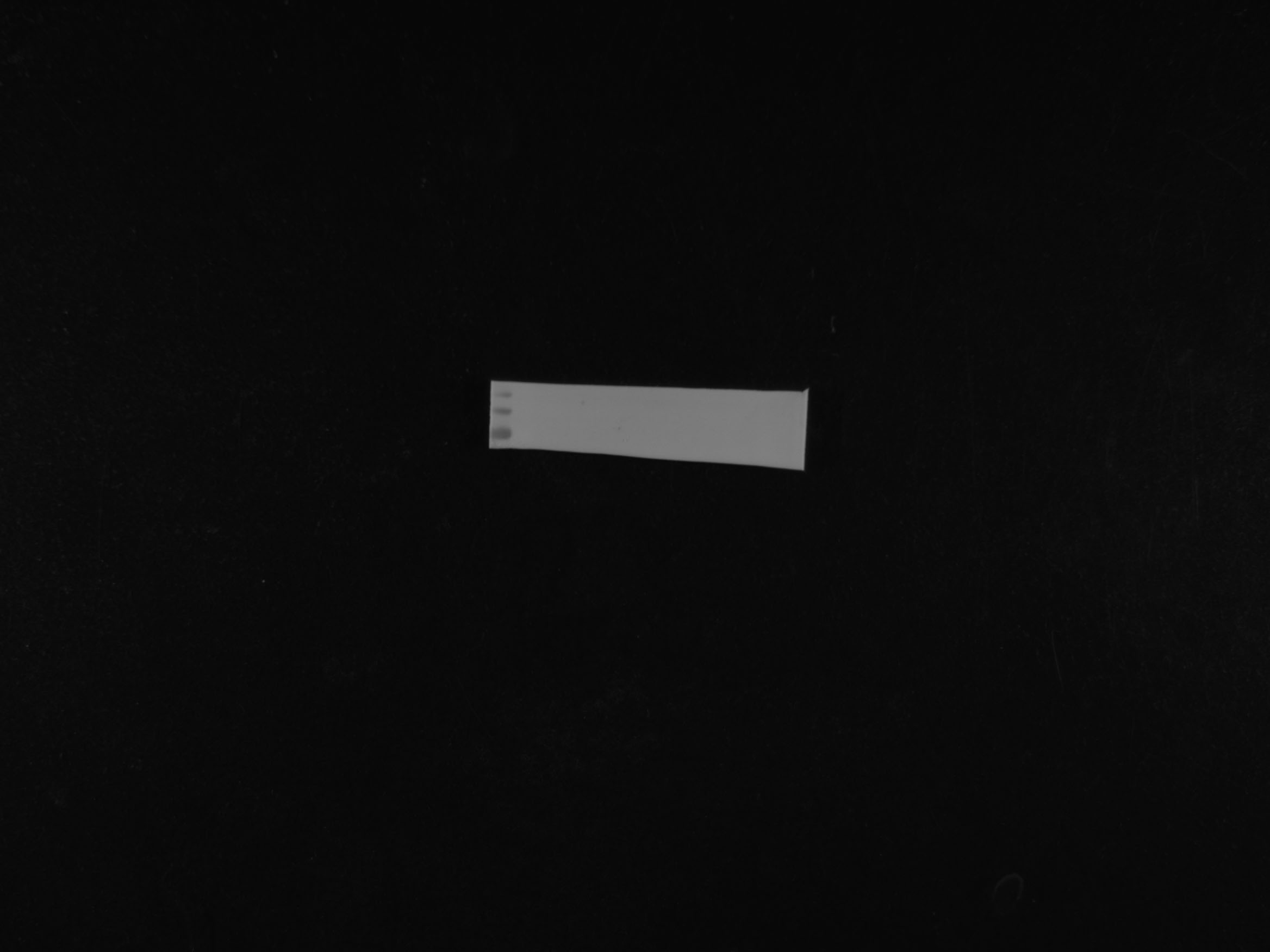

Supplement: Original Images for Blots.zip [file YRER_A_2313366_SM3875.zip › Original Images for Blots/Figure 4/Figure 4B/JNK signaling pathway/cle-PARP/Marker.jpg]

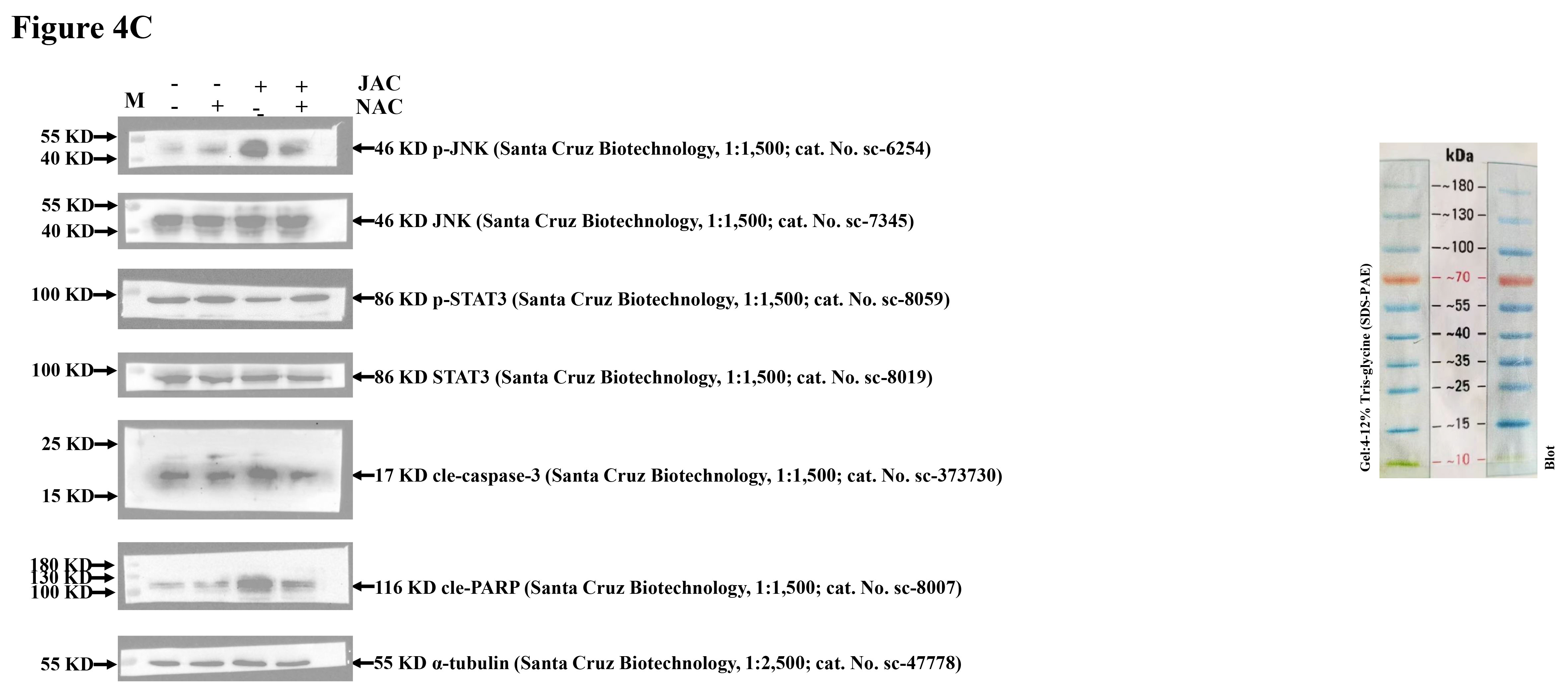

Supplement: Original Images for Blots.zip [file YRER_A_2313366_SM3875.zip › Original Images for Blots/Figure 4/Figure 4B/JNK signaling pathway/JNK signaling pathway.jpg]

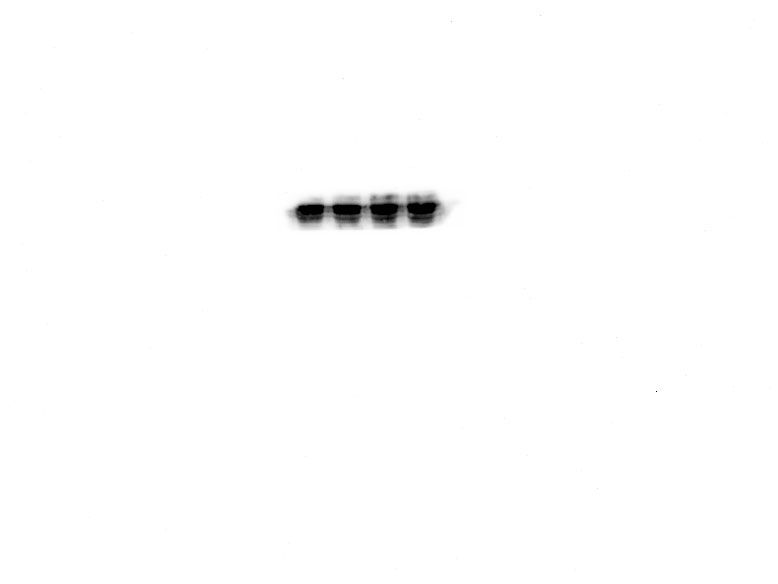

Supplement: Original Images for Blots.zip [file YRER_A_2313366_SM3875.zip › Original Images for Blots/Figure 4/Figure 4B/JNK signaling pathway/JNK/JNK.jpg]

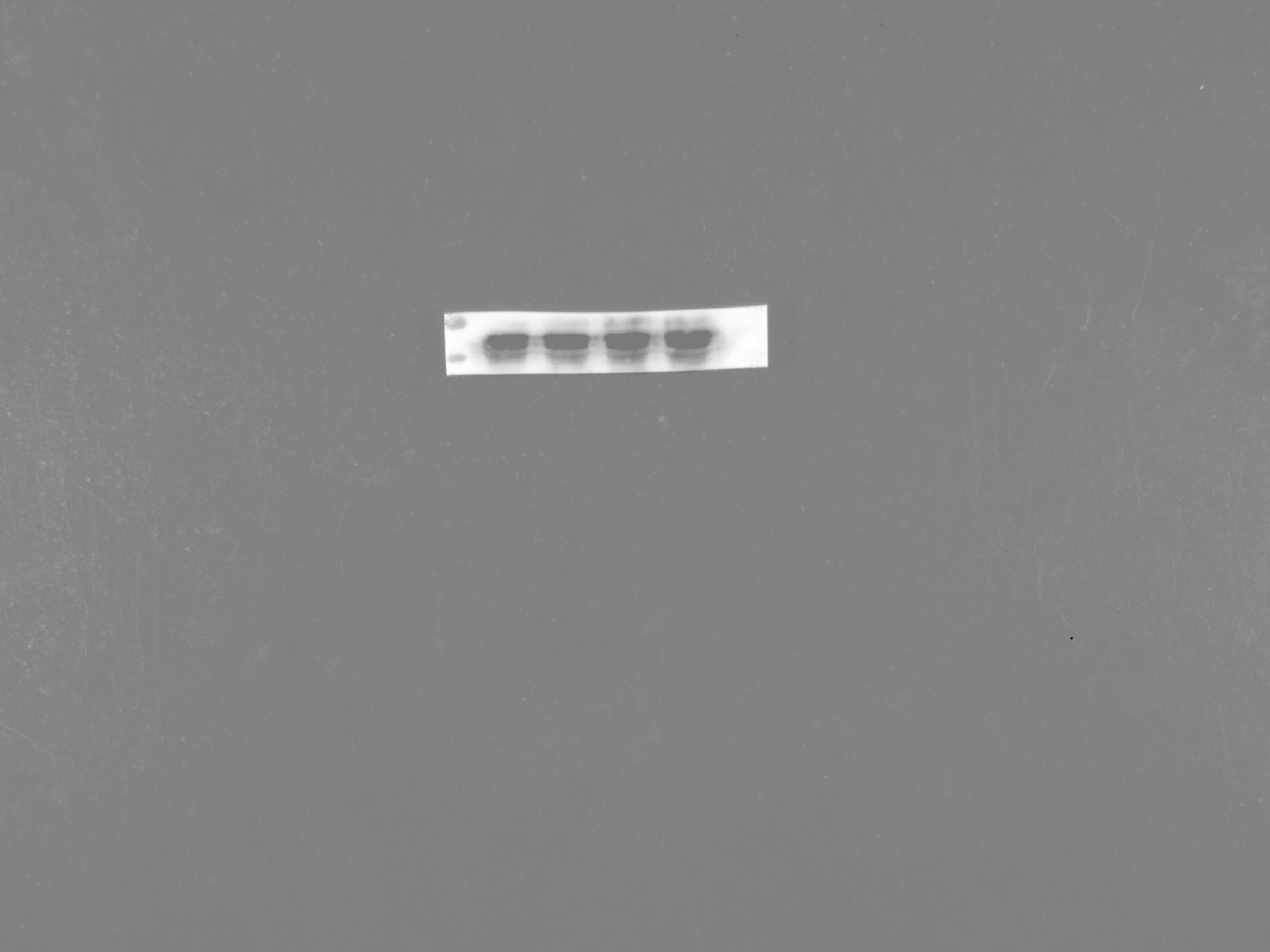

Supplement: Original Images for Blots.zip [file YRER_A_2313366_SM3875.zip › Original Images for Blots/Figure 4/Figure 4B/JNK signaling pathway/JNK/Marker+JNK.jpg]

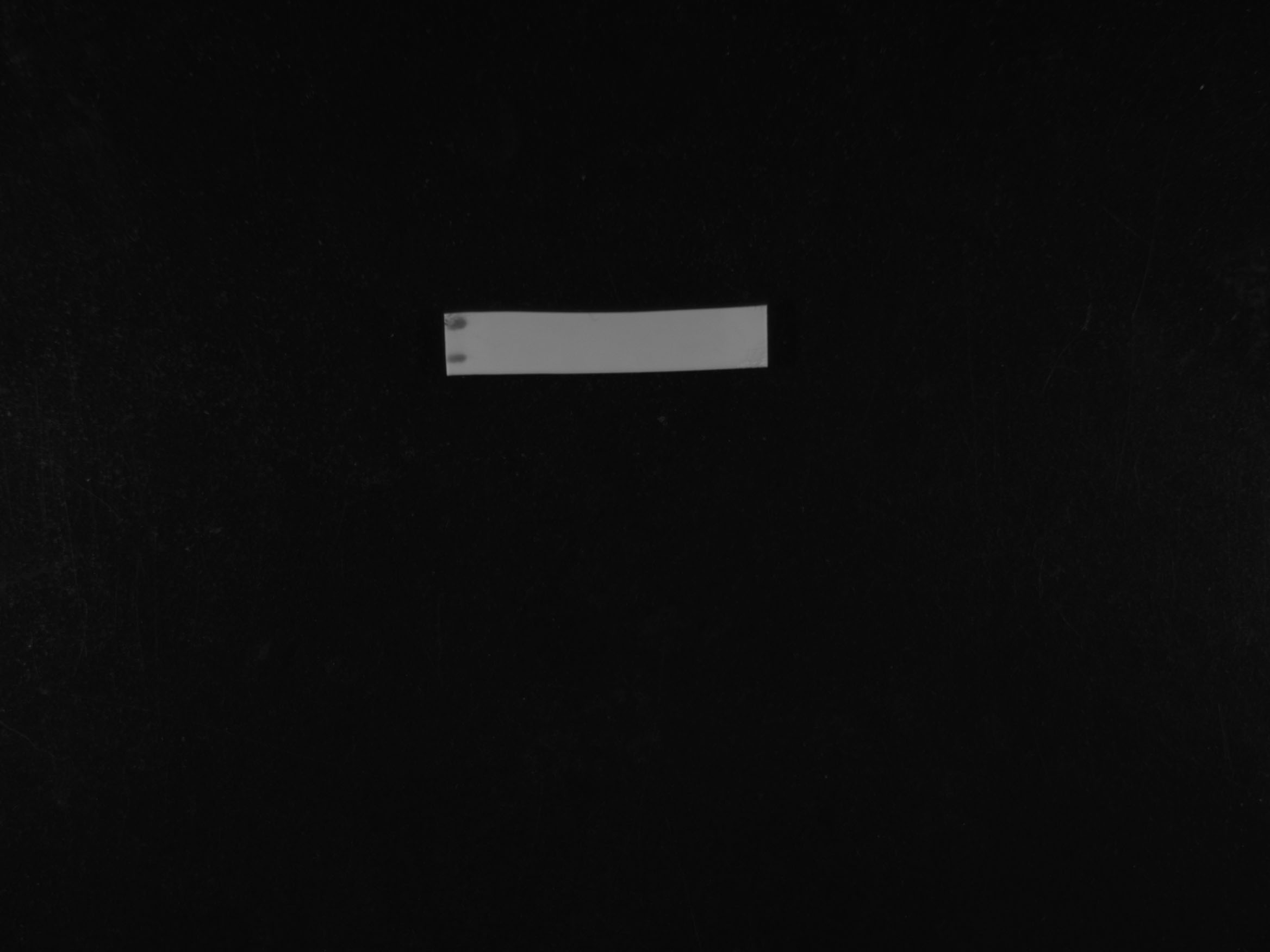

Supplement: Original Images for Blots.zip [file YRER_A_2313366_SM3875.zip › Original Images for Blots/Figure 4/Figure 4B/JNK signaling pathway/JNK/Marker.jpg]
